# Investigating the influence of physiologically relevant hydrostatic pressure on CHO cell batch culture

**DOI:** 10.1038/s41598-020-80576-8

**Published:** 2021-01-08

**Authors:** Menglin Shang, Taehong Kwon, Jean-Francois P. Hamel, Chwee Teck Lim, Bee Luan Khoo, Jongyoon Han

**Affiliations:** 1 Critical Analytics for Manufacturing Personalized-Medicine (CAMP) IRG, Singapore-MIT Alliance for Research and Technology (SMART) Centre, Singapore, Singapore; 2 Mechanobiology Institute, National University of Singapore, Singapore, Singapore; 3 Department of Biomedical Engineering, National University of Singapore, 7 Engineering Drive 1, Singapore, 117574 Singapore; 4 Department of Mechanical Engineering, National University of Singapore, Singapore, Singapore; 5 Department of Biomedical Engineering, City University of Hong Kong, Kowloon Tong, Hong Kong; 6 Department of Electrical Engineering and Computer Science, Department of Biological Engineering, Massachusetts Institute of Technology, Cambridge, MA USA; 7 Department of Chemical Engineering, Massachusetts Institute of Technology, Cambridge, MA USA; 8 Research Laboratory of Electronics, Massachusetts Institute of Technology, 50 Vassar St, Cambridge, MA 02139 USA; 9 Institute for Health Innovation and Technology, 14 Medical Drive, Singapore, 117599 Singapore

**Keywords:** Biophysical chemistry, Cell growth, Biotechnology

## Abstract

Chinese hamster ovary (CHO) cells have been the most commonly used mammalian host for large-scale commercial production of therapeutic proteins, such as monoclonal antibodies. Enhancement of productivity of these CHO cells is one of the top priorities in the biopharmaceutical industry to reduce manufacturing cost. Although there are many different methods (e.g. temperature, pH, feed) to improve protein production in CHO cells, the role of physiologically relevant hydrostatic pressure in CHO cell culture has not been reported yet. In this study, four different hydrostatic pressures (0, 30, 60, and 90 mmHg) were applied to batch CHO cells, and their cell growth/metabolism and IgG_1_ production were examined. Our results indicate that hydrostatic pressure can increase the maximum cell concentration by up to 50%. Moreover, overall IgG_1_ concentration on Day 5 showed that 30 mmHg pressure can increase IgG_1_ production by 26%. The percentage of non-disulphide-linked antibody aggregates had no significant change under pressure. Besides, no significant difference was observed between 30 mmHg and no pressure conditions in terms of cell clumping formation. All these findings are important for the optimization of fed-batch or perfusion culture for directing cell growth and improving antibody production.

## Introduction

Since the first therapeutic monoclonal antibody product, Orthoclone OKT3, was approved in 1986 for the prevention of kidney transplant rejection^1^, the biopharmaceutical industry has grown rapidly. The annual global market of protein biopharmaceuticals has expanded from $99 billion in the year 2009 to $188 billion in 2017^2–4^. By 2018, the total number of approved monoclonal antibody products in the US and Europe for the treatment of a variety of diseases had reached 169^4^. Recombinant Chinese hamster ovary (CHO) cell is the most commonly used mammalian host for large-scale commercial production of therapeutic proteins^5^. Among the top ten best-selling protein biopharmaceuticals in 2016, five were produced using CHO cells^6,7^.

To meet the rapidly growing demand for therapeutic proteins and reduce manufacturing cost, several strategies have been developed to improve biopharmaceutical productions in CHO cells. In general, these approaches can be categorized into two groups: cell engineering and bioprocess optimization^8^. Cell engineering aims to improve the expression of the target gene and protein yield from the cells^8–10^. It is typically achieved by introducing knock-out or silencing specific genes to overexpress beneficial genes or repress disadvantageous genes. For example, plasmid transfection of miR-23 into CHO cells was shown to increase the productivity of CHO cells by two folds without affecting its growth^11^. Bioprocess optimization involves many aspects such as bioreactor optimization (culture mode, pH, temperature, etc.) and medium modification to better support the CHO cells’ proliferation and protein production^8–10^. Compared with batch and fed-batch culture, CHO cells can reach higher cell concentration (> 50 × 10^6^ cells/mL) in perfusion culture systems where fresh culture medium is continuously supplied into the bioreactor and toxic metabolites are removed from the bioreactor. As a result, overall volumetric monoclonal antibody production can be significantly improved in such perfusion culture systems^12^.

As another way of bioprocess optimization, hydrostatic pressure can be considered. As a fundamental physical stimulus present in vivo, hydrostatic pressure influences cell behaviours in many parts of the human body, such as cartilage, eyes and vasculature^13,14^. For example, because of higher blood pressure in arteries, arteries have thicker blood vessel walls and less compliance than veins^15,16^. Optical nerves can also be damaged by high intraocular pressure, which is the major cause of glaucoma^17^. In ex vivo studies, it was also shown that hydrostatic pressure could change cell proliferation^18,19^, migration^20^ and affect folding, dynamics and interactions of proteins^21,22^. For example, a 1 Hz cyclic pressure of 90/70 mmHg was shown to increase mesenchymal stem cells (MSCs) proliferation by approximately six times through a 5-day experiment^19^. Until now, there have been three studies investigating the influence of static pressure on CHO cells. They found that 0.8 MPa (~ 6000 mmHg above atmosphere pressure) static pressure can enhance human granulocyte–macrophage stimulating factor (hGM-CSF) production in adherent CHO cells and also lower its intracellular pH from 6.60 to 5.24^23–25^. All these studies tested pressure of ~ 6000 mmHg, which was significantly higher than the hydrostatic pressure in vivo (typically less than 120 mmHg). Moreover, their findings were based on 2D adherent culture where cell behavior can be different from suspension culture. For example, at the same osmotic pressure of 450 mOsm/kg, the specific yield of tissue plasminogen activator (tPA) was maximized in suspension CHO cell culture but only reached 50% of its maximal value in adherent culture^26^.

By constructing a pressurized batch culture system, we found that hydrostatic pressure of 30–90 mmHg slowed down initial CHO cell proliferation but increased the maximum cell concentration at the end phase of the culture. It was also shown that 30 mmHg culture condition can increase overall antibody production by 26%. At this same condition, the non-disulphide-linked antibody aggregates percentage did not increase. However, more cell aggregation was observed when the pressure was greater than 60 mmHg, and it should be considered as a side effect of pressurized culture. Overall, an optimum hydrostatic pressure of 30 mmHg was beneficial for batch CHO cell culture, because it increased both maximum cell concentration and antibody production. Most importantly, this method of applying hydrostatic pressure can easily be adopted in many state-of-the-art cell culture systems, to either enhance antibody production or increase maximum cell concentration.

## Materials and methods

### Cell culture

CHO-DG44 cell line producing human IgG_1_ against CD40 ligand was given by Biogen Idec (Cambridge, MA, USA). It was cultured using CD OptiCHO Medium (Gibco) without additional growth factor or antibiotics. It was maintained in a 5% CO_2_ incubator at 37 °C for up to 7 days without medium change. Initial seeding concentration was maintained at about 0.35 million cells/ml for each passage.

### Device setup

**Figure 1:**
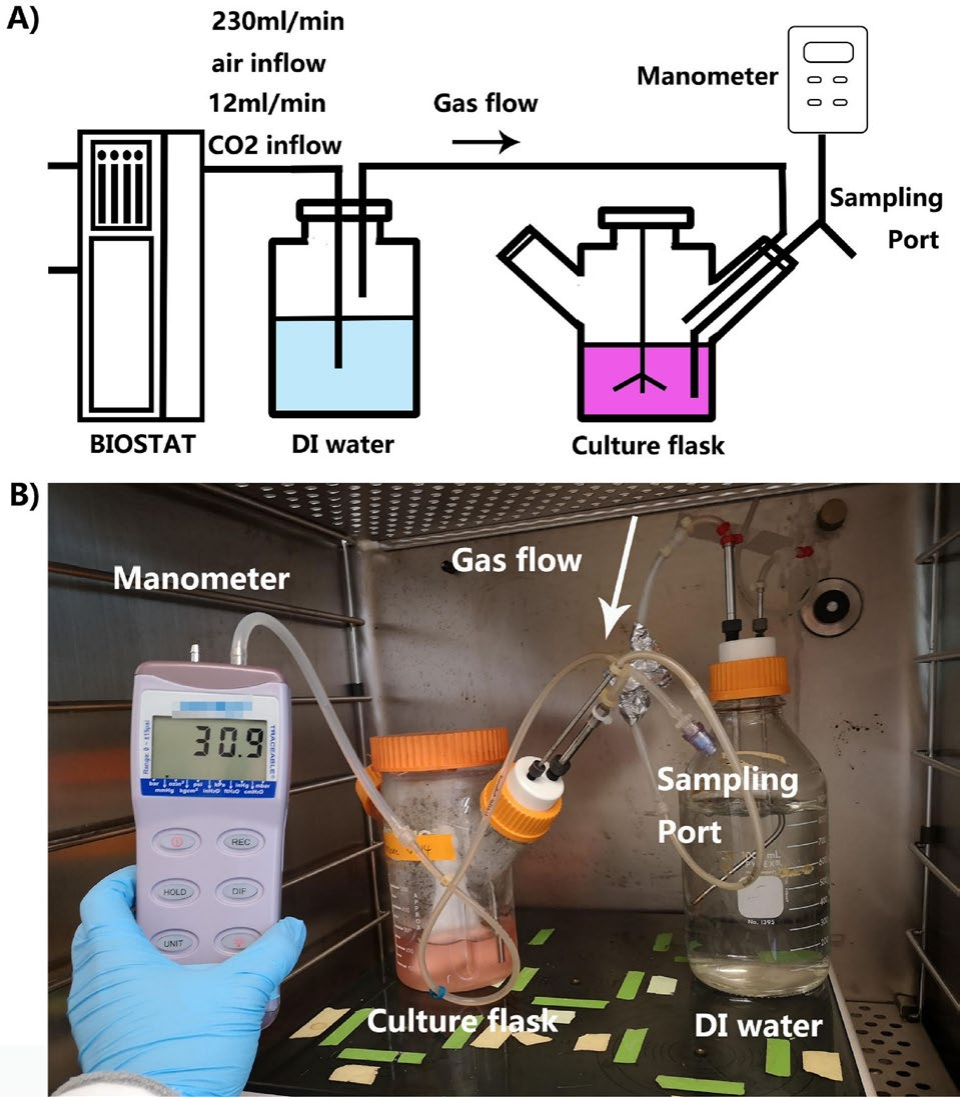
Experimental device design. (**A**) Schematic of pressurized CHO culture system. (**B**) Photo of actual setup in a standard CO_2_ incubator.

Hydrostatic pressure in the culture flask was established by pumping gas into a partially air-tight spinner flask as shown in Fig. 1A. The flow rate of input gas was controlled by a commercial bioreactor controller (BIOSTAT A plus, Sartorius, USA). Before entering the culture flask, mixed input gas was humidified through a DI water tank to prevent the evaporation of culture media. Hydrostatic pressure in the culture flask was controlled by adjusting the tightness of caps on the spinner flask, and it was continuously monitored by a manometer. The actual device setup was shown in Fig. 1B.

CHO cells were grown in a batch mode and seeded into the pressure culture system at 0.35 million viable cells/mL. The working volume of the pressure culture system was 250 mL. Subsequently, the individual parts of the pressure system were connected, and the tightness of caps was adjusted to obtain the desired hydrostatic pressure. CHO cells were cultured in the system for up to 7 days. Cell culture was sampled daily to measure viable cell concentration and cell diameter via an automated cell culture analyser (BioProfile FLEX2, Nova Biomedical, USA).

Four different pressure conditions, 0, 30, 60 and 90 mmHg, were applied to the batch culture. For each pressure condition, three independent experiments were performed and three measurements were performed for each experiment. Due to the limit in device availability, experiments of 0 and 30 mmHg conditions were conducted together using the same batch of cells. Likewise, 60 and 90 mmHg pressurized cultures were performed simultaneously. In total, 12 batch cultures were performed.

### Analytical methods

The automated cell culture analyser (BioProfile FLEX2, Nova Biomedical, USA) measured cell concentration, viability, average live-cell diameter, the partial pressure of oxygen and carbon dioxide, pH as well as the concentration of glucose, lactate and ammonium in the culture medium. There is an inbuilt programme in the automated cell analyser which identifies the boundary of cells based on phase-contrast images and measures cell diameters accordingly. Each measurement was repeated three times to reduce random errors caused by the human operation. Metabolite concentration change per cell was calculated using the following formula:

1 $$ Glucose\,consumption\,per\,cell_{day\,i} = \frac{{\left( {\left[ {Glucose} \right]_{day\,i} - \left[ {Glucose} \right]_{day\,i + 1} } \right)}}{{(\left[ {viable\,cell} \right]_{day\,i} + \left[ {viable\,cell} \right]_{day\,i + 1} )/2}} $$

2 $$ Lactate\,production\,per\,cell_{day\,i} = \frac{{\left( {\left[ {Lactate} \right]_{day\,i + 1} - \left[ {Lactete} \right]_{day\,i} } \right)}}{{(\left[ {viable\,cell} \right]_{day\,i} + \left[ {viable\,cell} \right]_{day\,i + 1} )/2}} $$

3 $$ Ammonium\,production\,per\,cell_{day\,i} = \frac{{\left( {\left[ {Ammonium} \right]_{day\,i + 1} - \left[ {Ammonium} \right]_{day\,i} } \right)}}{{(\left[ {viable\,cell} \right]_{day\,i} + \left[ {viable\,cell} \right]_{day\,i + 1} )/2}} $$

The images taken by the analyser were exported for cell cluster analysis using ImageJ^27^. The boundary of the cell cluster in the image was detected by adjusting its threshold level. Subsequently, the area of the cluster was measured by using the “analyse particles” function. Since the average cell diameter in this study is about 17 μm, the average area of a cell should be approximately 227 μm^2^. To ensure doubling cells are not included in our cluster count, we used a threshold area of 600 μm^2^ to identify cell clusters.

The culture sample was clarified with a syringe filter (0.2 µm pore size; 4612, Pall Laboratory, USA) for Immunoglobulin G (IgG_1_) analysis. Overall IgG_1_ level was measured using high-performance liquid chromatography (HPLC) with a protein A column (2,100,100, Thermo Fisher Scientific, USA). The IgG_1_ concentration of the sample was obtained using the area of IgG_1_ peak under the curve and a standard IgG_1_ concentration curve. The protein electrophoresis was performed using an analyzer (2100 Bioanalyzer System, Agilent, USA) with a labeling and denaturation kit (5067–1575, Agilent, USA) to quantify the percentage of non-disulphide-linked IgG_1_ aggregates. The dithiothreitol (646,563, MilliporeSigma, USA) was used to reduce the disulphide bonds of the IgG_1_. IgG_1_ purification was performed using NAb Protein A Plus Spin column (89,952, Thermo Fischer Scientific, USA).

Before Day 4, viable cell concentration basically followed an exponential increase. Therefore, the constant doubling time was assumed and cell growth rate before Day 3 was determined according to Eq. (5)

4 $$ Growth\,rate_{day\,i} = \frac{{ln\left( {\left[ {viable\,cell} \right]_{day\,i} /\left[ {viable\,cell} \right]_{day\,1} } \right)}}{time} $$

5 $$ Doubling\,time = \frac{ln2}{{Growth\,rate}} $$

Unpaired two-tail t-test without assuming equal variances was used to compare two sample data. When the *p* value is smaller than 0.05, we believed that there was a statistically significant difference between the two samples. Vice versa. *p* values of important comparison were shown in Suppl. Table S1 and Suppl. Table S2.

## Results

### Reduced initial proliferation rate and increased maximum cell concentration under hydrostatic pressure

**Figure 2:**
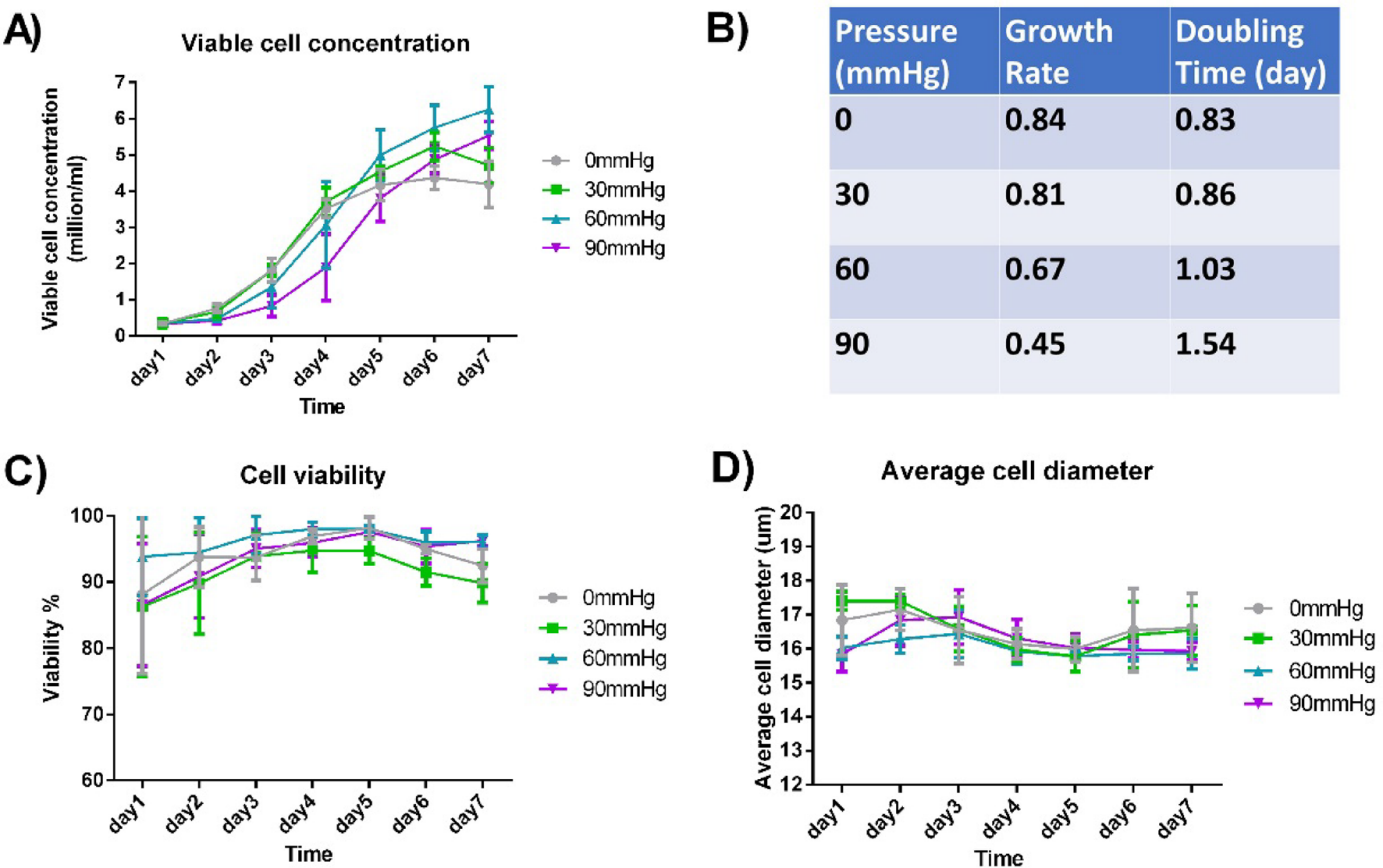
Cell growth under different pressure conditions. (**A**) Viable cell concentration in the culture system for up to 7 days. (**B**) CHO cells’ growth rate and doubling time under different pressure conditions on Day 3. (**C**) Cell viability across the 7-day culture. (**D**) Change of average live-cell diameter across the 7-day culture. (*n* = 3).

With the same initial viable seeding concentration of 0.35 million cells/ml, CHO-DG44 was cultured in our pressure system for 7 days. Four different pressure conditions (0, 30, 60, and 90 mmHg) were examined. Compared with 0 mmHg group, initial cell proliferation was significantly slowed down in 60 mmHg and 90 mmHg groups (Fig. 2A). On Day 3, the cell growth rate under 60 and 90 mmHg pressure was lower than 0 mmHg group by 20% and 46% respectively. However, pressurised cultures generally achieved higher maximum cell concentrations in the spinner flask on a later day (day 5–7, Fig. 2A). On Day 7, average cell concentration under 30, 60 and 90 mmHg was higher than 0 mmHg by 12%, 50% and 32% respectively. Statistically, a significant difference existed in the comparisons above (Suppl. Table S1).

Although cell viability was the lowest on Day 1 and Day 7 regardless of pressure condition, most cells were in good conditions as overall viability never dropped below 80% (Fig. 2B). No significant difference was observed in cell viability between different pressure conditions (Fig. 2C, Suppl. Table S1). As shown in Fig. 2D, the average live-cell diameter was mostly in a range of 15–18 μm, and no significant difference was found between different pressure conditions (Suppl. Table S1).

To confirm that the difference we observed was due to the pressure difference instead of dissolved gas concentration change, we also measured the partial pressure of oxygen (pO_2_), carbon dioxide (pCO_2_) and pH of the CHO cell culture using the cell culture analyser (Suppl. Fig. S1A–C). Comparing with the reported values that caused significant influence on CHO cell culture^28–30^, the variation of pO_2_, pCO_2_ and pH under different pressure conditions was negligible.

### Metabolite analysis under hydrostatic pressure

**Figure 3:**
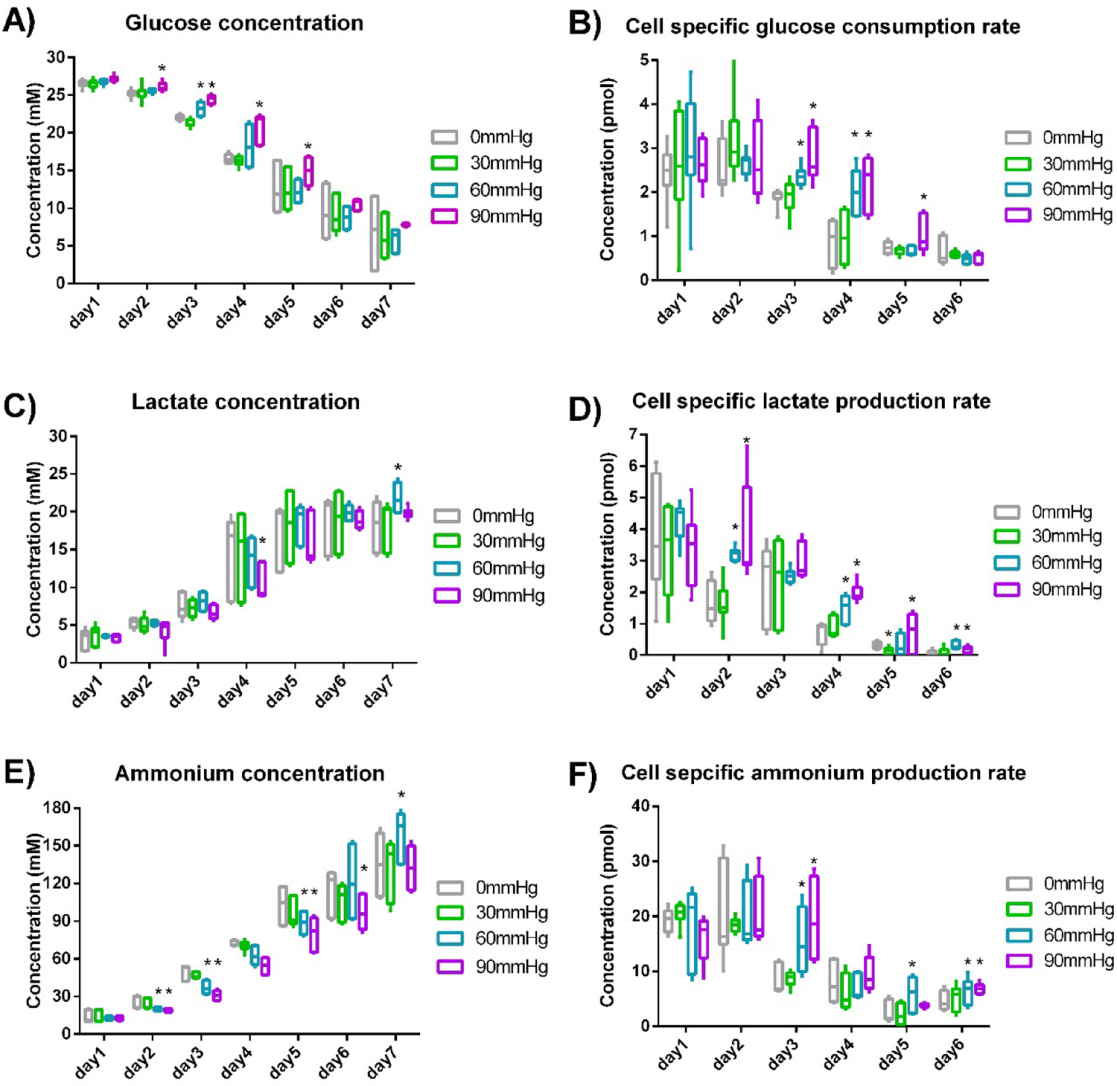
Metabolism of CHO cells. (**A**) Glucose concentration change. (**B**) Cell-specific glucose consumption rate. (**C**) Lactate concentration change. (**D**) Cell-specific lactate production rate. (**E**) Ammonium concentration change. (**F**) Cell-specific ammonium production rate. (*n* = 3). *Indicates a statistically significant difference (*p* < 0.05) when comparing with 0 mmHg condition.

The concentration of glucose, lactate and ammonium in the culture medium was analyzed daily using the automated cell culture analyser. The overall glucose, lactate and ammonium concentrations were shown in Fig. 3A,C,E. No significant difference in overall metabolite concentration was found between 0 and 30 mmHg pressure conditions (Suppl. Table S2). From Day 2 to Day 5, glucose concentration under 60 and 90 mmHg pressure was generally higher than no pressure condition (Fig. 3A, Suppl. Table S2). Comparing with no pressure condition, ammonium concentration from Day 2 to Day 5 under 60 mmHg and 90 mmHg pressure was lower by 16–32% (Fig. 3E, Suppl. Table S2). Even though viable cell concentration under pressure on Day 6 and Day 7 was significantly higher than that without pressure by more than 30% (Fig. 2A), less metabolites’ concentration difference was observed on Day 6 and Day 7 (Fig. 3, Suppl. Table S2) because of the reduced metabolic rate when there was insufficient glucose in the medium. This was shown by metabolites’ concentration change per cell per day (Fig. 3B,D,F). As glucose concentration decreased, cell-specific glucose consumption rate also dropped down. As lactate and ammonium concentration increased, lactate and cell-specific ammonium production rate decreased. By comparing cell proliferation and lactate/glucose ratio on Day 3, Day 5 and Day 7 from this study and literature (Fig. 2A, Suppl. Fig. S1D,E), CHO cells were in exponential growth and stationary phase in this study. The trend of metabolite concentration change basically followed the general trends of CHO cell growth in batch culture^31–34^. Besides, no significant difference was observed in lactate/glucose ratio between different pressure conditions (Suppl. Fig. S1D).

No significant difference in metabolite concentration change was observed between different pressure conditions on Day 1, Day 5 and Day 6. On Day 3 and Day 4, cell-specific glucose consumption rate under 60 and 90 mmHg was statistically higher than that under 0 mmHg by 25–155% (Fig. 3B, Suppl. Table S2). When compared with 0 mmHg condition, cell-specific lactate production rate under 60 and 90 mmHg on Day 2 and Day 4 was higher by 82–177% (Fig. 3D, Suppl. Table S2). On Day 3, cell-specific ammonium production rate under 60 and 90 mmHg was higher than no pressure condition by 76% and 127%, respectively (Fig. 3F, Suppl. Table S2). Metabolite change under 30 mmHg was not different from 0 mmHg in a statistically significant way (Fig. 3, Suppl. Table S2).

### IgG production and aggregate formation under hydrostatic pressure

**Figure 4:**
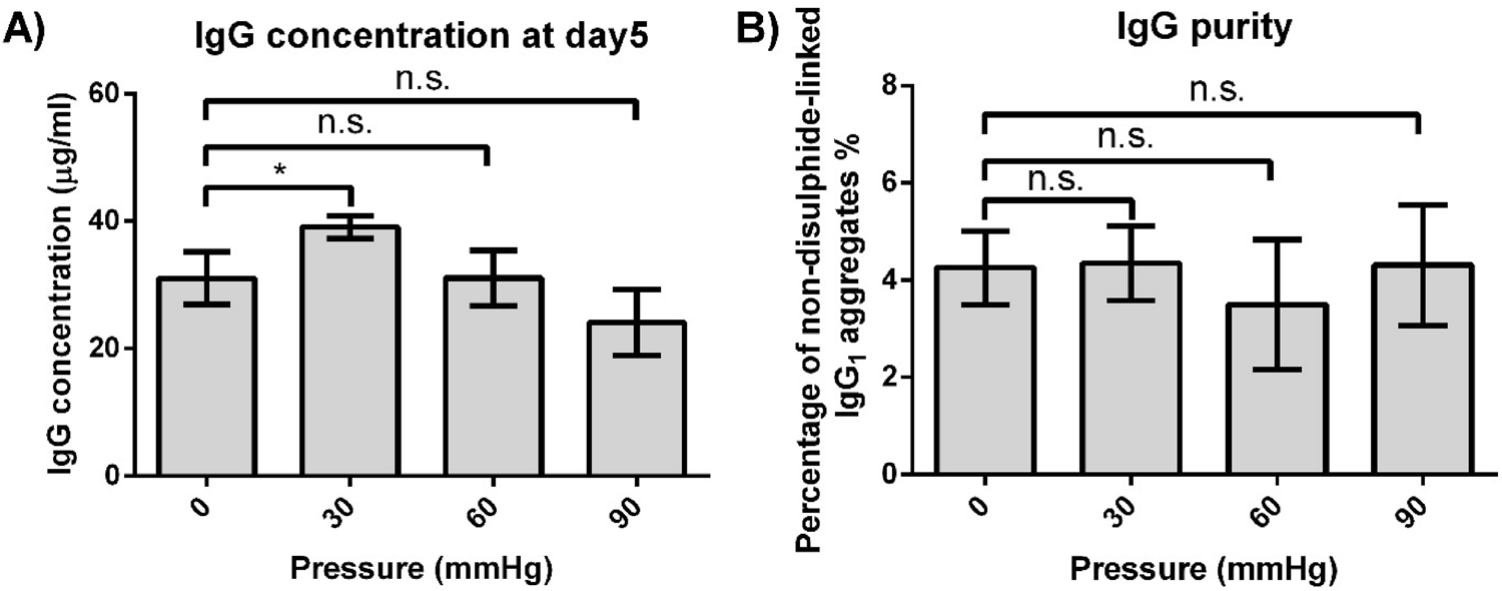
Antibody concentration and non-disulphide-linked IgG_1_ aggregates under different pressure conditions. (**A**) Overall IgG_1_ concentration measured on Day 5. (**B**) The proportion of IgG_1_ aggregates under reducing condition on Day 5. (*n* = 3) *Denotes statistical significance (*p* < 0.05) while n.s. denotes no statistically significant difference (*p* > 0.05).

On Day 3, Day 5 and Day 7, overall IgG_1_ concentration from pressure culture was measured using HPLC with a protein A column (Suppl. Fig. S2). IgG_1_ concentration under 30 mmHg on Day 5 was higher than 0 mmHg group by 26% (Fig. 4A). When compared with 0 mmHg condition, no statistically significant difference was observed in 60 mmHg and 90 mmHg conditions. However, the average IgG_1_ concentration under 90 mmHg was lower than 0 mmHg group by 22%.

Integral of viable cells (IVC) is introduced to compare IgG_1_ productivity of a single CHO cell. IVC in a day is the summation of viable cell concentration before that day. Normalized IgG_1_ concentration is obtained by dividing overall IgG_1_ concentration with IVC, indicating the antibody productivity per cell. Compared with 0 mmHg condition, antibody productivity per cell under 30 mmHg, 60 mmHg, and 90 mmHg increased by 21%, 10% and 19% respectively (Table 1). In addition, there was a 4.4% increase in IVC under 30 mmHg. As a result, overall IgG_1_ concentration under 30 mmHg was higher than that under 0 mmHg by 26%.

**Table 1:** Integral of viable cells and normalized IgG_1_ concentration at Day 5 (Value in the table is shown as mean ± SEM).

Pressure (mmHg)	Integral of viable cells (day*million/ml)	Percentage increase compared with 0 mmHg condition (%)	Normalized IgG_1_ concentration (µg/ml)	Percentage increase compared with 0 mmHg condition (%)
0	10.6 ± 0.2	–	2.9 ± 0.2	–
30	11.1 ± 0.1	4.4, n.s	3.5 ± 0.1	21*
60	10.2 ± 0.8	−3.5, n.s	3.2 ± 0.6	10, n.s
90	7.3 ± 0.6	−31, n.s	3.5 ± 0.6	19, n.s

Comparing 30, 60 and 90 mmHg pressure conditions with 0 mmHg, the result of their two-tailed T-test was included. n.s. is *p* > 0.05. * is *p* < 0.05.

By performing IgG_1_ purification and on-chip electrophoresis, non-disulphide-linked IgG_1_ aggregates were detected. The proportion of these aggregates was calculated based on the area under peaks (Suppl. Fig. S3). No significant difference was observed between different pressure conditions (Fig. 4B). This indicates that a hydrostatic pressure up to 90 mmHg does not affect IgG_1_ non-disulphide-linked IgG_1_ aggregates.

### Cell cluster formation

**Figure 5:**
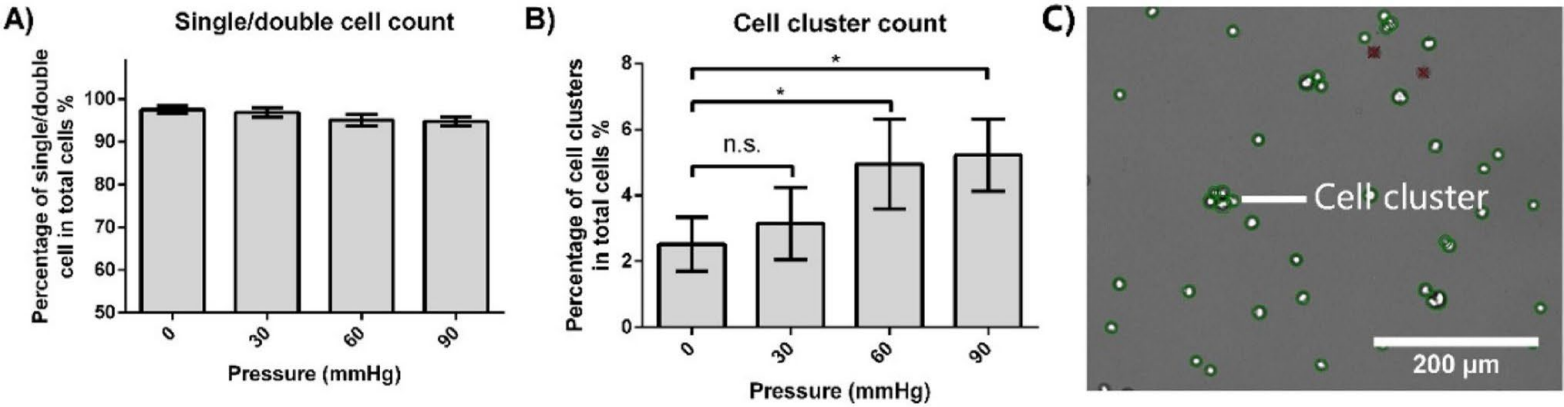
Percentage of cell cluster formation on Day 5. (**A**) The proportion of single and double cells under different pressure conditions. (**B**) Proportion of cell clusters under different pressure conditions. (**C**) Representative image from the cell culture analyzer. Green circles indicate viable cells, and red crosses indicate dead cells based on Trypan blue exclusion. (*n* = 3) *Denotes statistical significance (*p* < 0.05). “n.s.” denotes no statistically significant difference (*p* > 0.05).

Cell aggregate formation is an undesired phenomenon in CHO cell culture as it inhibits nutrient transport and reduces cell viability and overall productivity^35^. Using images taken by the automated cell analyser, we quantified the percentage of cell clumping under different pressure conditions on Day 5 (Fig. 5). The cell clusters having more than two cells in a single aggregate were counted (Fig. 5C).

Single and double cells were more than 90% in all different pressure culture groups (Fig. 5A). When compared with 0 mmHg condition, the proportion of CHO cell clusters under 30 mmHg showed no significant difference (Fig. 5B). However, cell clumping under 60 and 90 mmHg pressure conditions were approximately twice that in 0 mmHg group.

## Discussion

In this work, we have demonstrated that both cell concentration and antibody production from CHO cell culture can be significantly enhanced by the application of moderate hydrostatic pressure. If further validated in a larger scale culture with production-quality CHO cell lines, this result will have immediate utility and impact in the current biomanufacturing workflow.

In industrial-scale CHO cell culture, an overpressure of several hundred mbar is often used to prevent microbial contamination and the height of the liquid in such bioreactors could be 1–3 m which result in a 100–300 mbar (75–225 mmHg) hydrostatic pressure^36^. Although the optimal pressure for antibody production in our study (40–80 mbar, or 30–60 mmHg) cannot be directly applied to these industrial bioreactors, our study suggests the existence of an optimal hydrostatic pressure range in the large bioreactors. Depending on the optimal pressure, modification of bioreactor design or usage may be required. For example, if a pressure range of 100–200 mbar (75–150 mmHg) could increase the overall antibody production by 30% without comprising product quality, the liquid in current bioreactors should be limited to one meter in height and the gas pressure in bioreactors should be 100 mbar. Alternatively, new horizontally larger and shorter bioreactors may be used if the optimal pressure range is lower.

Even if it is challenging to find an optimal pressure range that can increase the productivity in the large-scale bioreactor, our work would be still meaningful because it is reveals another important yet under-studied factor in the bioreactor. Most process development in the industry is now performed in small scale bioreactors with minimal or no pressure applied, while the actual large-scale biologics production occurs in a quite different pressure environment. Our results suggest that we may need to vary the pressure conditions in process development to identify truly optimal conditions for large scale CHO cell-based biologics production.

The detailed molecular-level mechanism behind our observation is yet unclear, just as many cell phenotypic changes resulting from biophysical cues. We have demonstrated that the initial proliferation rate of CHO cells was slowed down (while the maximum cell concentration increased) under a hydrostatic pressure of 30–90 mmHg (Fig. 2A, 2B). Besides, we also observed some increase in cell-specific glucose consumption, lactate and ammonium production rate under 60 mmHg and 90 mmHg from Day 2 to Day 4 (Fig. 3B, 3D, 3F and Suppl. Table S2). A potential explanation is that hydrostatic pressure may have selected out a subpopulation of the CHO cells that have a higher metabolic rate and proliferate faster. This selection may be achieved via an increase in necrosis factor expression or a mechano-transductive mechanism^18,37^. Because of the selection process, the subpopulation of cells not favoured for expansion may have stopped proliferation, and a decrease in the initial proliferation rate could occur. More molecular biology experiments and long-term pressurized CHO cell culture will be required to validate this speculation in the future.

The increased cell concentration under pressure after one week of culture was also observed in many other cell types previously, such as MSCs^19,38^, bovine cartilage^39^ and many different cancer cell lines^18,40^. Also, the optimal pressure (30 mmHg) in this work is well in line with the typical interstitial pressure found in human or animal tissue/organs, therefore suggesting that this effect is indeed related to the inherent in vivo cellular environment. This increase in cell proliferation may be caused by facilitating cell cycle initiation^38^ and/or activation of the tyrosine kinase pathway^40^. Hydrostatic pressure can promote cell cycle initiation by enhancing activities of cytoskeletal regulatory proteins Ras homolog gene family member A (RhoA) and Ras-related C3 botulinum toxin substrate 1 (Rac1)^38^. Both pathways eventually lead to increased cyclin-dependent kinase (CDK) activity which positively regulates G1 phase progression of the cell^41,42^.

The influence of hydrostatic pressure on overall IgG_1_ production was also observed in this study (Fig. 4A). The increased overall IgG_1_ concentration (26%) under 30 mmHg resulted from a slight increase (4.4%) in IVC and enhanced antibody productivity (21%) per cell under hydrostatic pressure (Table 1). The increase in normalized IgG_1_ concentration under pressure may suggest that the pressure-favoured subpopulation of CHO cells may have higher IgG_1_ productivity. Cellular metabolism and proliferation change may indicate the happening of this kind of cell subpopulation selection. For example, chondrogenic MSC subpopulation showed significantly reduced oxygen consumption after 24hrs pellet culture whereas the MSCs in osteogenic culture did not manifest this change^43^. The decrease in IgG_1_ level under 90 mmHg, when compared with 0 mmHg condition (Fig. 4A), was probably due to its lower IVC and a significant increase in cell cluster formation. As shown in Table 1, the IVC of 90 mmHg was lower than 0 mmHg condition by 31%. In addition, 90 mmHg hydrostatic pressure also greatly promoted cell cluster formation (Fig. 5B) which may affect cell behaviour and hinder antibody production^35,44^. Although normalized IgG_1_ concentration was higher under the 90 mmHg condition when compared to 0 mmHg group, its lower IVC and severe cell clumping still resulted in an overall decrease in IgG_1_ production.

The influence of hydrostatic pressure on CHO cells’ proliferation and antibody productivity makes it an attractive lever for improving the titer of antibody products. In this study, 30 mmHg pressure was shown to be the optimum pressure for antibody production. Yet, we recognize that modern production-capable CHO cells are highly engineered and optimized, which suggests that the optimal pressure conditions for better production rates may be different, or in some cases, the strategy presented here may not be generally applicable to all CHO cells used in the industry. At the same time, antibody production using CHO cells is increasingly done via long-term perfusion cultures, with potentially different processing environments. However, the reduced IVC under higher pressure conditions may be overcome through long-term (perfusion) culture because the selection process is likely to disappear after several passages under the same pressure environment. Increased cell clumping (observed when pressure is higher than 60 mmHg in this work) is also possible to be resolved by implementing methods such as using a commercial anti-clumping agent or special culture medium^45^ to avoid cell cluster formation. By overcoming the issues of reduced IVC and increased cluster formation, higher pressure conditions could potentially increase overall IgG concentration even further.

## Conclusion

In this work, we found that pressurized culture may favour a subpopulation of CHO cells with a higher metabolic and proliferation rate. We have also demonstrated that pressurized culture could increase the maximum CHO cell concentration during one-week batch culture, although cell cluster formation significantly increased at > 60 mmHg. It was also shown that applying hydrostatic pressure of 30 mmHg to CHO cells stably expressing monoclonal antibodies can increase harvest titers by up to 26% without compromising antibody aggregate formation. The results suggest that creating an optimum pressure environment is a straightforward and attractive method for rapidly improving the overall yield of biopharmaceutical products.

## Supplementary Information

Supplementary Information 1.

Supplementary Information 2.

## Data Availability

All data used to generate the figures and tables presented in this manuscript can be found in the S1 file in the supporting information. There are no restrictions on the availability of these data.
