# Supplementary figures and images for: Investigating the influence of physiologically relevant hydrostatic pressure on CHO cell batch culture (part 2 of 2)
Source: Sci Rep. 2021 Jan 8;11:162. doi: 10.1038/s41598-020-80576-8 (PMC7794228; doi:10.1038/s41598-020-80576-8)

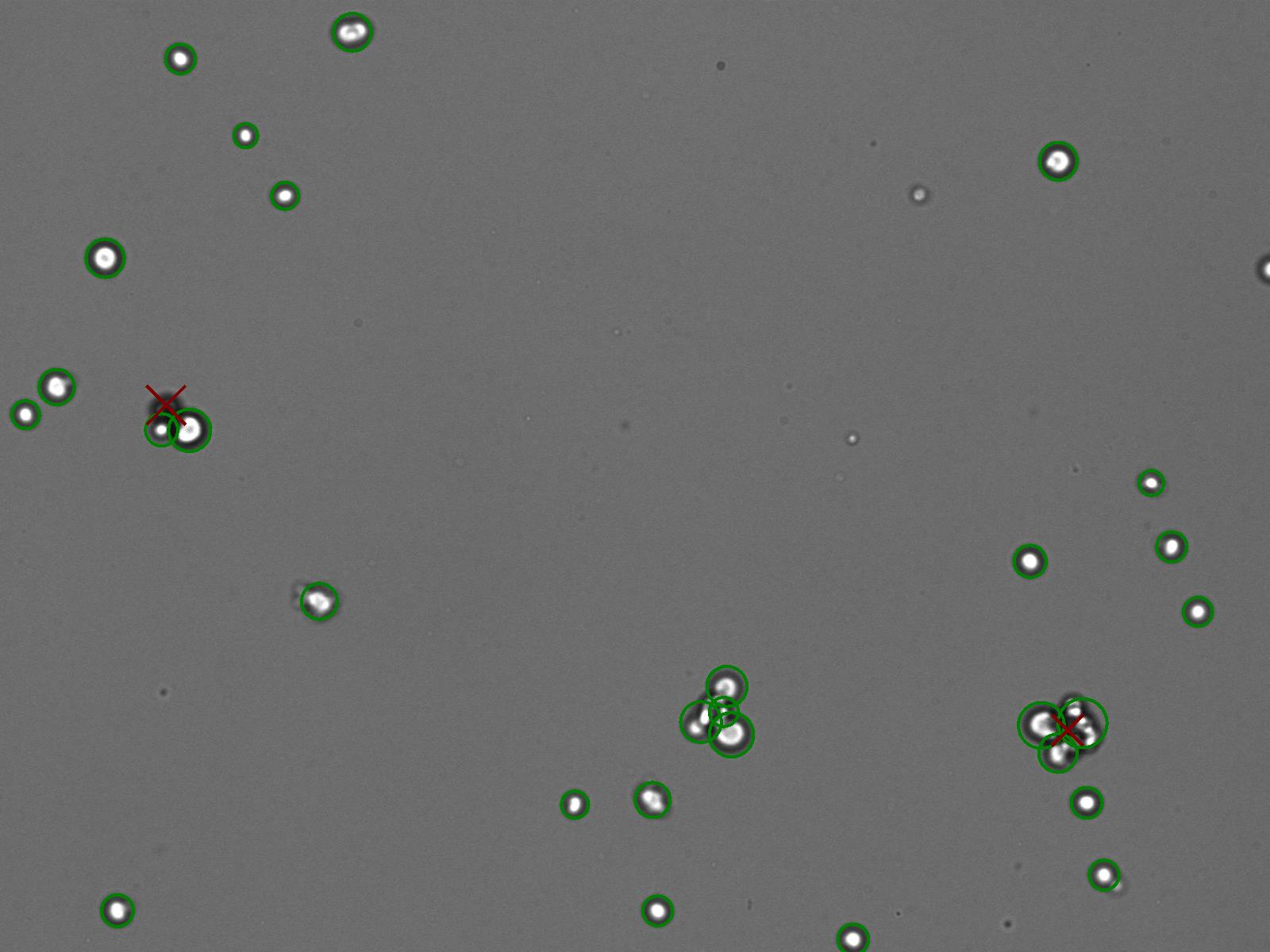

Supplement: Supplementary file 1 — Supplementary Information 1. [file 41598_2020_80576_MOESM1_ESM.zip › S1/Aggregate counts/day5/0mmHg Jan10 41 39/ML SS1 3-050_2019-02-19_112529.bmp]

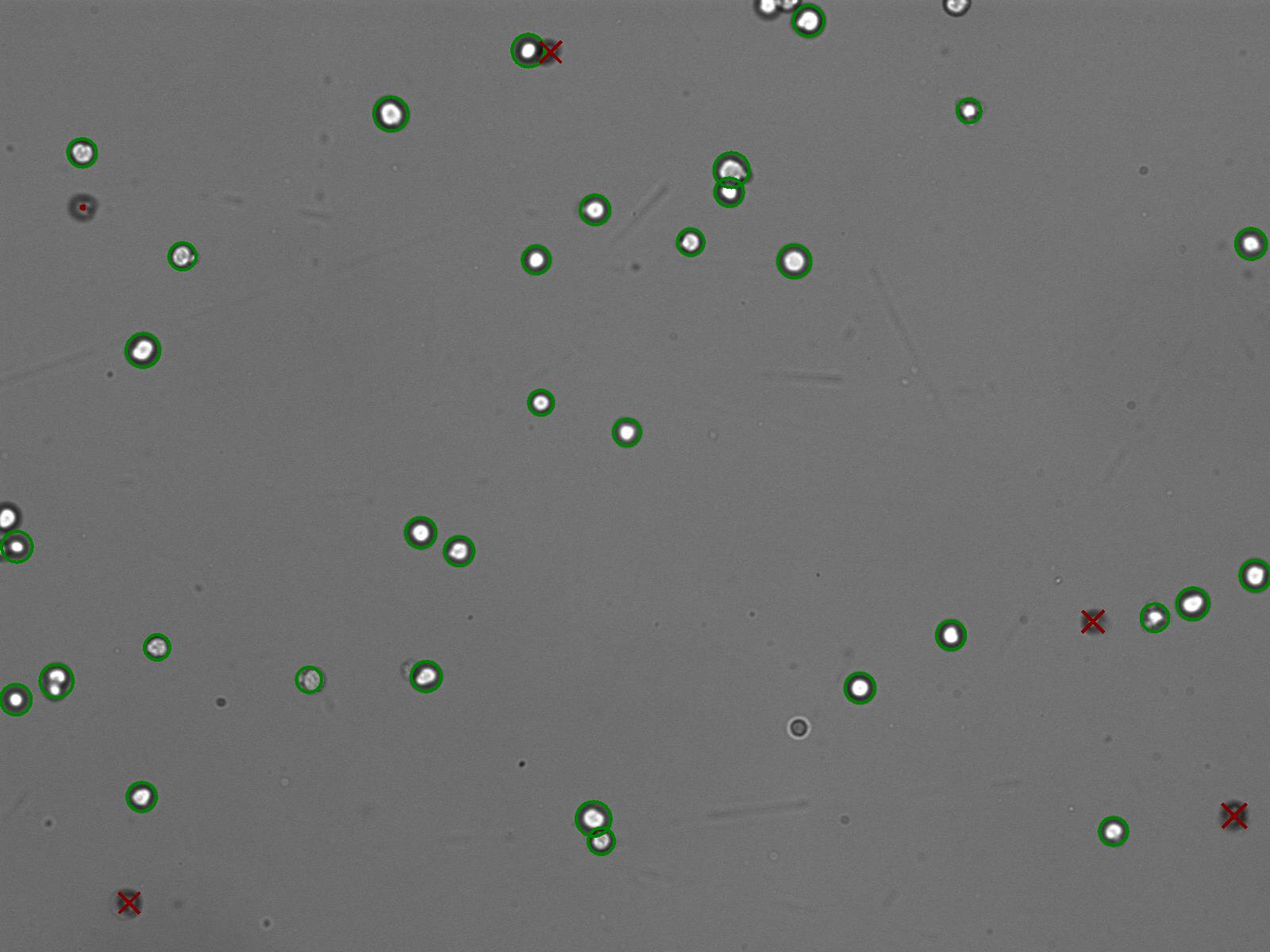

Supplement: Supplementary file 1 — Supplementary Information 1. [file 41598_2020_80576_MOESM1_ESM.zip › S1/Aggregate counts/day5/30mmHg Dec18 54 44/ML P2-001_2019-02-19_124633.bmp]

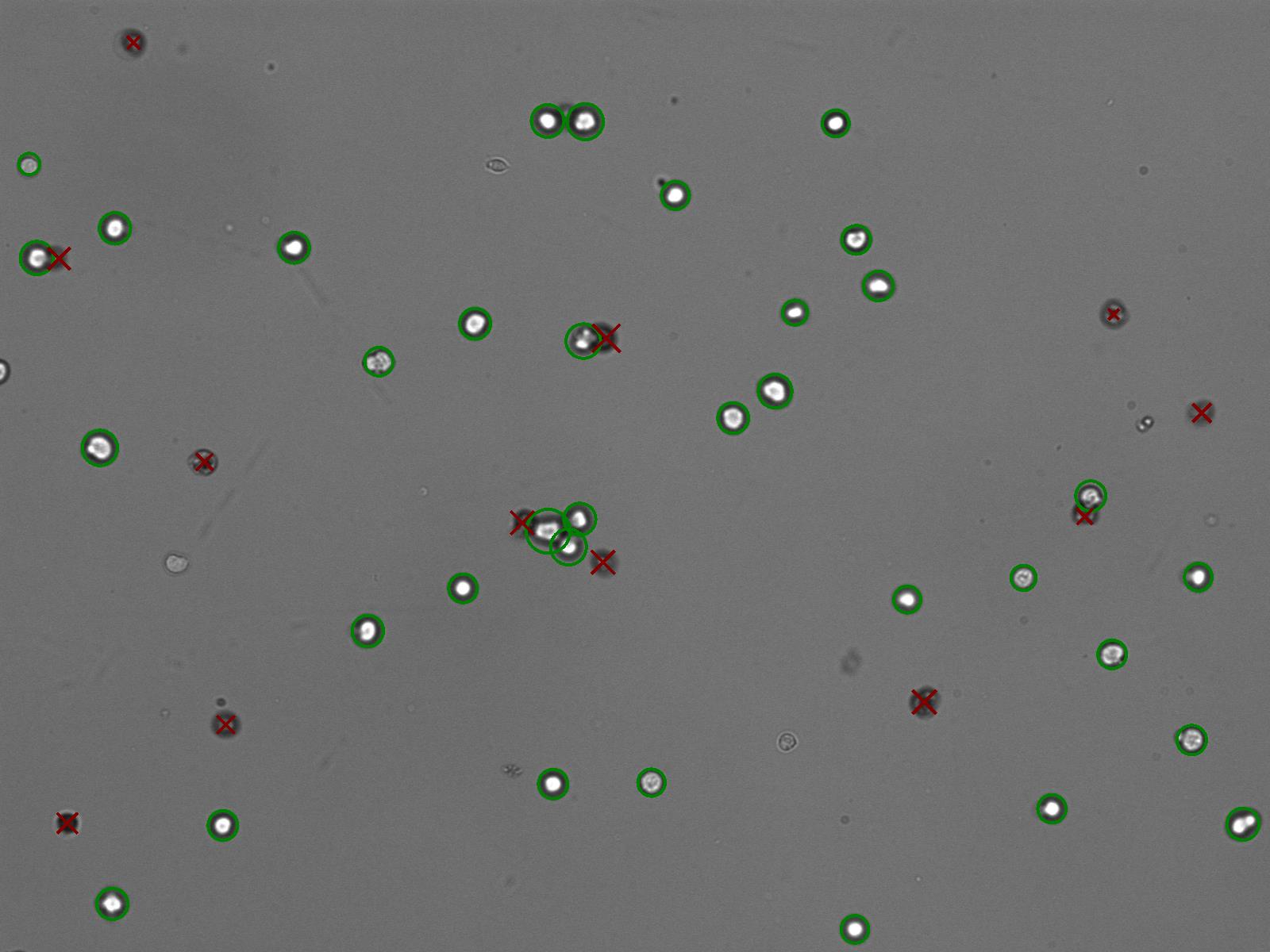

Supplement: Supplementary file 1 — Supplementary Information 1. [file 41598_2020_80576_MOESM1_ESM.zip › S1/Aggregate counts/day5/30mmHg Dec18 54 44/ML P2-002_2019-02-19_124634.bmp]

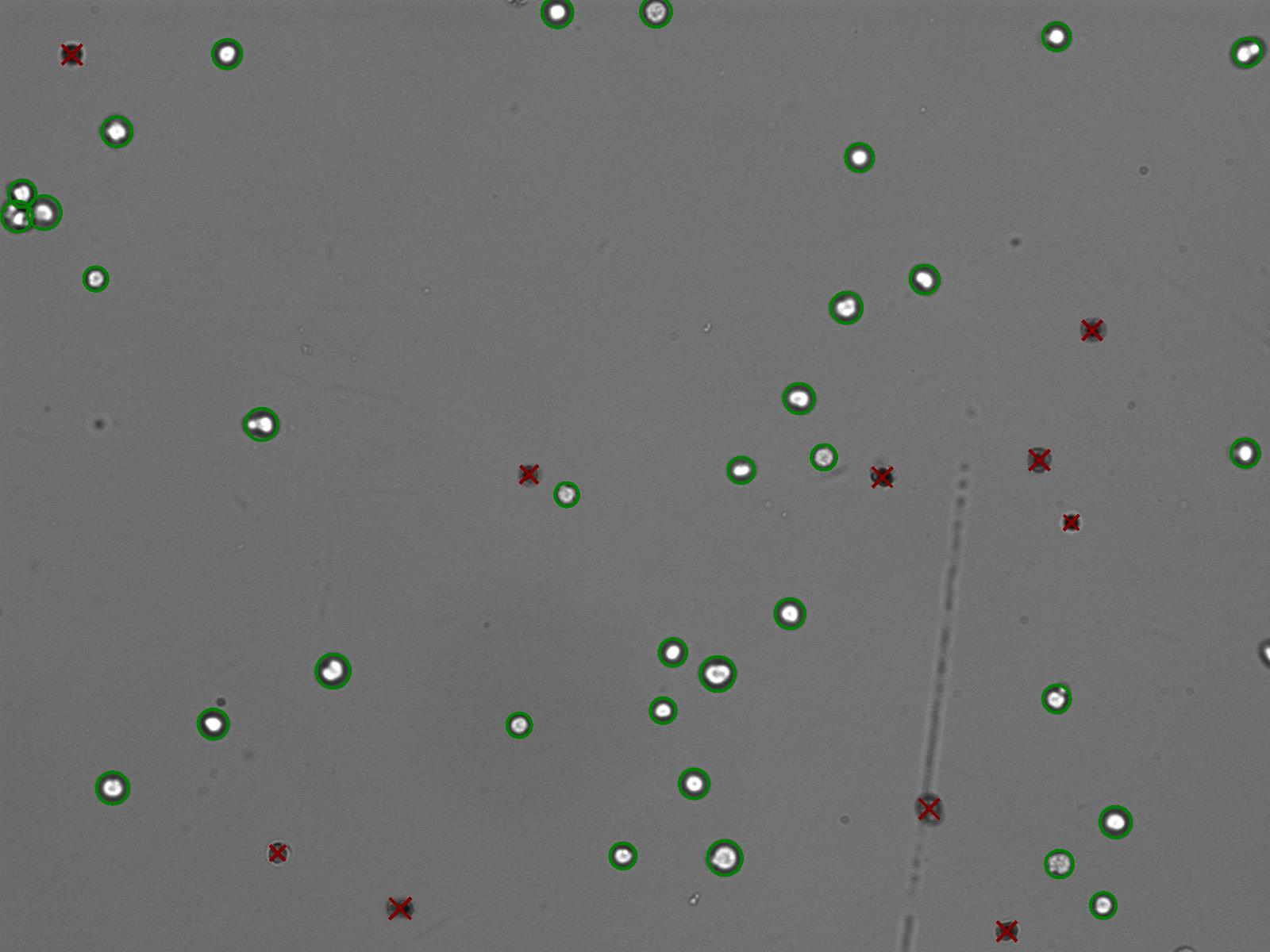

Supplement: Supplementary file 1 — Supplementary Information 1. [file 41598_2020_80576_MOESM1_ESM.zip › S1/Aggregate counts/day5/30mmHg Dec18 54 44/ML P2-003_2019-02-19_124634.bmp]

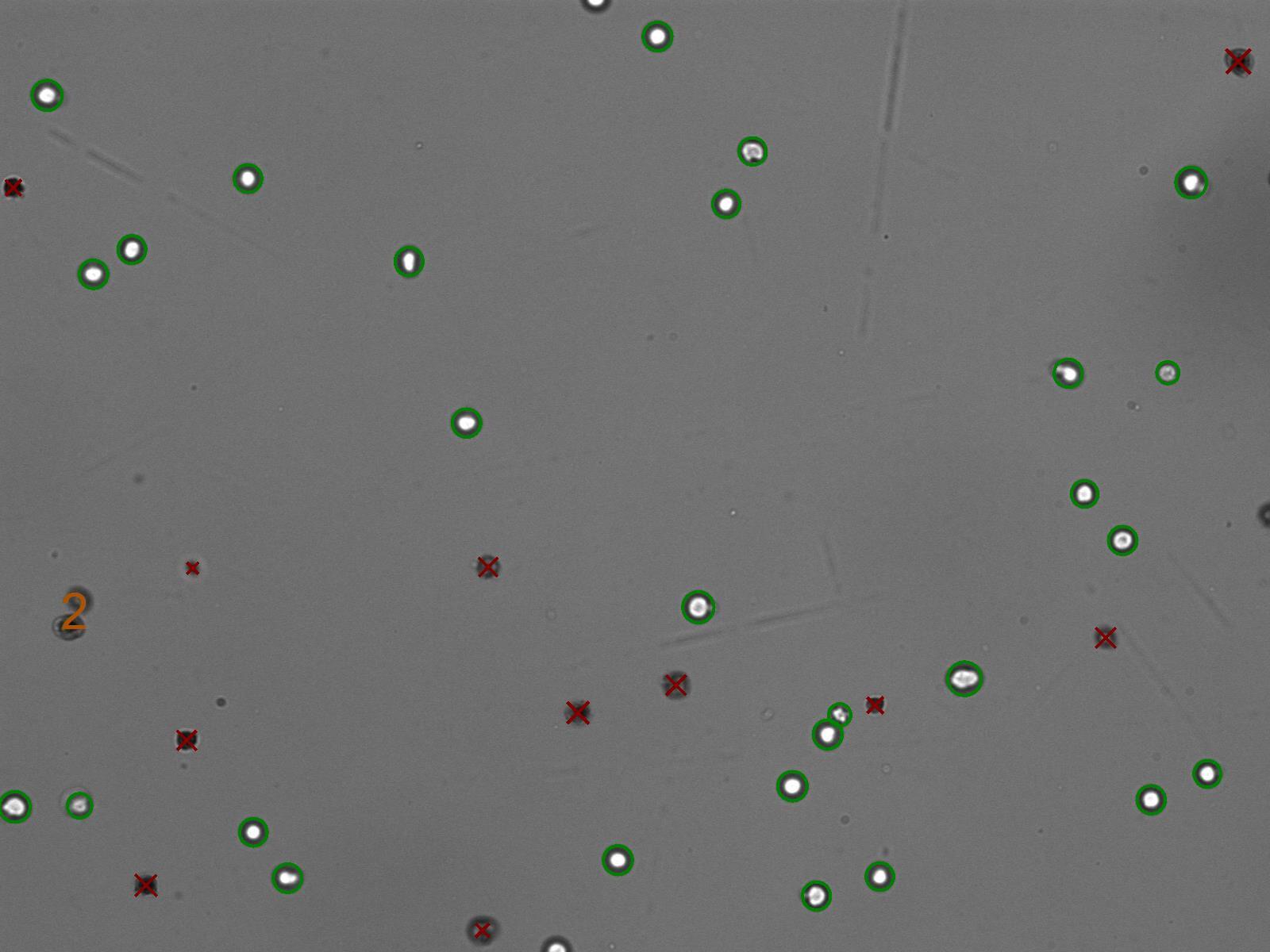

Supplement: Supplementary file 1 — Supplementary Information 1. [file 41598_2020_80576_MOESM1_ESM.zip › S1/Aggregate counts/day5/30mmHg Dec18 54 44/ML P2-004_2019-02-19_124634.bmp]

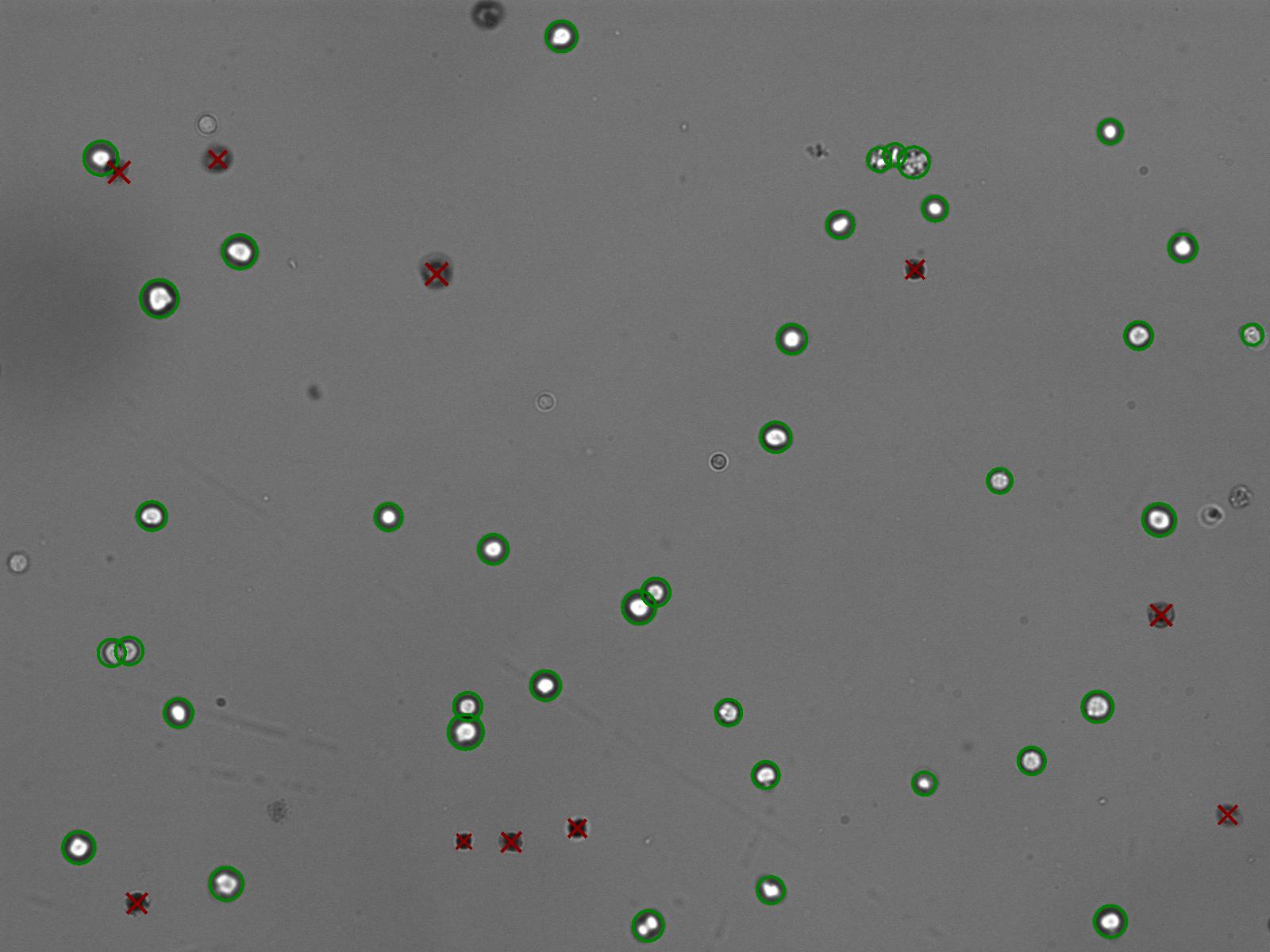

Supplement: Supplementary file 1 — Supplementary Information 1. [file 41598_2020_80576_MOESM1_ESM.zip › S1/Aggregate counts/day5/30mmHg Dec18 54 44/ML P2-005_2019-02-19_124635.bmp]

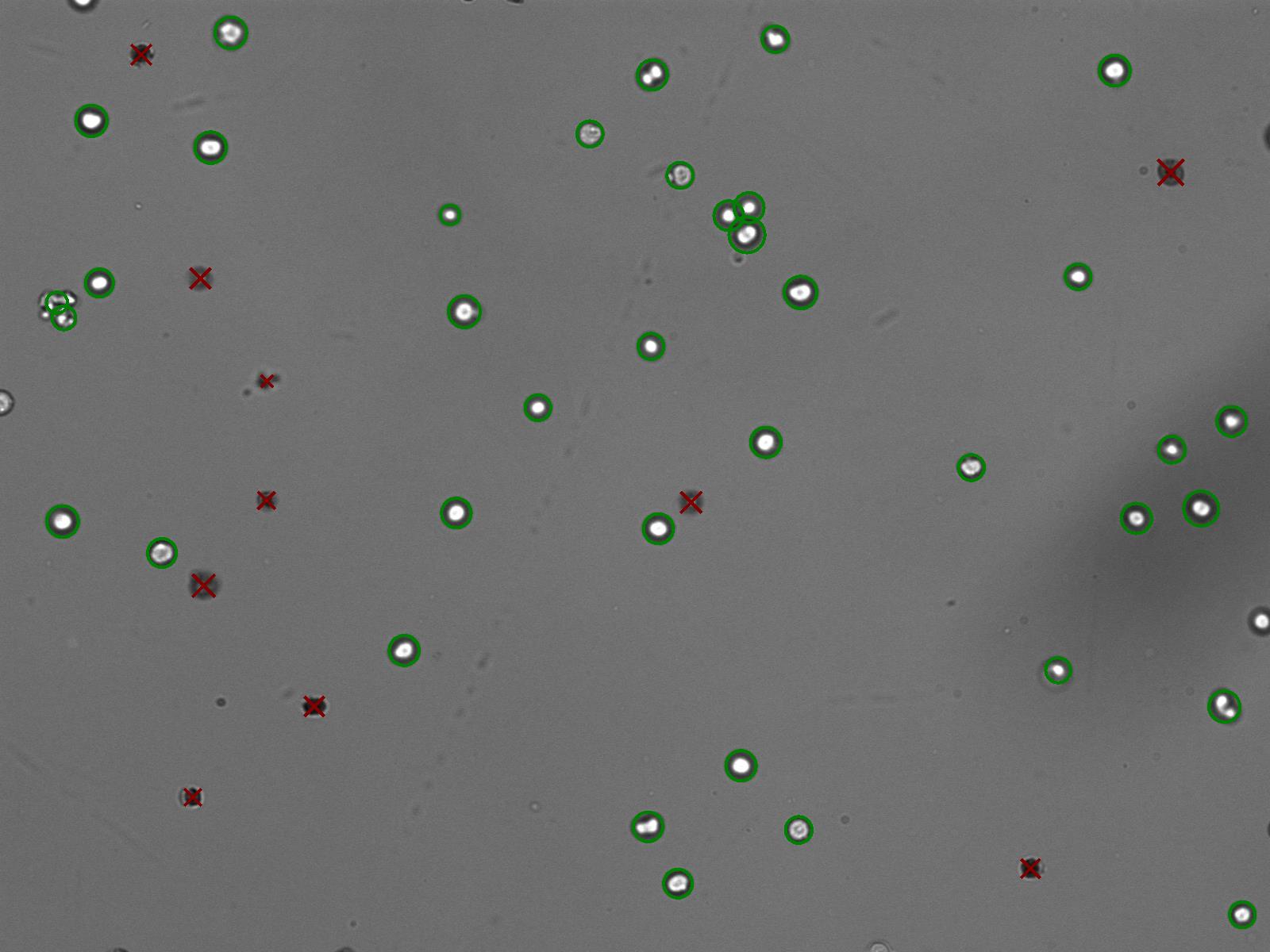

Supplement: Supplementary file 1 — Supplementary Information 1. [file 41598_2020_80576_MOESM1_ESM.zip › S1/Aggregate counts/day5/30mmHg Dec18 54 44/ML P2-006_2019-02-19_124635.bmp]

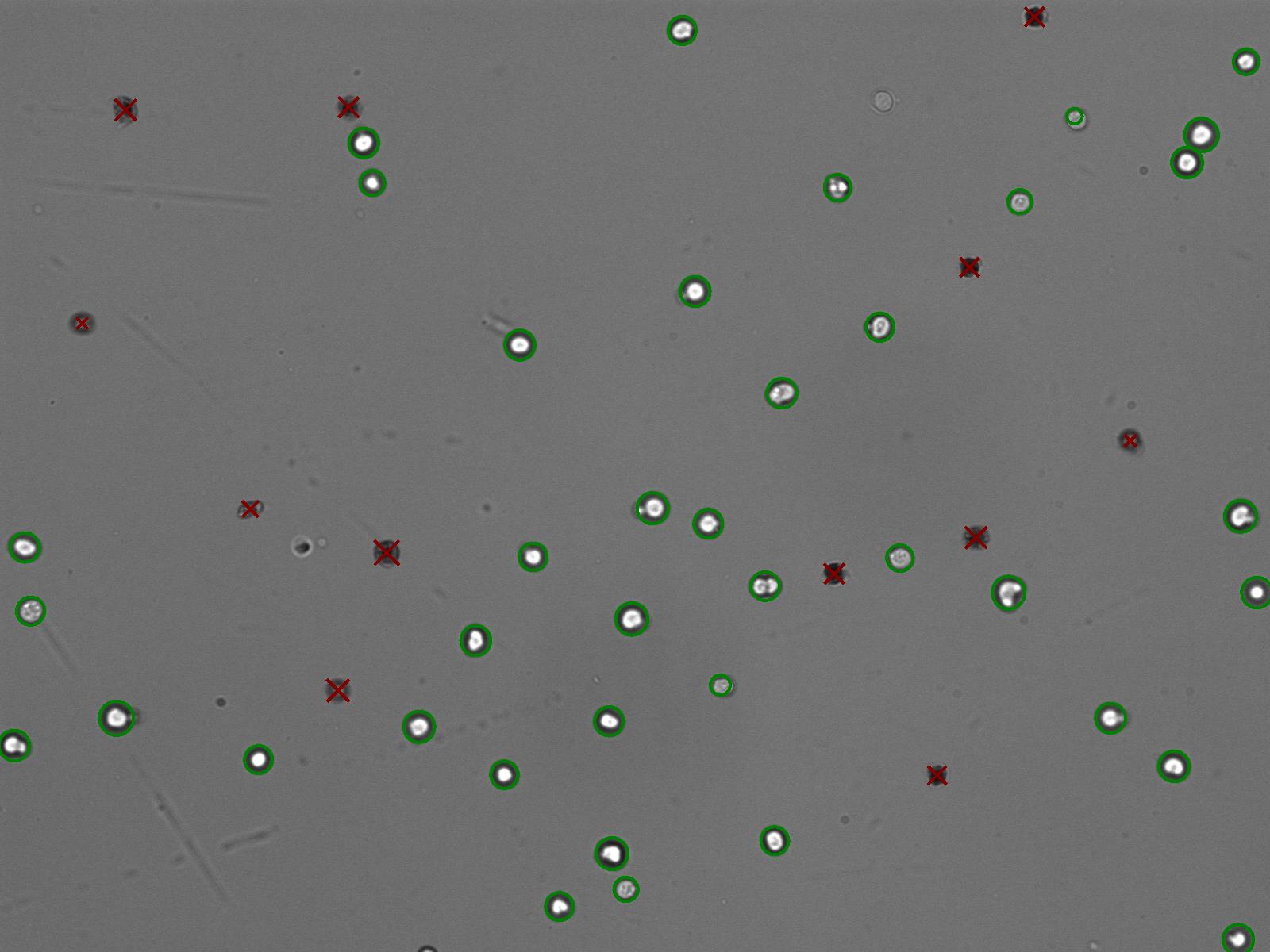

Supplement: Supplementary file 1 — Supplementary Information 1. [file 41598_2020_80576_MOESM1_ESM.zip › S1/Aggregate counts/day5/30mmHg Dec18 54 44/ML P2-007_2019-02-19_124635.bmp]

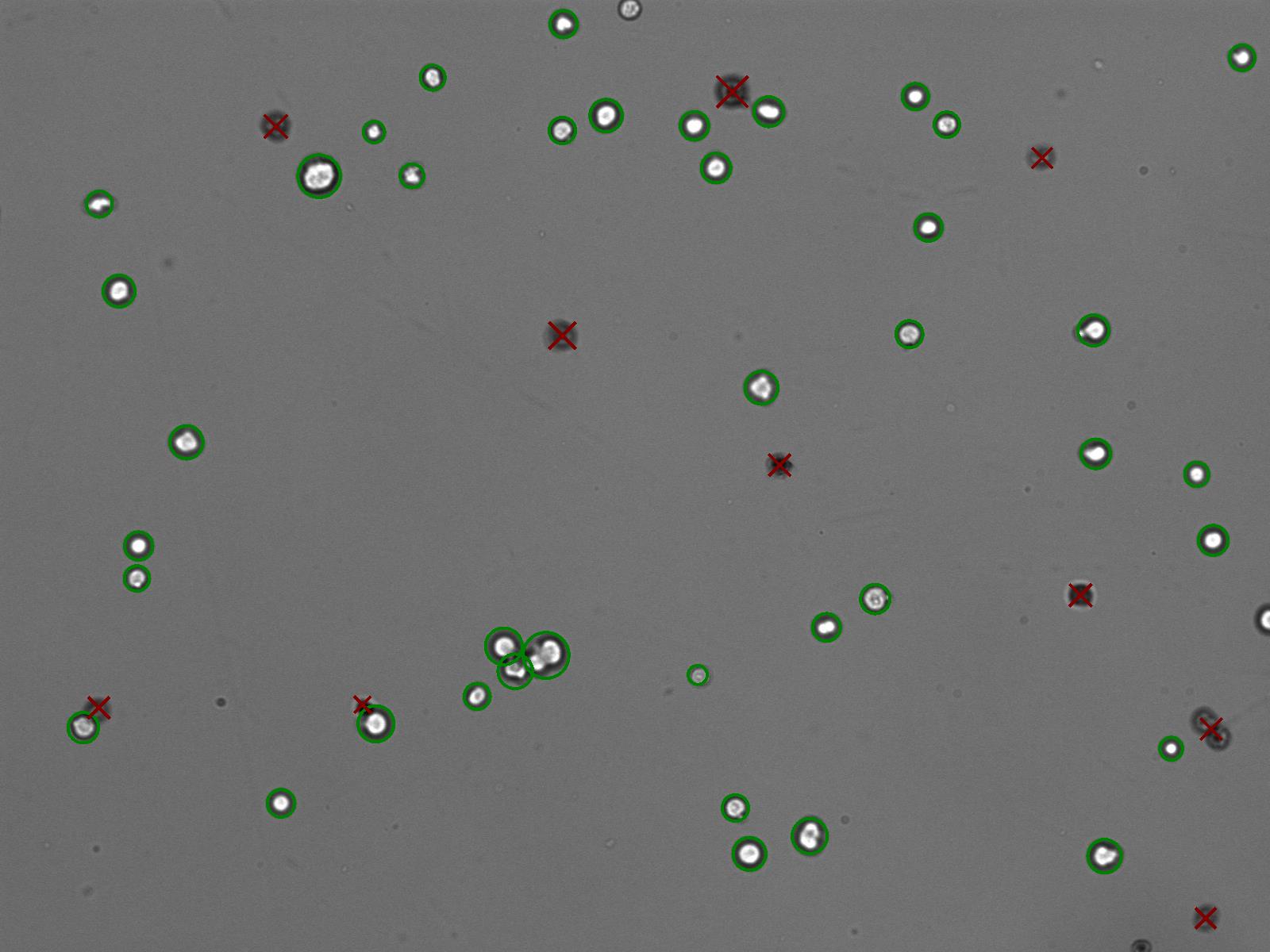

Supplement: Supplementary file 1 — Supplementary Information 1. [file 41598_2020_80576_MOESM1_ESM.zip › S1/Aggregate counts/day5/30mmHg Dec18 54 44/ML P2-008_2019-02-19_124636.bmp]

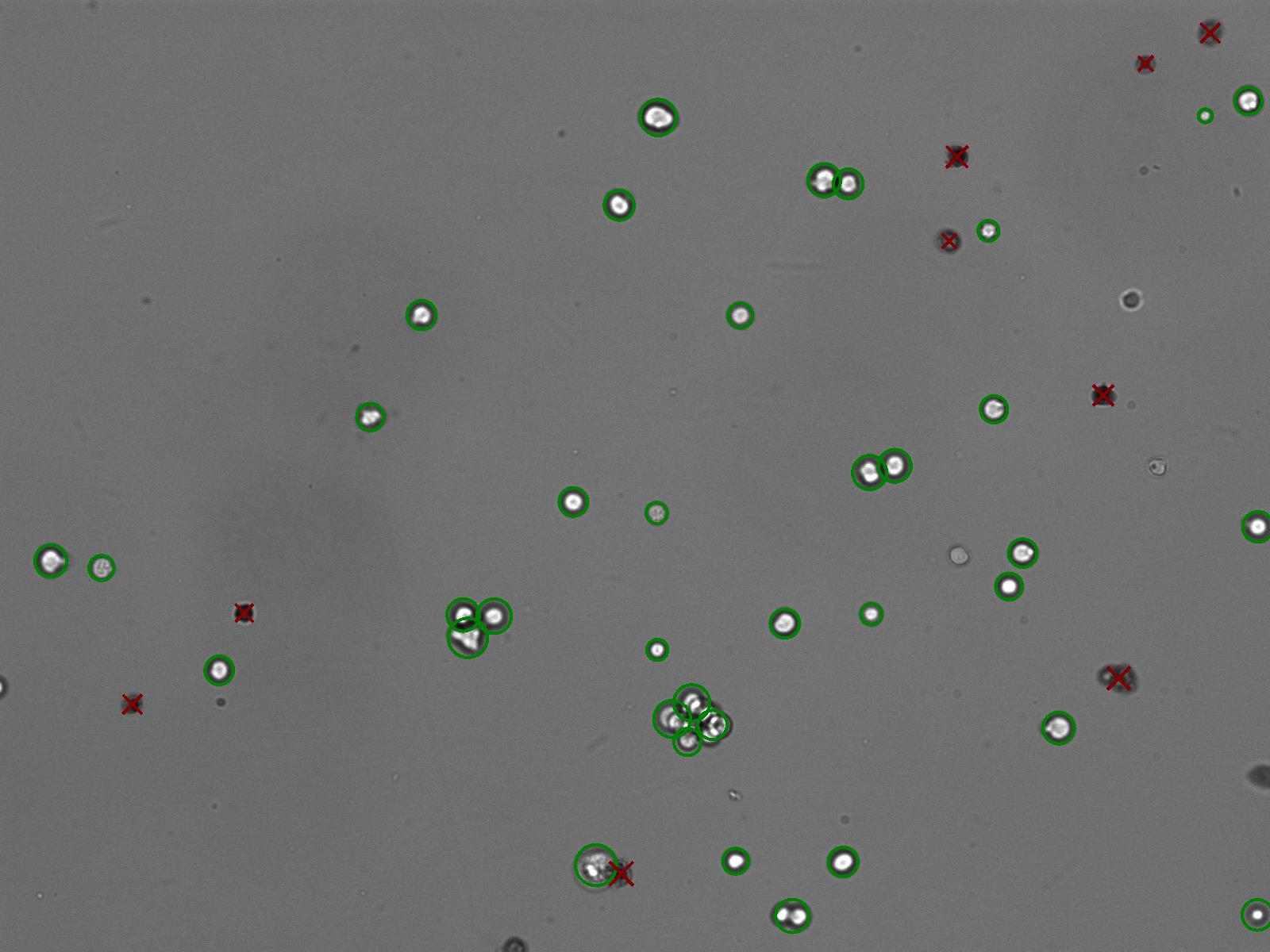

Supplement: Supplementary file 1 — Supplementary Information 1. [file 41598_2020_80576_MOESM1_ESM.zip › S1/Aggregate counts/day5/30mmHg Dec18 54 44/ML P2-009_2019-02-19_124636.bmp]

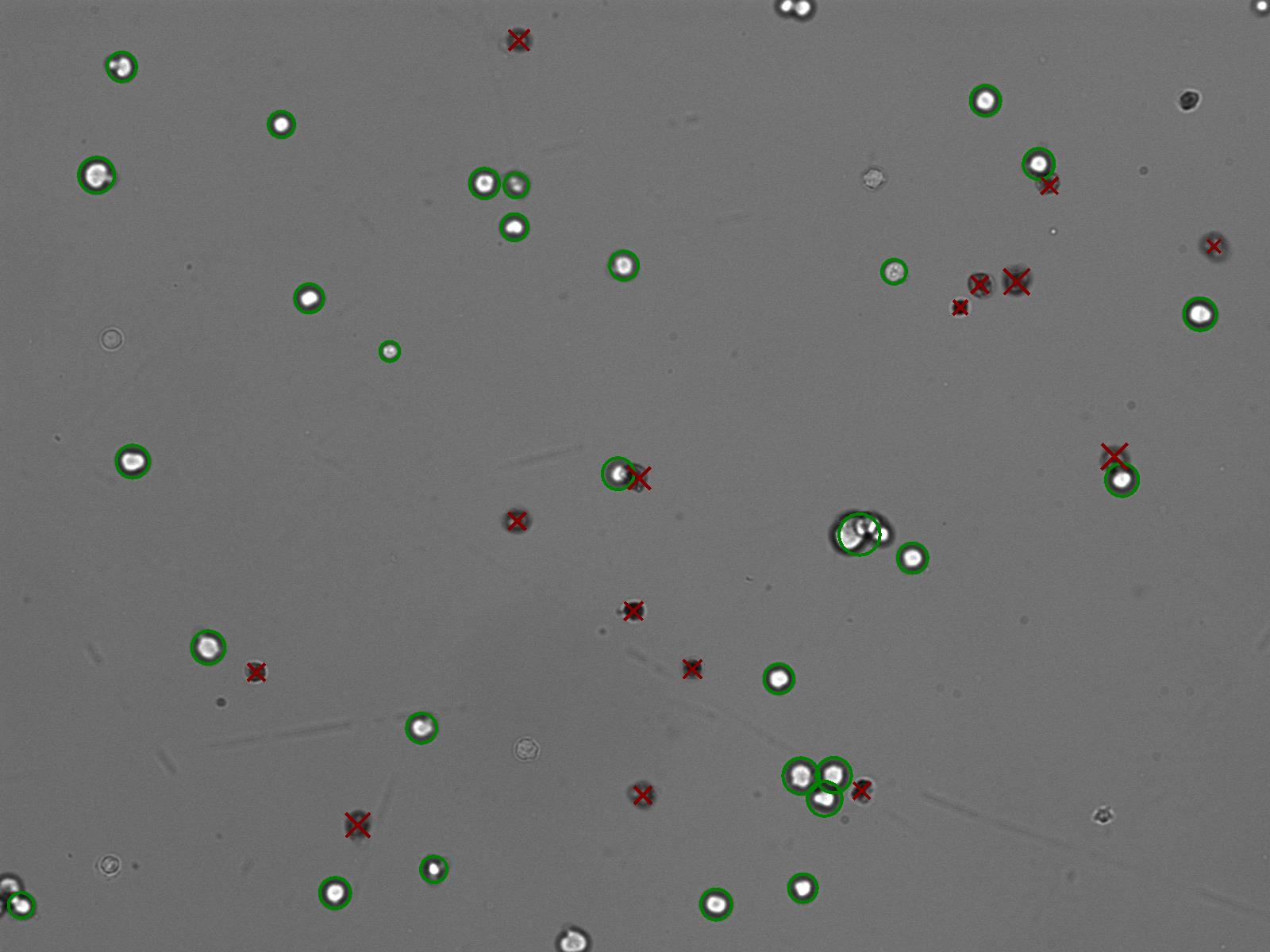

Supplement: Supplementary file 1 — Supplementary Information 1. [file 41598_2020_80576_MOESM1_ESM.zip › S1/Aggregate counts/day5/30mmHg Dec18 54 44/ML P2-010_2019-02-19_124636.bmp]

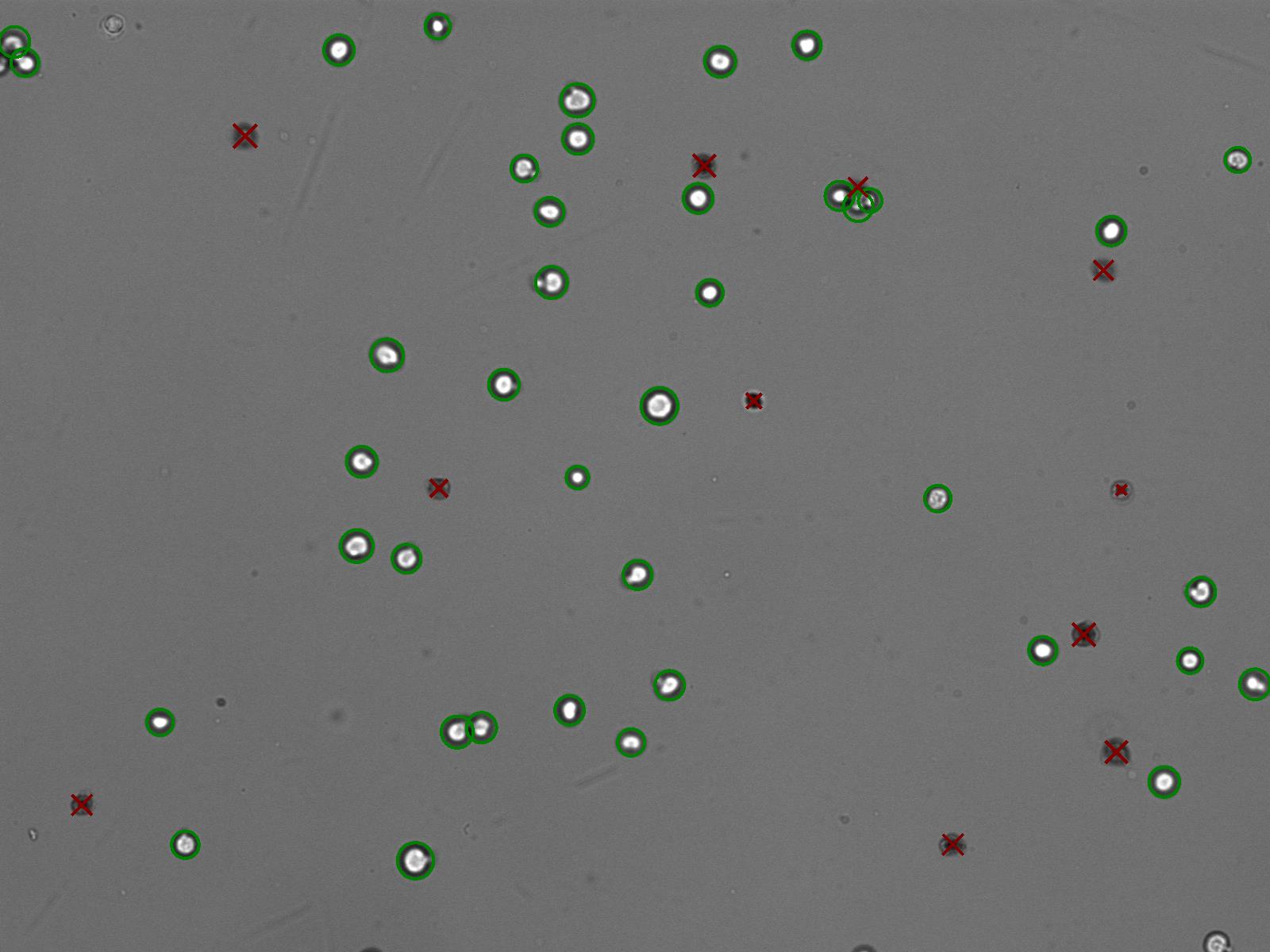

Supplement: Supplementary file 1 — Supplementary Information 1. [file 41598_2020_80576_MOESM1_ESM.zip › S1/Aggregate counts/day5/30mmHg Dec18 54 44/ML P2-011_2019-02-19_124637.bmp]

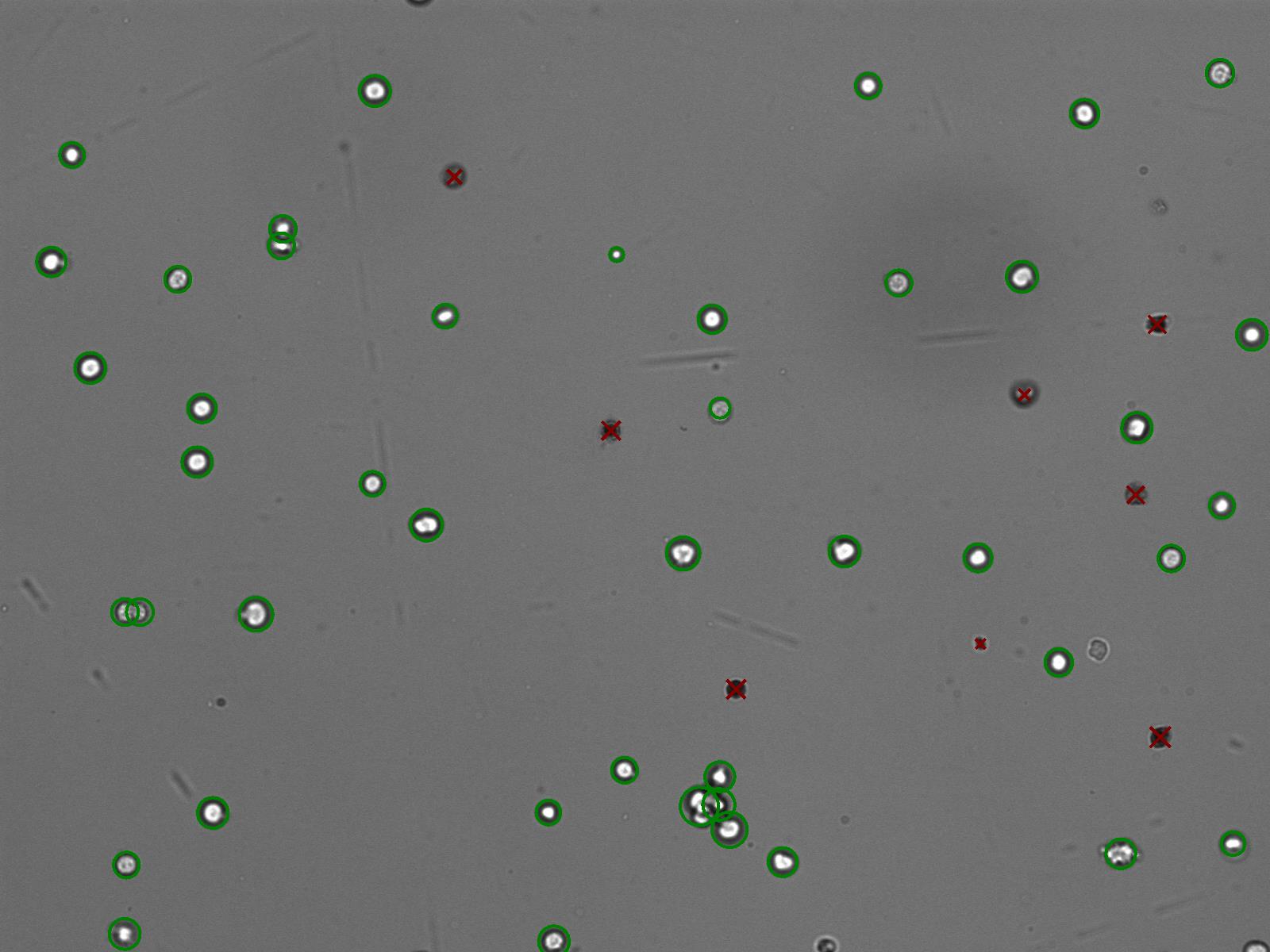

Supplement: Supplementary file 1 — Supplementary Information 1. [file 41598_2020_80576_MOESM1_ESM.zip › S1/Aggregate counts/day5/30mmHg Dec18 54 44/ML P2-012_2019-02-19_124637.bmp]

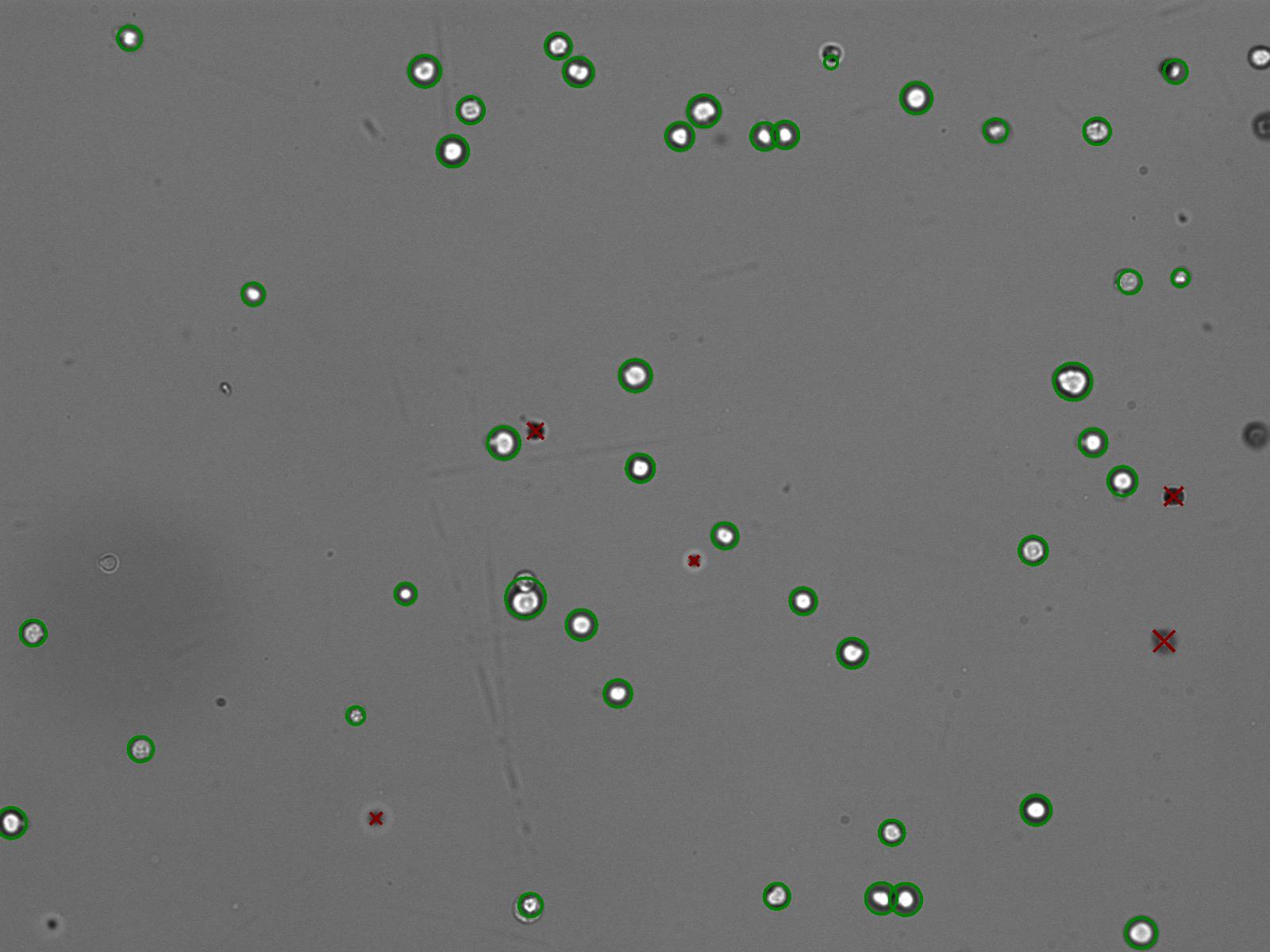

Supplement: Supplementary file 1 — Supplementary Information 1. [file 41598_2020_80576_MOESM1_ESM.zip › S1/Aggregate counts/day5/30mmHg Dec18 54 44/ML P2-013_2019-02-19_124638.bmp]

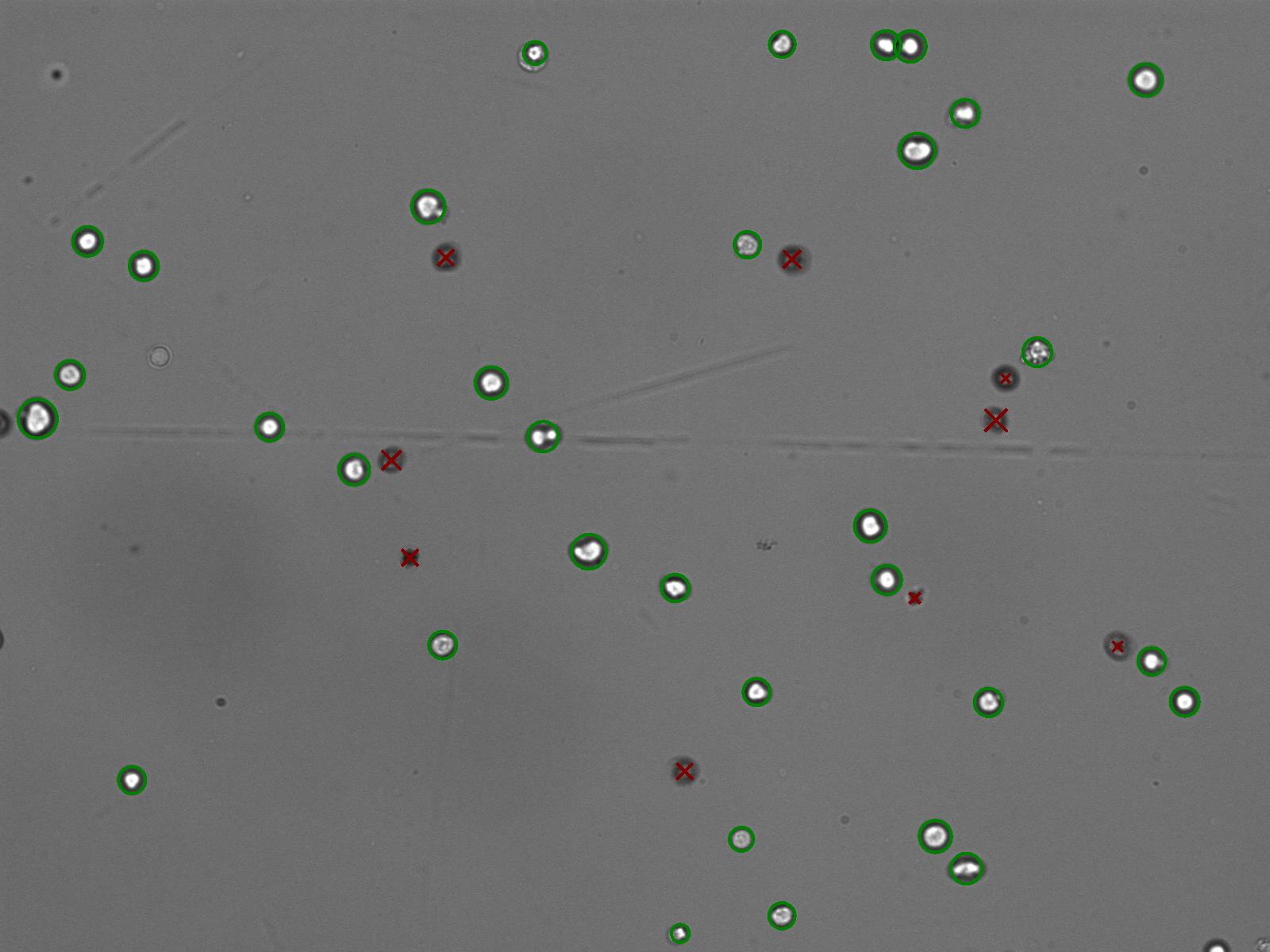

Supplement: Supplementary file 1 — Supplementary Information 1. [file 41598_2020_80576_MOESM1_ESM.zip › S1/Aggregate counts/day5/30mmHg Dec18 54 44/ML P2-014_2019-02-19_124638.bmp]

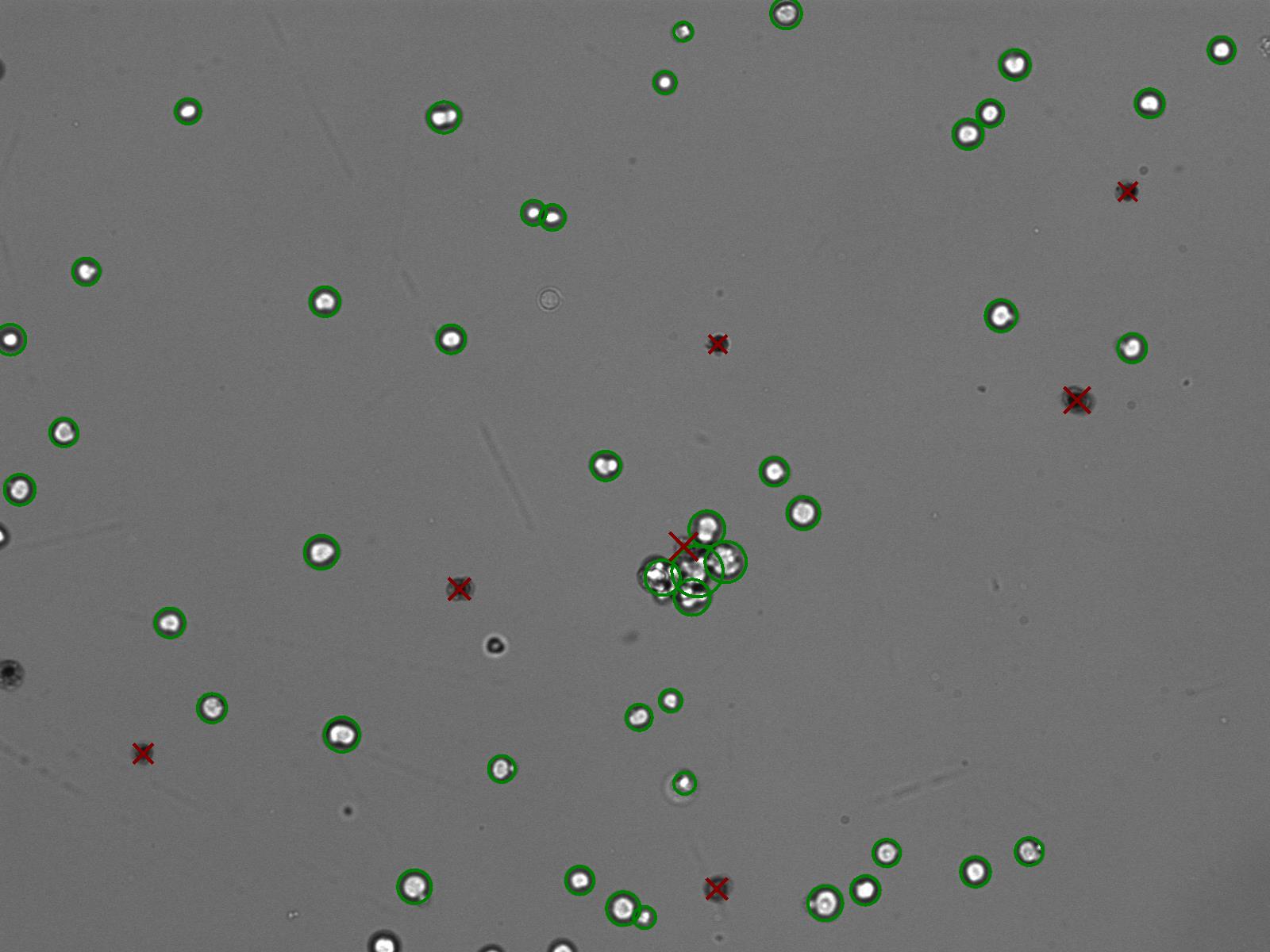

Supplement: Supplementary file 1 — Supplementary Information 1. [file 41598_2020_80576_MOESM1_ESM.zip › S1/Aggregate counts/day5/30mmHg Dec18 54 44/ML P2-015_2019-02-19_124638.bmp]

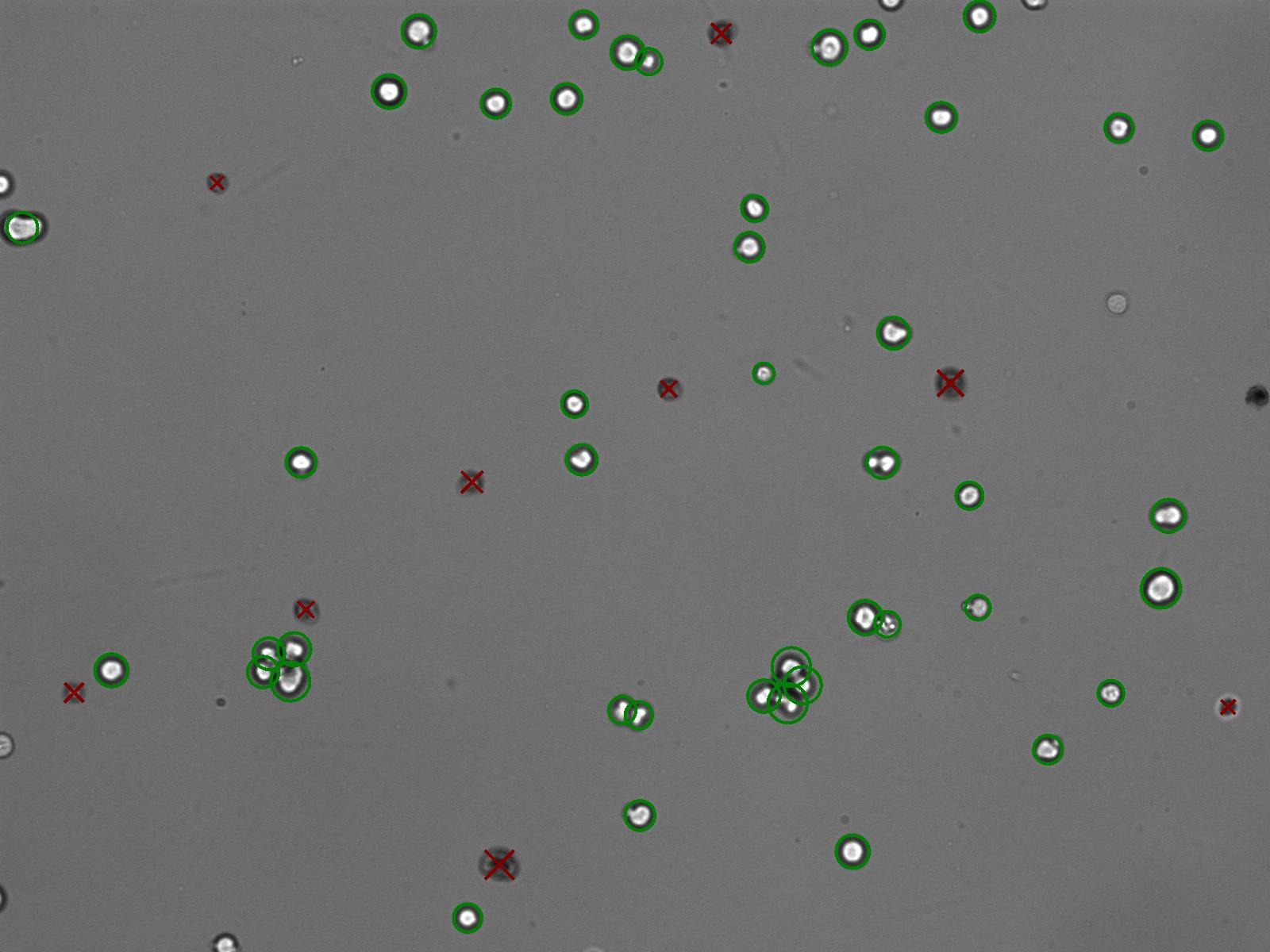

Supplement: Supplementary file 1 — Supplementary Information 1. [file 41598_2020_80576_MOESM1_ESM.zip › S1/Aggregate counts/day5/30mmHg Dec18 54 44/ML P2-016_2019-02-19_124639.bmp]

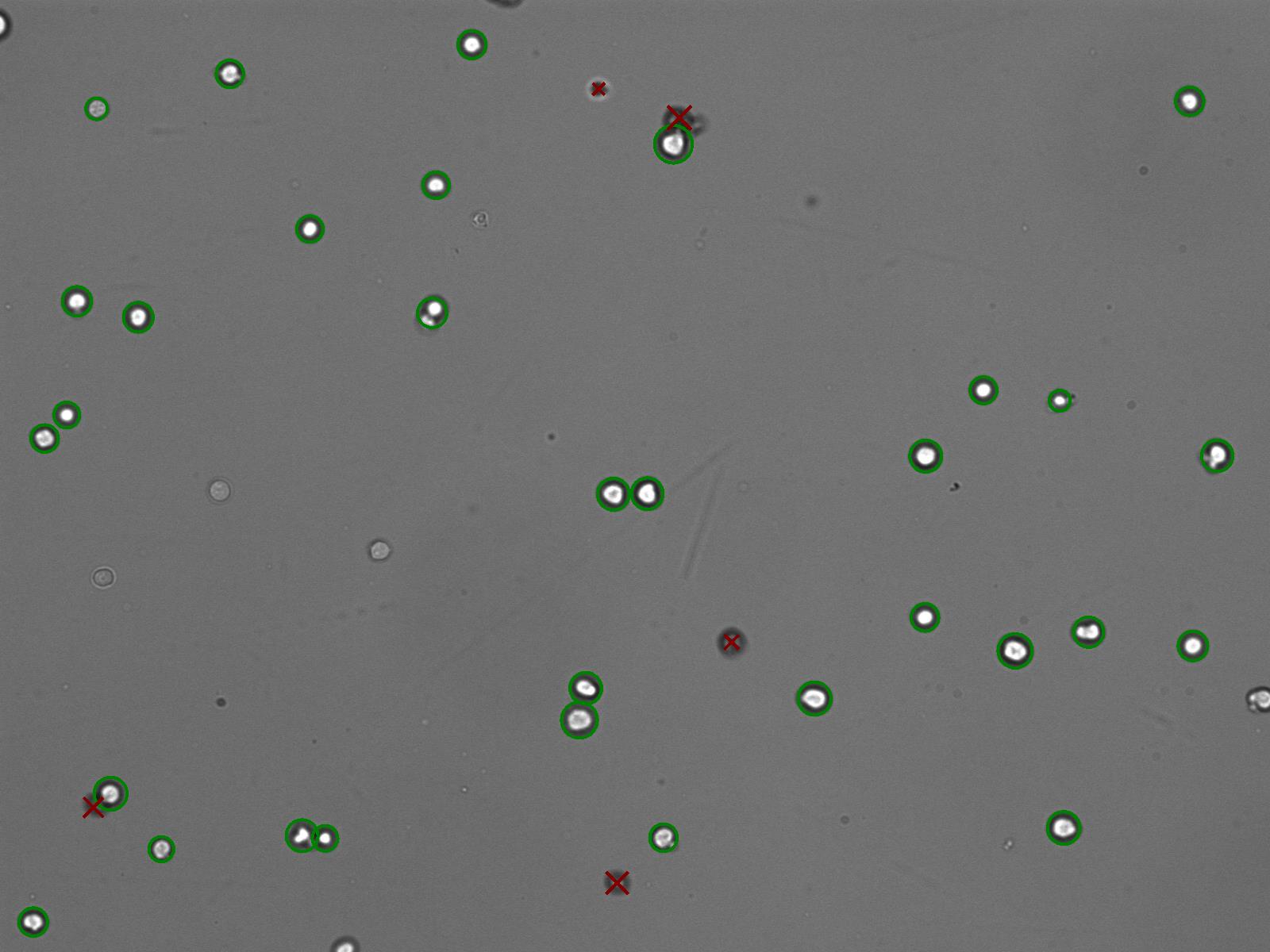

Supplement: Supplementary file 1 — Supplementary Information 1. [file 41598_2020_80576_MOESM1_ESM.zip › S1/Aggregate counts/day5/30mmHg Dec18 54 44/ML P2-017_2019-02-19_124639.bmp]

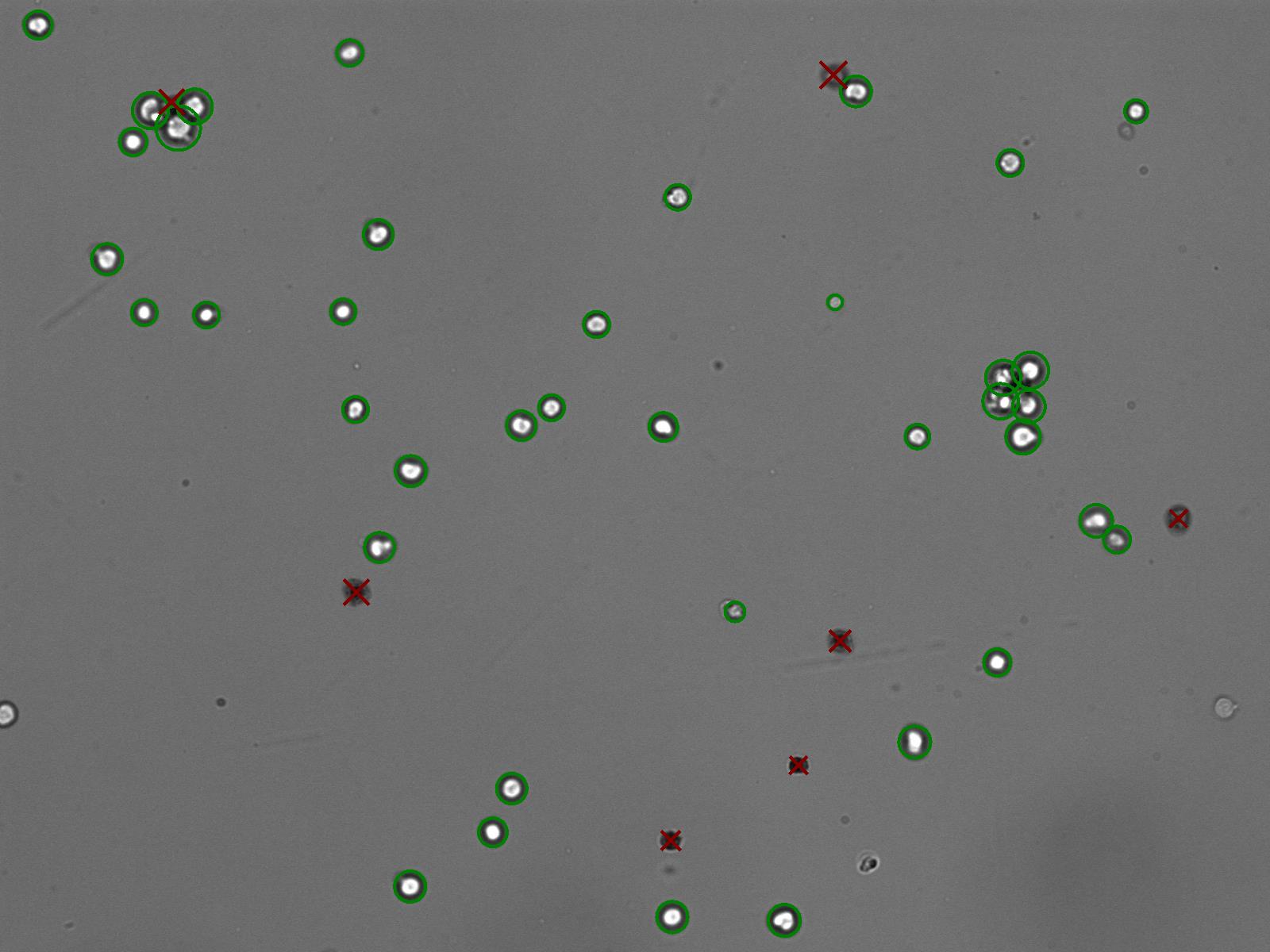

Supplement: Supplementary file 1 — Supplementary Information 1. [file 41598_2020_80576_MOESM1_ESM.zip › S1/Aggregate counts/day5/30mmHg Dec18 54 44/ML P2-018_2019-02-19_124639.bmp]

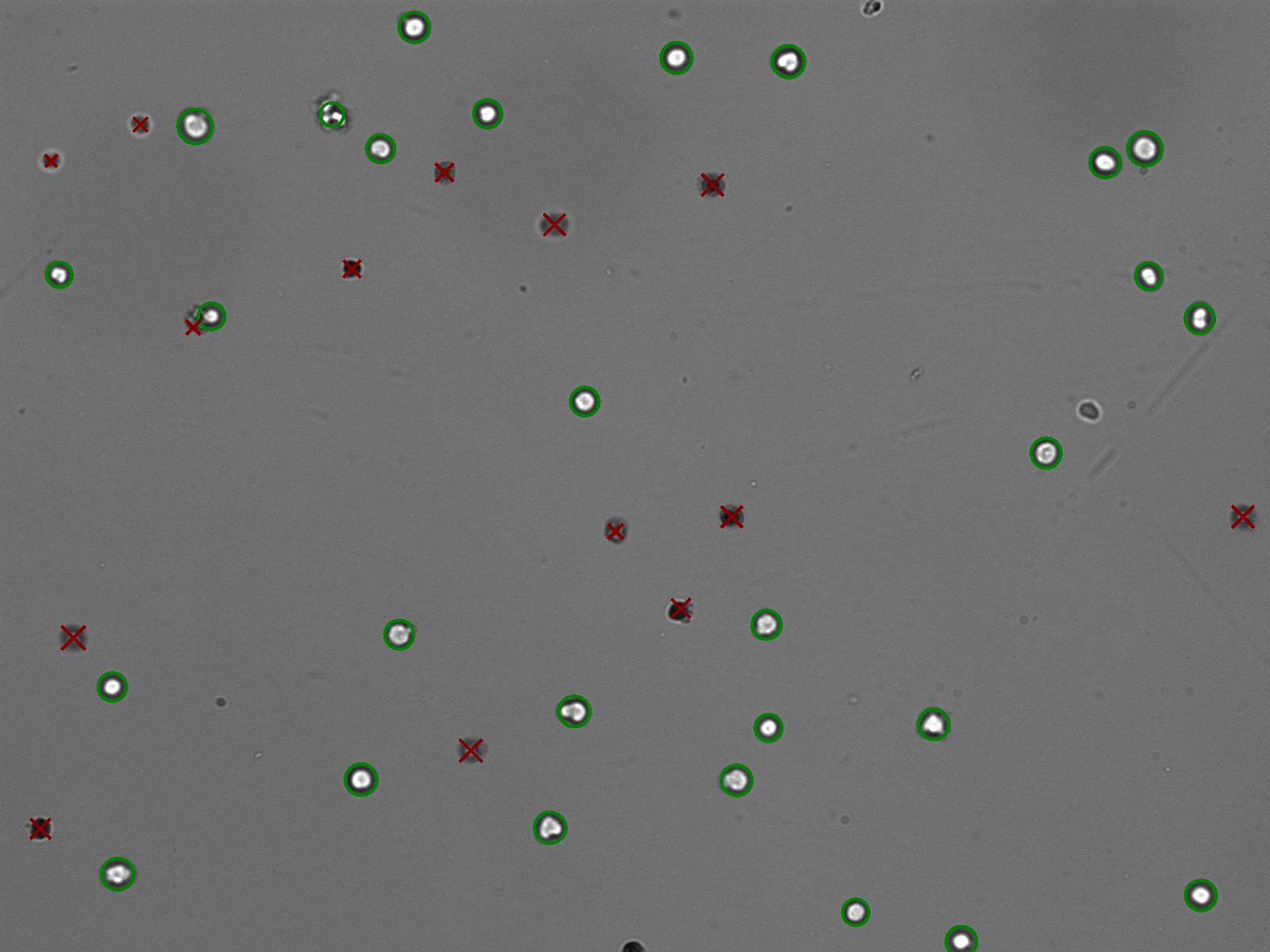

Supplement: Supplementary file 1 — Supplementary Information 1. [file 41598_2020_80576_MOESM1_ESM.zip › S1/Aggregate counts/day5/30mmHg Dec18 54 44/ML P2-019_2019-02-19_124640.bmp]

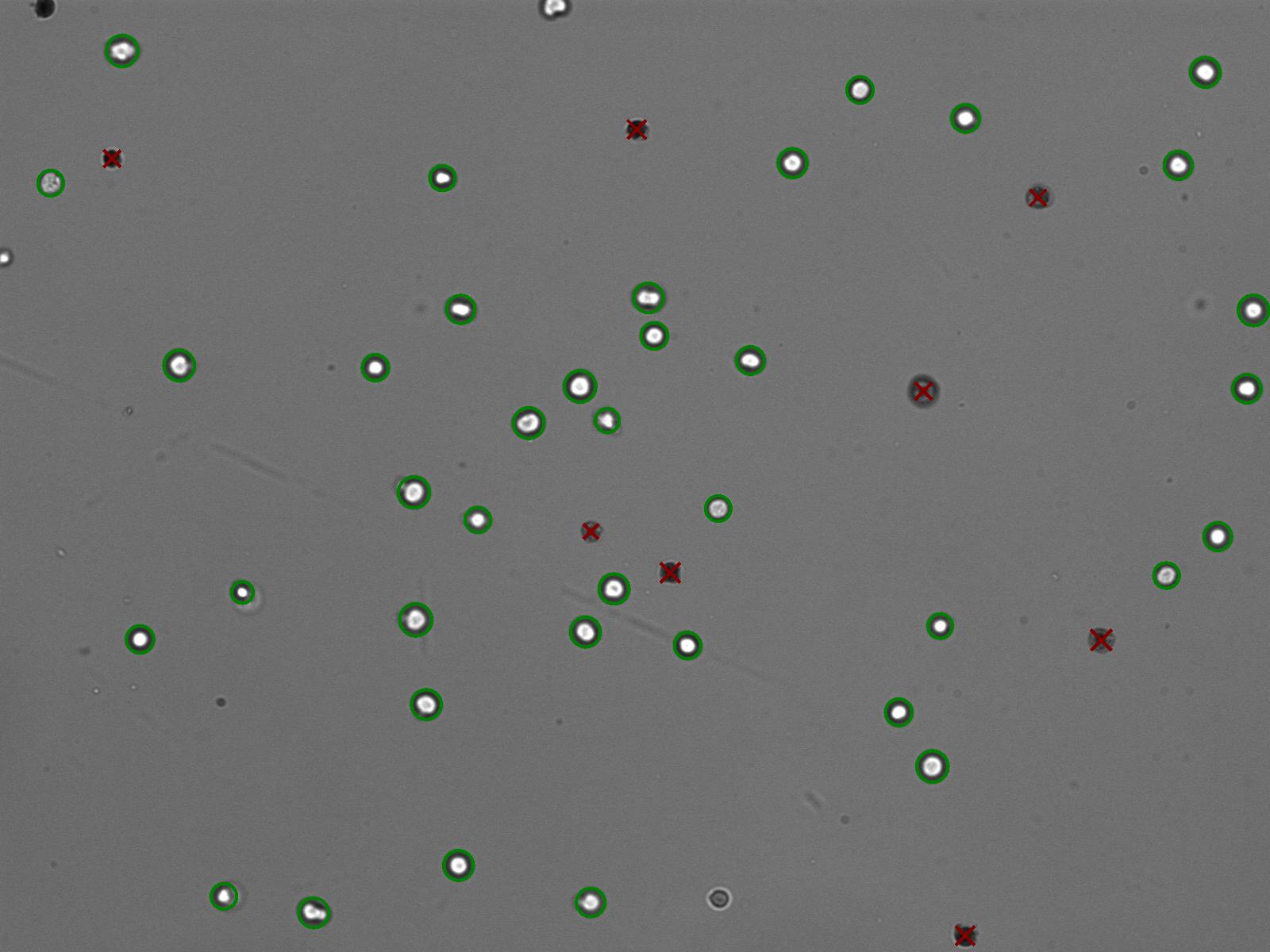

Supplement: Supplementary file 1 — Supplementary Information 1. [file 41598_2020_80576_MOESM1_ESM.zip › S1/Aggregate counts/day5/30mmHg Dec18 54 44/ML P2-020_2019-02-19_124640.bmp]

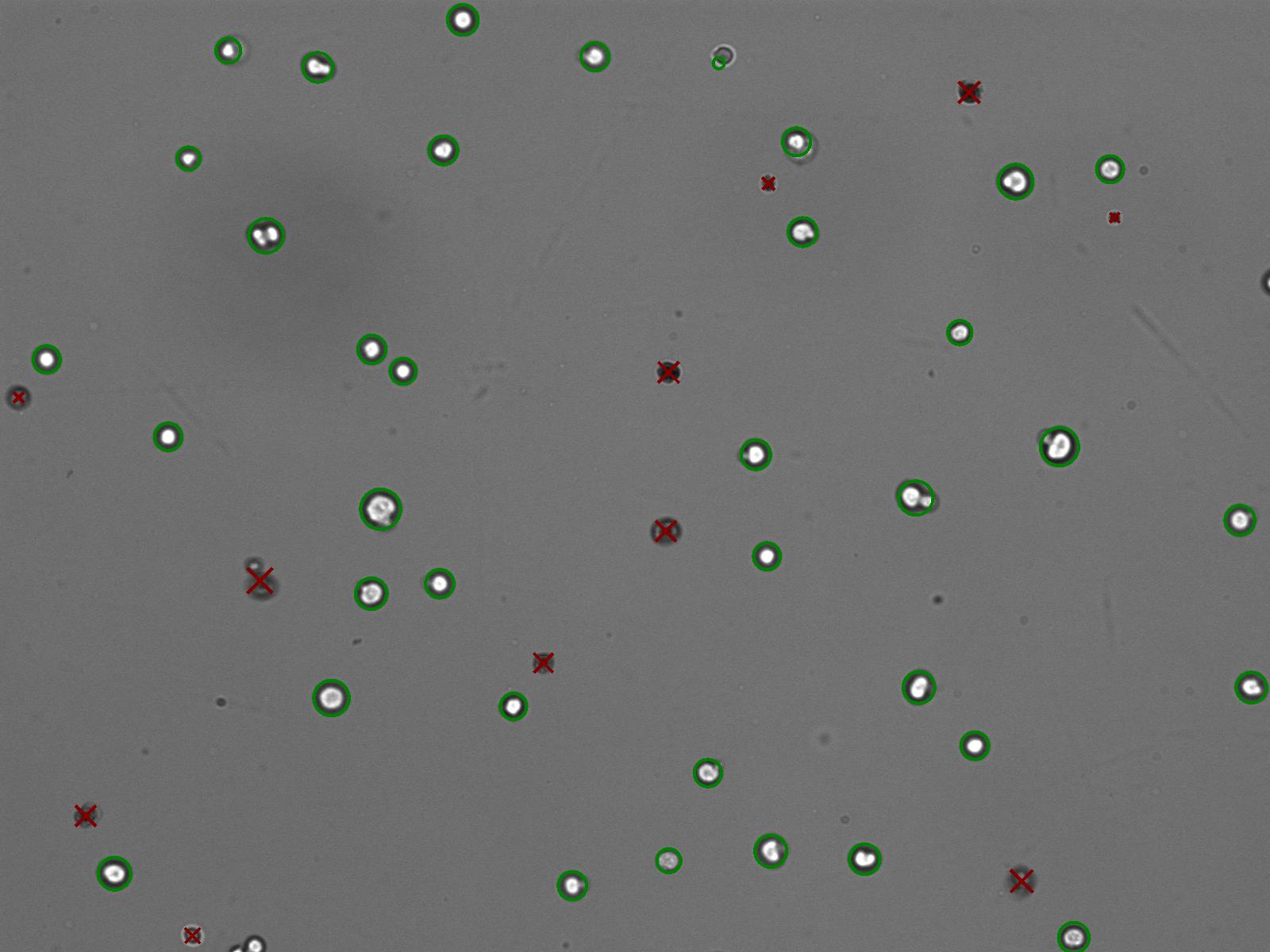

Supplement: Supplementary file 1 — Supplementary Information 1. [file 41598_2020_80576_MOESM1_ESM.zip › S1/Aggregate counts/day5/30mmHg Dec18 54 44/ML P2-021_2019-02-19_124640.bmp]

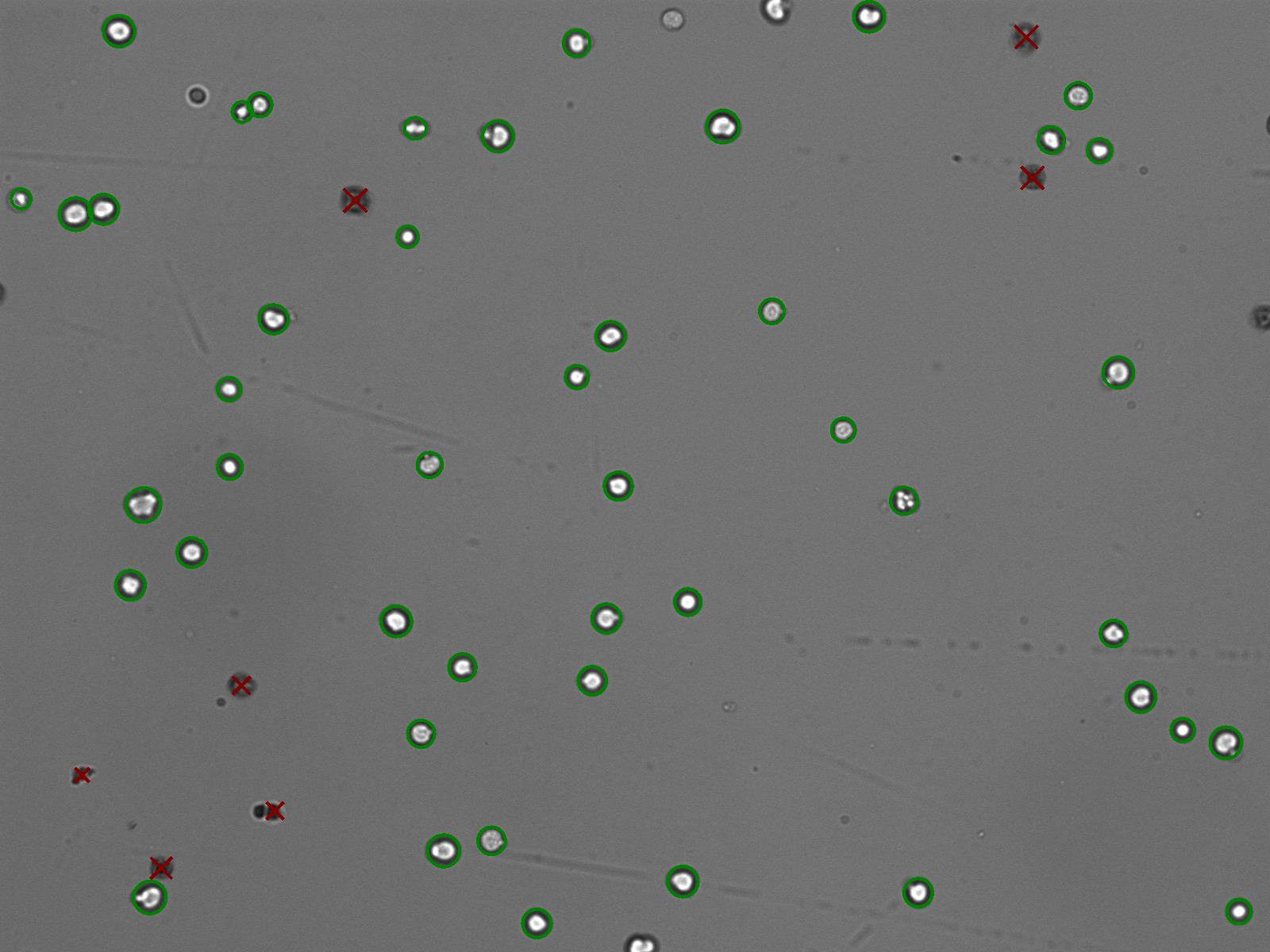

Supplement: Supplementary file 1 — Supplementary Information 1. [file 41598_2020_80576_MOESM1_ESM.zip › S1/Aggregate counts/day5/30mmHg Dec18 54 44/ML P2-022_2019-02-19_124641.bmp]

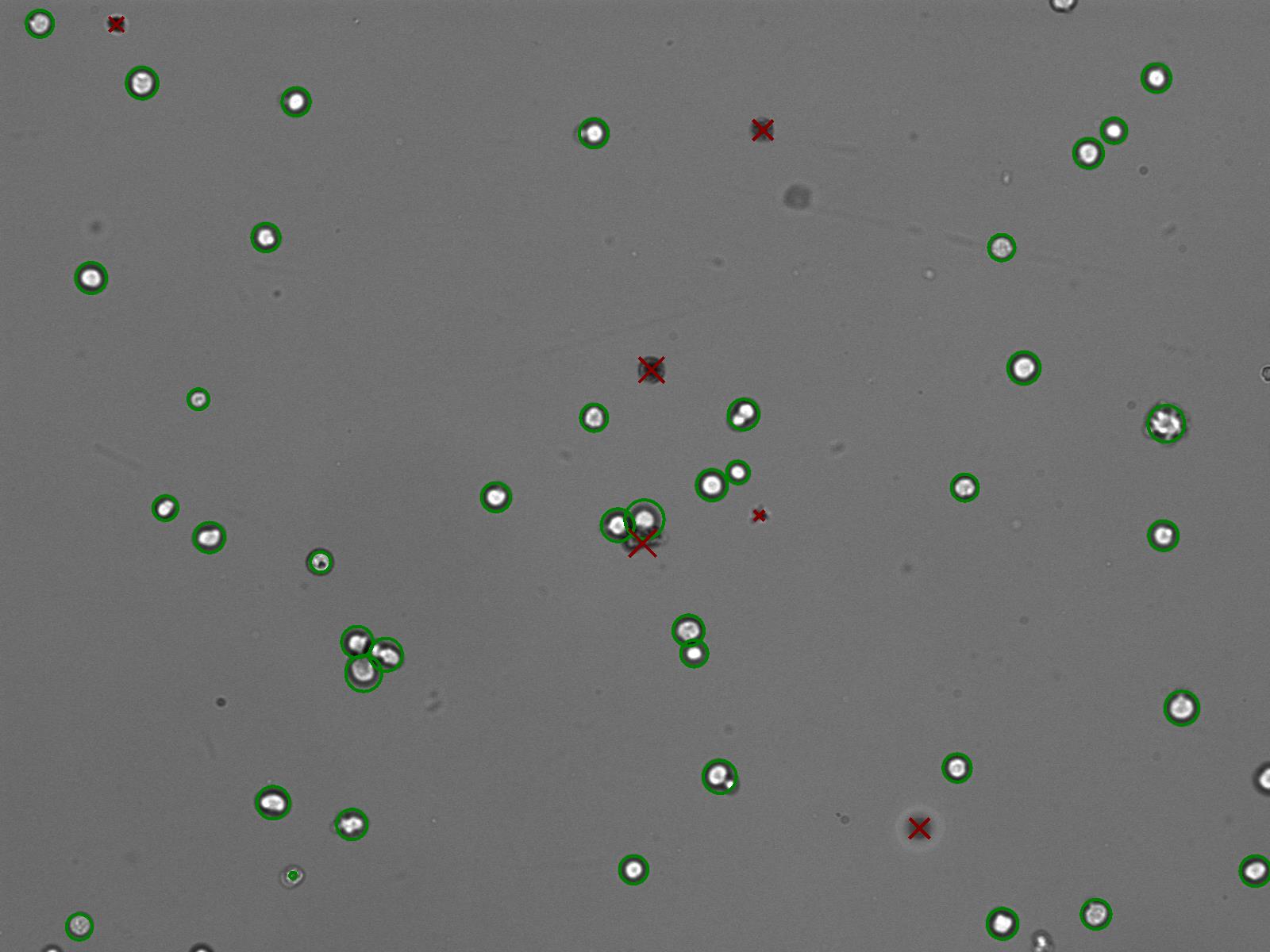

Supplement: Supplementary file 1 — Supplementary Information 1. [file 41598_2020_80576_MOESM1_ESM.zip › S1/Aggregate counts/day5/30mmHg Dec18 54 44/ML P2-023_2019-02-19_124641.bmp]

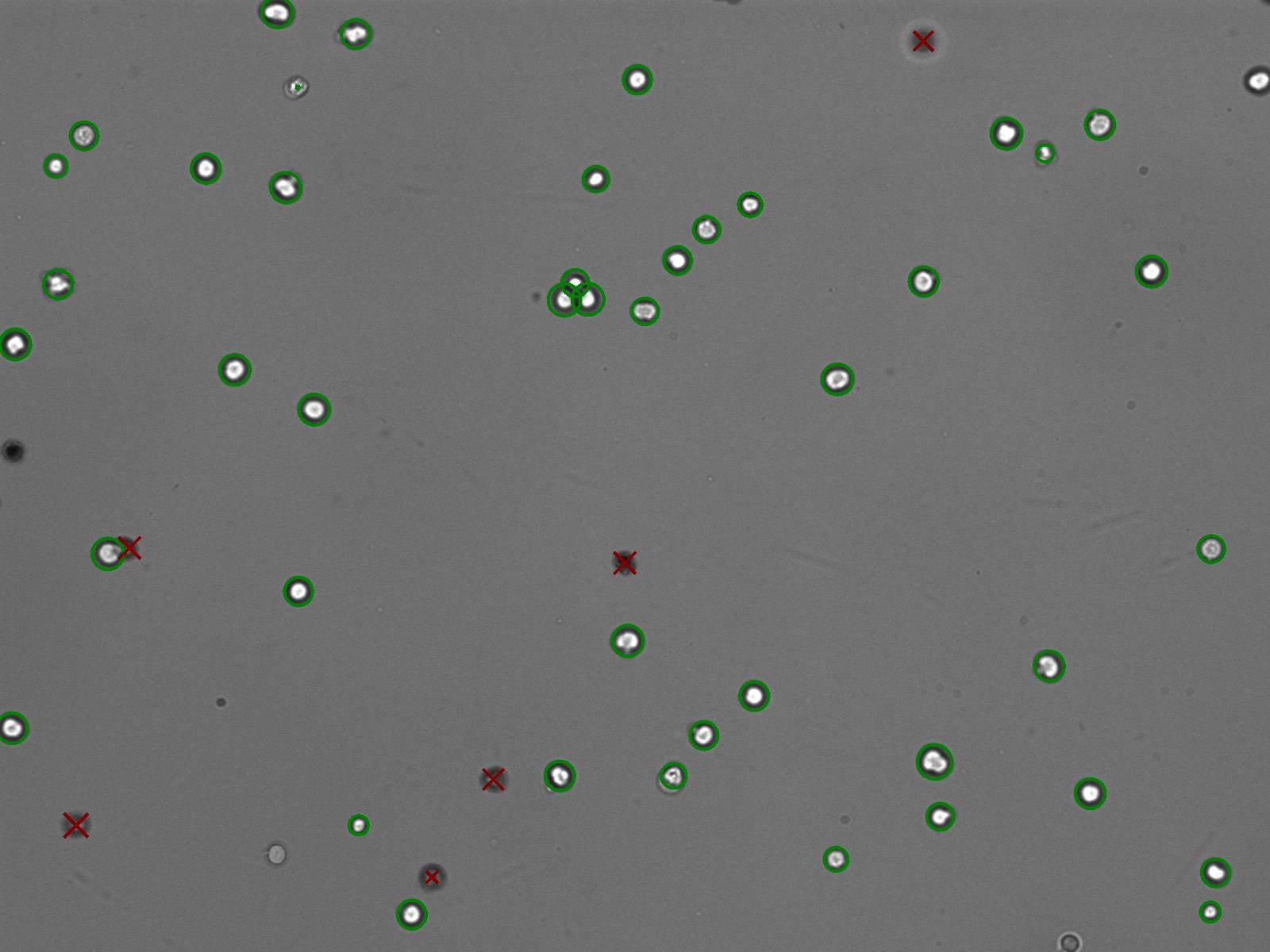

Supplement: Supplementary file 1 — Supplementary Information 1. [file 41598_2020_80576_MOESM1_ESM.zip › S1/Aggregate counts/day5/30mmHg Dec18 54 44/ML P2-024_2019-02-19_124641.bmp]

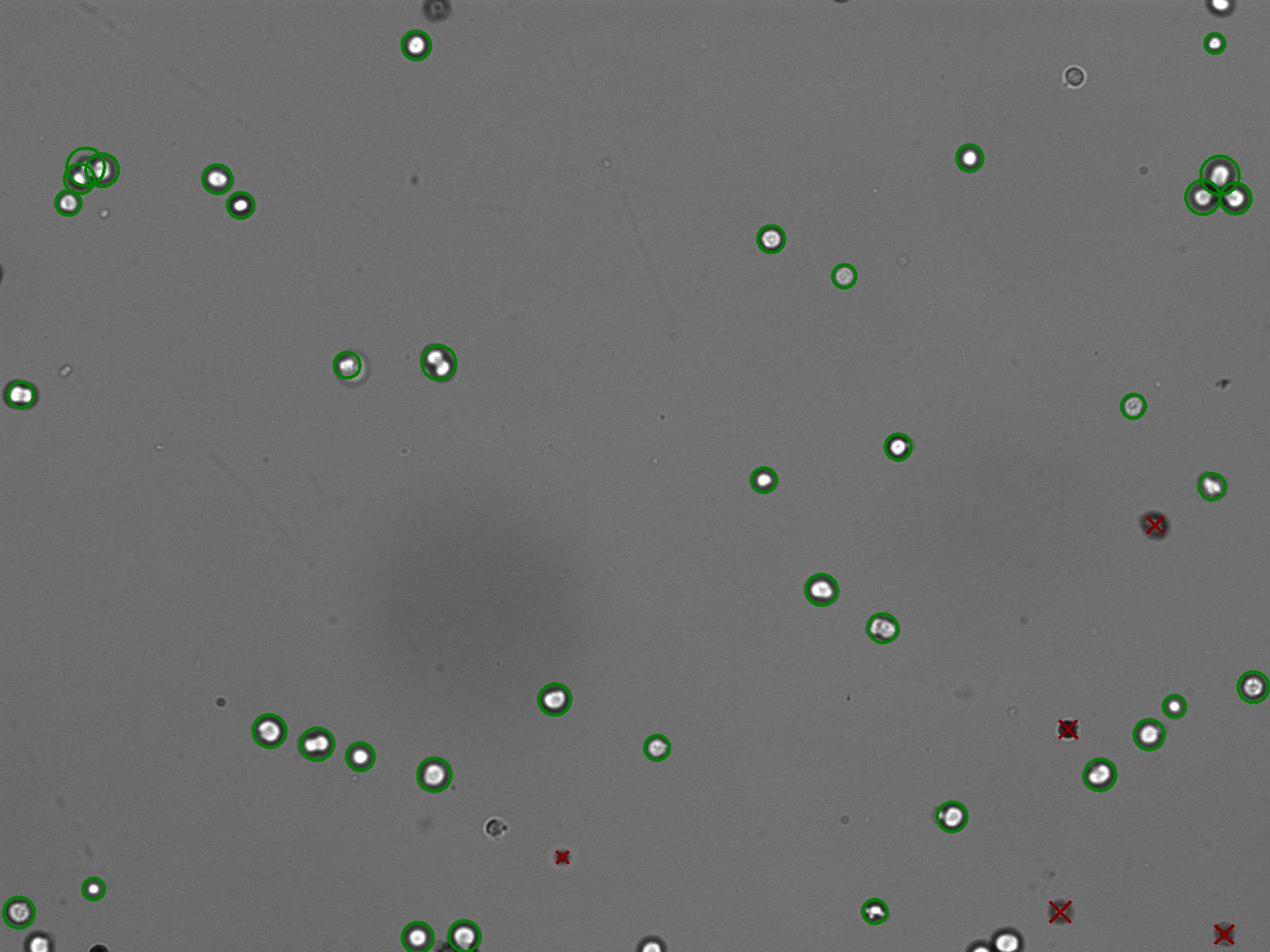

Supplement: Supplementary file 1 — Supplementary Information 1. [file 41598_2020_80576_MOESM1_ESM.zip › S1/Aggregate counts/day5/30mmHg Dec18 54 44/ML P2-025_2019-02-19_124642.bmp]

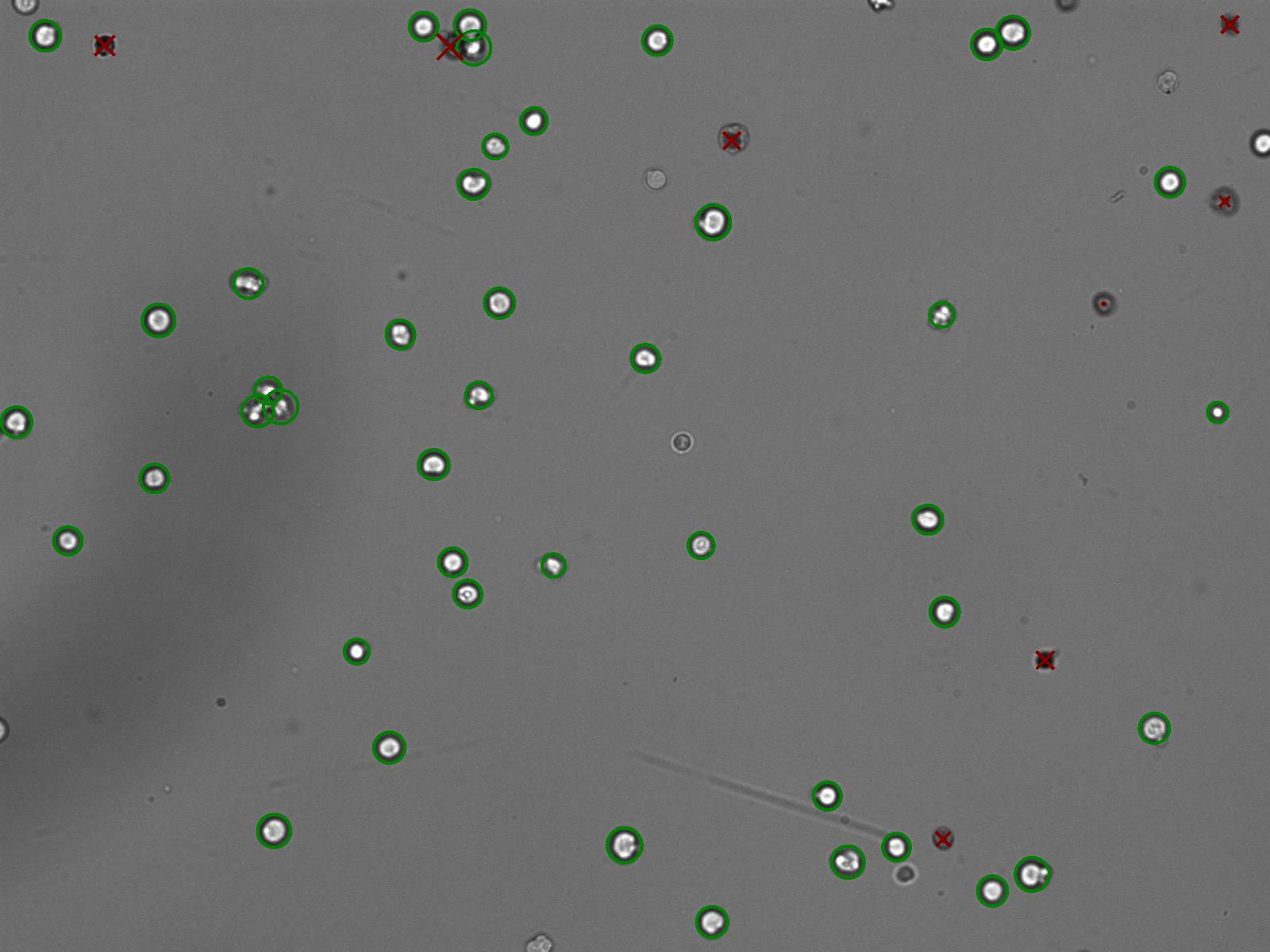

Supplement: Supplementary file 1 — Supplementary Information 1. [file 41598_2020_80576_MOESM1_ESM.zip › S1/Aggregate counts/day5/30mmHg Dec18 54 44/ML P2-026_2019-02-19_124642.bmp]

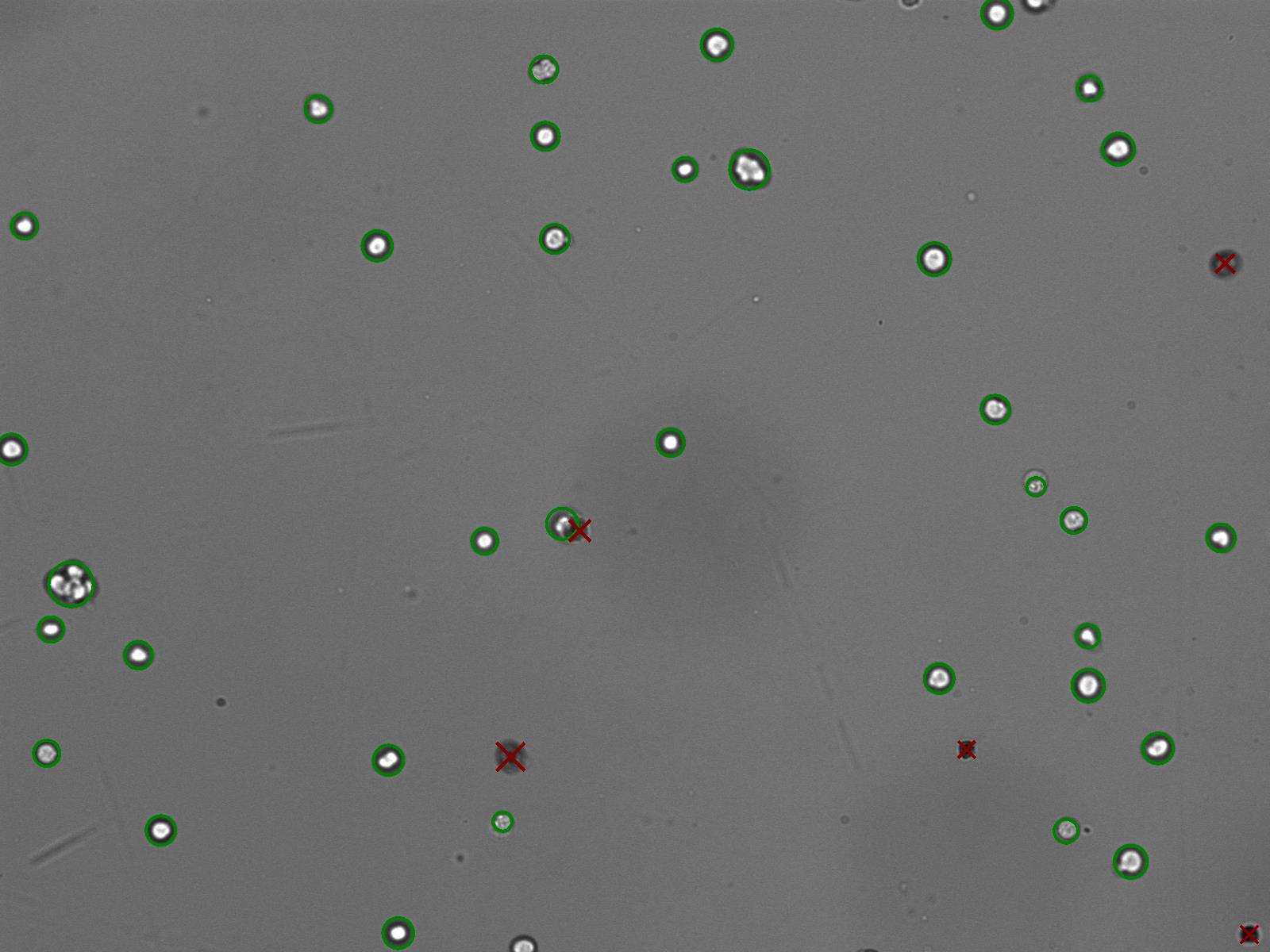

Supplement: Supplementary file 1 — Supplementary Information 1. [file 41598_2020_80576_MOESM1_ESM.zip › S1/Aggregate counts/day5/30mmHg Dec18 54 44/ML P2-027_2019-02-19_124643.bmp]

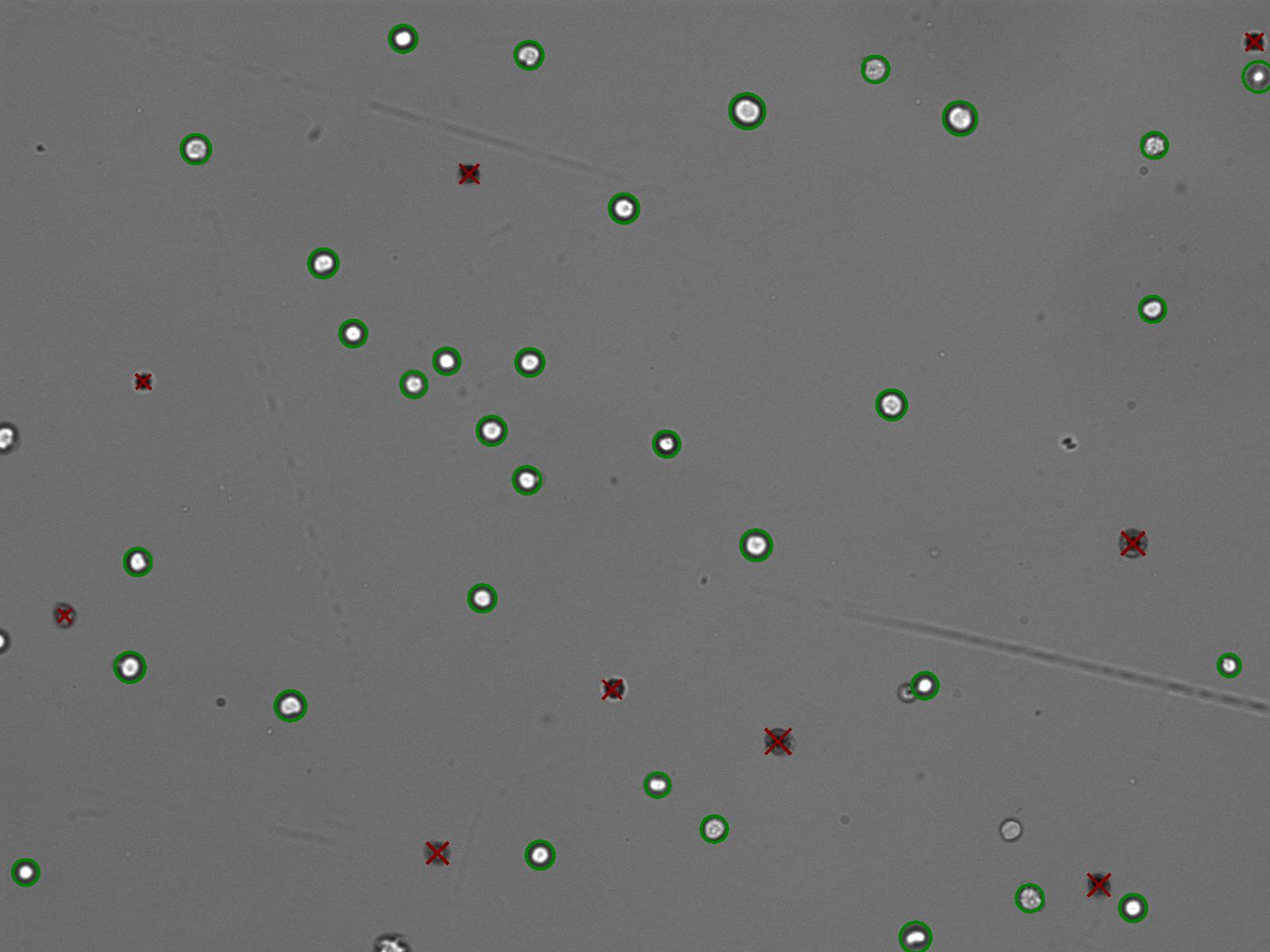

Supplement: Supplementary file 1 — Supplementary Information 1. [file 41598_2020_80576_MOESM1_ESM.zip › S1/Aggregate counts/day5/30mmHg Dec18 54 44/ML P2-028_2019-02-19_124643.bmp]

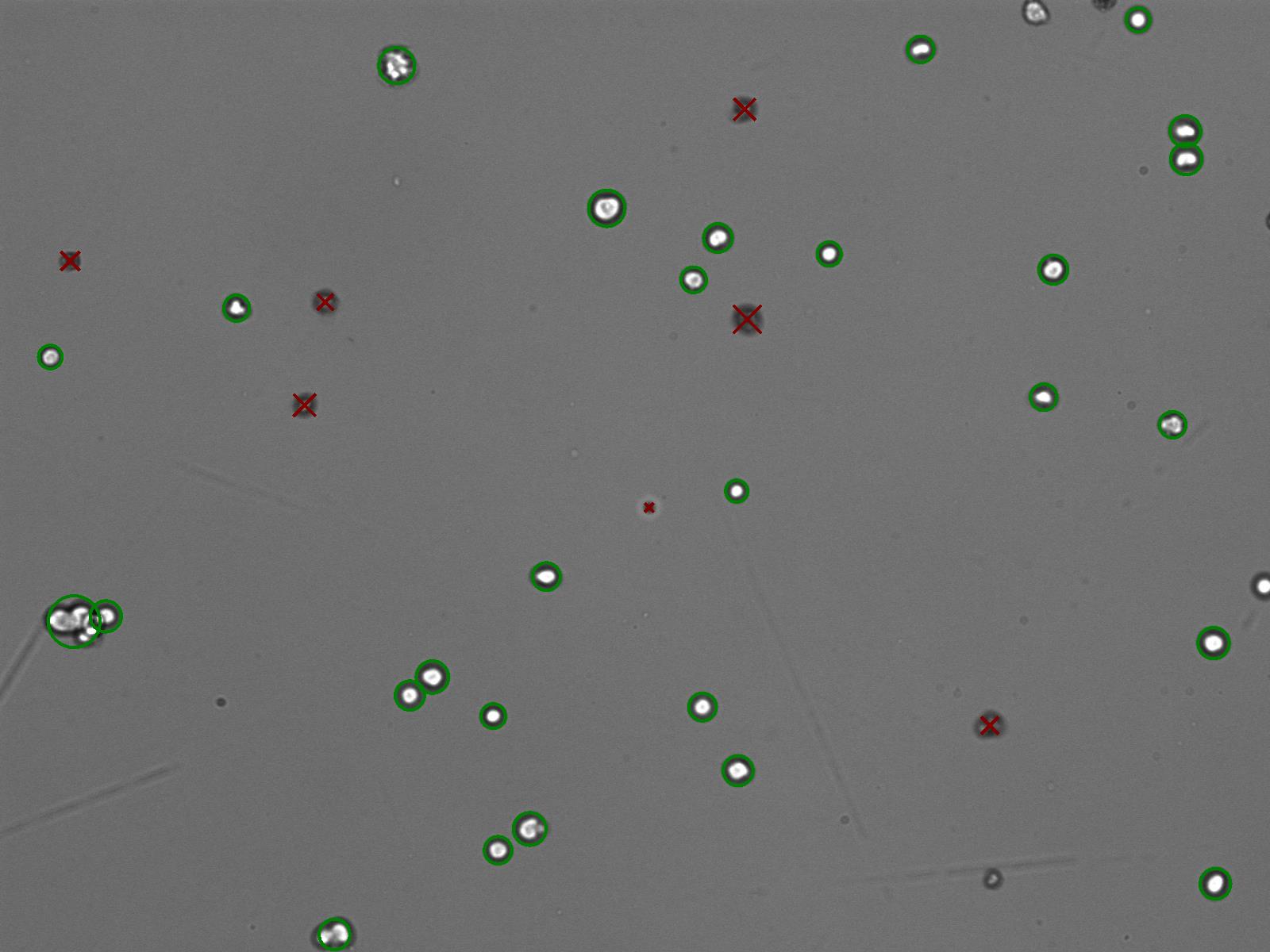

Supplement: Supplementary file 1 — Supplementary Information 1. [file 41598_2020_80576_MOESM1_ESM.zip › S1/Aggregate counts/day5/30mmHg Dec18 54 44/ML P2-029_2019-02-19_124643.bmp]

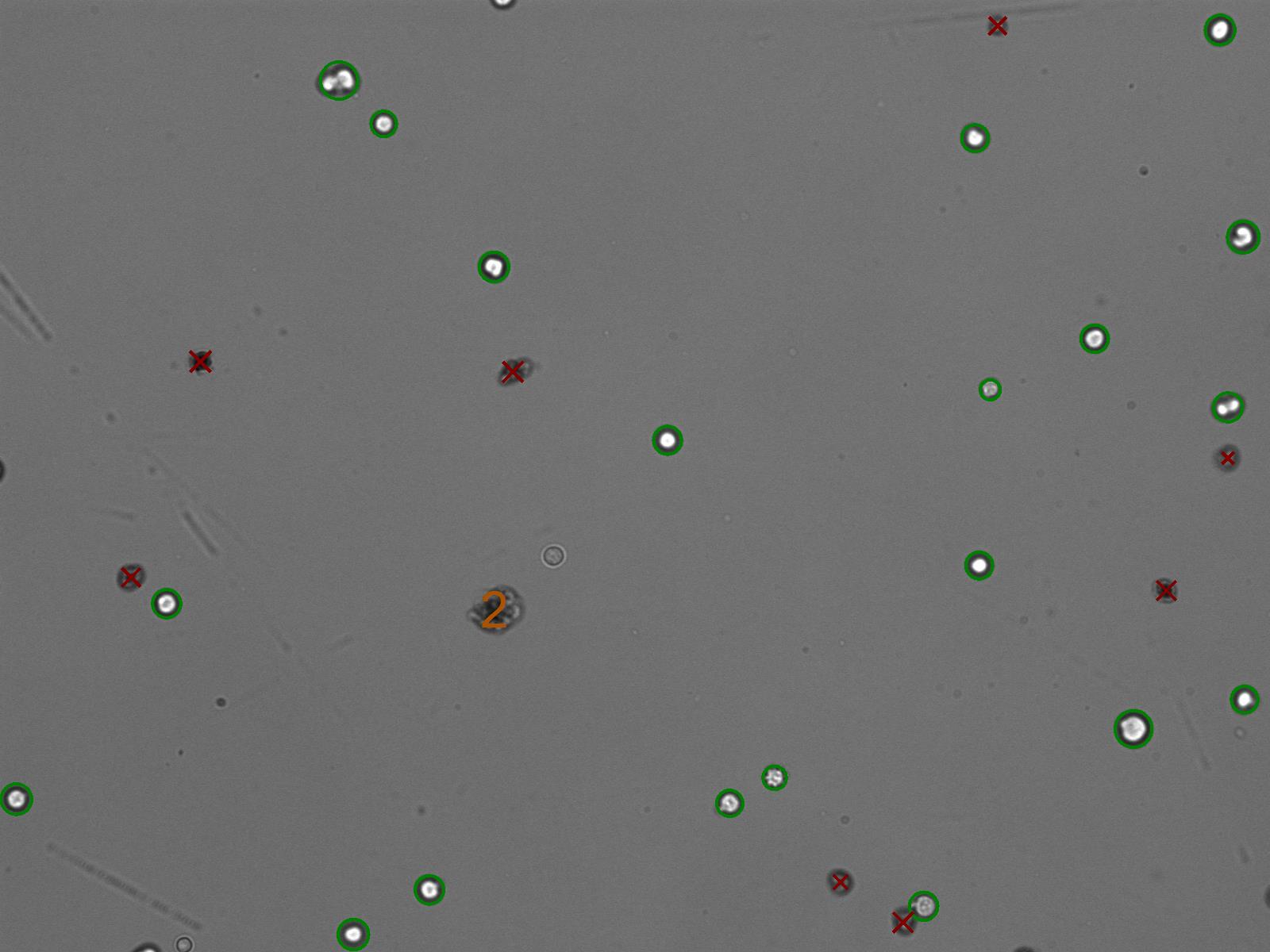

Supplement: Supplementary file 1 — Supplementary Information 1. [file 41598_2020_80576_MOESM1_ESM.zip › S1/Aggregate counts/day5/30mmHg Dec18 54 44/ML P2-030_2019-02-19_124644.bmp]

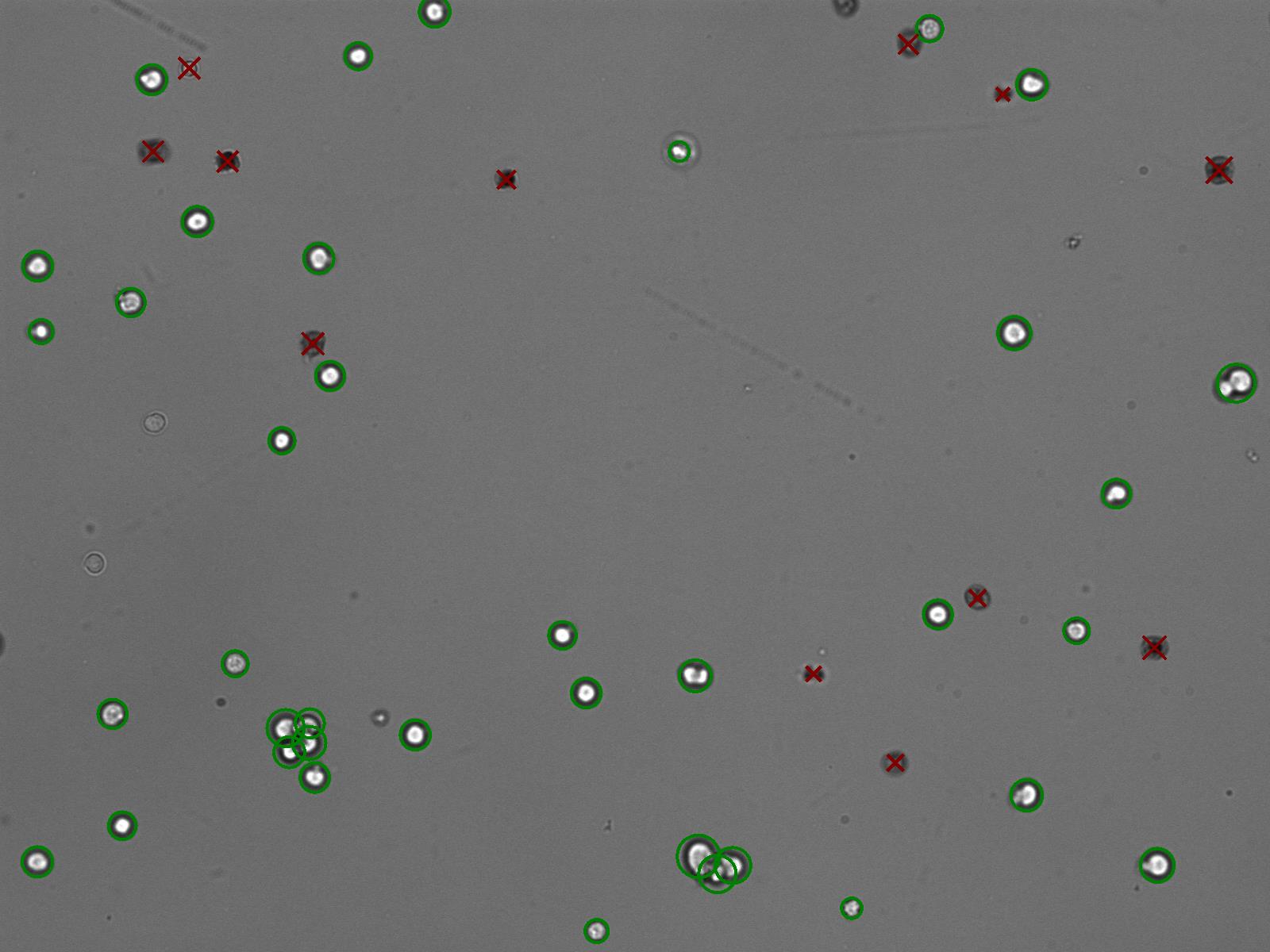

Supplement: Supplementary file 1 — Supplementary Information 1. [file 41598_2020_80576_MOESM1_ESM.zip › S1/Aggregate counts/day5/30mmHg Dec18 54 44/ML P2-031_2019-02-19_124644.bmp]

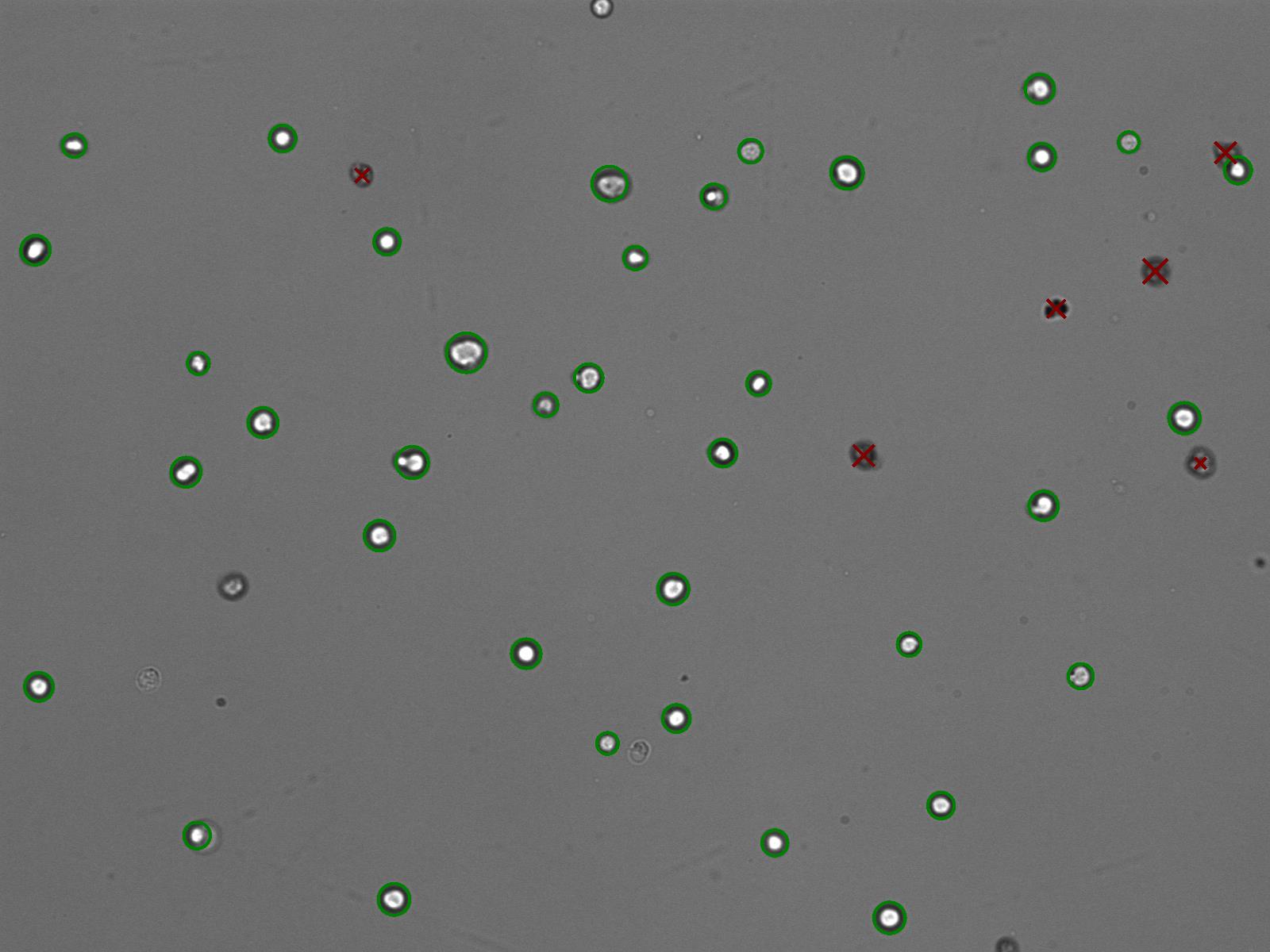

Supplement: Supplementary file 1 — Supplementary Information 1. [file 41598_2020_80576_MOESM1_ESM.zip › S1/Aggregate counts/day5/30mmHg Dec18 54 44/ML P2-032_2019-02-19_124644.bmp]

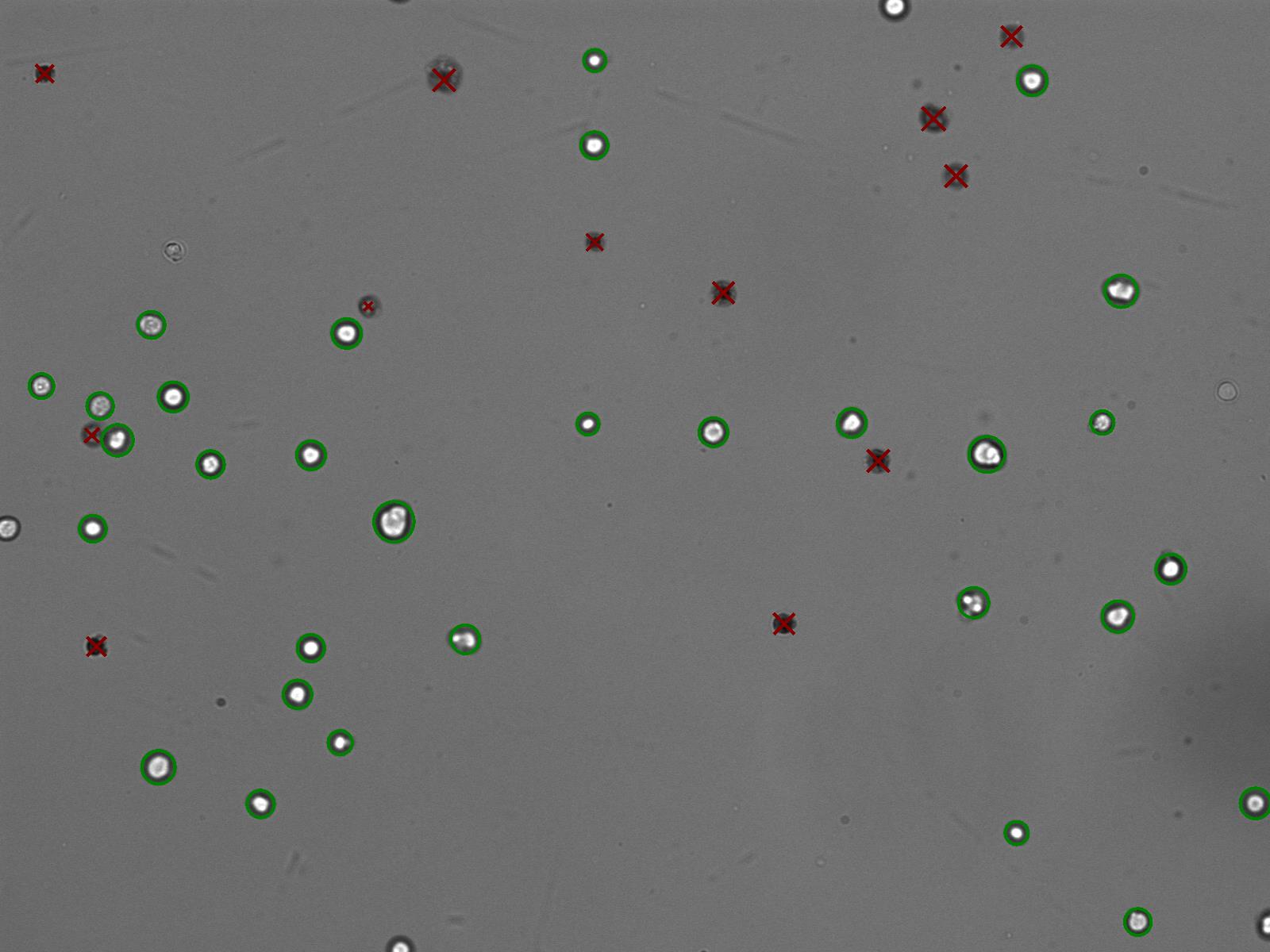

Supplement: Supplementary file 1 — Supplementary Information 1. [file 41598_2020_80576_MOESM1_ESM.zip › S1/Aggregate counts/day5/30mmHg Dec18 54 44/ML P2-033_2019-02-19_124645.bmp]

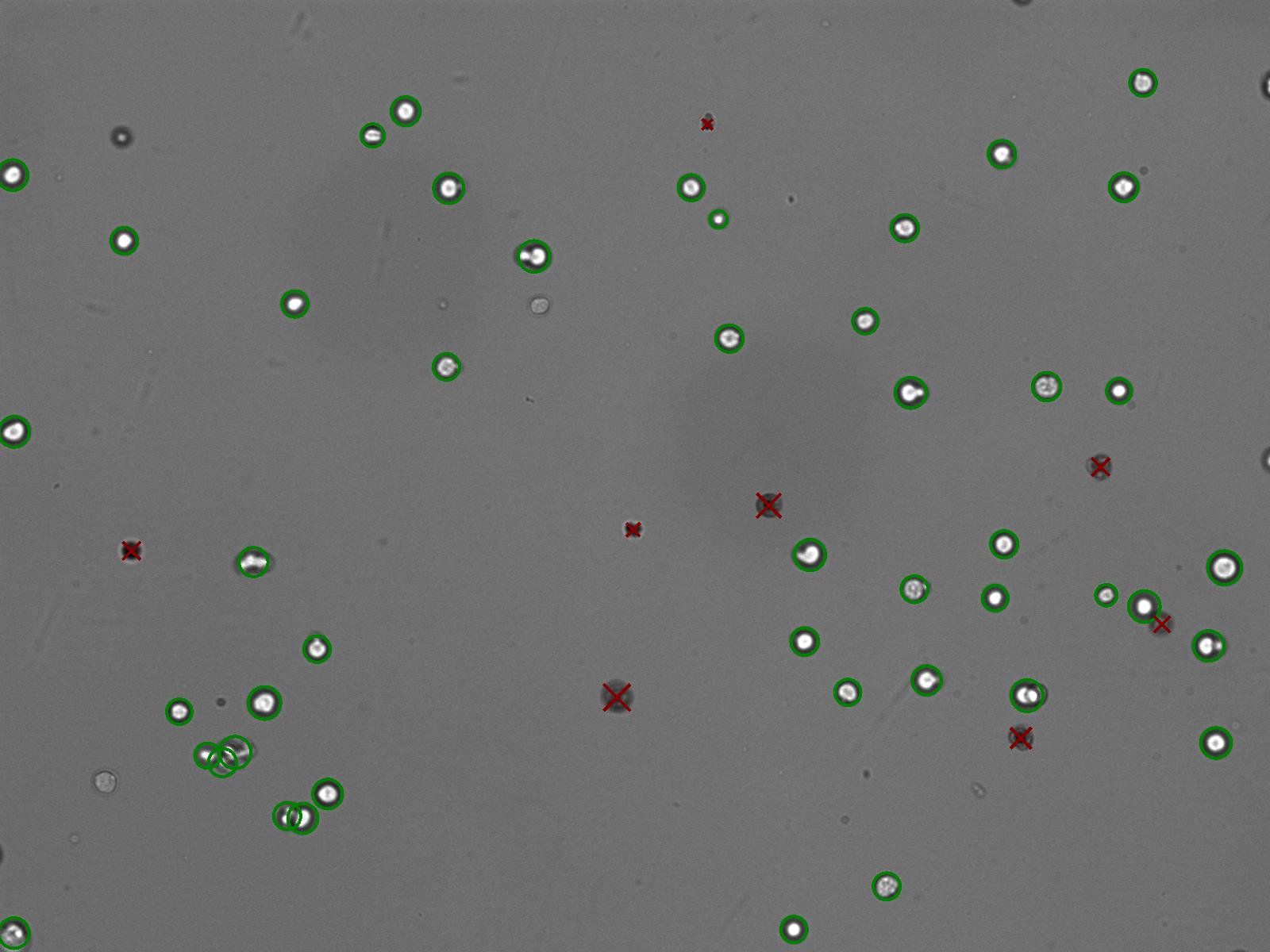

Supplement: Supplementary file 1 — Supplementary Information 1. [file 41598_2020_80576_MOESM1_ESM.zip › S1/Aggregate counts/day5/30mmHg Dec18 54 44/ML P2-034_2019-02-19_124645.bmp]

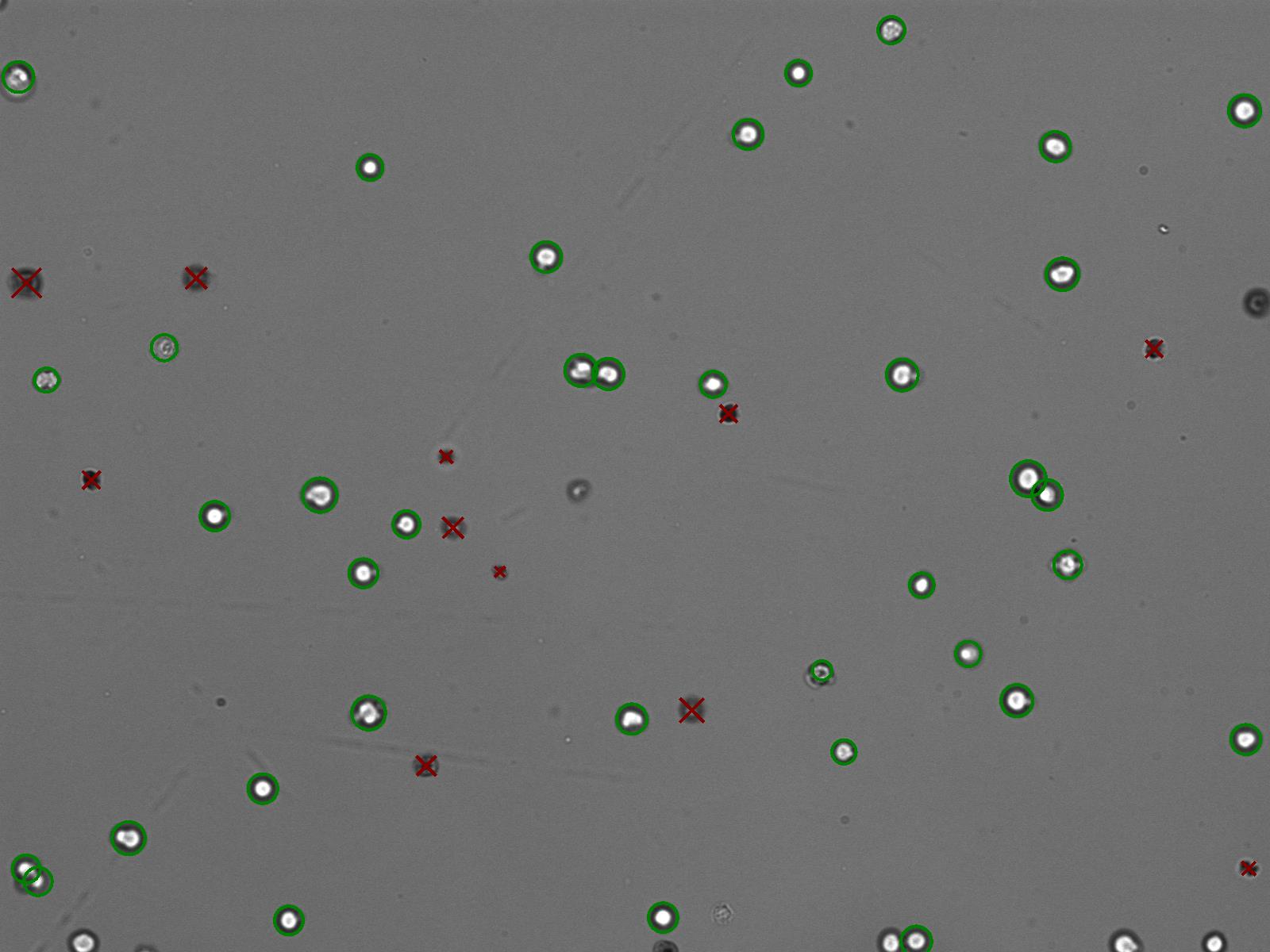

Supplement: Supplementary file 1 — Supplementary Information 1. [file 41598_2020_80576_MOESM1_ESM.zip › S1/Aggregate counts/day5/30mmHg Dec18 54 44/ML P2-035_2019-02-19_124645.bmp]

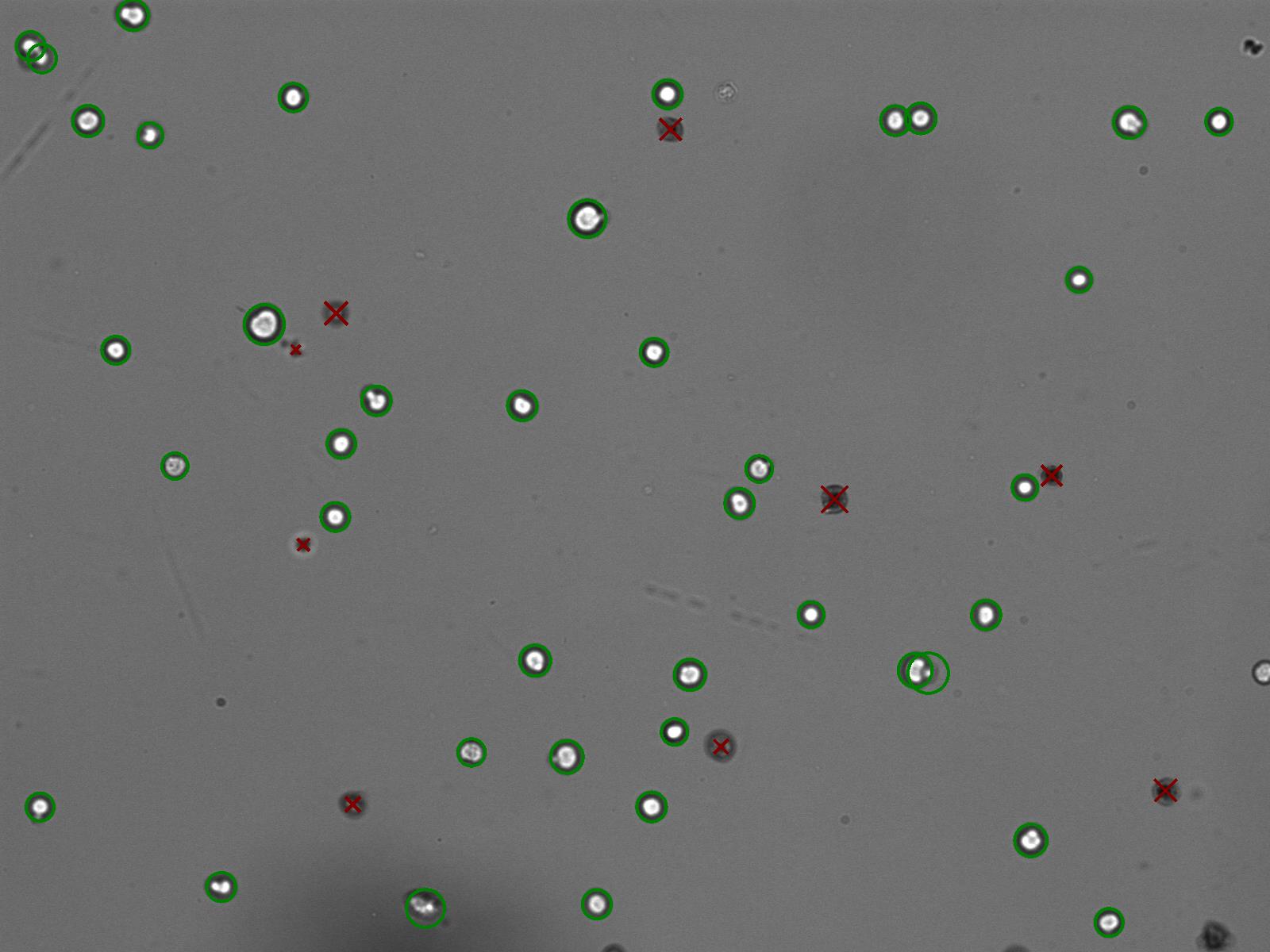

Supplement: Supplementary file 1 — Supplementary Information 1. [file 41598_2020_80576_MOESM1_ESM.zip › S1/Aggregate counts/day5/30mmHg Dec18 54 44/ML P2-036_2019-02-19_124646.bmp]

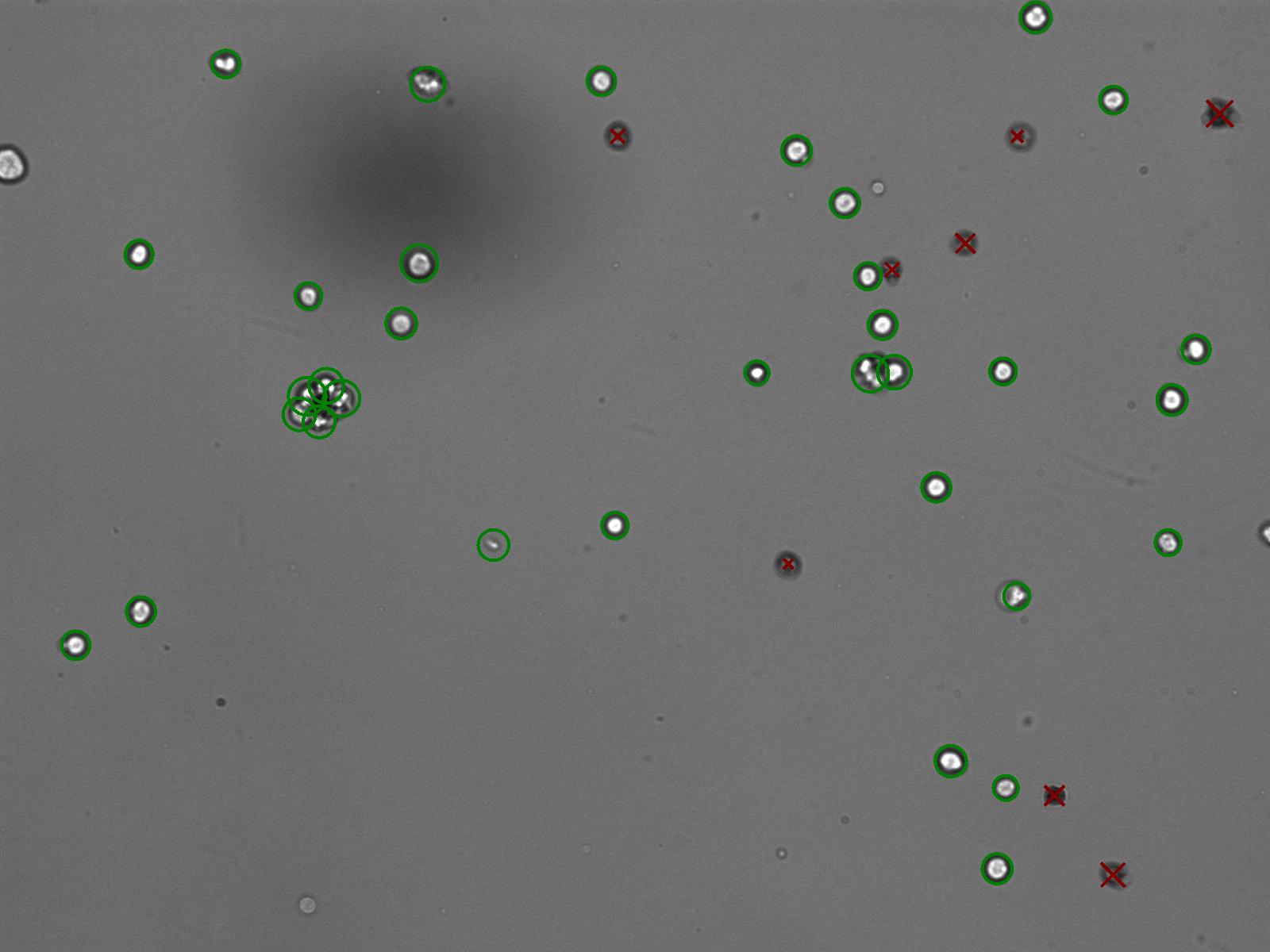

Supplement: Supplementary file 1 — Supplementary Information 1. [file 41598_2020_80576_MOESM1_ESM.zip › S1/Aggregate counts/day5/30mmHg Dec18 54 44/ML P2-037_2019-02-19_124646.bmp]

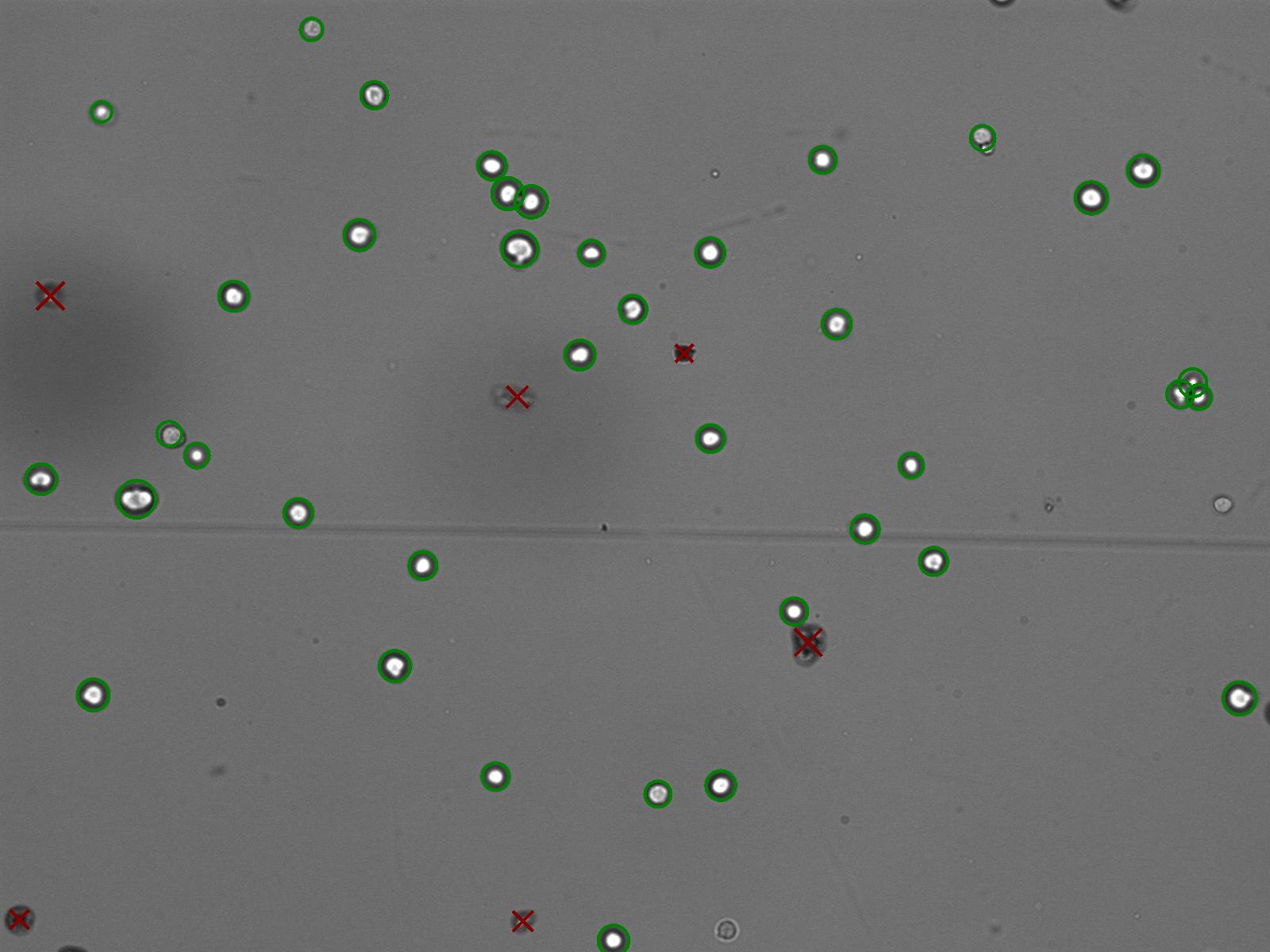

Supplement: Supplementary file 1 — Supplementary Information 1. [file 41598_2020_80576_MOESM1_ESM.zip › S1/Aggregate counts/day5/30mmHg Dec18 54 44/ML P2-038_2019-02-19_124646.bmp]

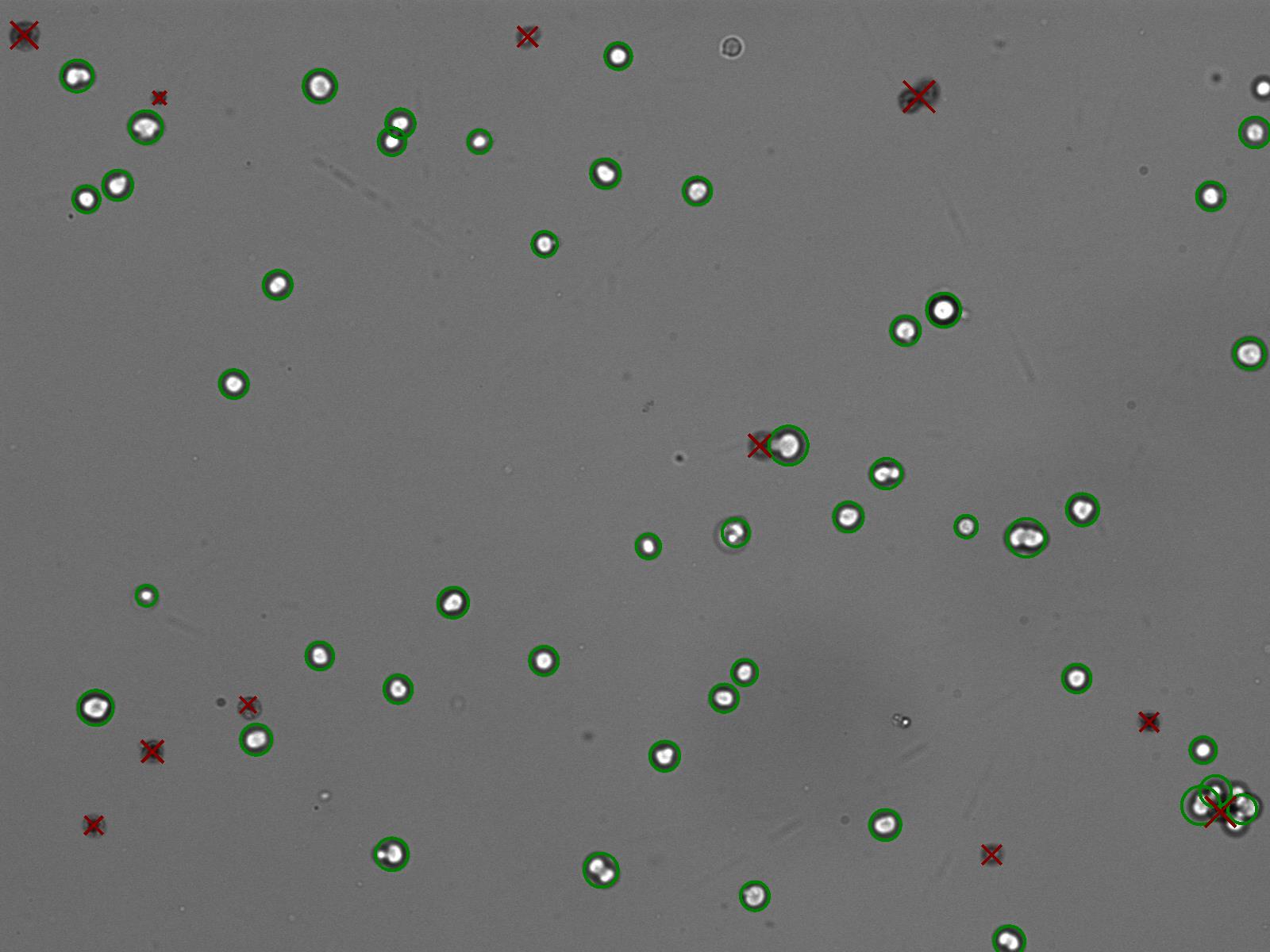

Supplement: Supplementary file 1 — Supplementary Information 1. [file 41598_2020_80576_MOESM1_ESM.zip › S1/Aggregate counts/day5/30mmHg Dec18 54 44/ML P2-039_2019-02-19_124647.bmp]

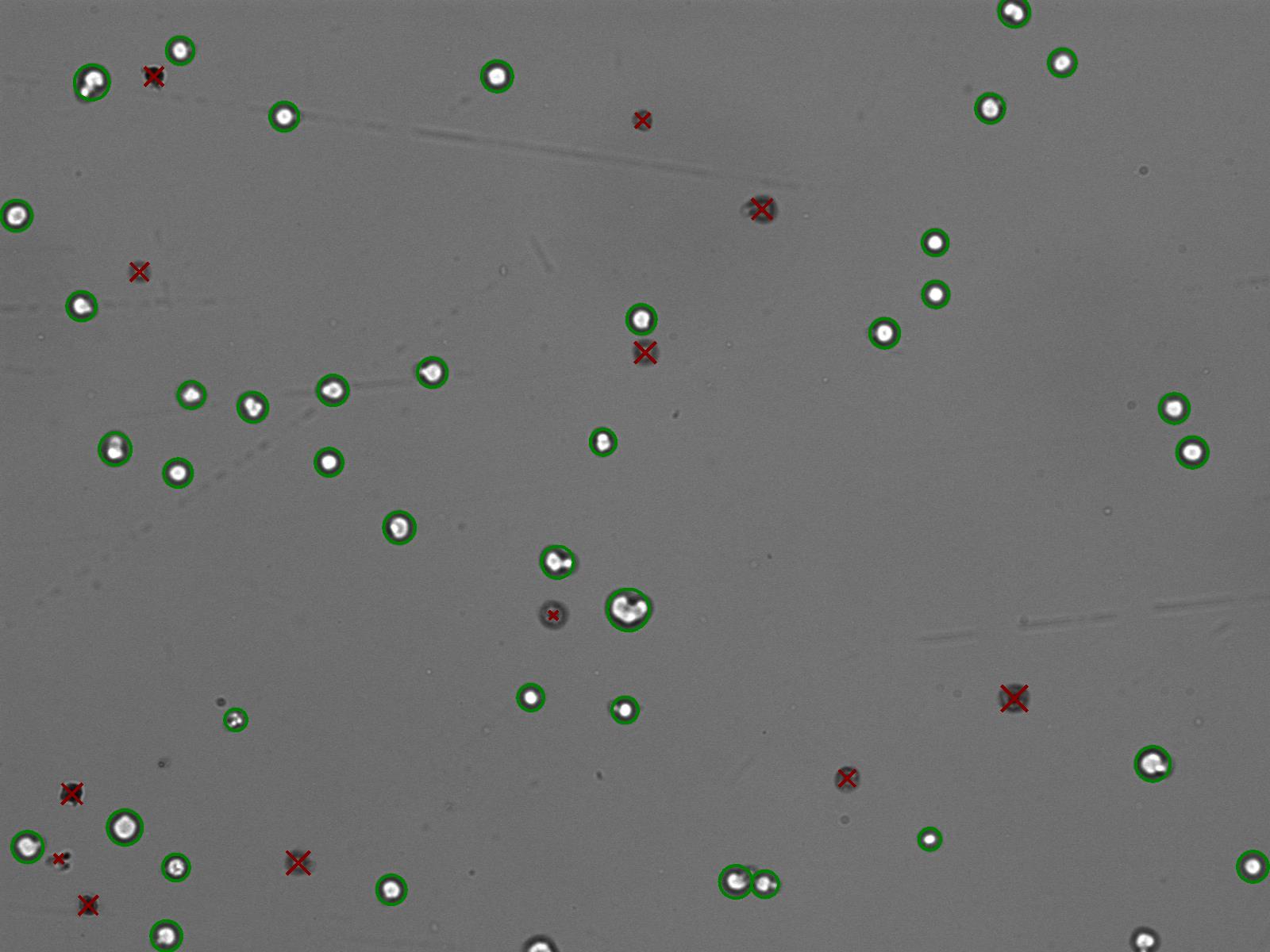

Supplement: Supplementary file 1 — Supplementary Information 1. [file 41598_2020_80576_MOESM1_ESM.zip › S1/Aggregate counts/day5/30mmHg Dec18 54 44/ML P2-040_2019-02-19_124647.bmp]

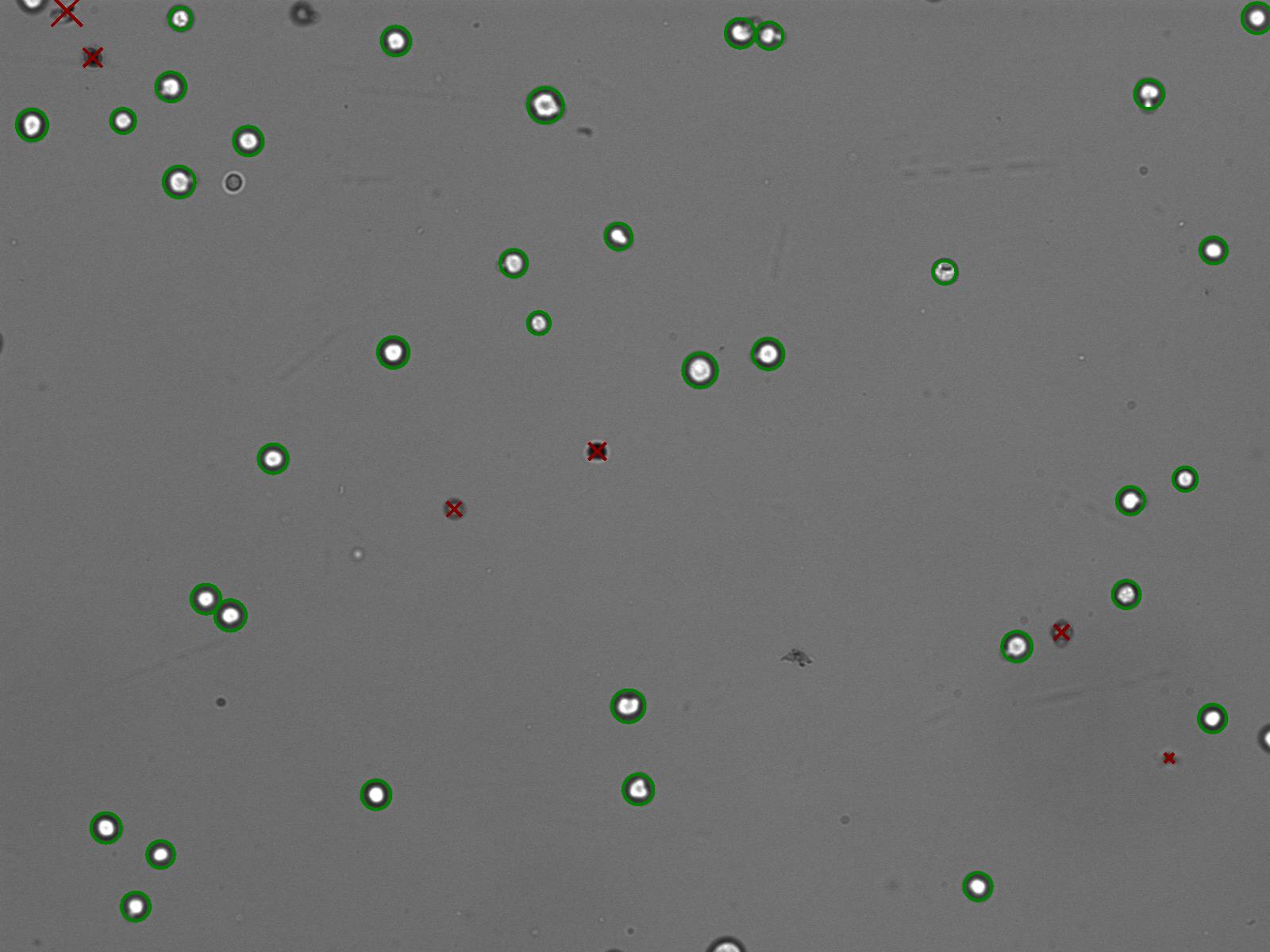

Supplement: Supplementary file 1 — Supplementary Information 1. [file 41598_2020_80576_MOESM1_ESM.zip › S1/Aggregate counts/day5/30mmHg Dec18 54 44/ML P2-041_2019-02-19_124647.bmp]

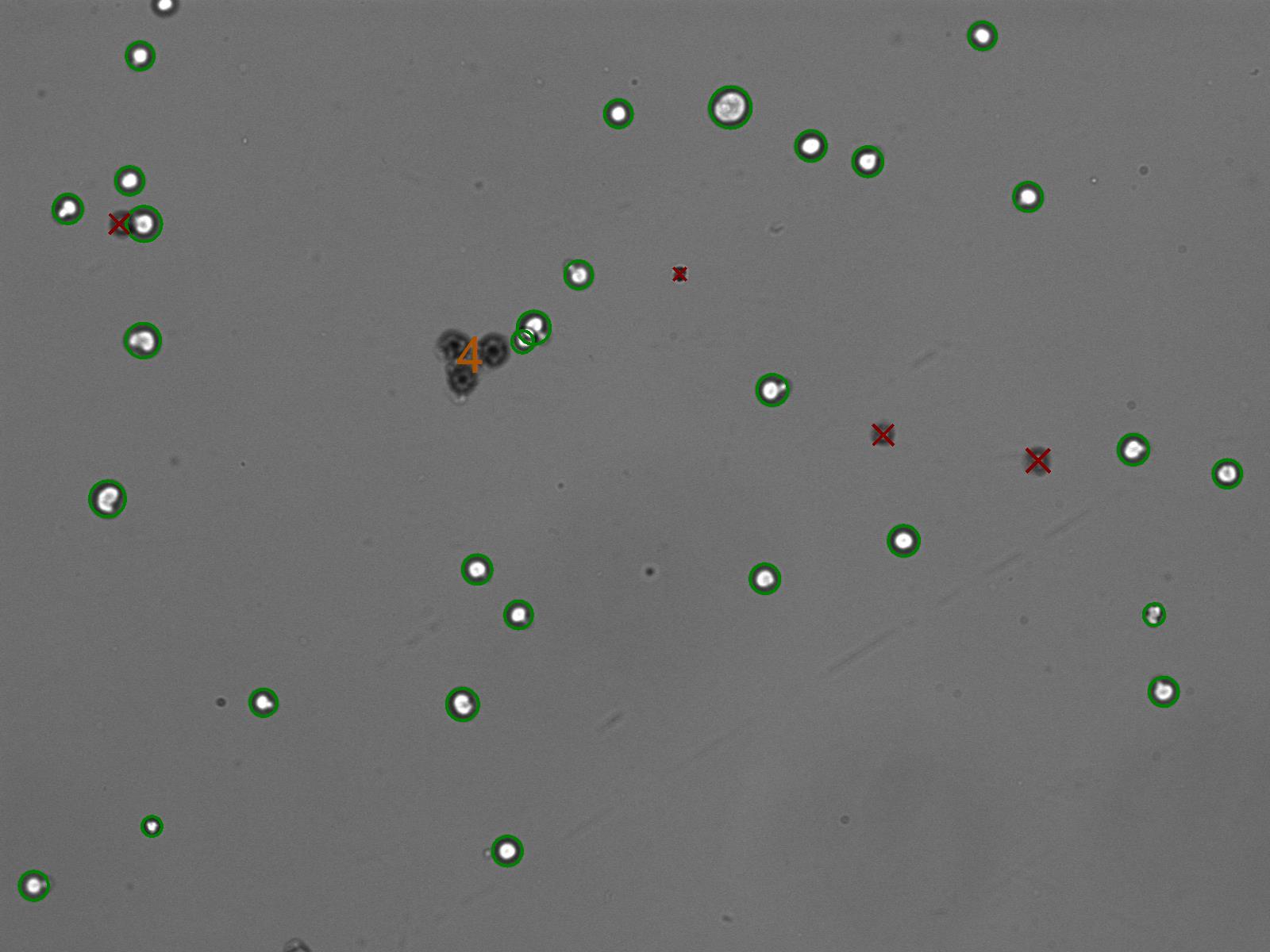

Supplement: Supplementary file 1 — Supplementary Information 1. [file 41598_2020_80576_MOESM1_ESM.zip › S1/Aggregate counts/day5/30mmHg Dec18 54 44/ML P2-042_2019-02-19_124648.bmp]

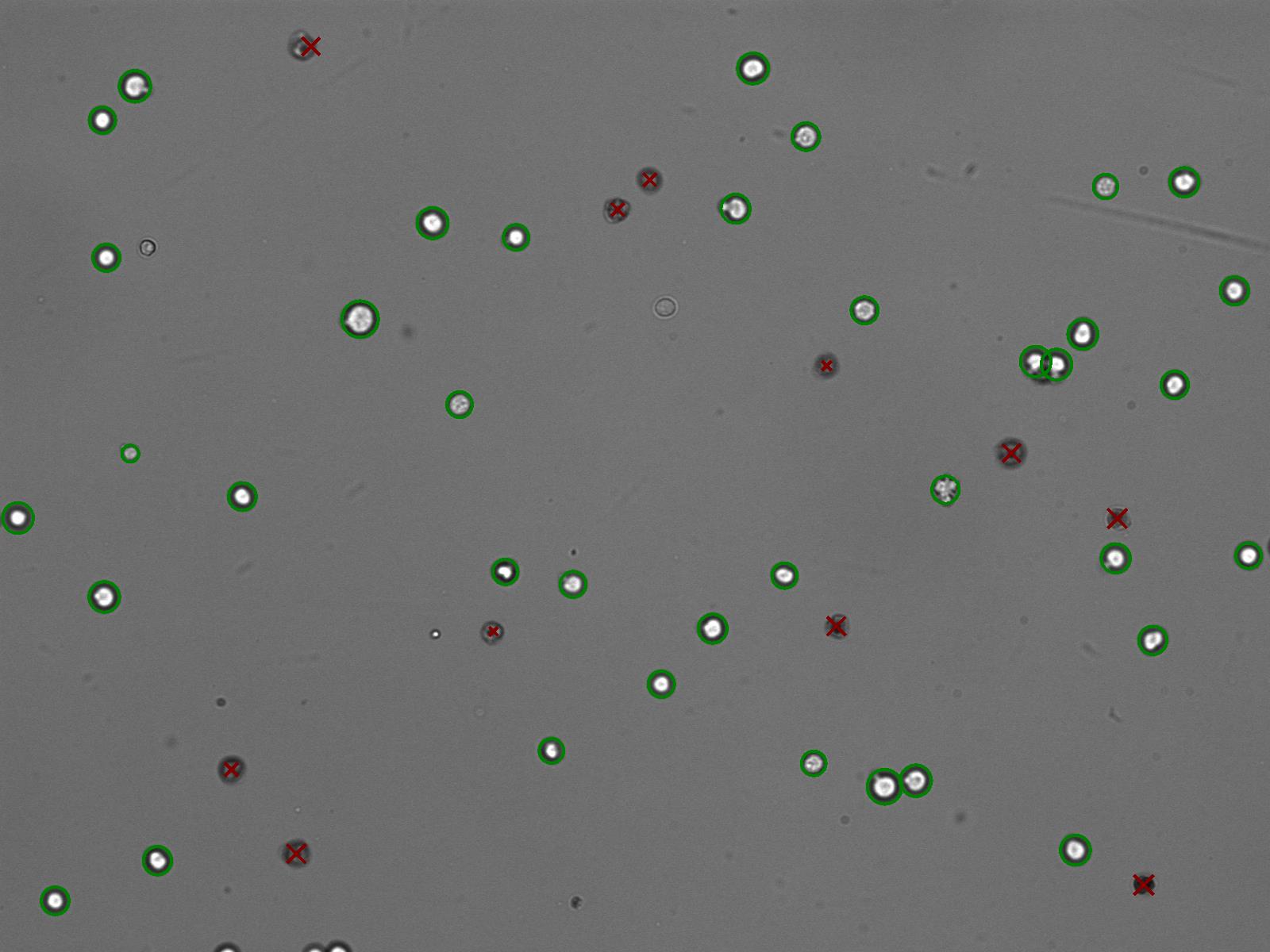

Supplement: Supplementary file 1 — Supplementary Information 1. [file 41598_2020_80576_MOESM1_ESM.zip › S1/Aggregate counts/day5/30mmHg Dec18 54 44/ML P2-043_2019-02-19_124648.bmp]

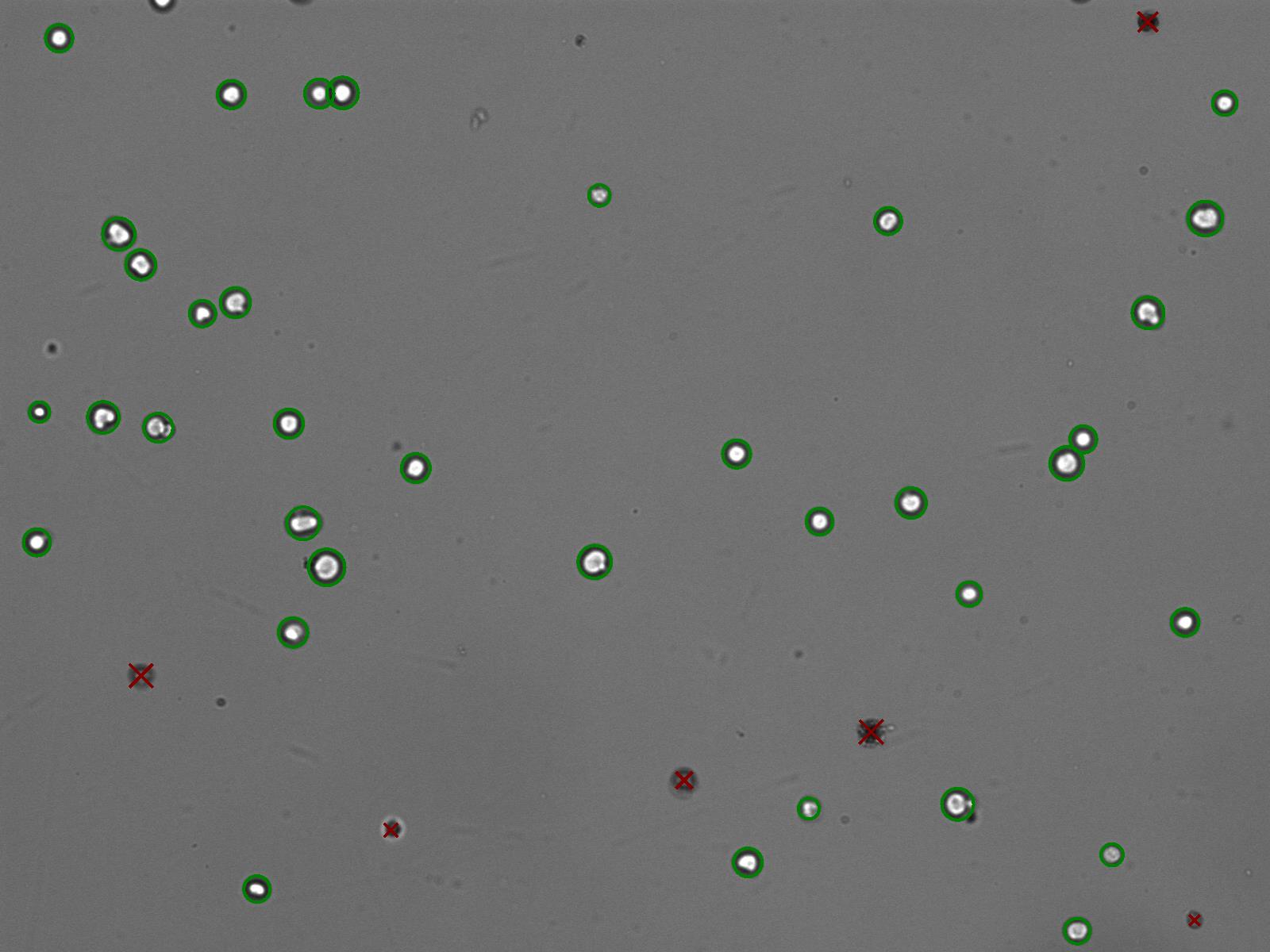

Supplement: Supplementary file 1 — Supplementary Information 1. [file 41598_2020_80576_MOESM1_ESM.zip › S1/Aggregate counts/day5/30mmHg Dec18 54 44/ML P2-044_2019-02-19_124648.bmp]

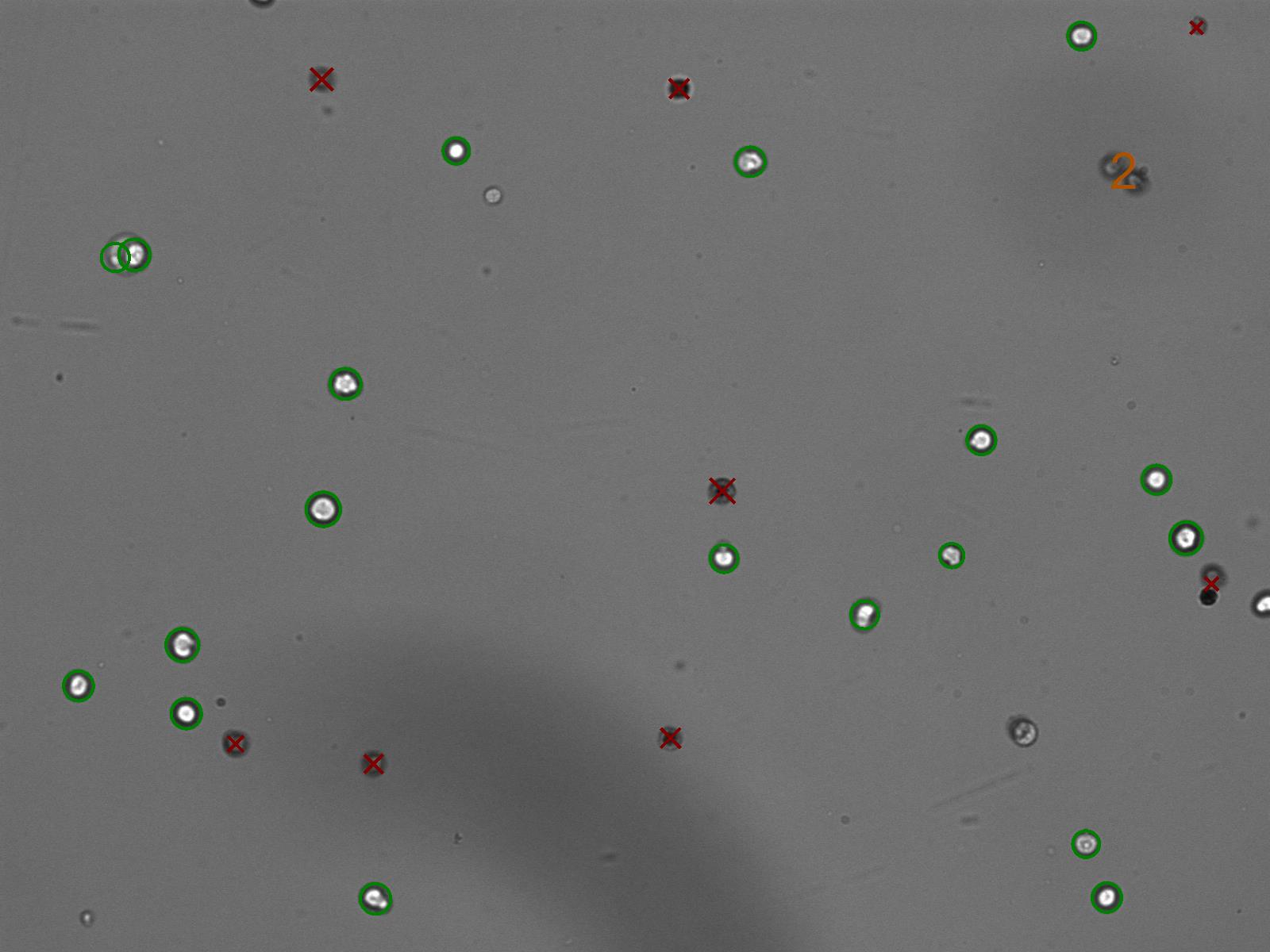

Supplement: Supplementary file 1 — Supplementary Information 1. [file 41598_2020_80576_MOESM1_ESM.zip › S1/Aggregate counts/day5/30mmHg Dec18 54 44/ML P2-045_2019-02-19_124649.bmp]

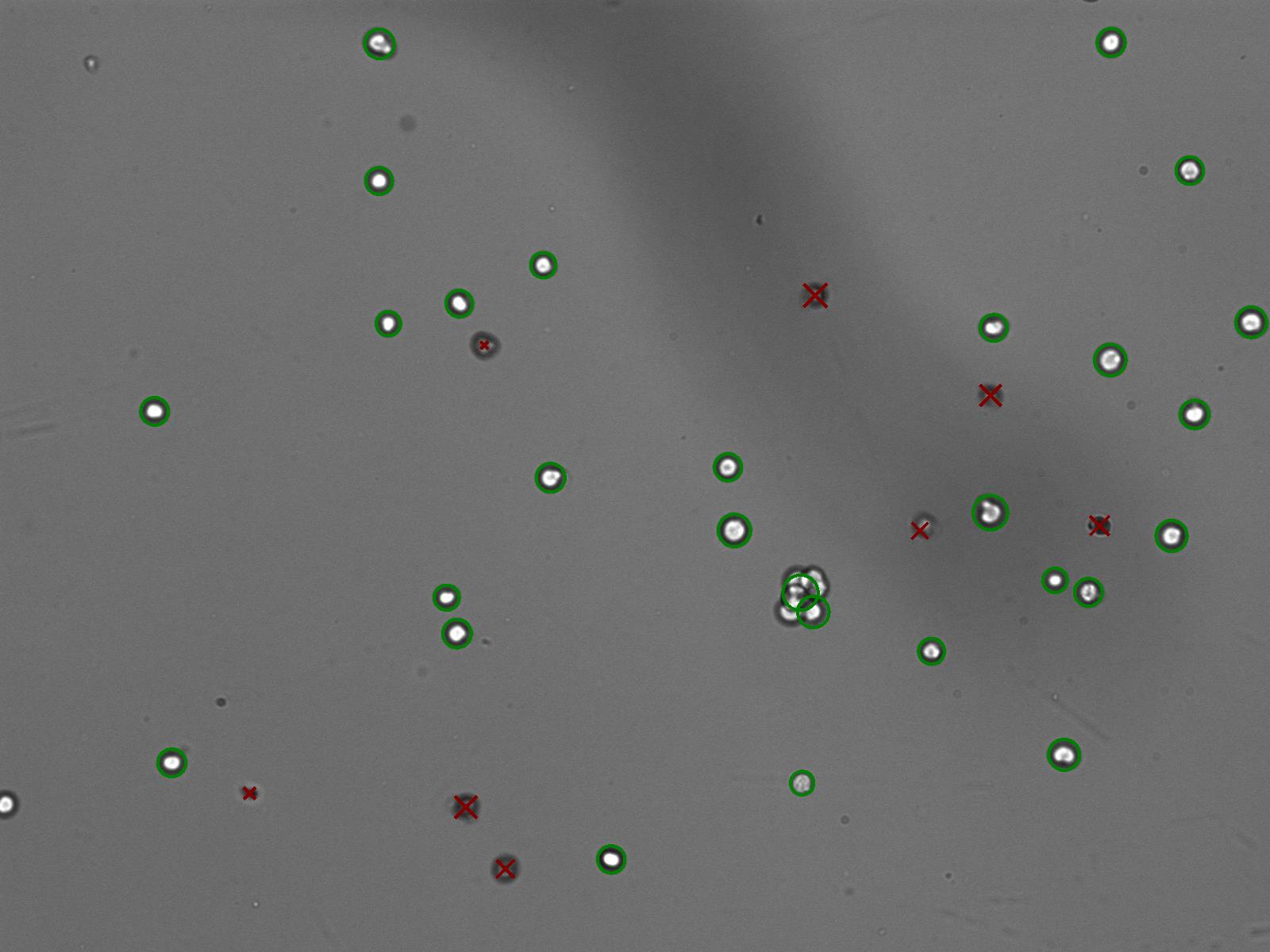

Supplement: Supplementary file 1 — Supplementary Information 1. [file 41598_2020_80576_MOESM1_ESM.zip › S1/Aggregate counts/day5/30mmHg Dec18 54 44/ML P2-046_2019-02-19_124649.bmp]

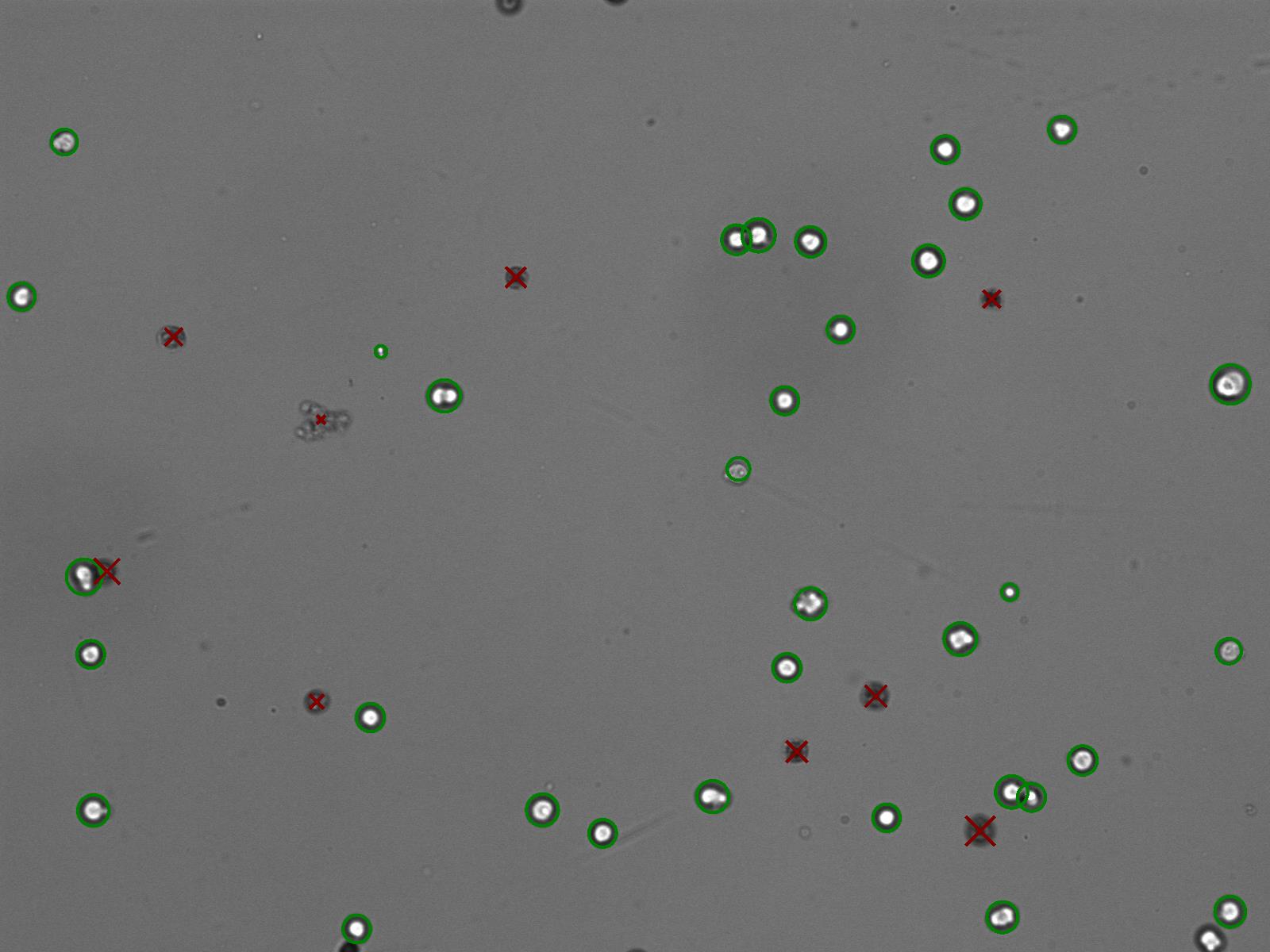

Supplement: Supplementary file 1 — Supplementary Information 1. [file 41598_2020_80576_MOESM1_ESM.zip › S1/Aggregate counts/day5/30mmHg Dec18 54 44/ML P2-047_2019-02-19_124649.bmp]

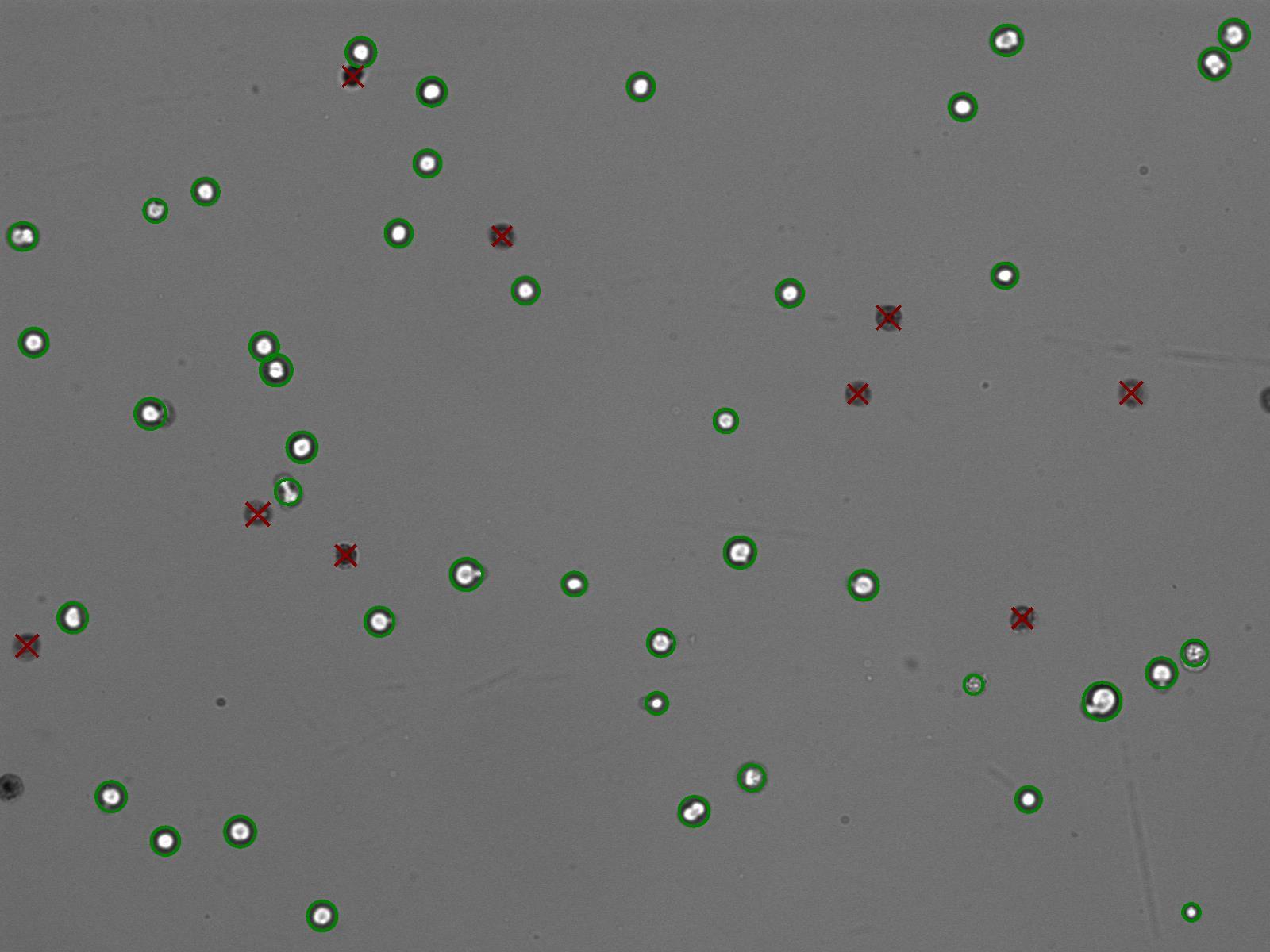

Supplement: Supplementary file 1 — Supplementary Information 1. [file 41598_2020_80576_MOESM1_ESM.zip › S1/Aggregate counts/day5/30mmHg Dec18 54 44/ML P2-048_2019-02-19_124650.bmp]

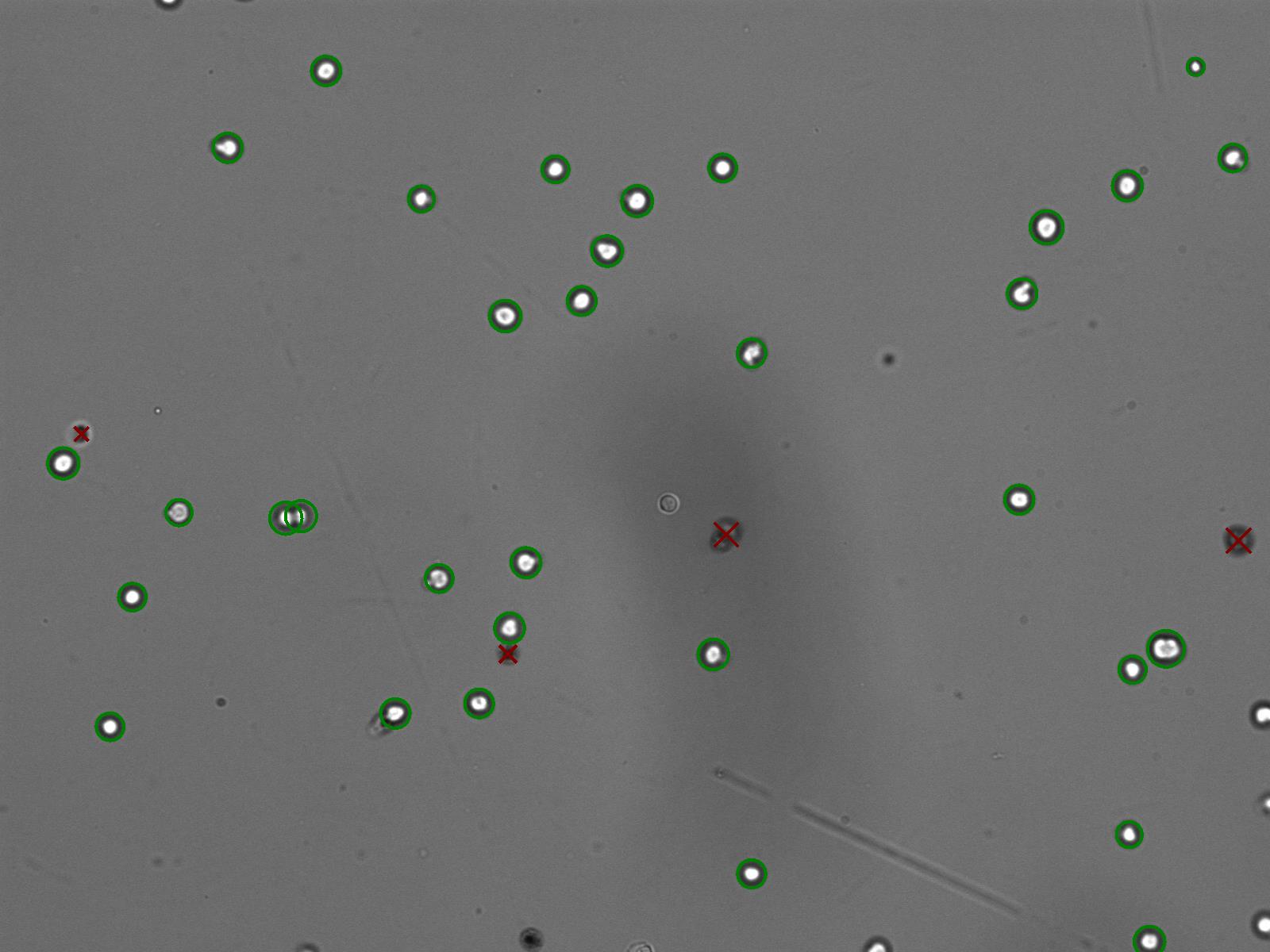

Supplement: Supplementary file 1 — Supplementary Information 1. [file 41598_2020_80576_MOESM1_ESM.zip › S1/Aggregate counts/day5/30mmHg Dec18 54 44/ML P2-049_2019-02-19_124650.bmp]

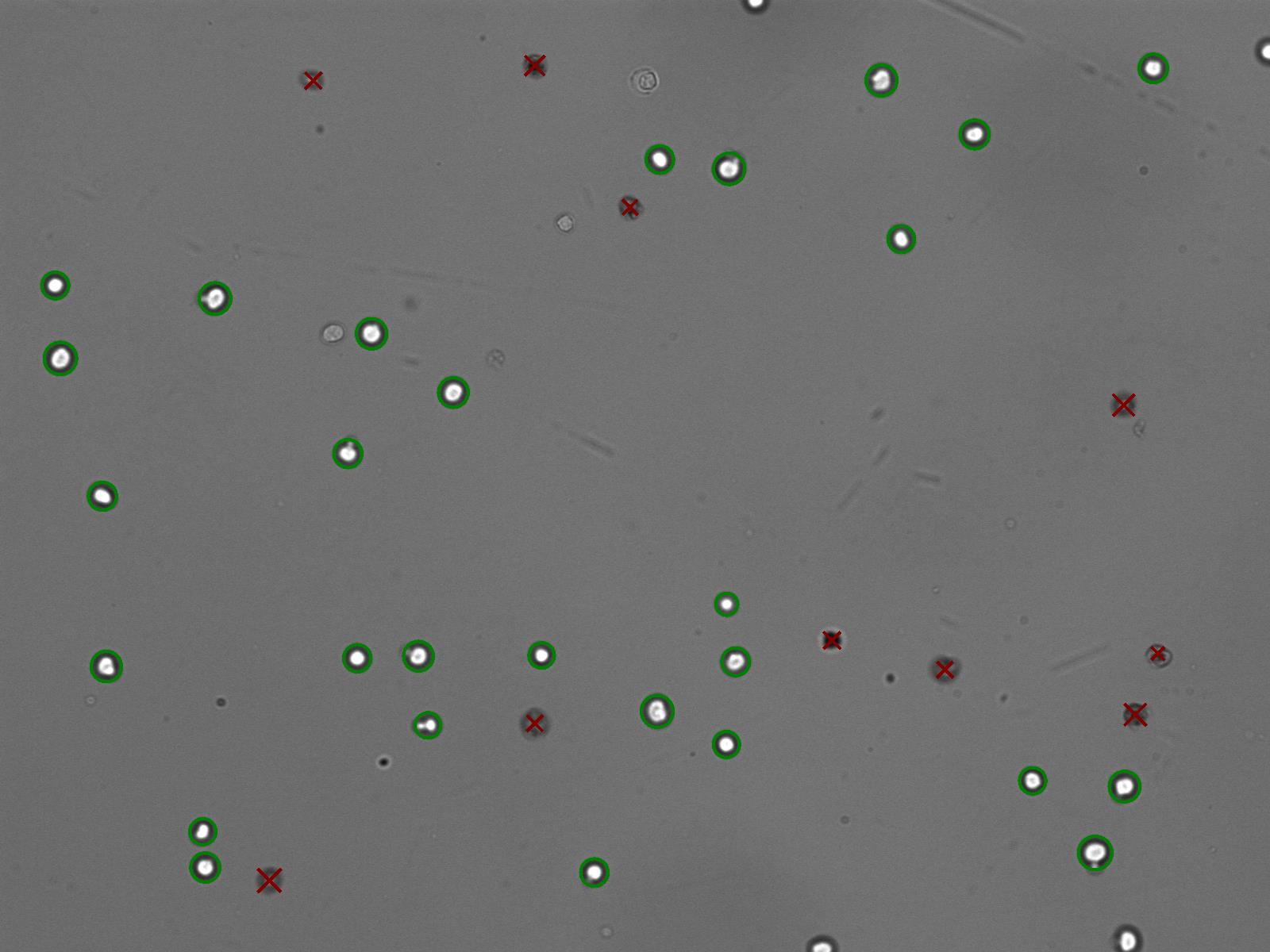

Supplement: Supplementary file 1 — Supplementary Information 1. [file 41598_2020_80576_MOESM1_ESM.zip › S1/Aggregate counts/day5/30mmHg Dec18 54 44/ML P2-050_2019-02-19_124651.bmp]

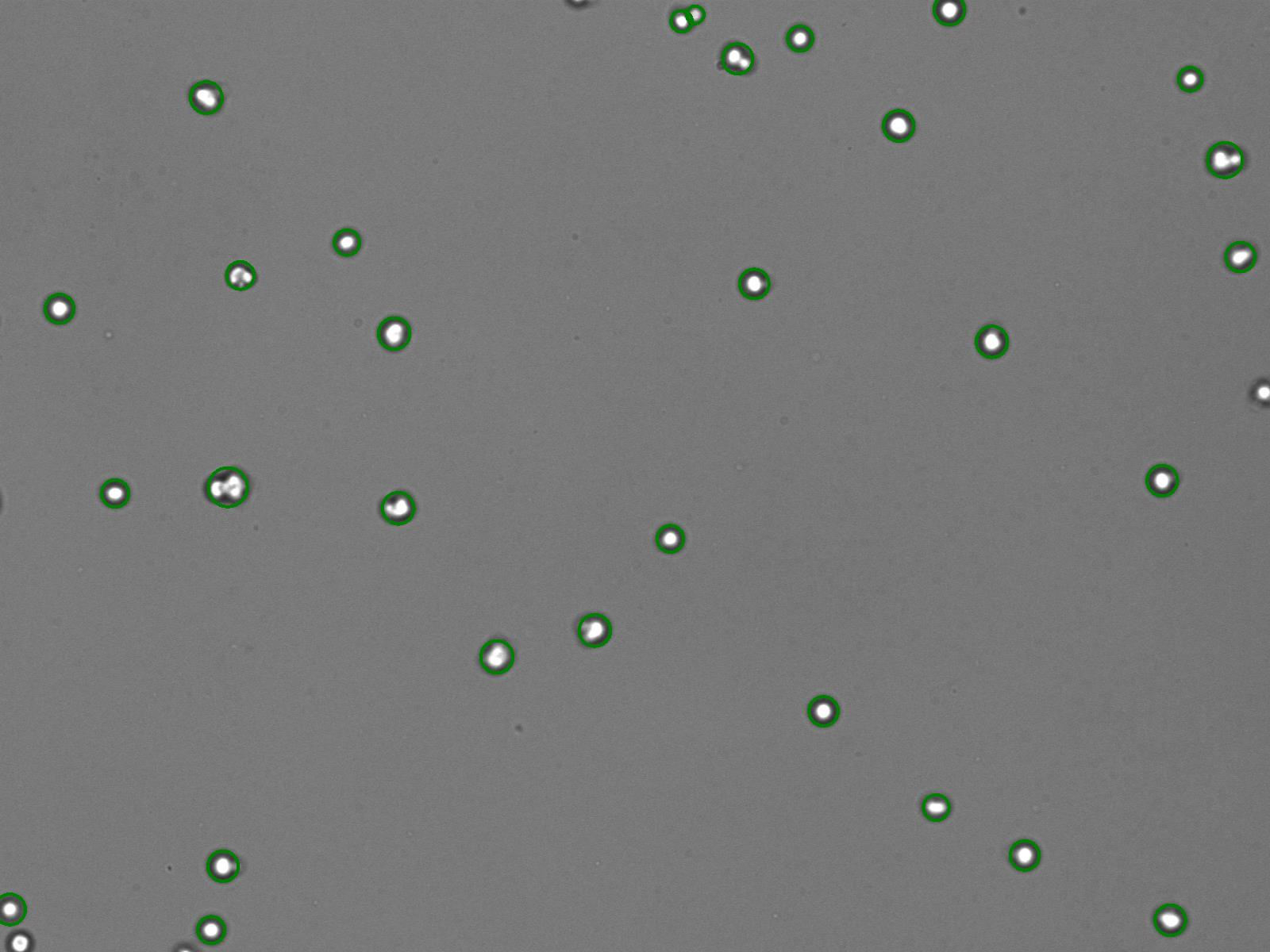

Supplement: Supplementary file 1 — Supplementary Information 1. [file 41598_2020_80576_MOESM1_ESM.zip › S1/Aggregate counts/day5/30mmHg Jan18 47 43/ML P3-001_2019-02-11_151843.bmp]

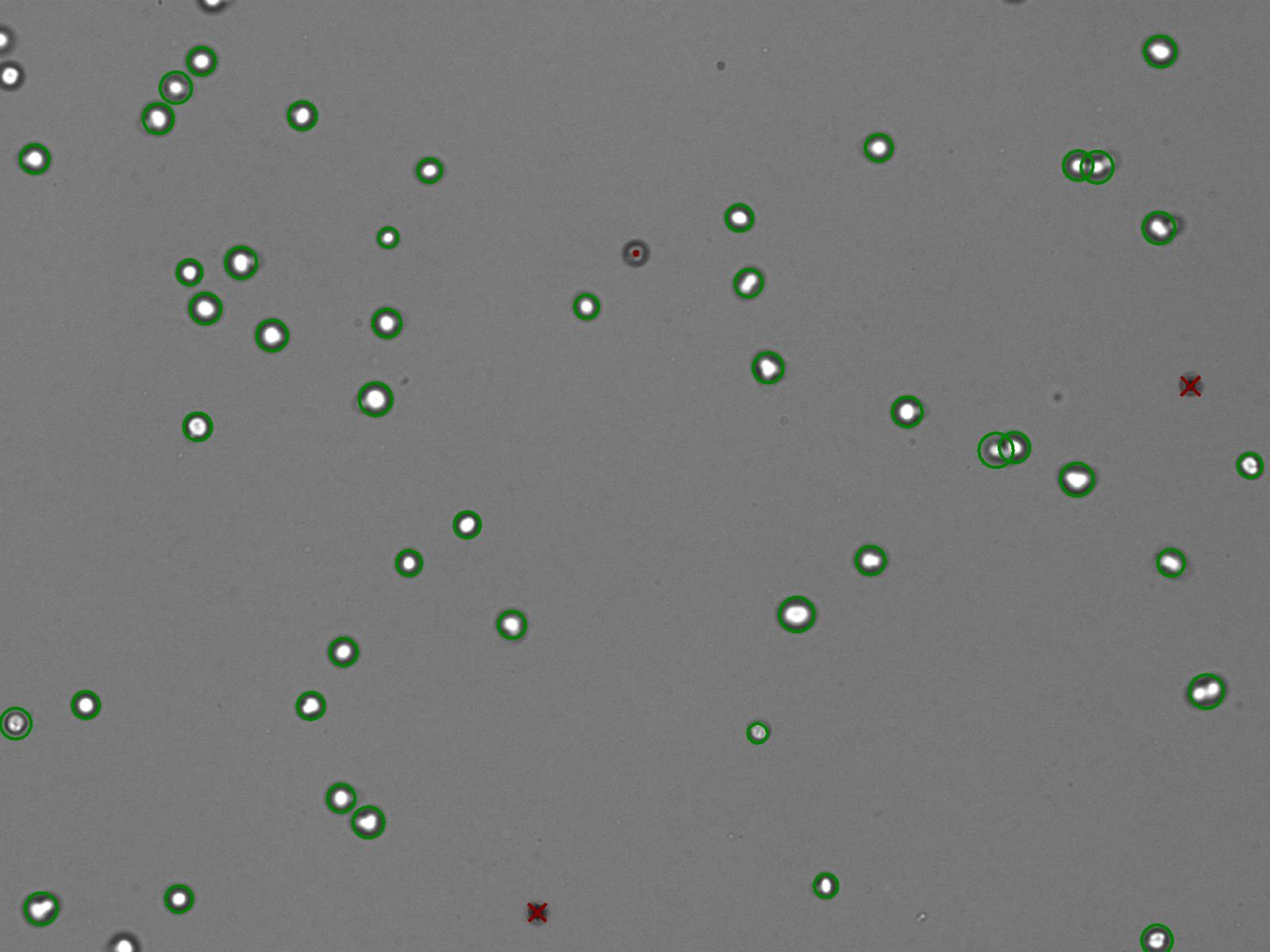

Supplement: Supplementary file 1 — Supplementary Information 1. [file 41598_2020_80576_MOESM1_ESM.zip › S1/Aggregate counts/day5/30mmHg Jan18 47 43/ML P3-002_2019-02-11_151844.bmp]

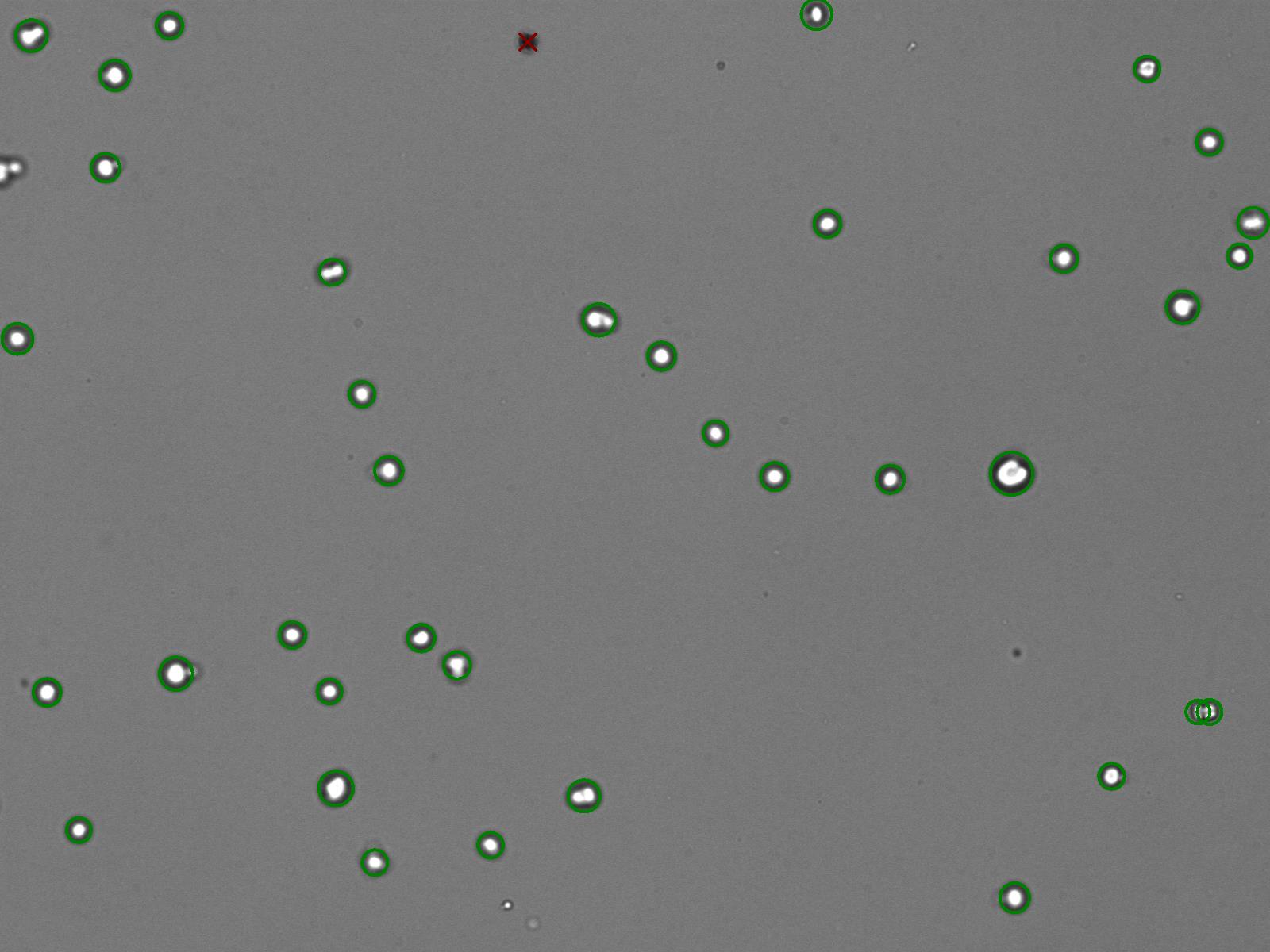

Supplement: Supplementary file 1 — Supplementary Information 1. [file 41598_2020_80576_MOESM1_ESM.zip › S1/Aggregate counts/day5/30mmHg Jan18 47 43/ML P3-003_2019-02-11_151844.bmp]

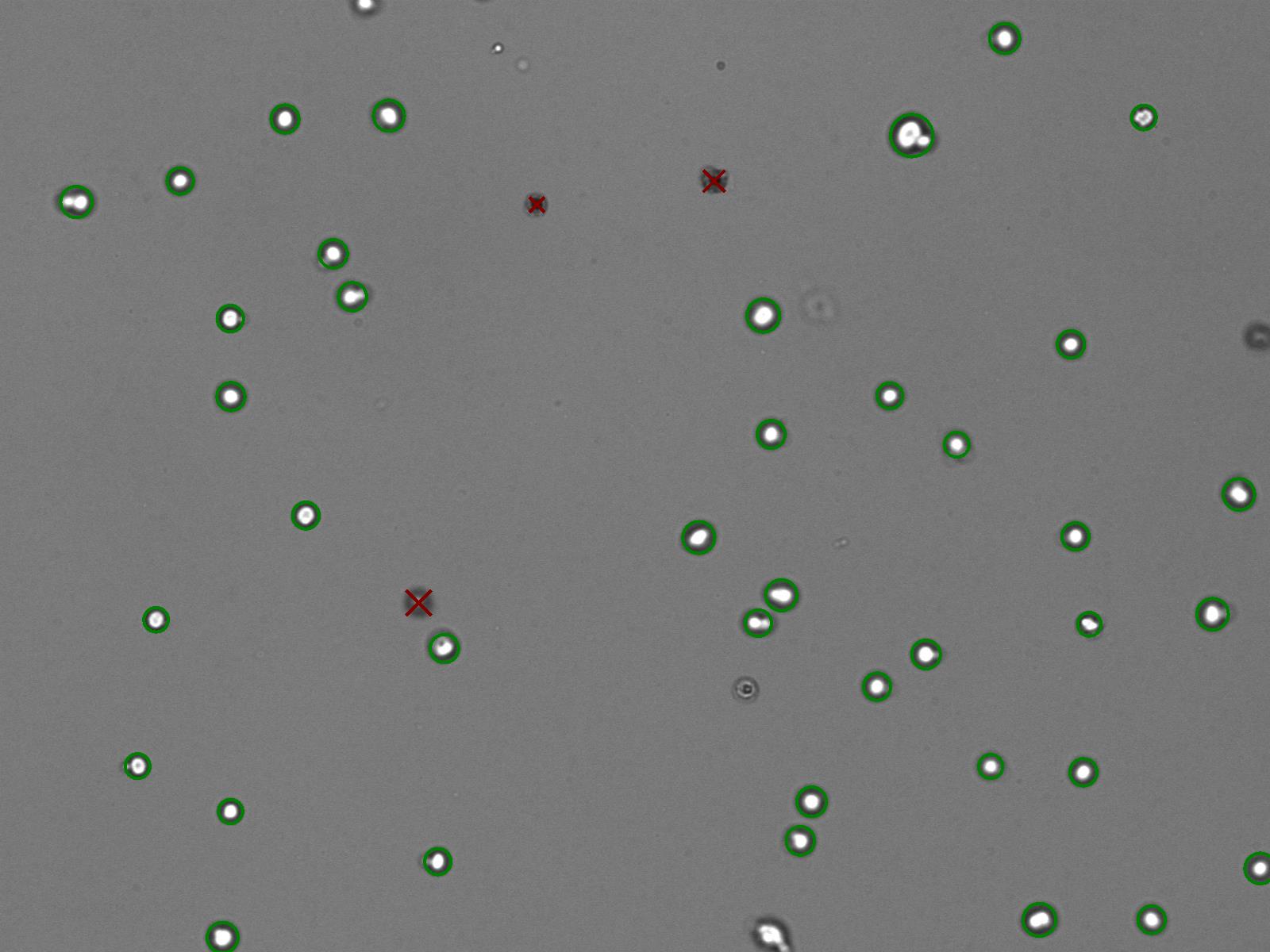

Supplement: Supplementary file 1 — Supplementary Information 1. [file 41598_2020_80576_MOESM1_ESM.zip › S1/Aggregate counts/day5/30mmHg Jan18 47 43/ML P3-004_2019-02-11_151844.bmp]

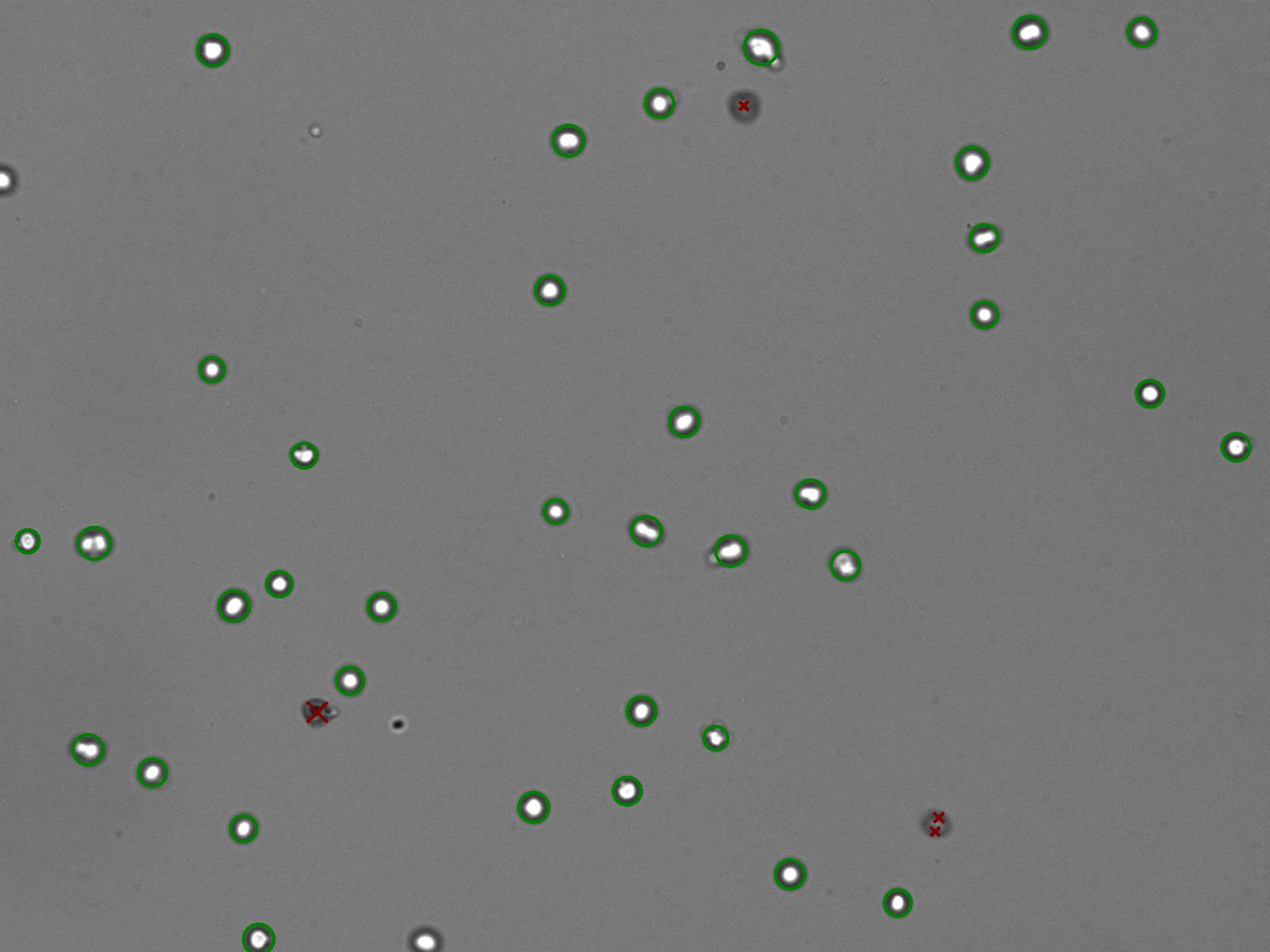

Supplement: Supplementary file 1 — Supplementary Information 1. [file 41598_2020_80576_MOESM1_ESM.zip › S1/Aggregate counts/day5/30mmHg Jan18 47 43/ML P3-005_2019-02-11_151845.bmp]

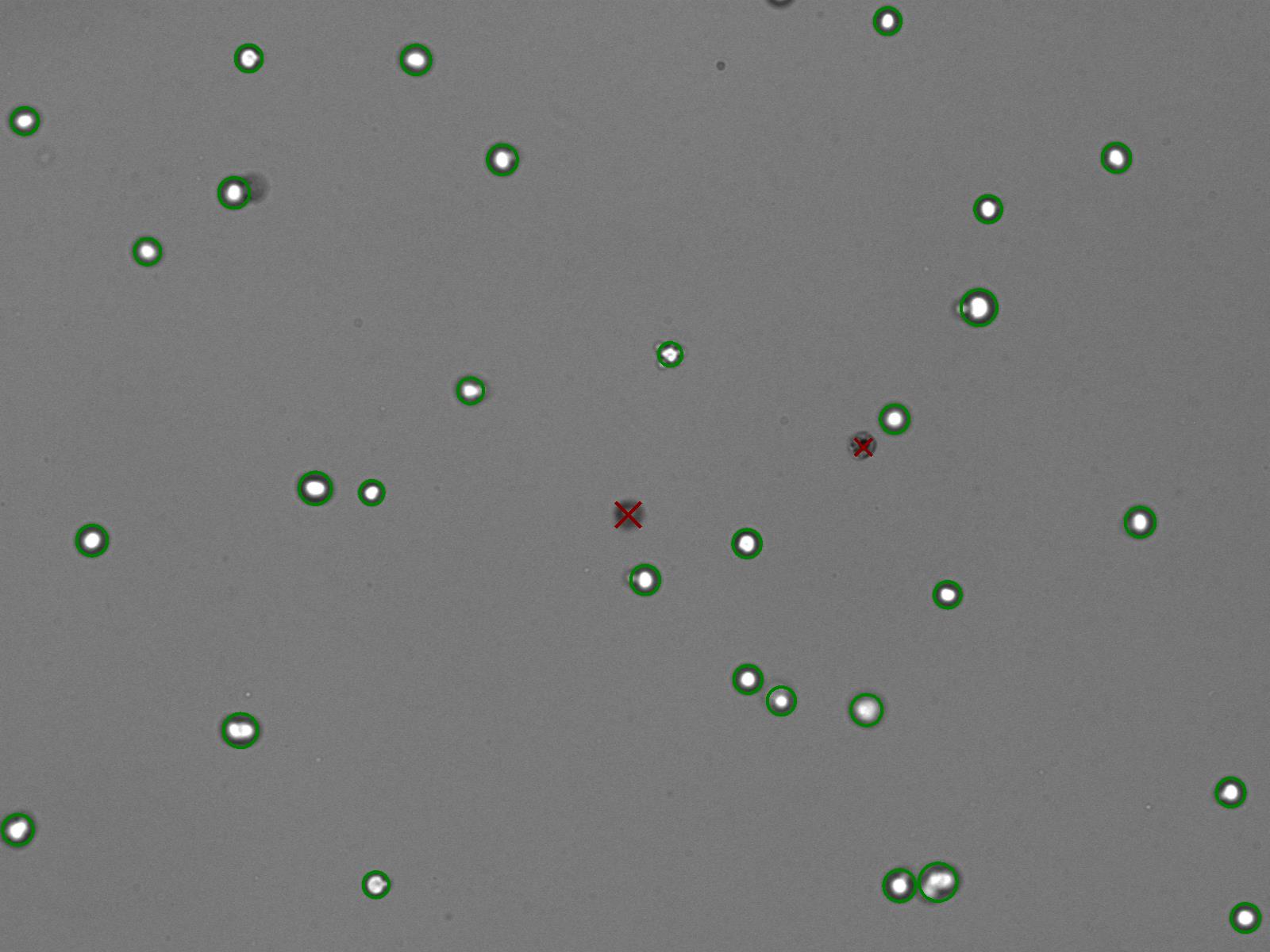

Supplement: Supplementary file 1 — Supplementary Information 1. [file 41598_2020_80576_MOESM1_ESM.zip › S1/Aggregate counts/day5/30mmHg Jan18 47 43/ML P3-006_2019-02-11_151845.bmp]

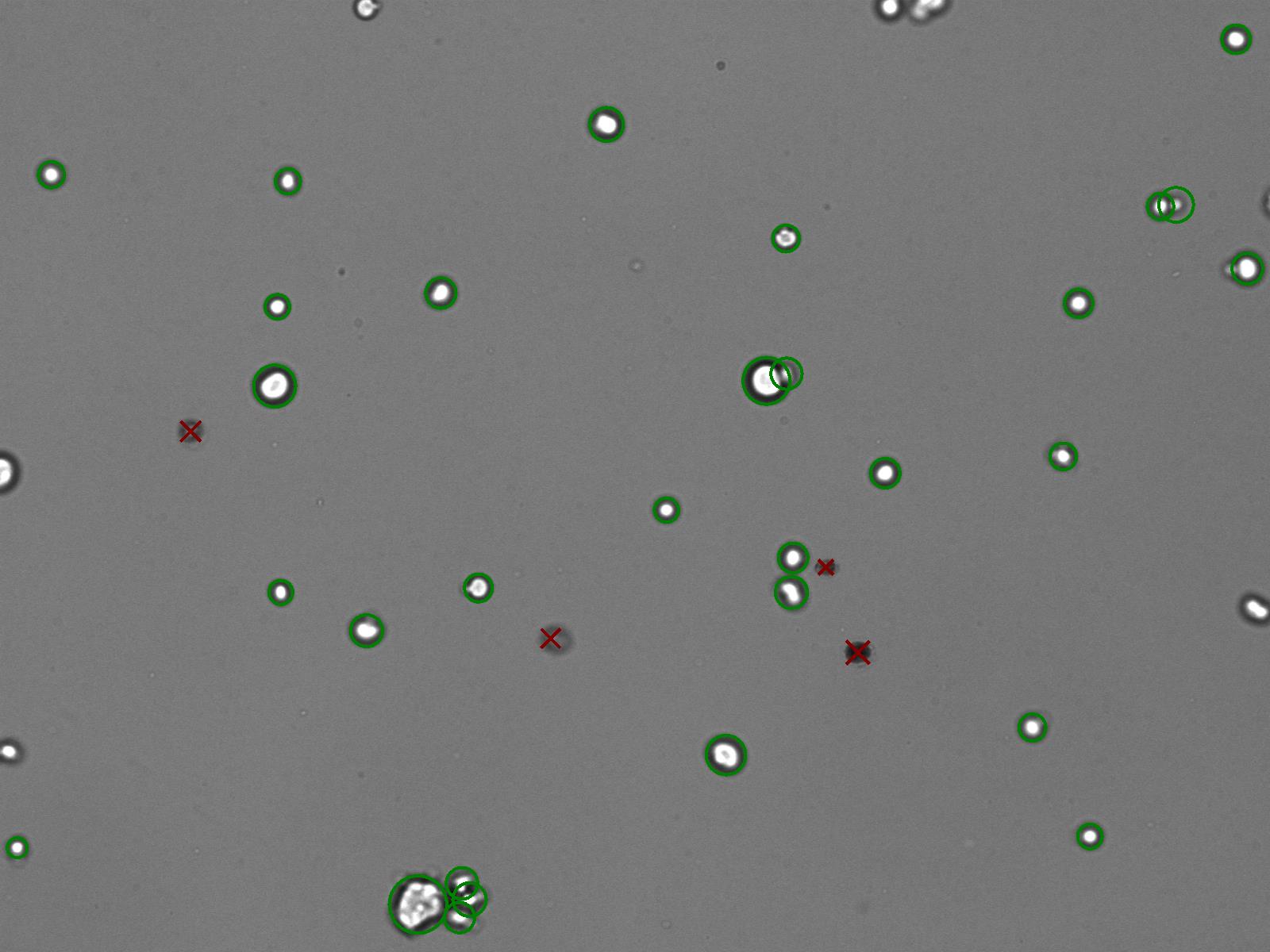

Supplement: Supplementary file 1 — Supplementary Information 1. [file 41598_2020_80576_MOESM1_ESM.zip › S1/Aggregate counts/day5/30mmHg Jan18 47 43/ML P3-007_2019-02-11_151845.bmp]

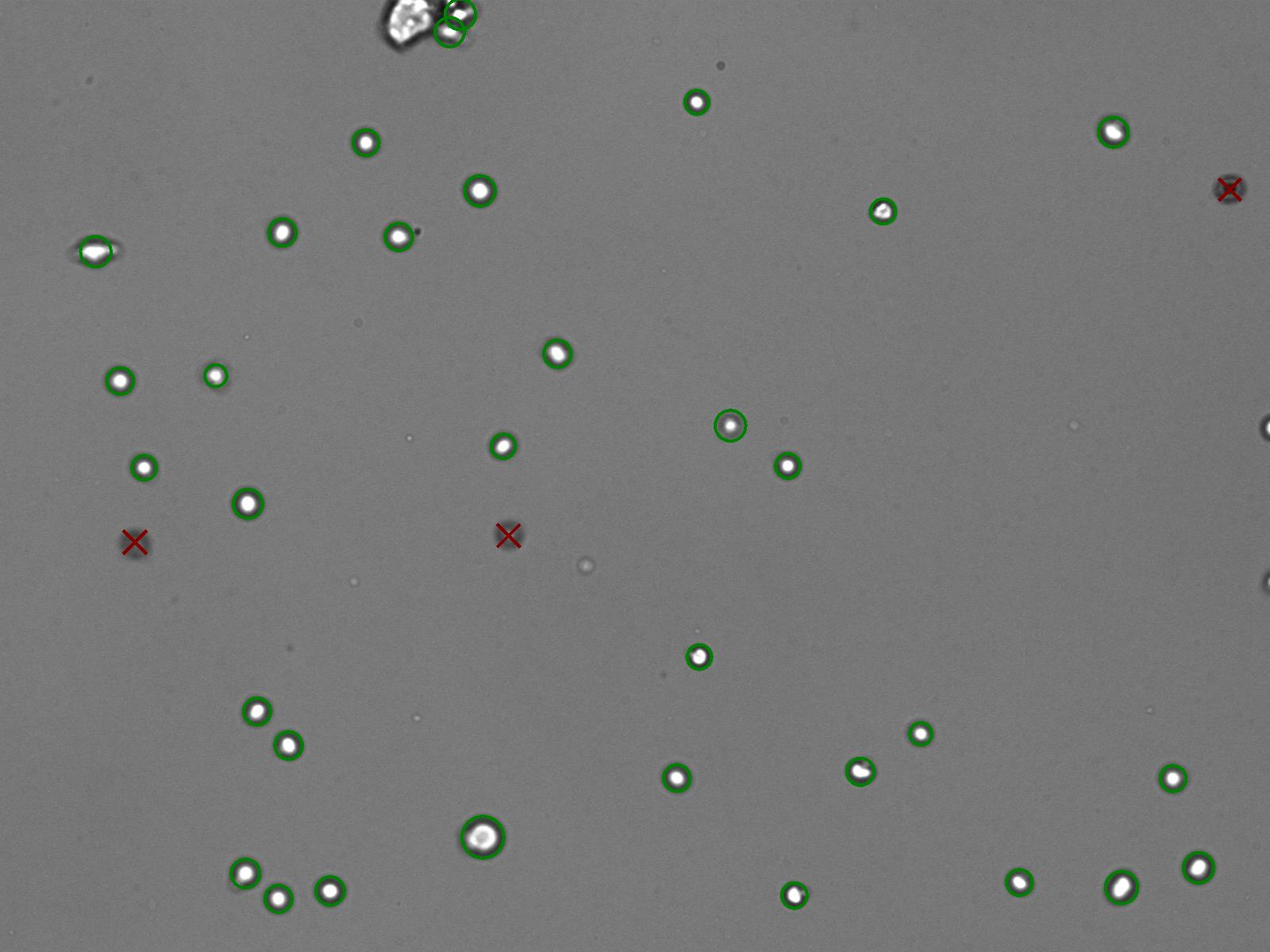

Supplement: Supplementary file 1 — Supplementary Information 1. [file 41598_2020_80576_MOESM1_ESM.zip › S1/Aggregate counts/day5/30mmHg Jan18 47 43/ML P3-008_2019-02-11_151846.bmp]

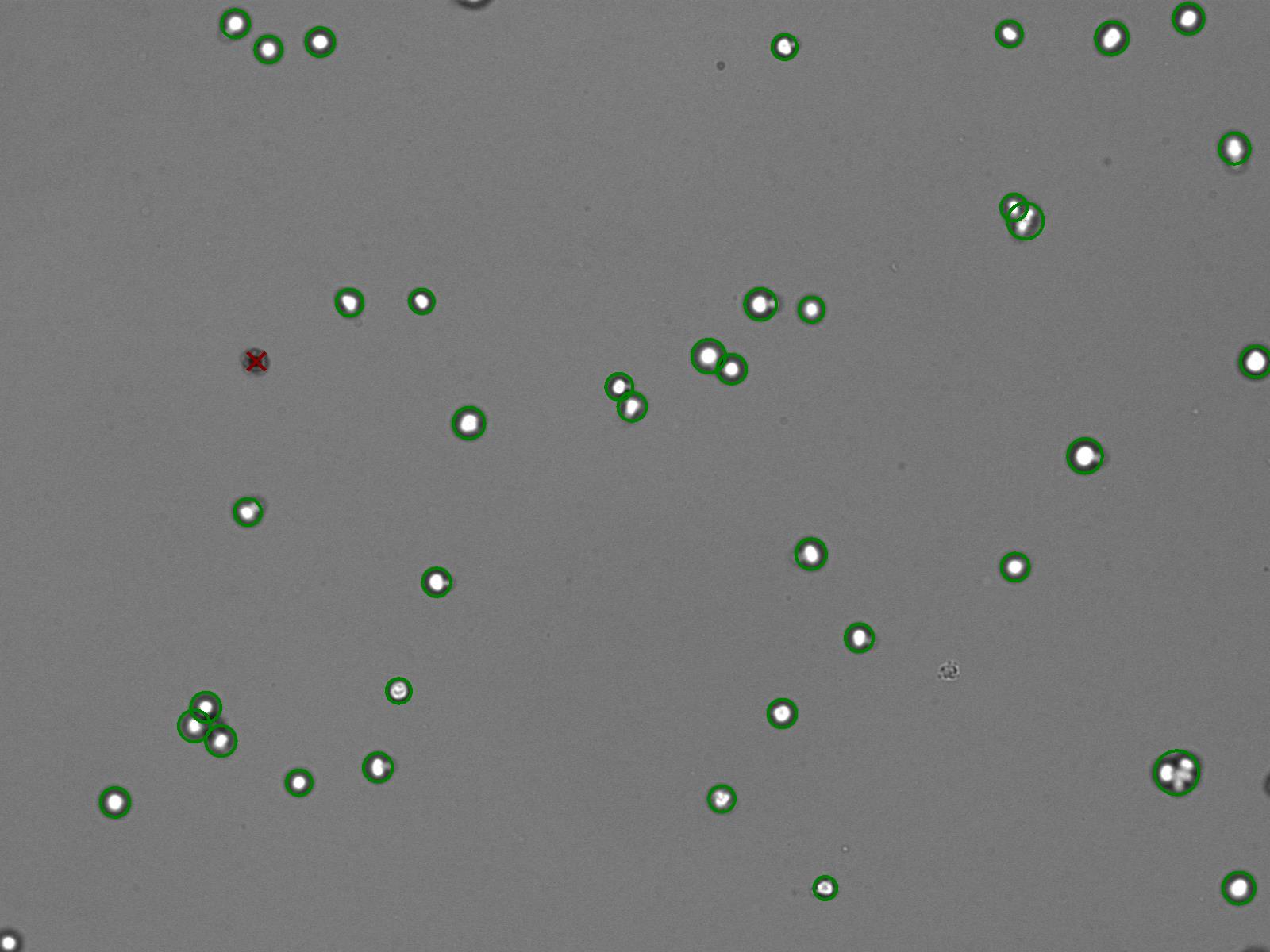

Supplement: Supplementary file 1 — Supplementary Information 1. [file 41598_2020_80576_MOESM1_ESM.zip › S1/Aggregate counts/day5/30mmHg Jan18 47 43/ML P3-009_2019-02-11_151846.bmp]

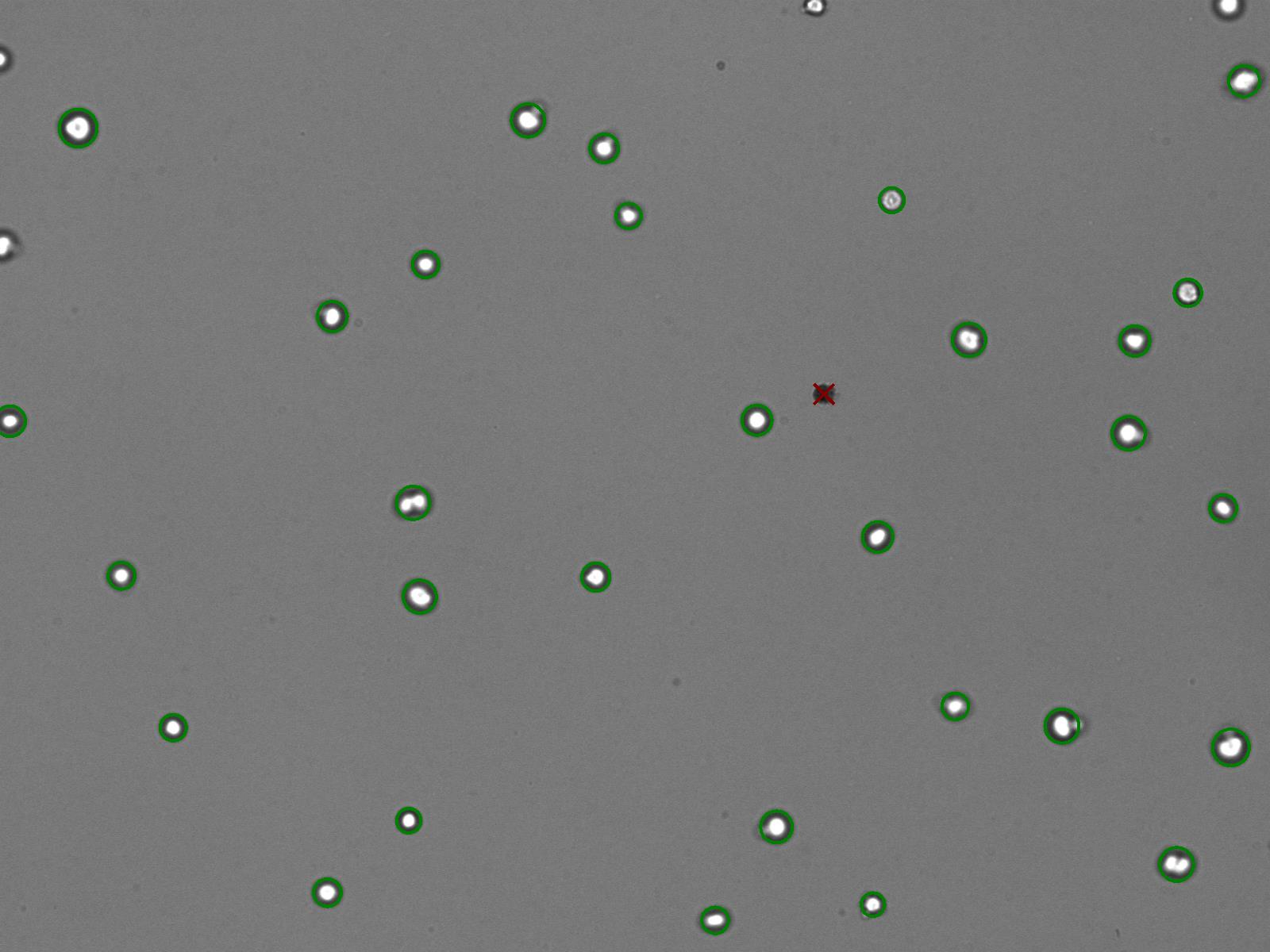

Supplement: Supplementary file 1 — Supplementary Information 1. [file 41598_2020_80576_MOESM1_ESM.zip › S1/Aggregate counts/day5/30mmHg Jan18 47 43/ML P3-010_2019-02-11_151846.bmp]

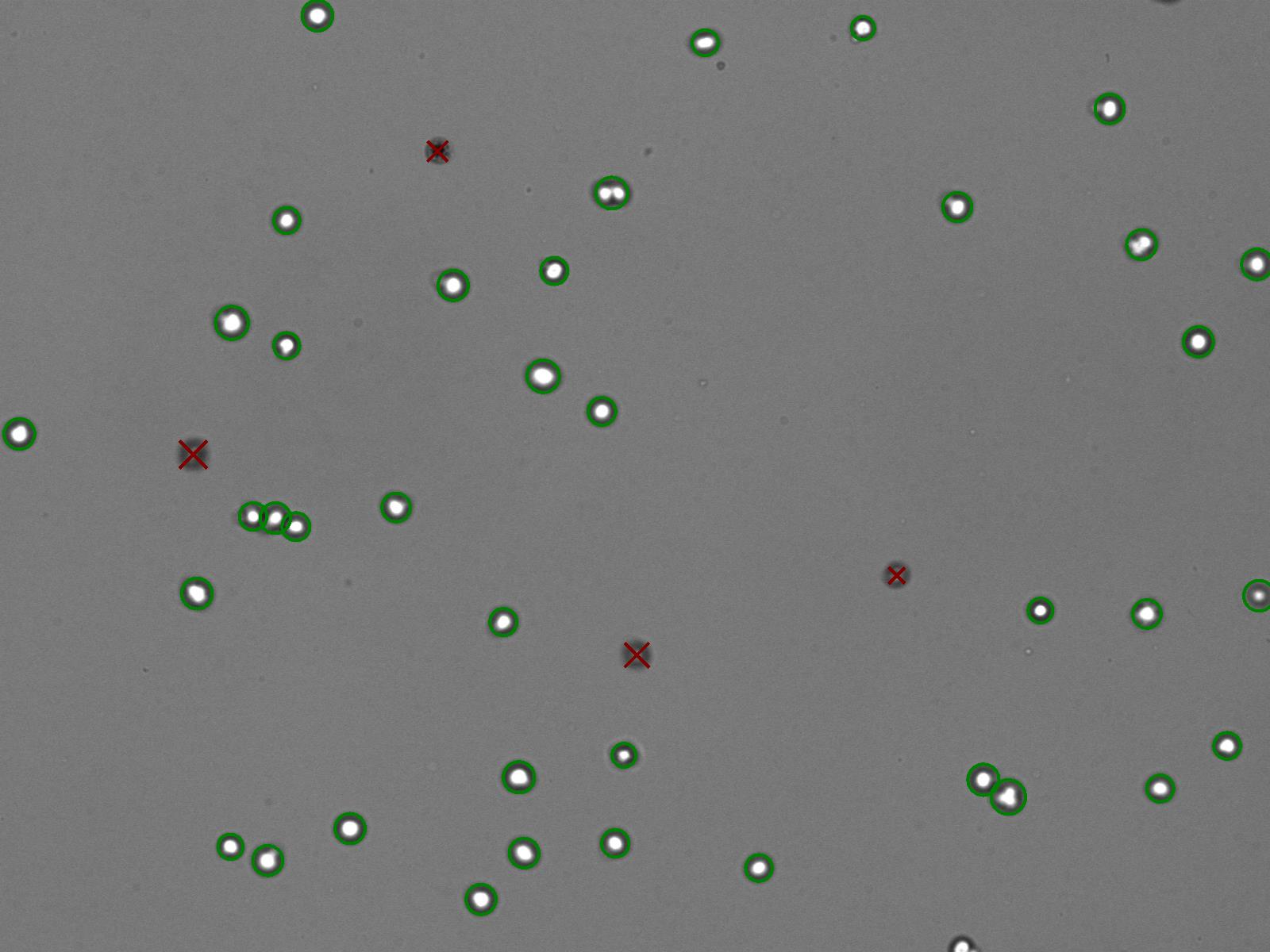

Supplement: Supplementary file 1 — Supplementary Information 1. [file 41598_2020_80576_MOESM1_ESM.zip › S1/Aggregate counts/day5/30mmHg Jan18 47 43/ML P3-011_2019-02-11_151846.bmp]

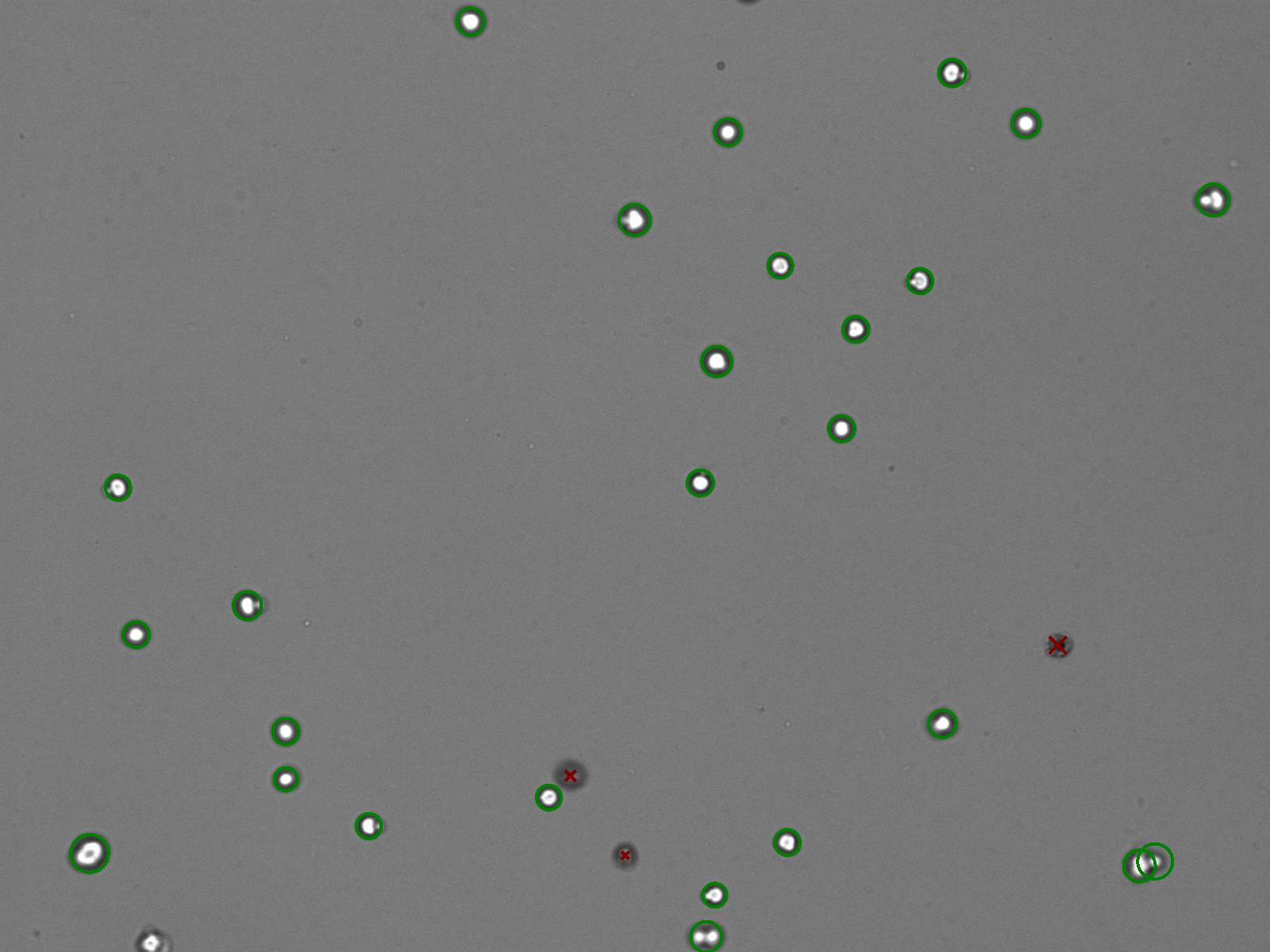

Supplement: Supplementary file 1 — Supplementary Information 1. [file 41598_2020_80576_MOESM1_ESM.zip › S1/Aggregate counts/day5/30mmHg Jan18 47 43/ML P3-012_2019-02-11_151847.bmp]

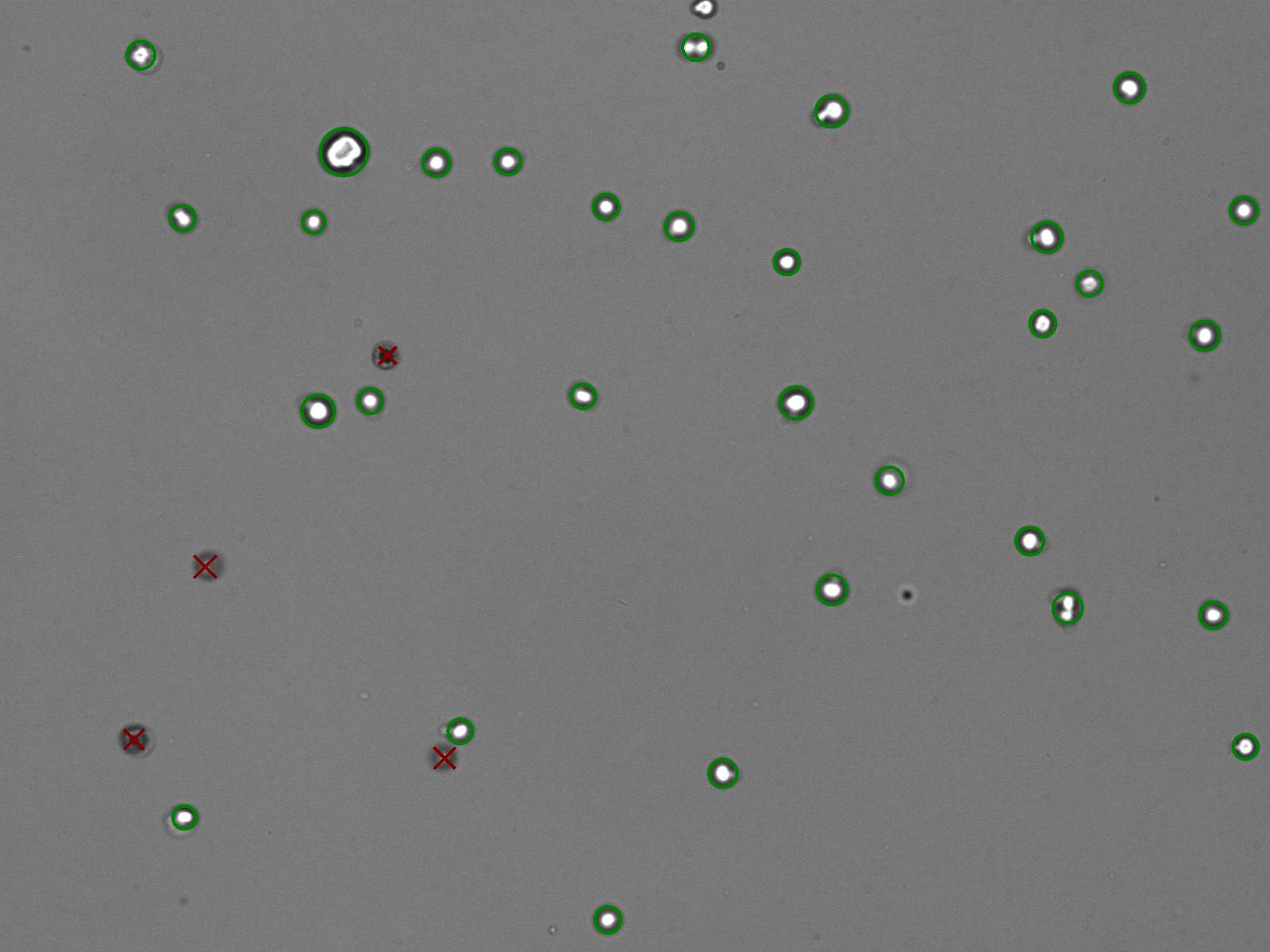

Supplement: Supplementary file 1 — Supplementary Information 1. [file 41598_2020_80576_MOESM1_ESM.zip › S1/Aggregate counts/day5/30mmHg Jan18 47 43/ML P3-013_2019-02-11_151847.bmp]

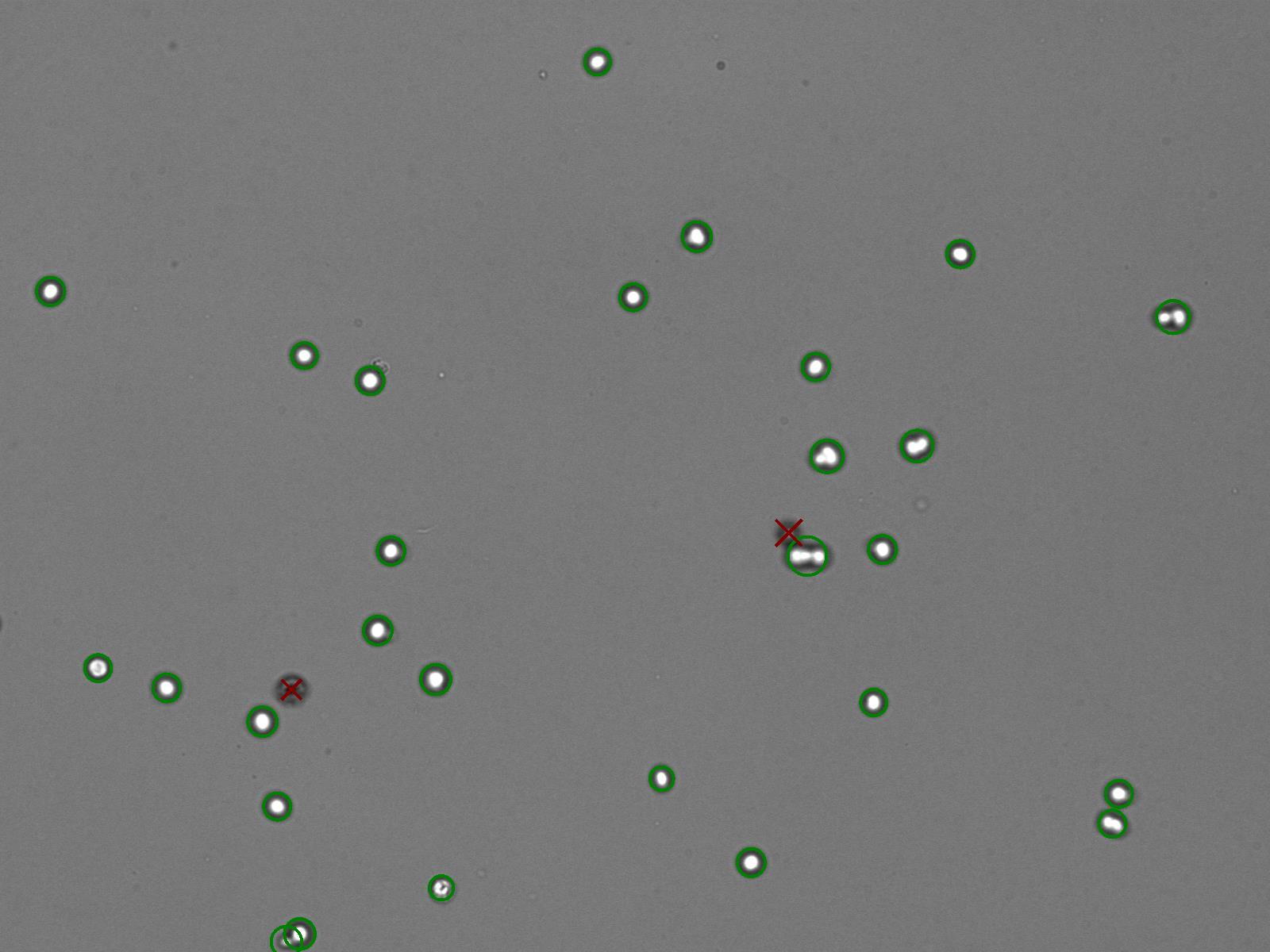

Supplement: Supplementary file 1 — Supplementary Information 1. [file 41598_2020_80576_MOESM1_ESM.zip › S1/Aggregate counts/day5/30mmHg Jan18 47 43/ML P3-014_2019-02-11_151847.bmp]

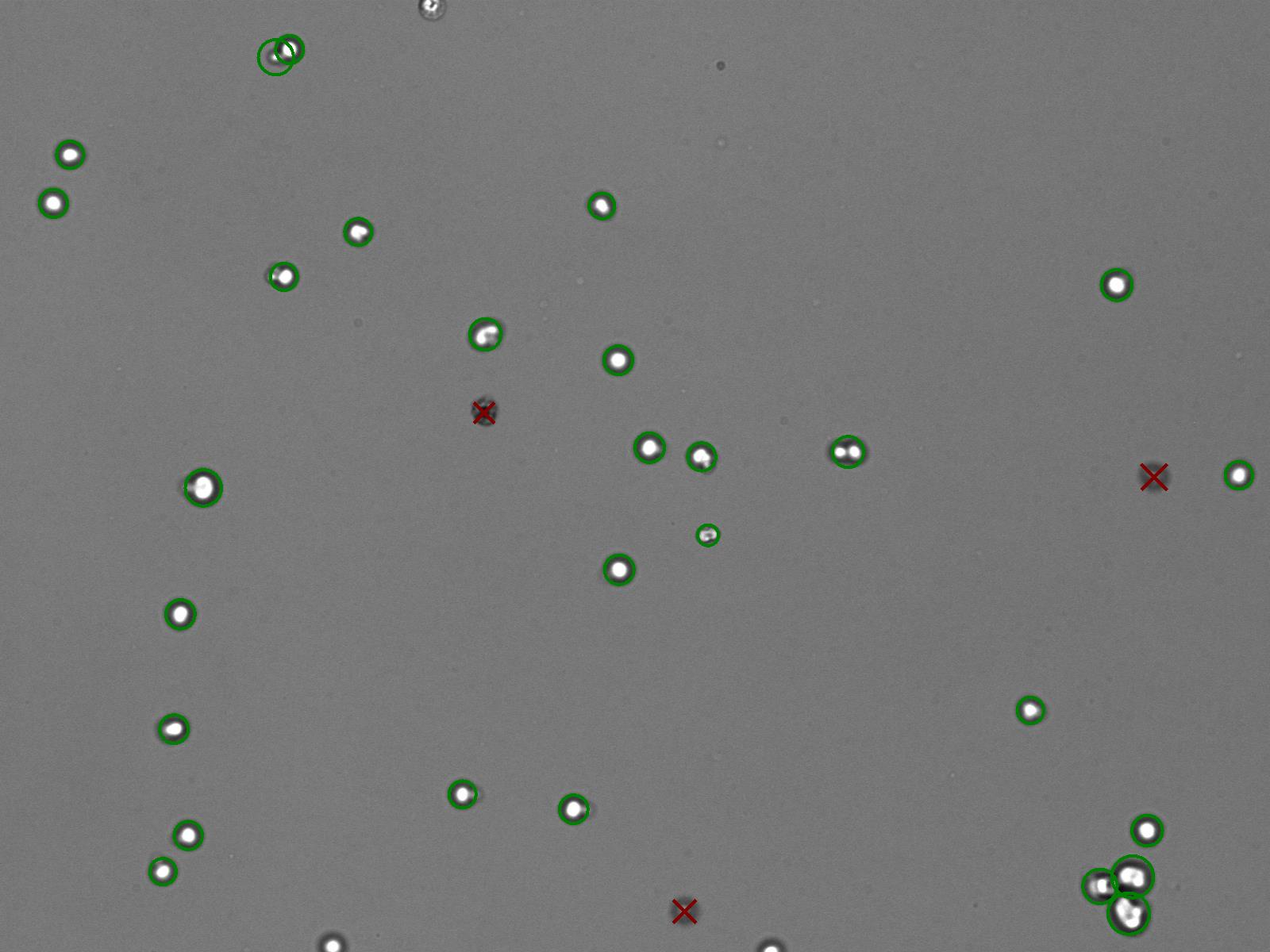

Supplement: Supplementary file 1 — Supplementary Information 1. [file 41598_2020_80576_MOESM1_ESM.zip › S1/Aggregate counts/day5/30mmHg Jan18 47 43/ML P3-015_2019-02-11_151847.bmp]

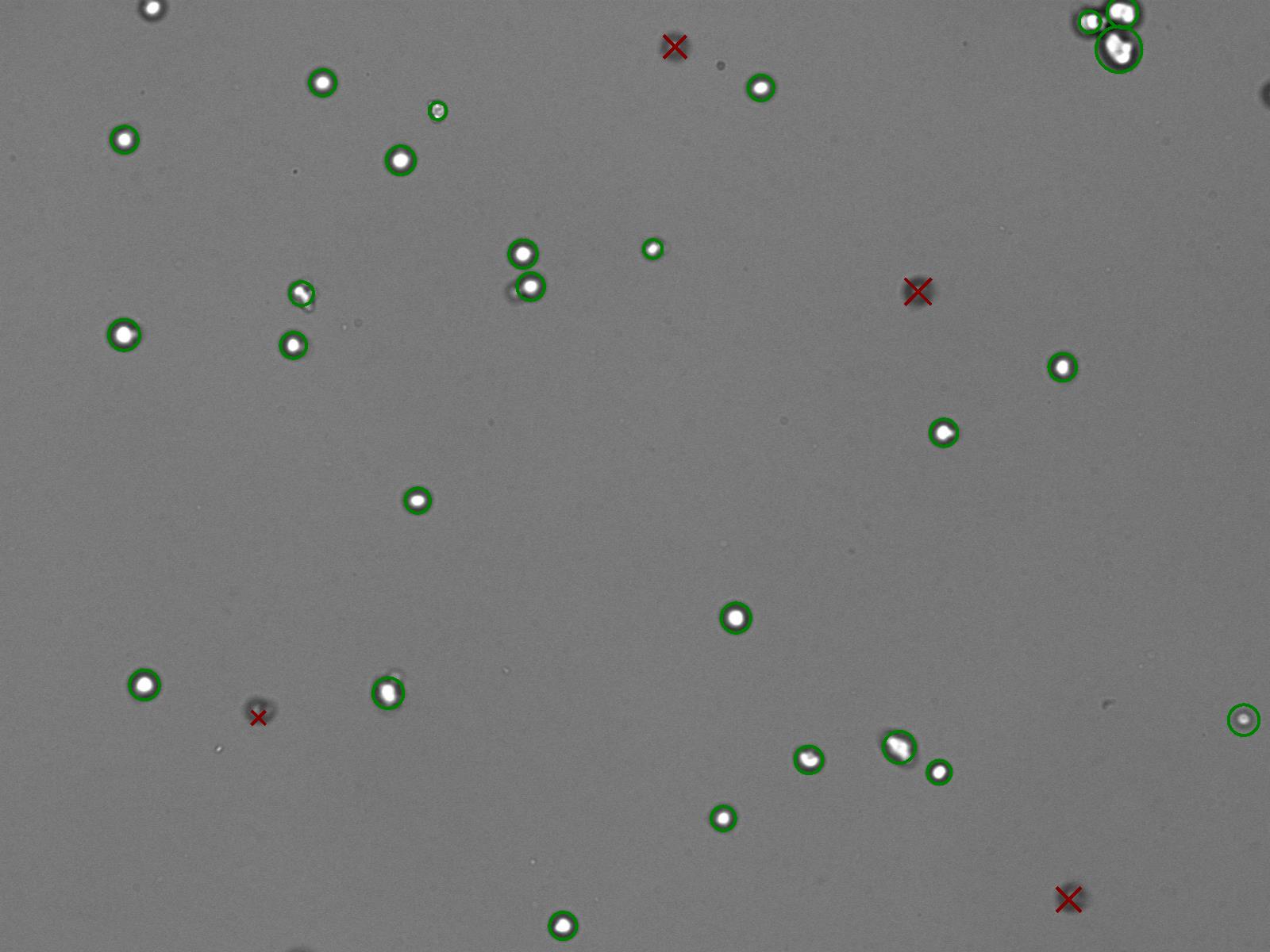

Supplement: Supplementary file 1 — Supplementary Information 1. [file 41598_2020_80576_MOESM1_ESM.zip › S1/Aggregate counts/day5/30mmHg Jan18 47 43/ML P3-016_2019-02-11_151848.bmp]

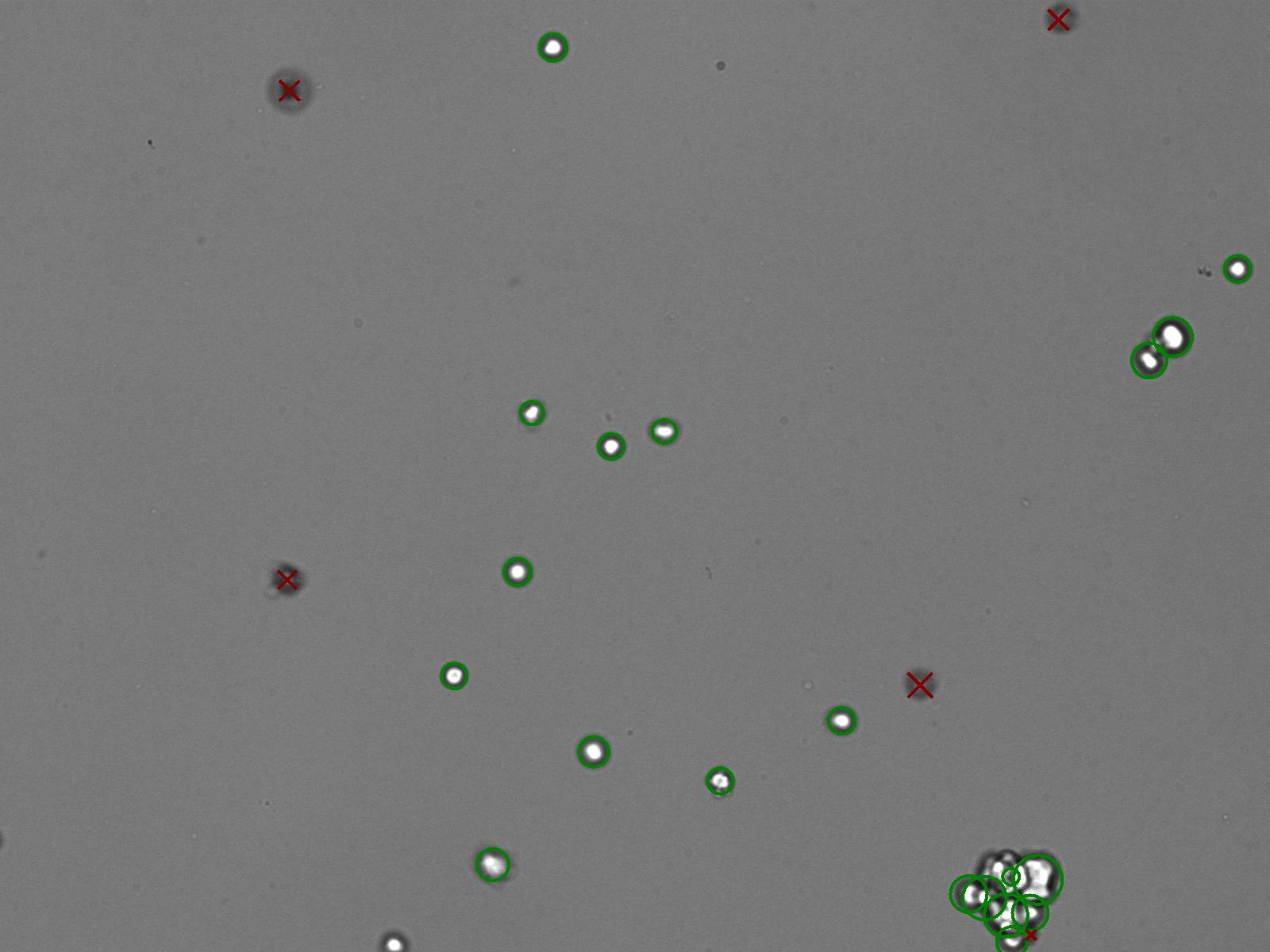

Supplement: Supplementary file 1 — Supplementary Information 1. [file 41598_2020_80576_MOESM1_ESM.zip › S1/Aggregate counts/day5/30mmHg Jan18 47 43/ML P3-017_2019-02-11_151848.bmp]

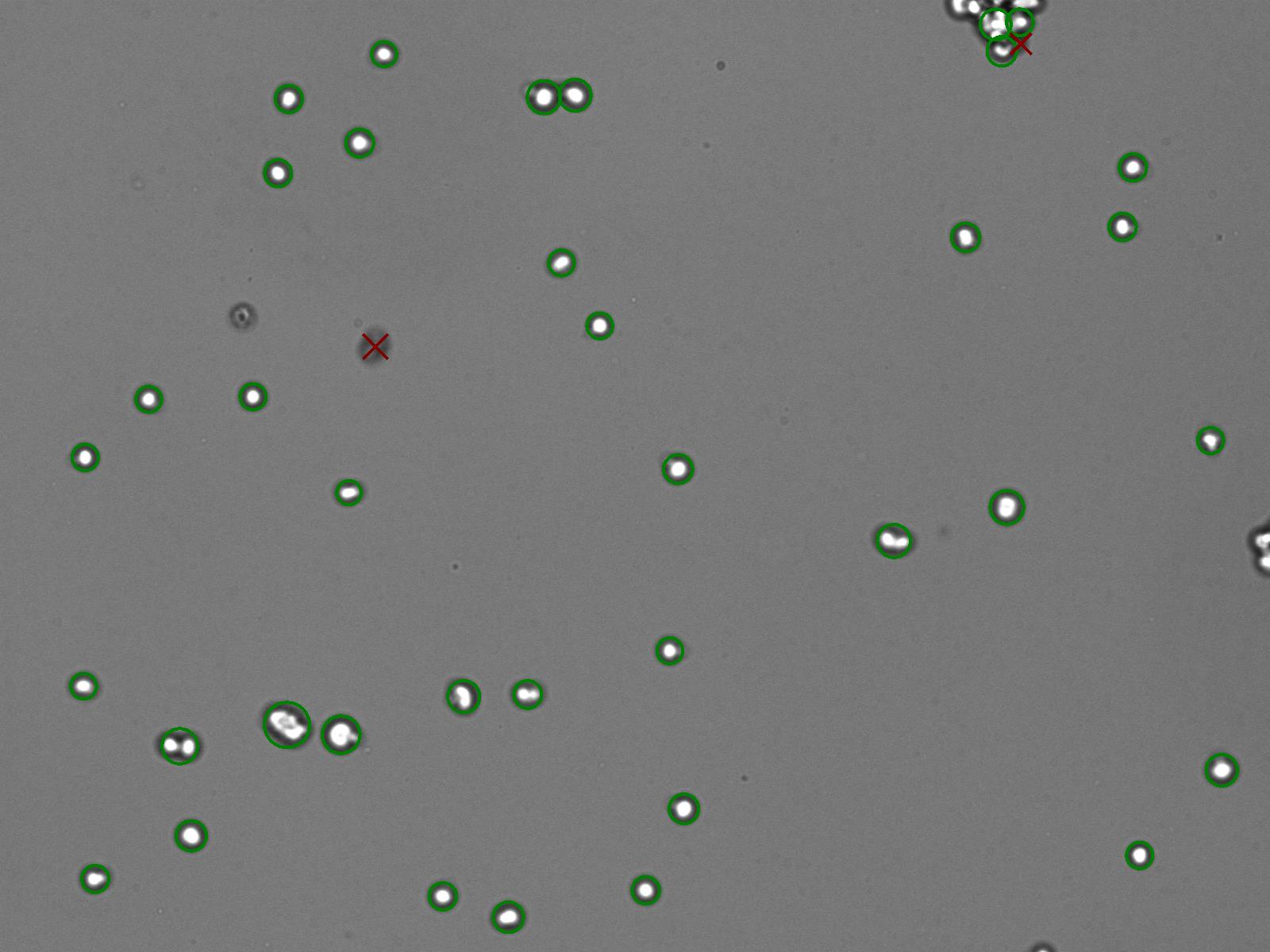

Supplement: Supplementary file 1 — Supplementary Information 1. [file 41598_2020_80576_MOESM1_ESM.zip › S1/Aggregate counts/day5/30mmHg Jan18 47 43/ML P3-018_2019-02-11_151849.bmp]

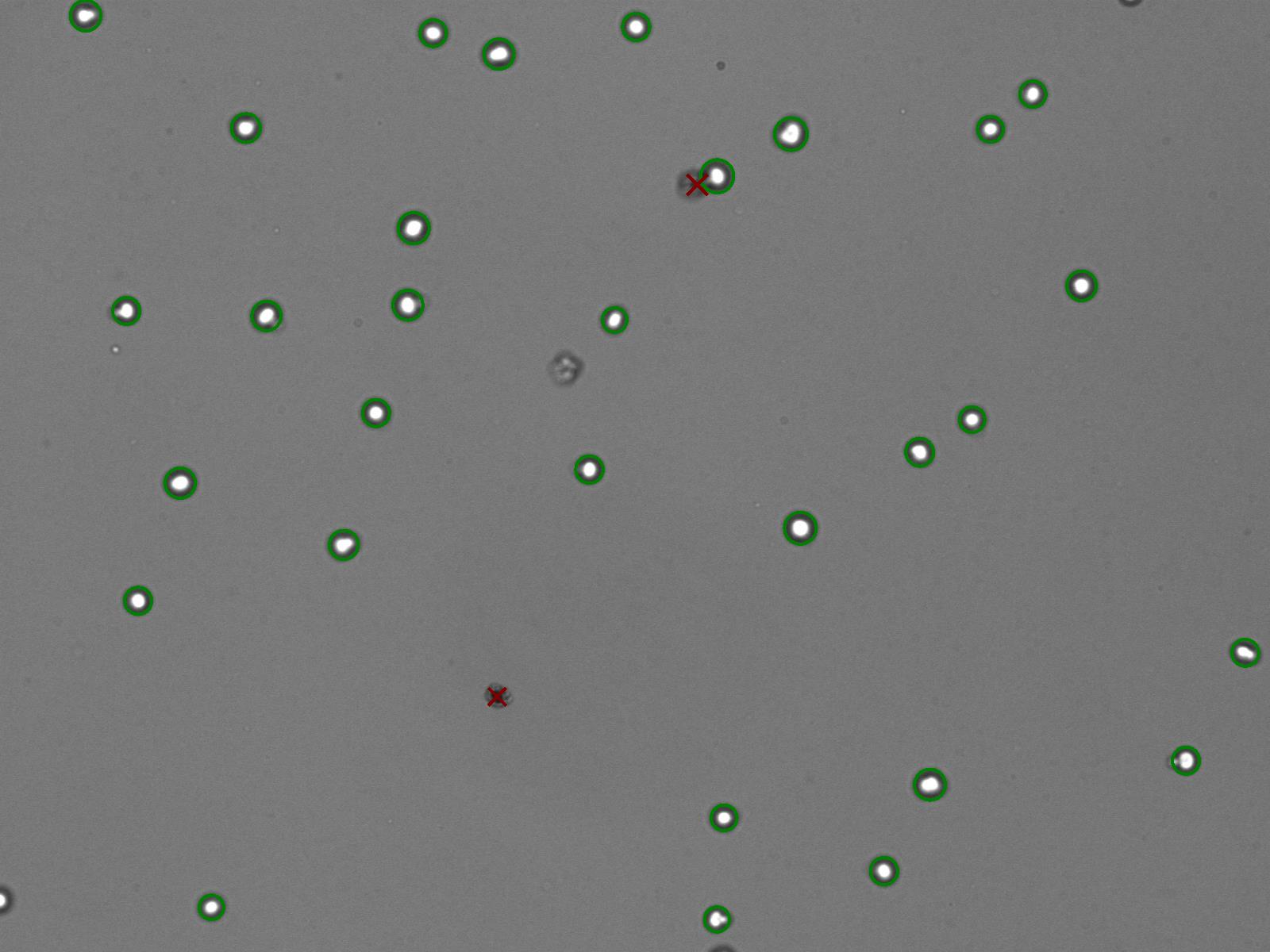

Supplement: Supplementary file 1 — Supplementary Information 1. [file 41598_2020_80576_MOESM1_ESM.zip › S1/Aggregate counts/day5/30mmHg Jan18 47 43/ML P3-019_2019-02-11_151849.bmp]

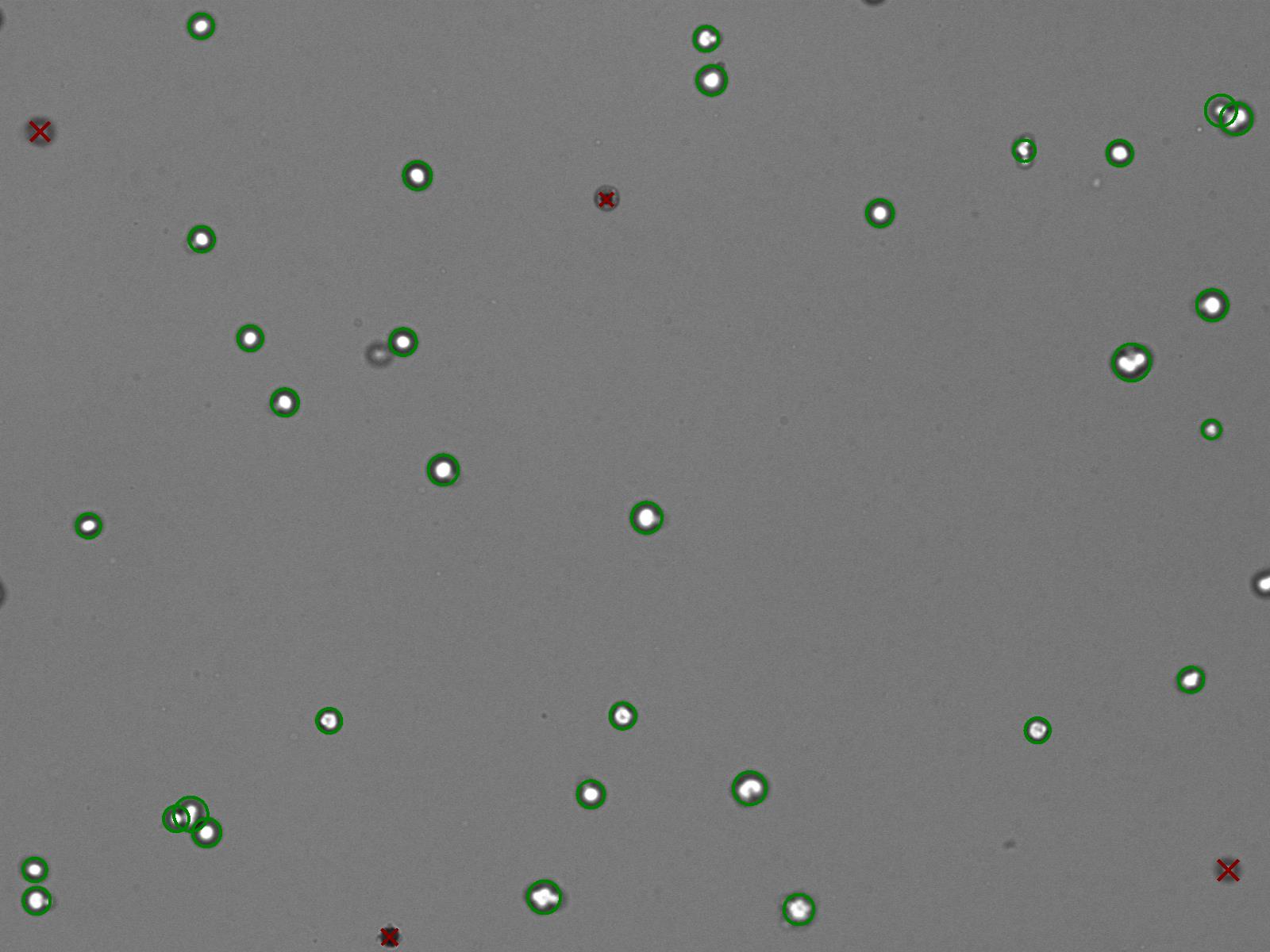

Supplement: Supplementary file 1 — Supplementary Information 1. [file 41598_2020_80576_MOESM1_ESM.zip › S1/Aggregate counts/day5/30mmHg Jan18 47 43/ML P3-020_2019-02-11_151849.bmp]

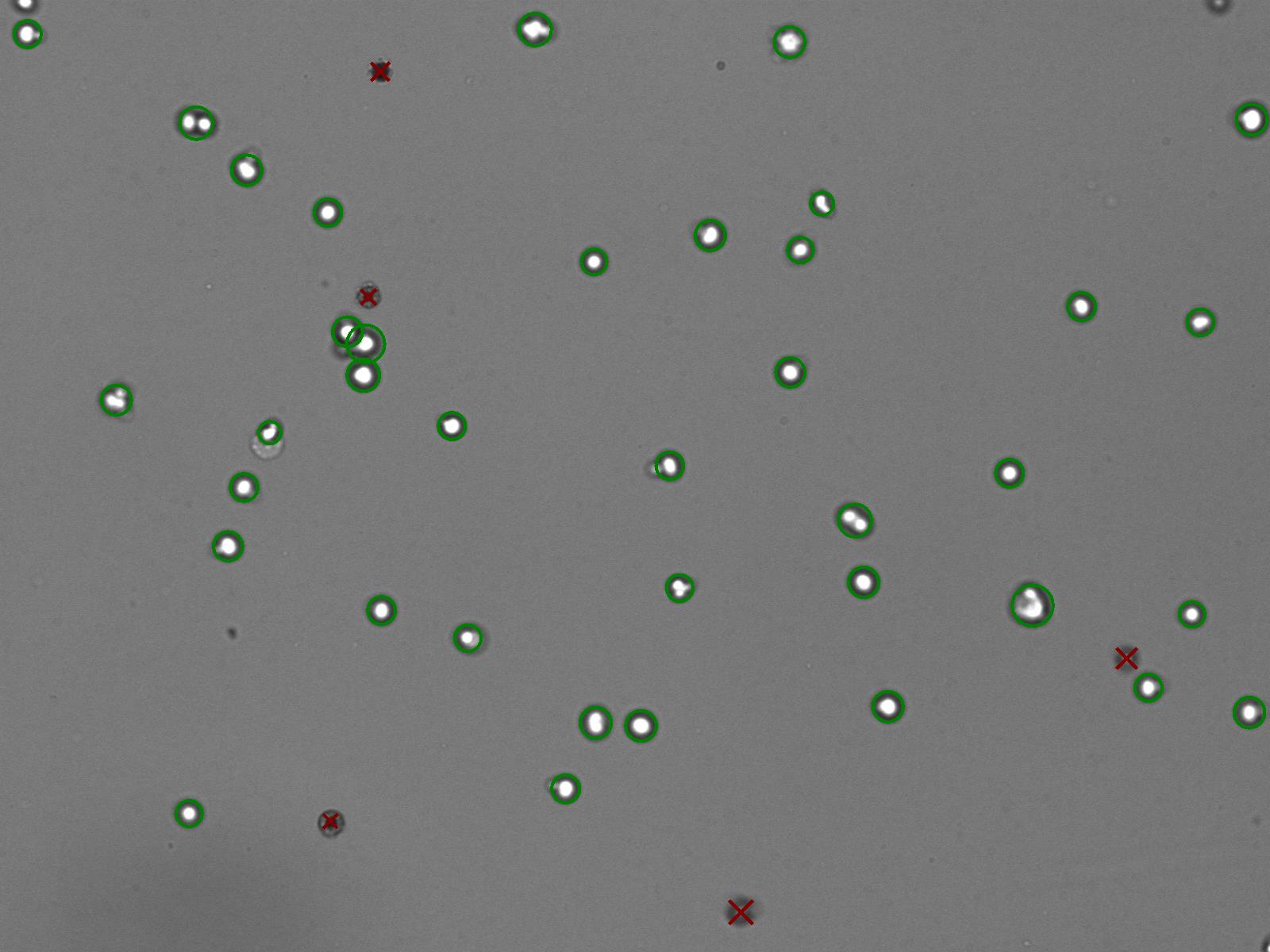

Supplement: Supplementary file 1 — Supplementary Information 1. [file 41598_2020_80576_MOESM1_ESM.zip › S1/Aggregate counts/day5/30mmHg Jan18 47 43/ML P3-021_2019-02-11_151850.bmp]

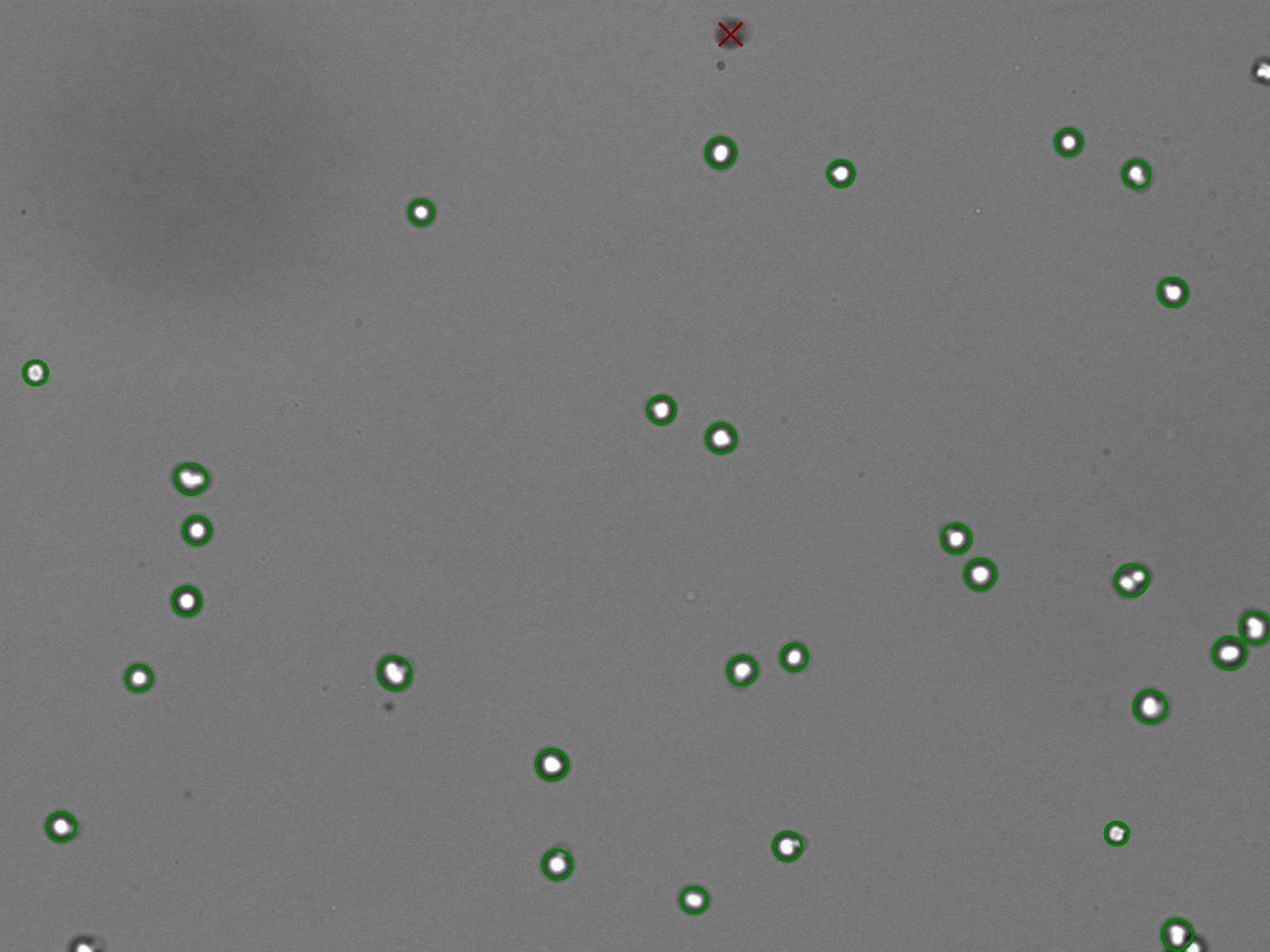

Supplement: Supplementary file 1 — Supplementary Information 1. [file 41598_2020_80576_MOESM1_ESM.zip › S1/Aggregate counts/day5/30mmHg Jan18 47 43/ML P3-022_2019-02-11_151850.bmp]

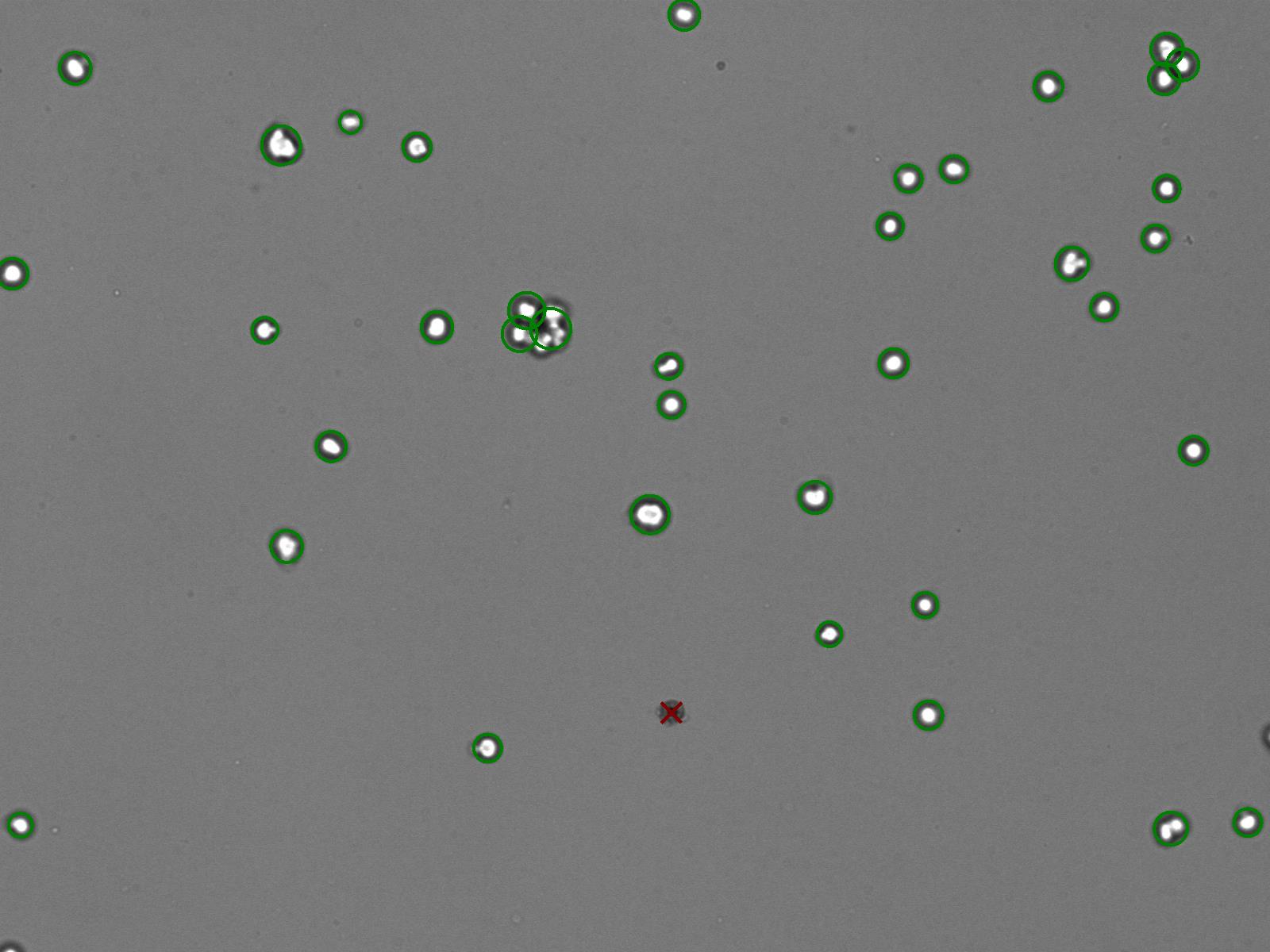

Supplement: Supplementary file 1 — Supplementary Information 1. [file 41598_2020_80576_MOESM1_ESM.zip › S1/Aggregate counts/day5/30mmHg Jan18 47 43/ML P3-023_2019-02-11_151850.bmp]

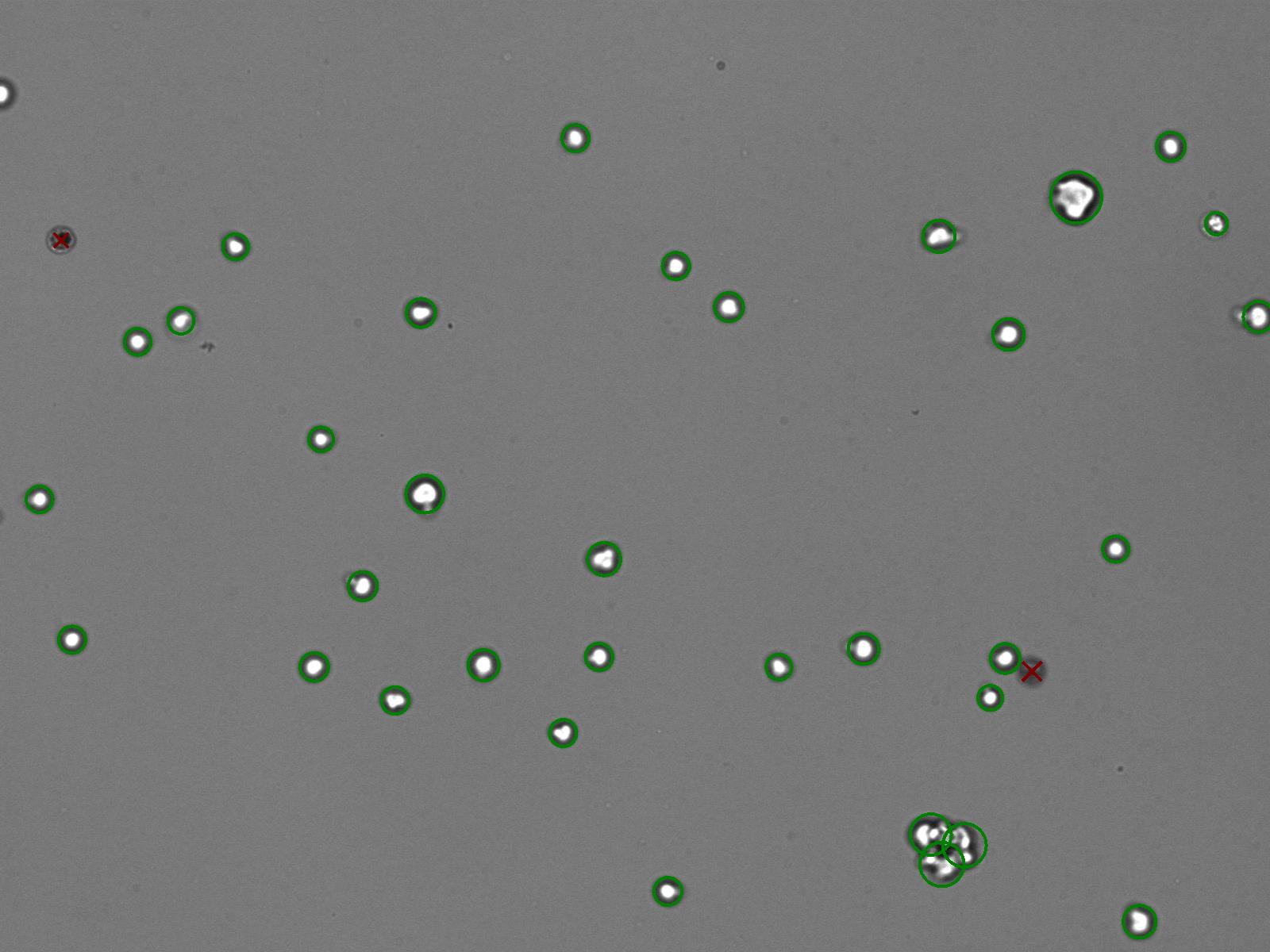

Supplement: Supplementary file 1 — Supplementary Information 1. [file 41598_2020_80576_MOESM1_ESM.zip › S1/Aggregate counts/day5/30mmHg Jan18 47 43/ML P3-024_2019-02-11_151850.bmp]

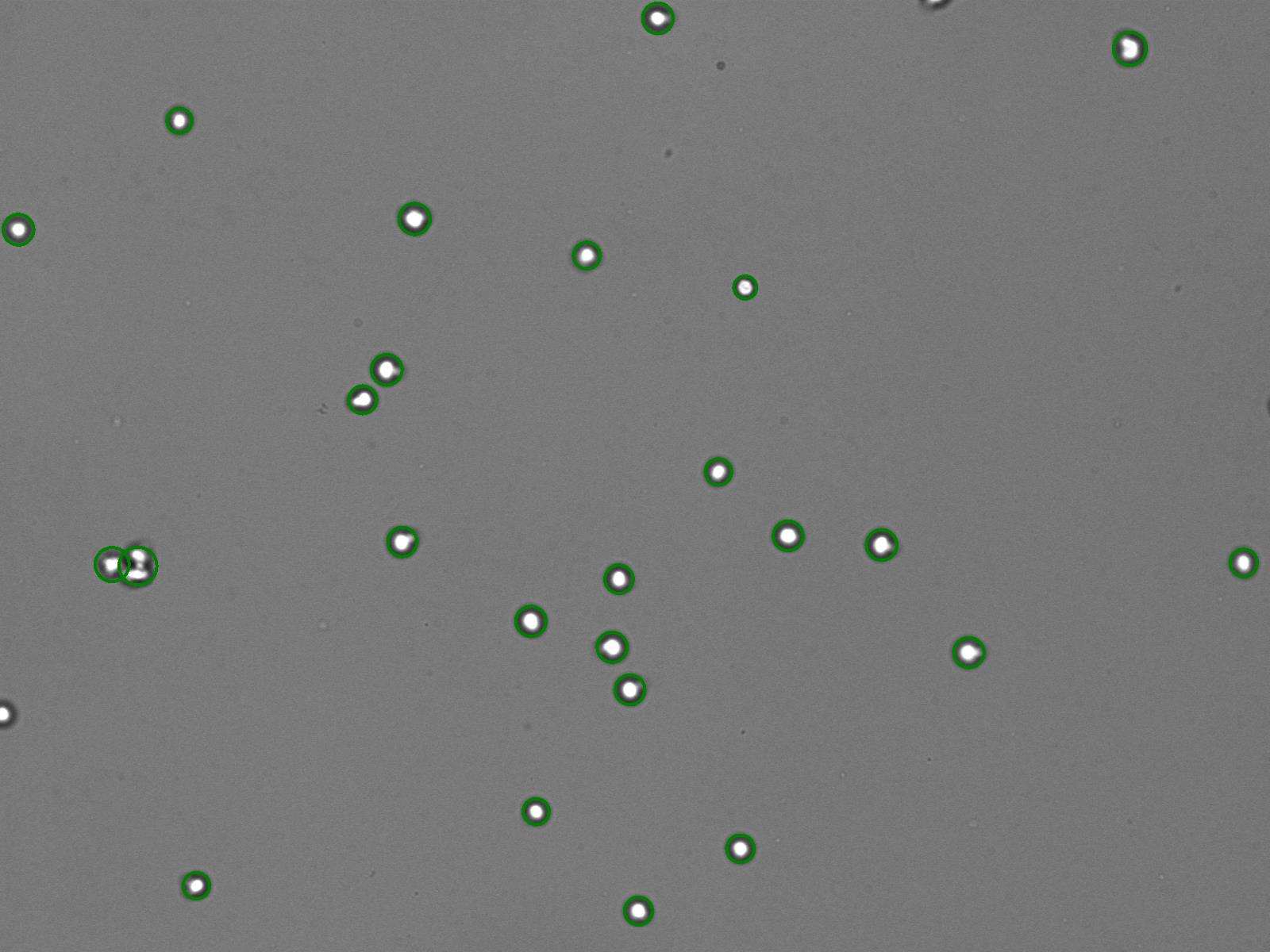

Supplement: Supplementary file 1 — Supplementary Information 1. [file 41598_2020_80576_MOESM1_ESM.zip › S1/Aggregate counts/day5/30mmHg Jan18 47 43/ML P3-025_2019-02-11_151851.bmp]

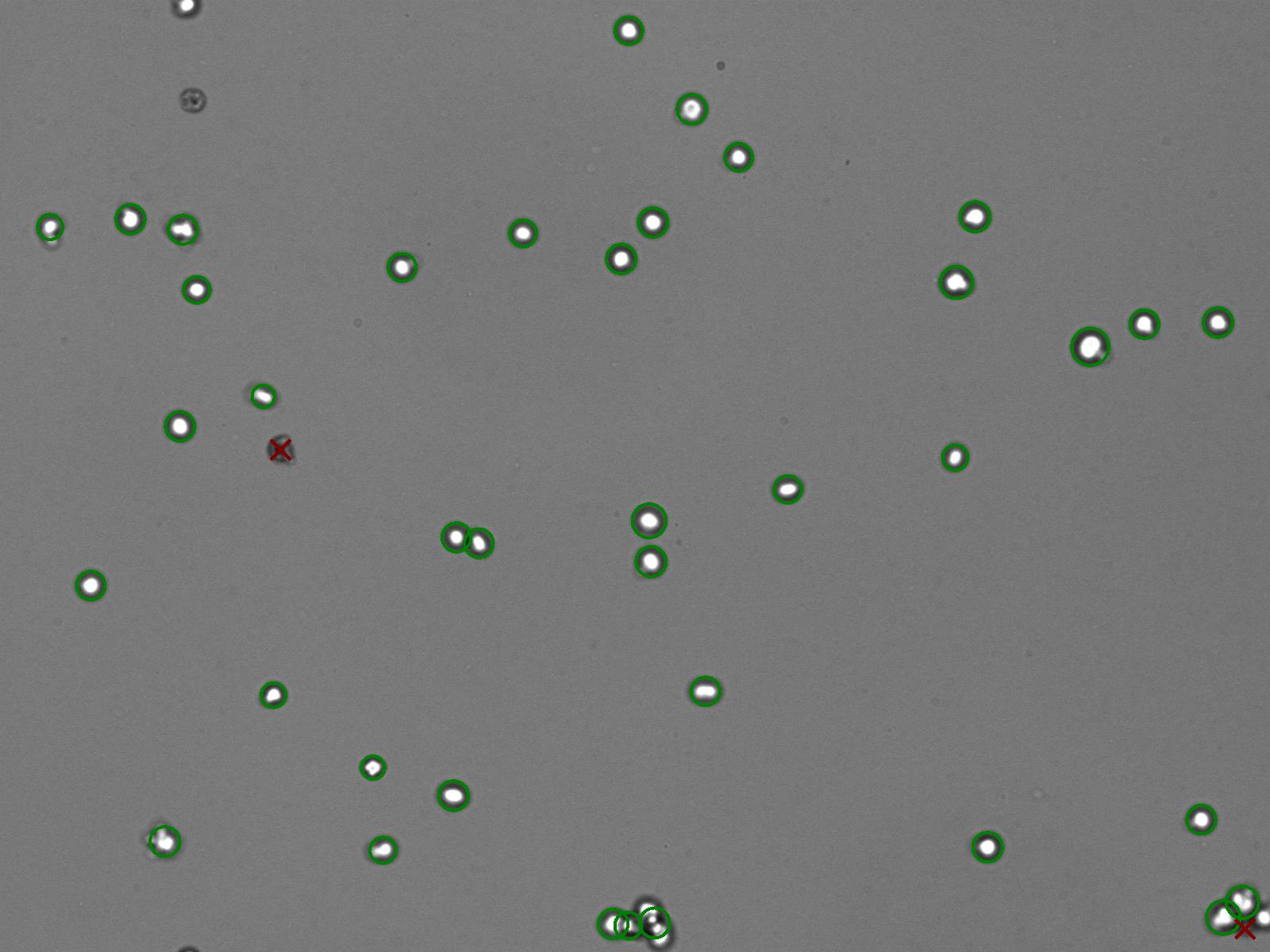

Supplement: Supplementary file 1 — Supplementary Information 1. [file 41598_2020_80576_MOESM1_ESM.zip › S1/Aggregate counts/day5/30mmHg Jan18 47 43/ML P3-026_2019-02-11_151851.bmp]

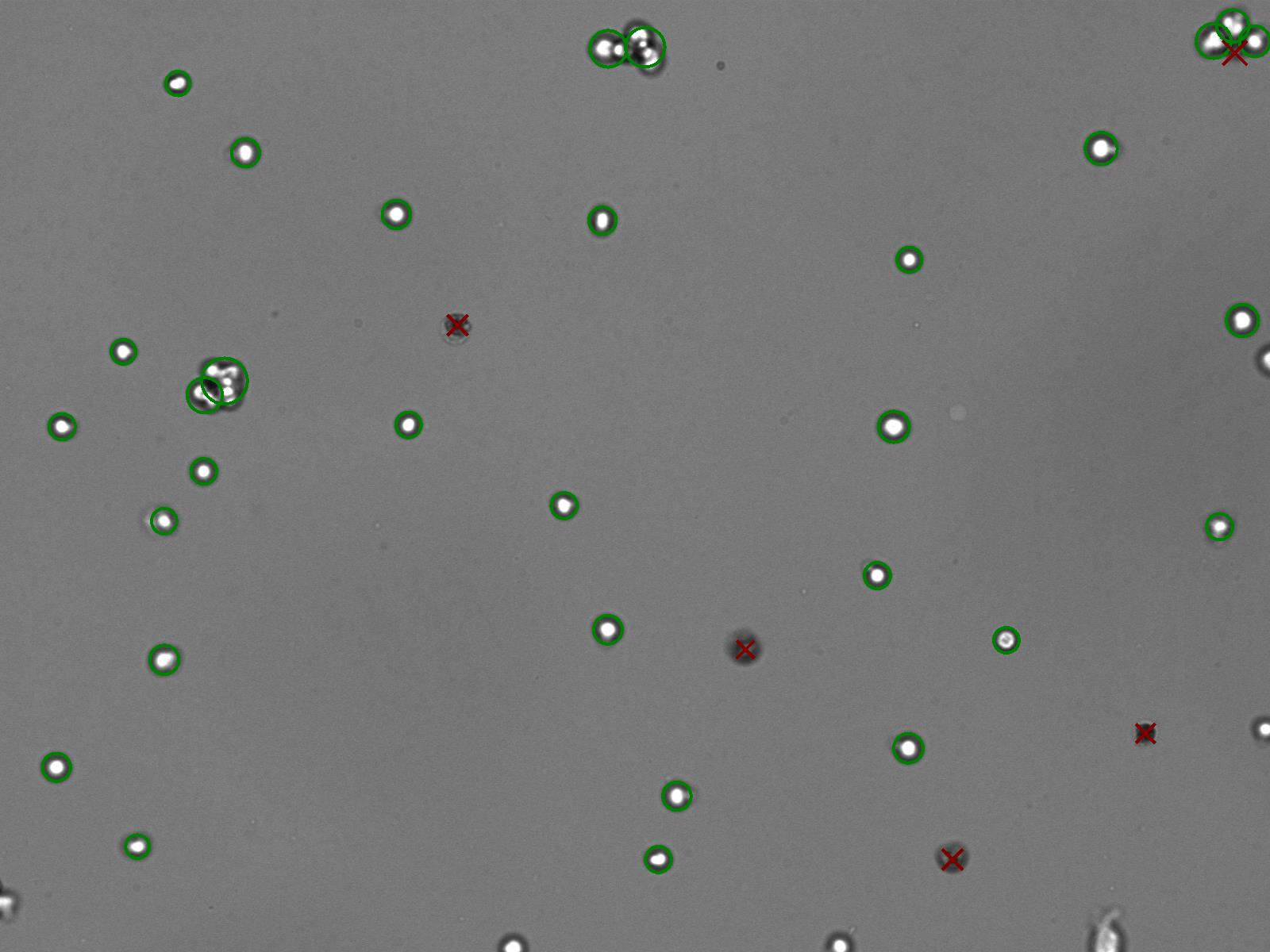

Supplement: Supplementary file 1 — Supplementary Information 1. [file 41598_2020_80576_MOESM1_ESM.zip › S1/Aggregate counts/day5/30mmHg Jan18 47 43/ML P3-027_2019-02-11_151851.bmp]

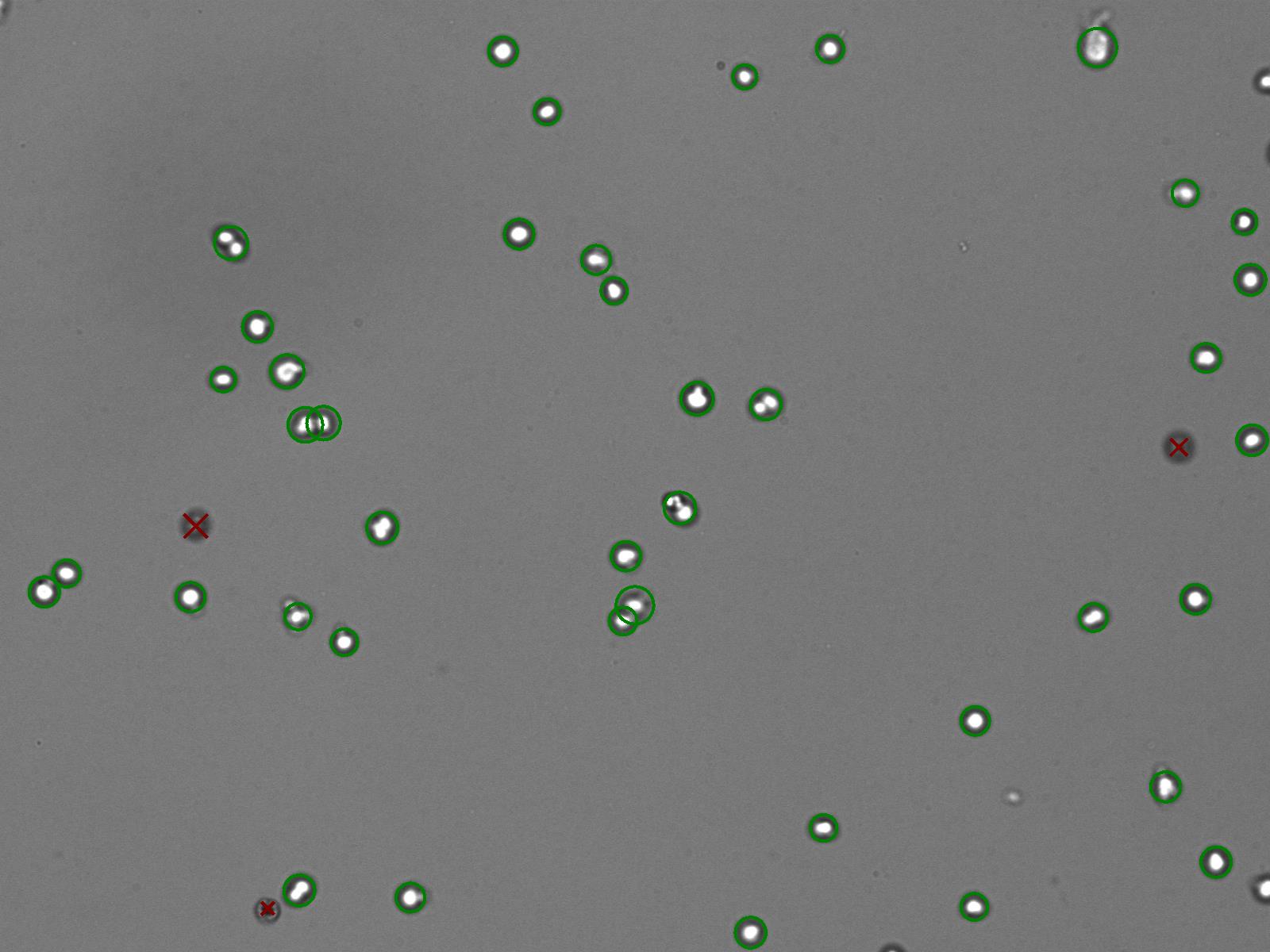

Supplement: Supplementary file 1 — Supplementary Information 1. [file 41598_2020_80576_MOESM1_ESM.zip › S1/Aggregate counts/day5/30mmHg Jan18 47 43/ML P3-028_2019-02-11_151852.bmp]

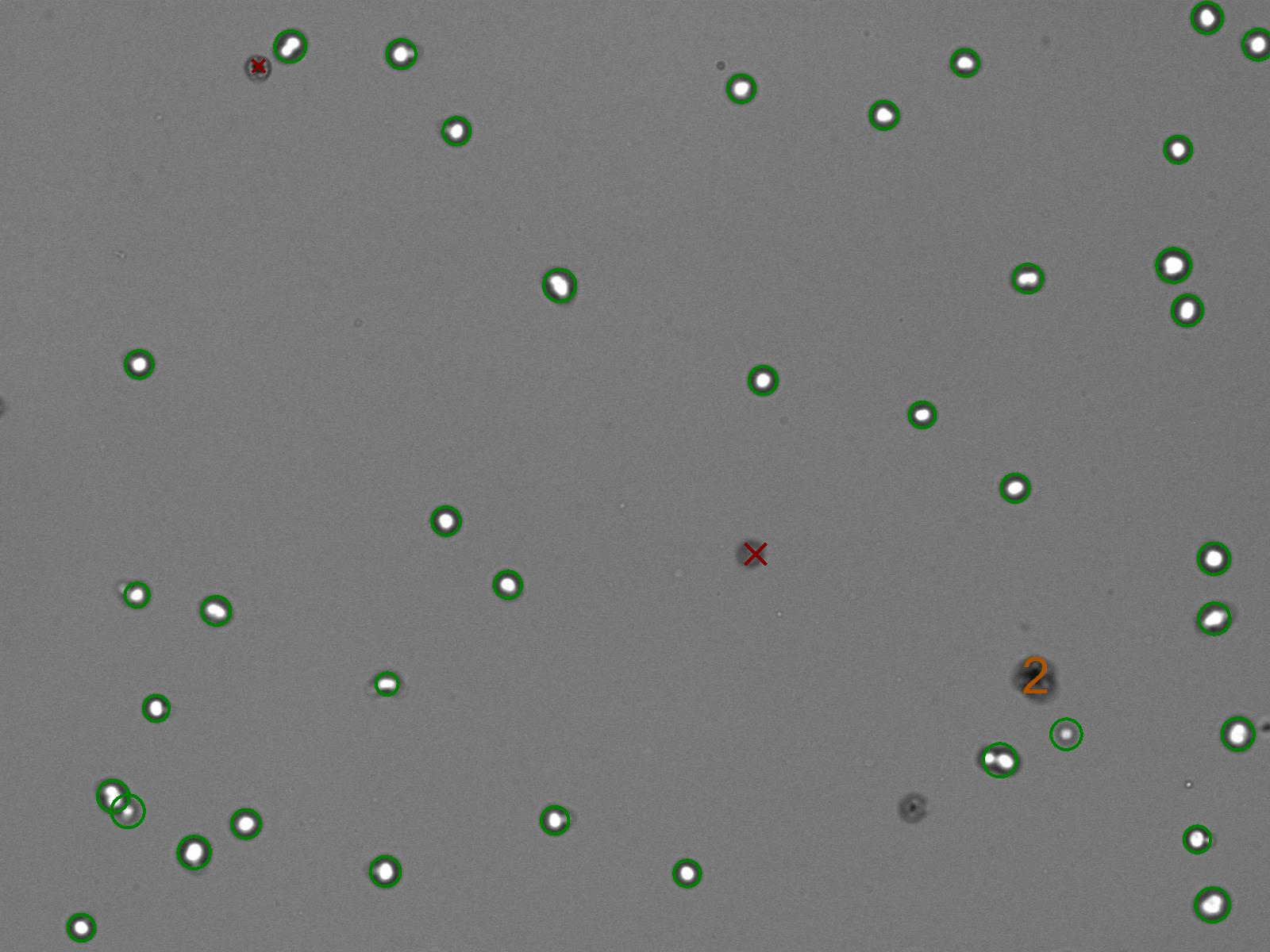

Supplement: Supplementary file 1 — Supplementary Information 1. [file 41598_2020_80576_MOESM1_ESM.zip › S1/Aggregate counts/day5/30mmHg Jan18 47 43/ML P3-029_2019-02-11_151852.bmp]

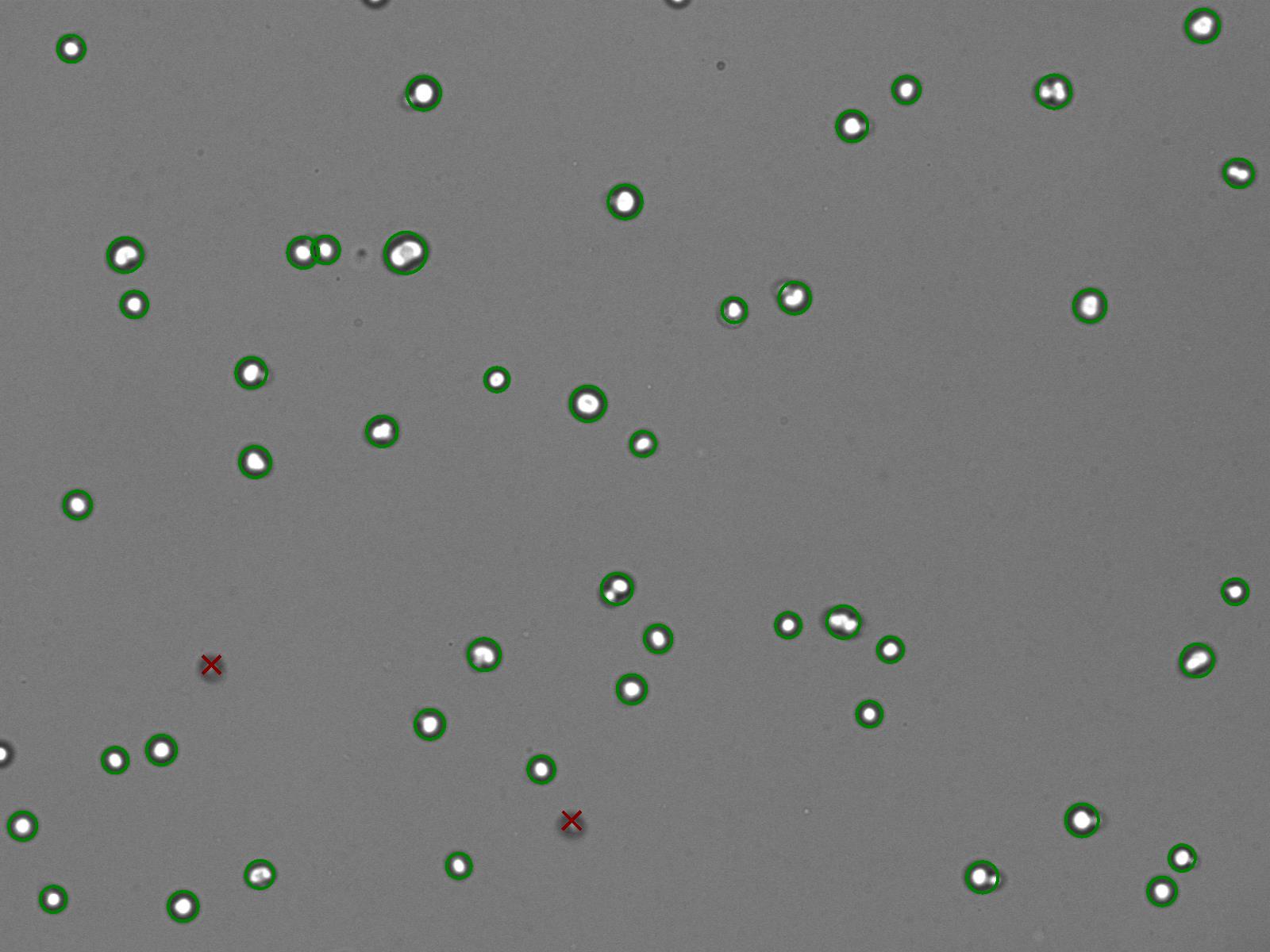

Supplement: Supplementary file 1 — Supplementary Information 1. [file 41598_2020_80576_MOESM1_ESM.zip › S1/Aggregate counts/day5/30mmHg Jan18 47 43/ML P3-030_2019-02-11_151852.bmp]

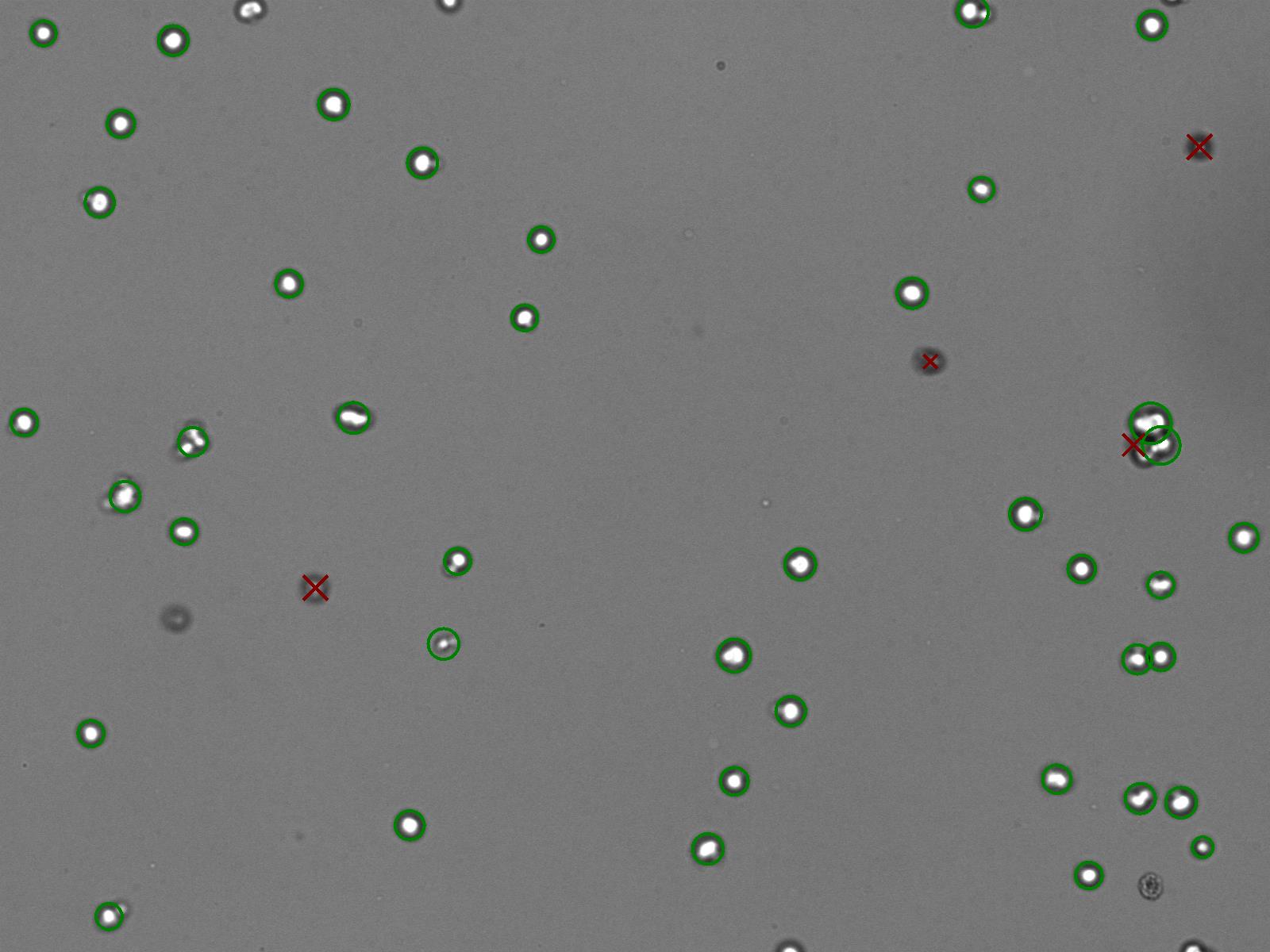

Supplement: Supplementary file 1 — Supplementary Information 1. [file 41598_2020_80576_MOESM1_ESM.zip › S1/Aggregate counts/day5/30mmHg Jan18 47 43/ML P3-031_2019-02-11_151853.bmp]

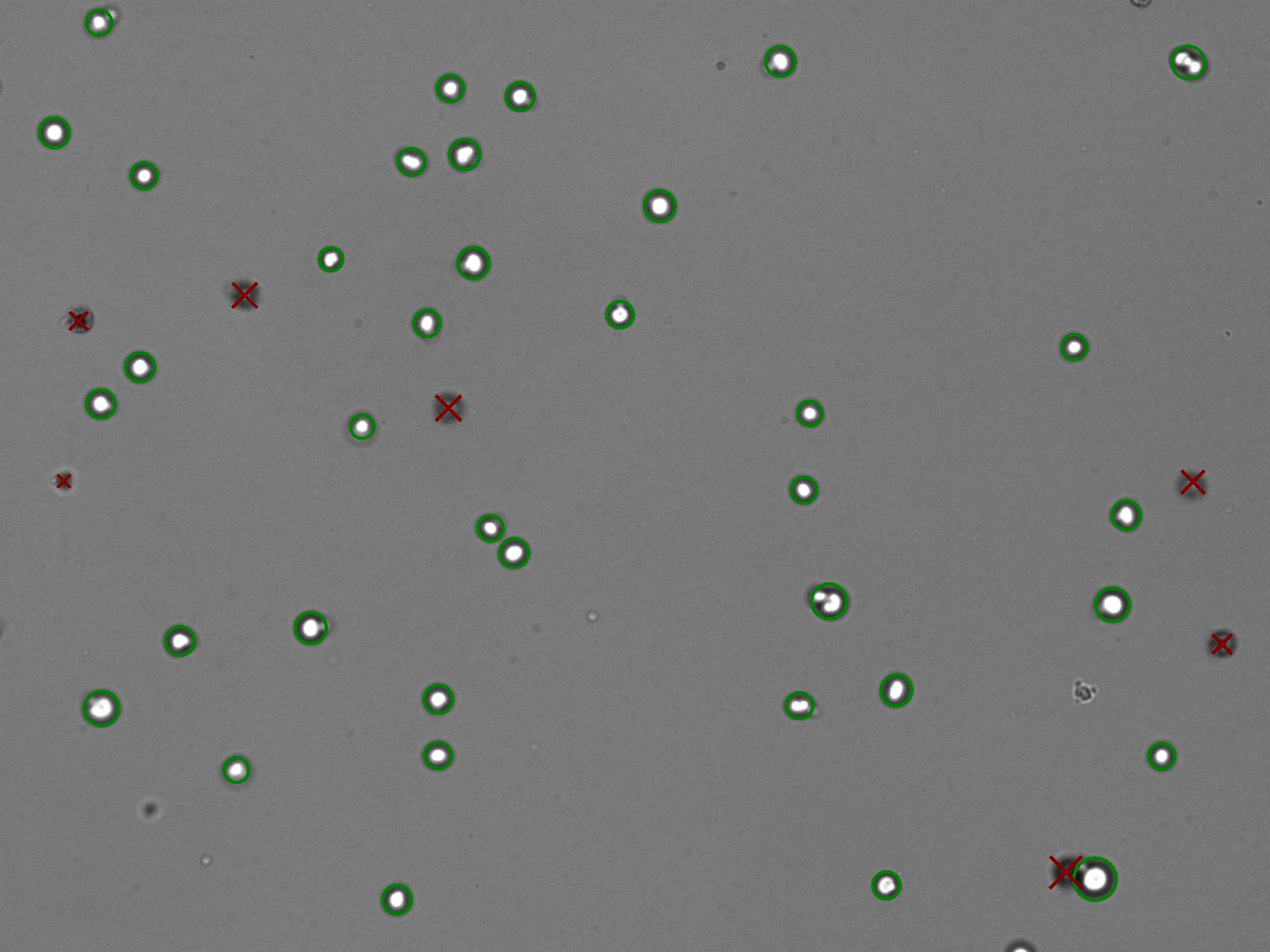

Supplement: Supplementary file 1 — Supplementary Information 1. [file 41598_2020_80576_MOESM1_ESM.zip › S1/Aggregate counts/day5/30mmHg Jan18 47 43/ML P3-032_2019-02-11_151853.bmp]
