# Supplementary figures and images for: Investigating the influence of physiologically relevant hydrostatic pressure on CHO cell batch culture (part 1 of 2)
Source: Sci Rep. 2021 Jan 8;11:162. doi: 10.1038/s41598-020-80576-8 (PMC7794228; doi:10.1038/s41598-020-80576-8)

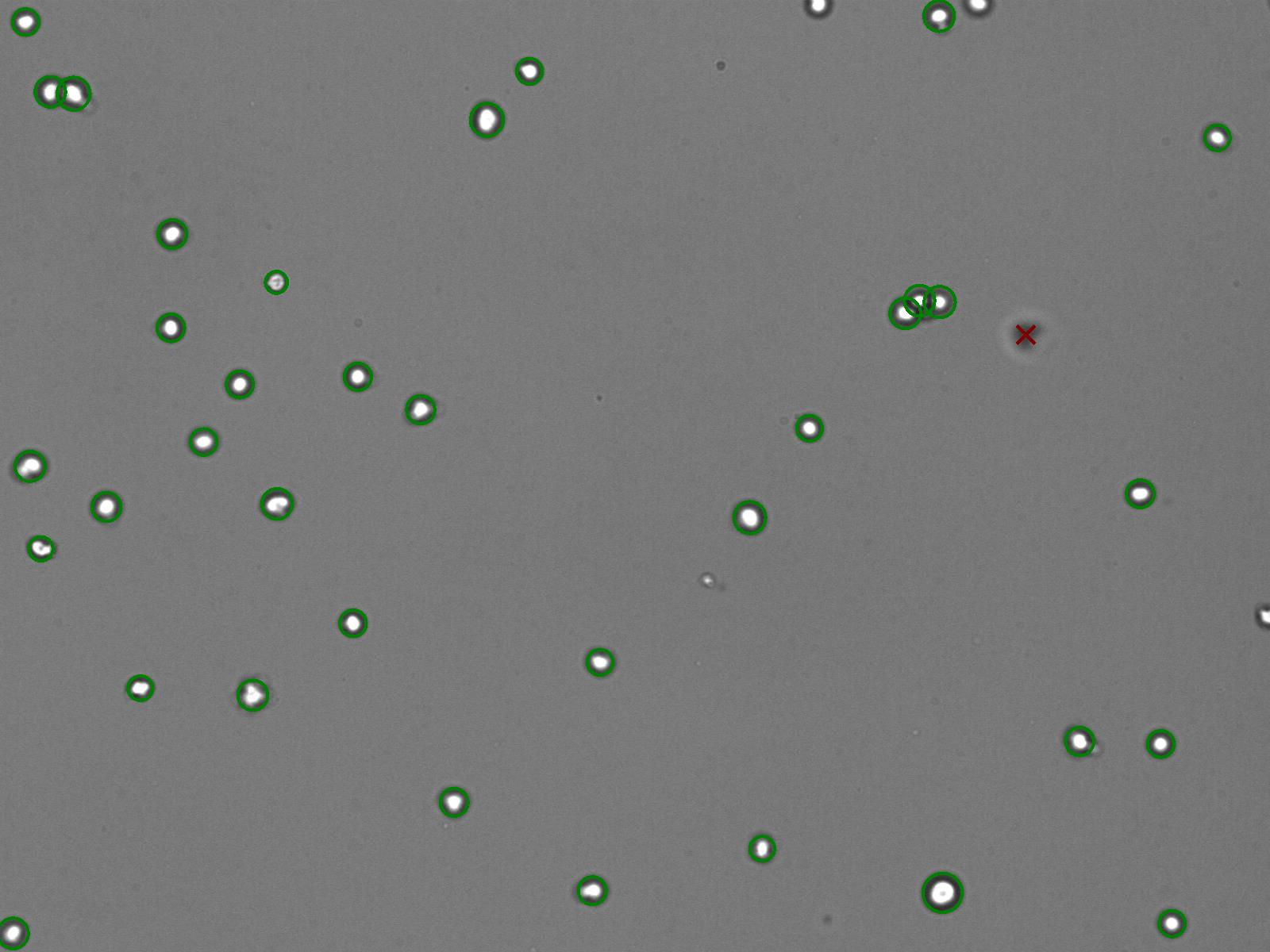

Supplement: Supplementary file 1 — Supplementary Information 1. [file 41598_2020_80576_MOESM1_ESM.zip › S1/Aggregate counts/day5/0mmHg Feb1 47 46/ML C3-001_2019-02-11_153016.bmp]

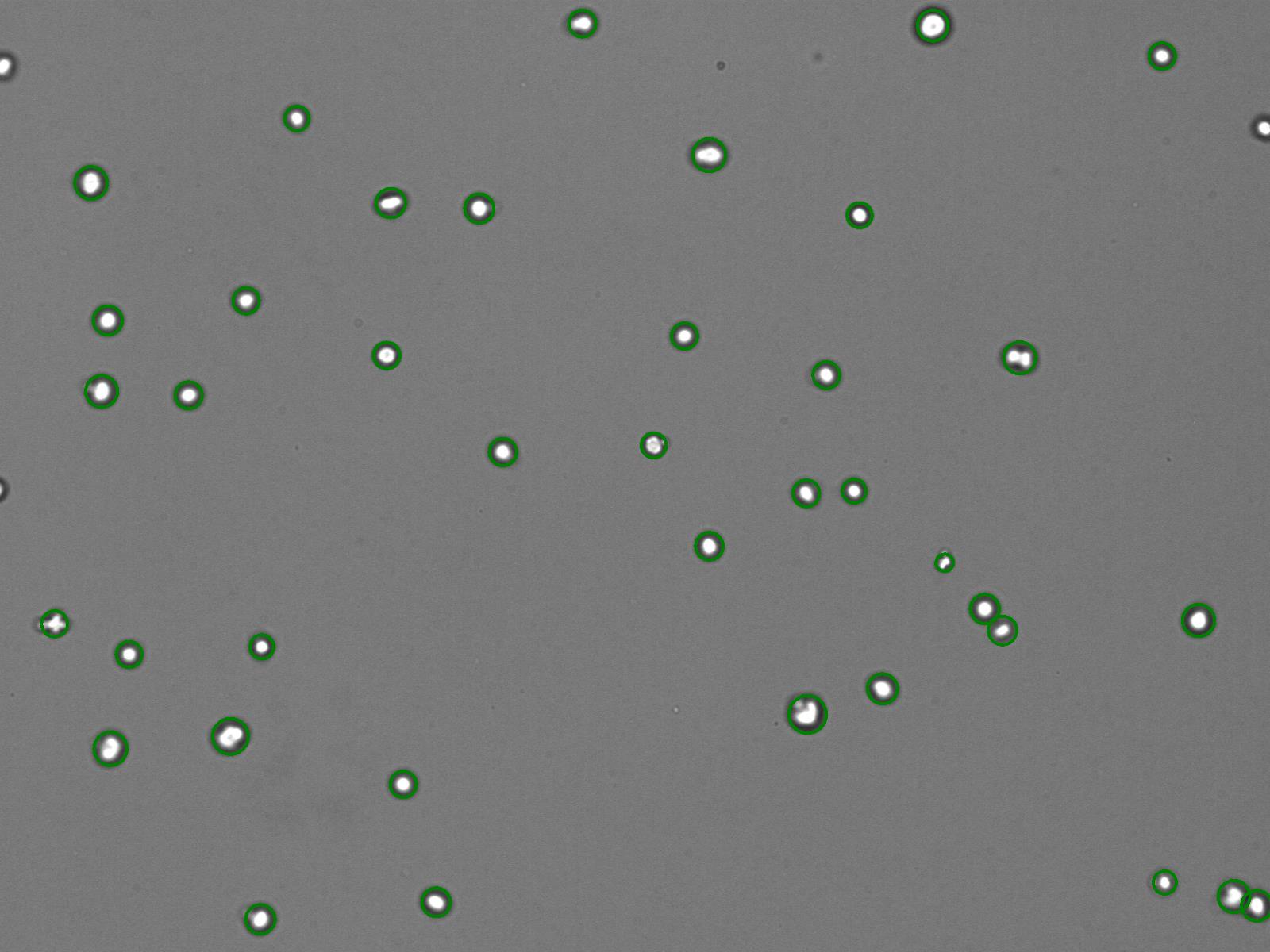

Supplement: Supplementary file 1 — Supplementary Information 1. [file 41598_2020_80576_MOESM1_ESM.zip › S1/Aggregate counts/day5/0mmHg Feb1 47 46/ML C3-002_2019-02-11_153016.bmp]

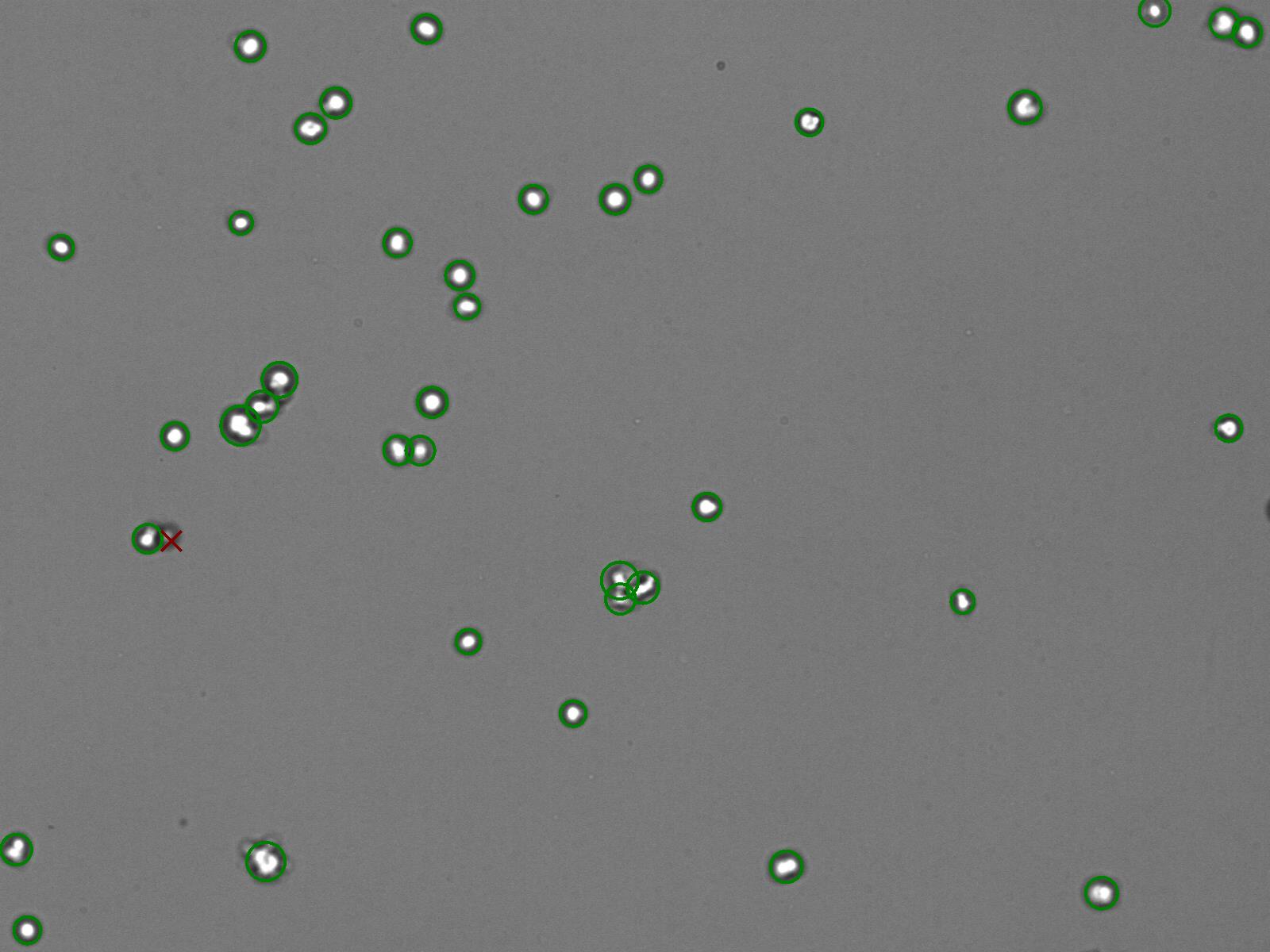

Supplement: Supplementary file 1 — Supplementary Information 1. [file 41598_2020_80576_MOESM1_ESM.zip › S1/Aggregate counts/day5/0mmHg Feb1 47 46/ML C3-003_2019-02-11_153017.bmp]

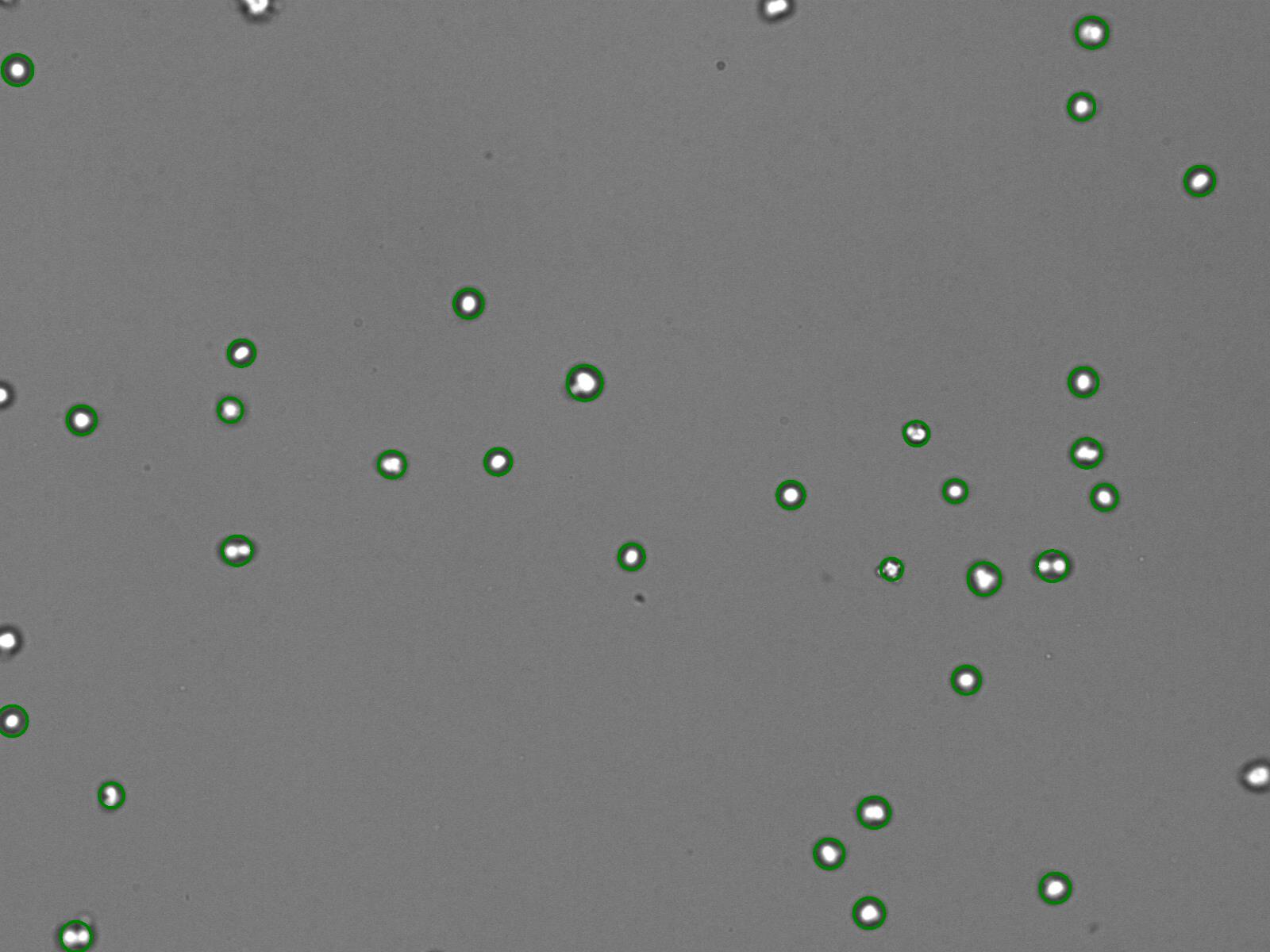

Supplement: Supplementary file 1 — Supplementary Information 1. [file 41598_2020_80576_MOESM1_ESM.zip › S1/Aggregate counts/day5/0mmHg Feb1 47 46/ML C3-004_2019-02-11_153017.bmp]

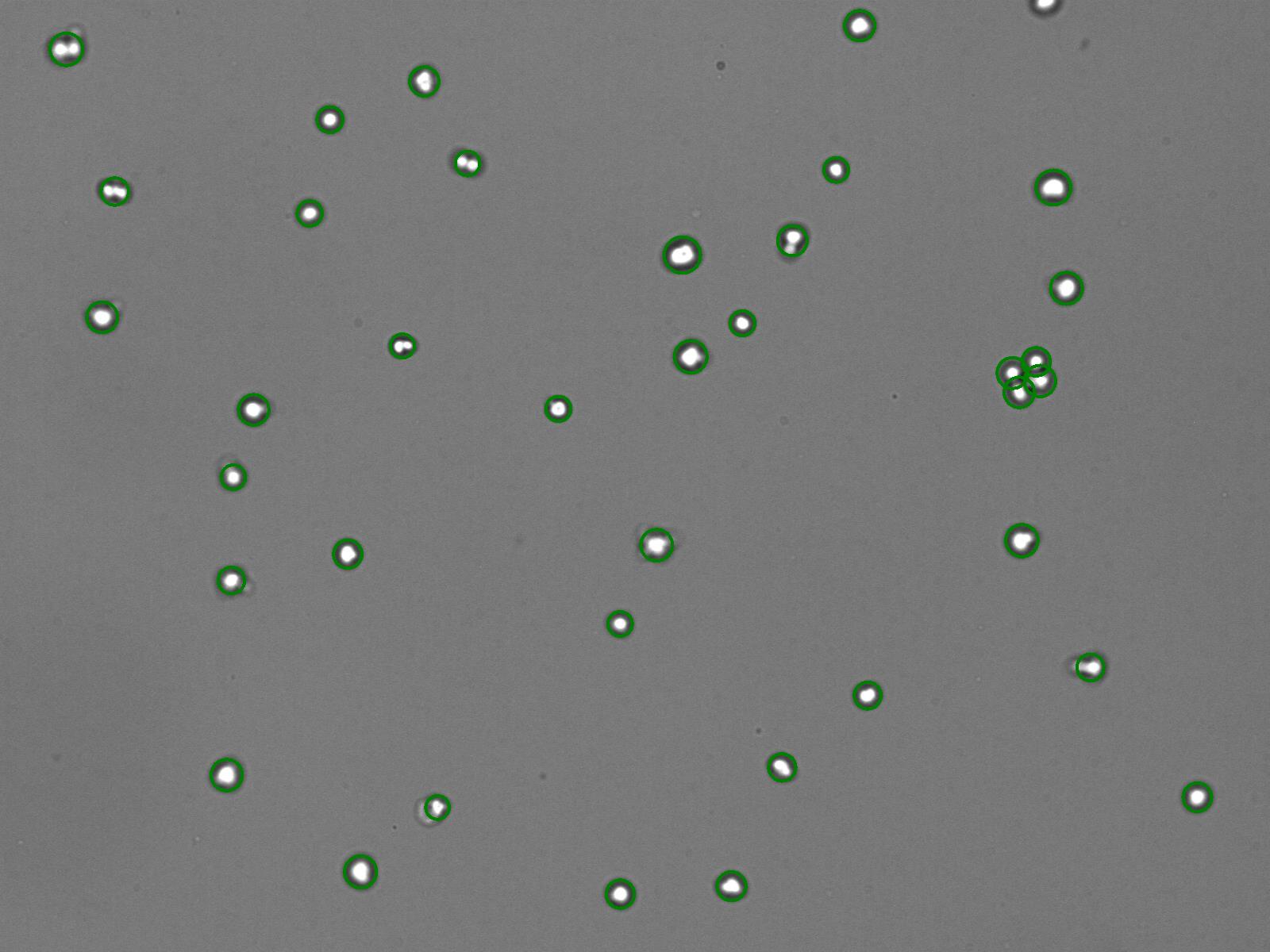

Supplement: Supplementary file 1 — Supplementary Information 1. [file 41598_2020_80576_MOESM1_ESM.zip › S1/Aggregate counts/day5/0mmHg Feb1 47 46/ML C3-005_2019-02-11_153017.bmp]

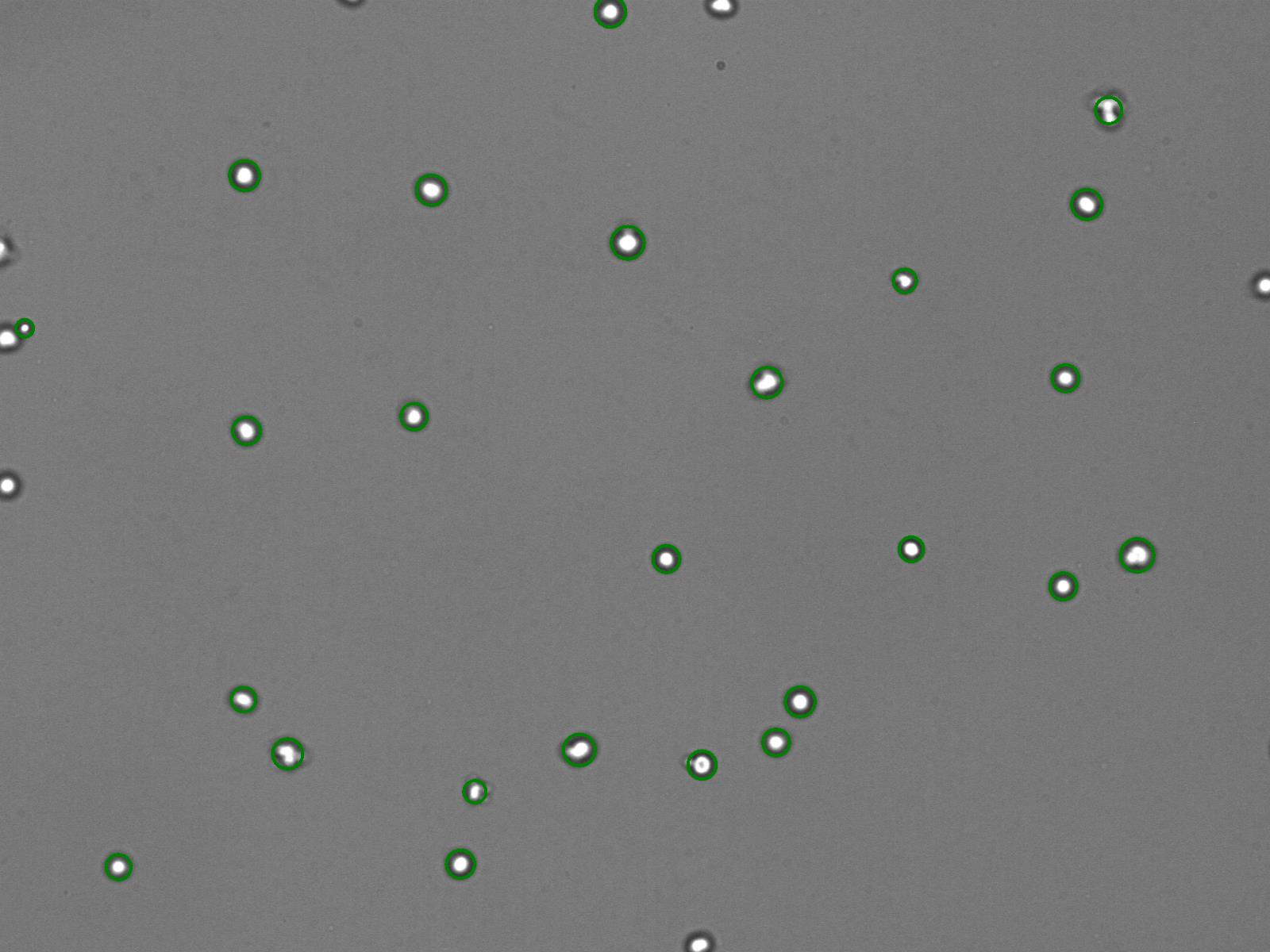

Supplement: Supplementary file 1 — Supplementary Information 1. [file 41598_2020_80576_MOESM1_ESM.zip › S1/Aggregate counts/day5/0mmHg Feb1 47 46/ML C3-006_2019-02-11_153018.bmp]

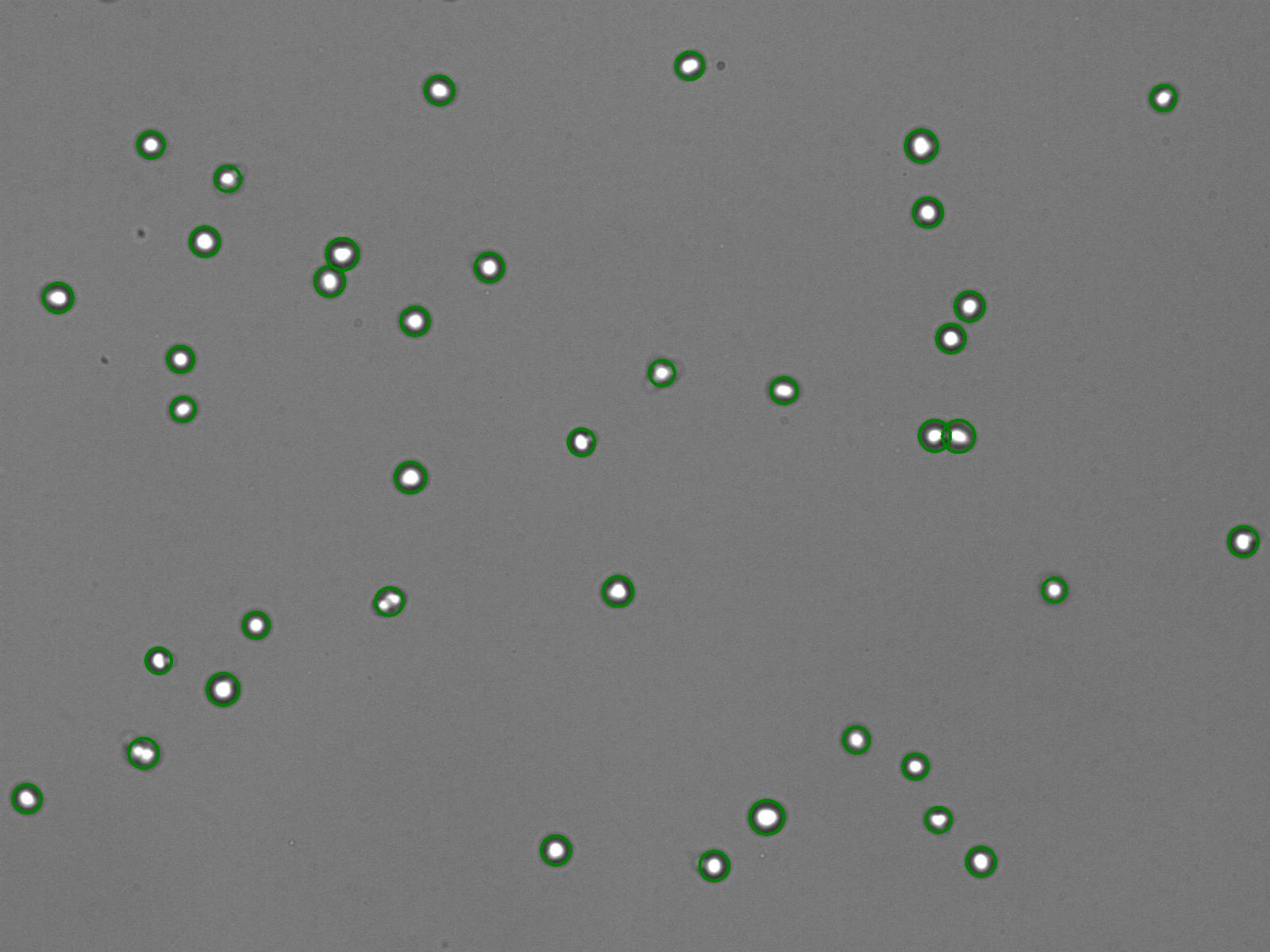

Supplement: Supplementary file 1 — Supplementary Information 1. [file 41598_2020_80576_MOESM1_ESM.zip › S1/Aggregate counts/day5/0mmHg Feb1 47 46/ML C3-007_2019-02-11_153018.bmp]

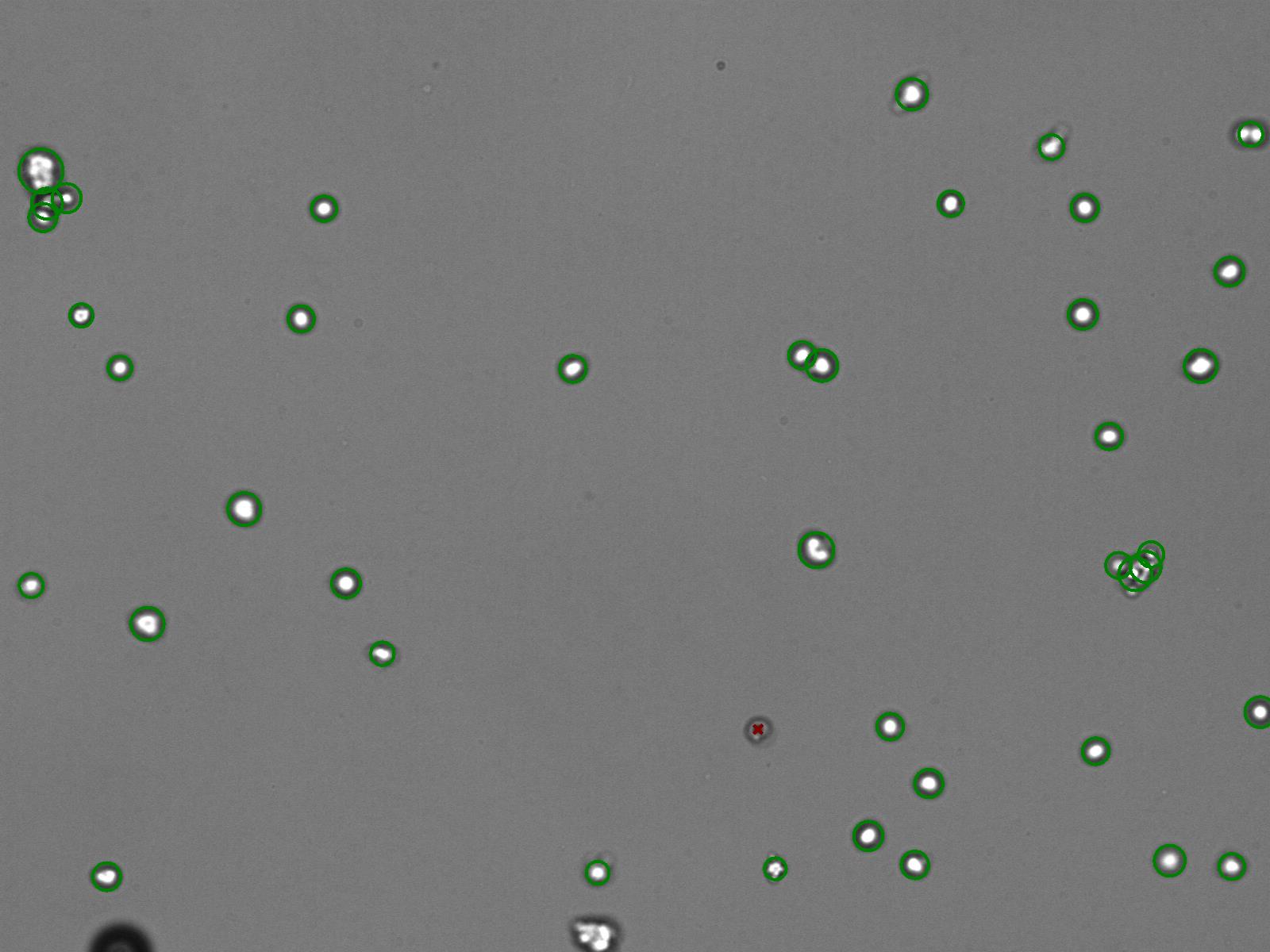

Supplement: Supplementary file 1 — Supplementary Information 1. [file 41598_2020_80576_MOESM1_ESM.zip › S1/Aggregate counts/day5/0mmHg Feb1 47 46/ML C3-008_2019-02-11_153018.bmp]

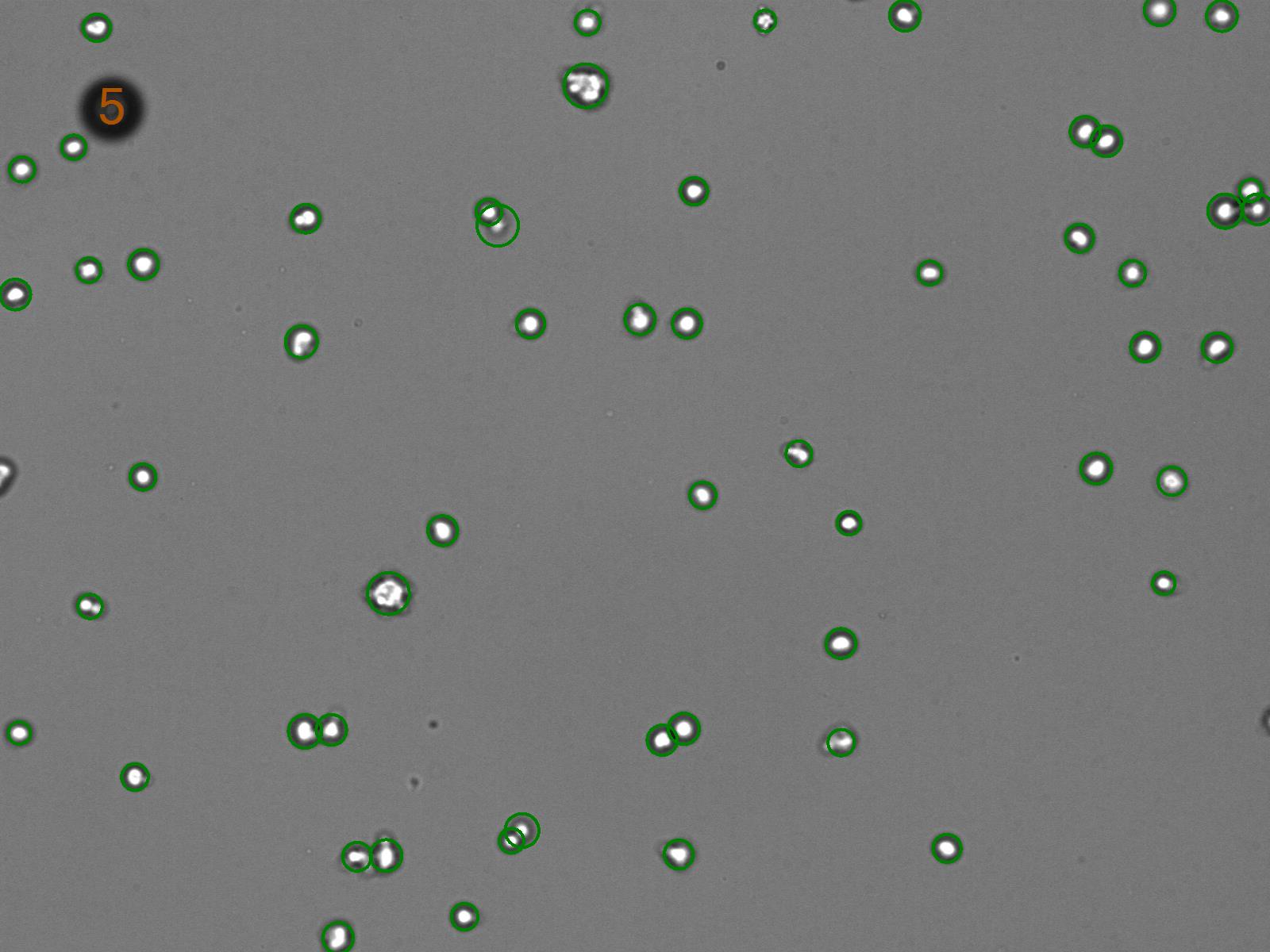

Supplement: Supplementary file 1 — Supplementary Information 1. [file 41598_2020_80576_MOESM1_ESM.zip › S1/Aggregate counts/day5/0mmHg Feb1 47 46/ML C3-009_2019-02-11_153019.bmp]

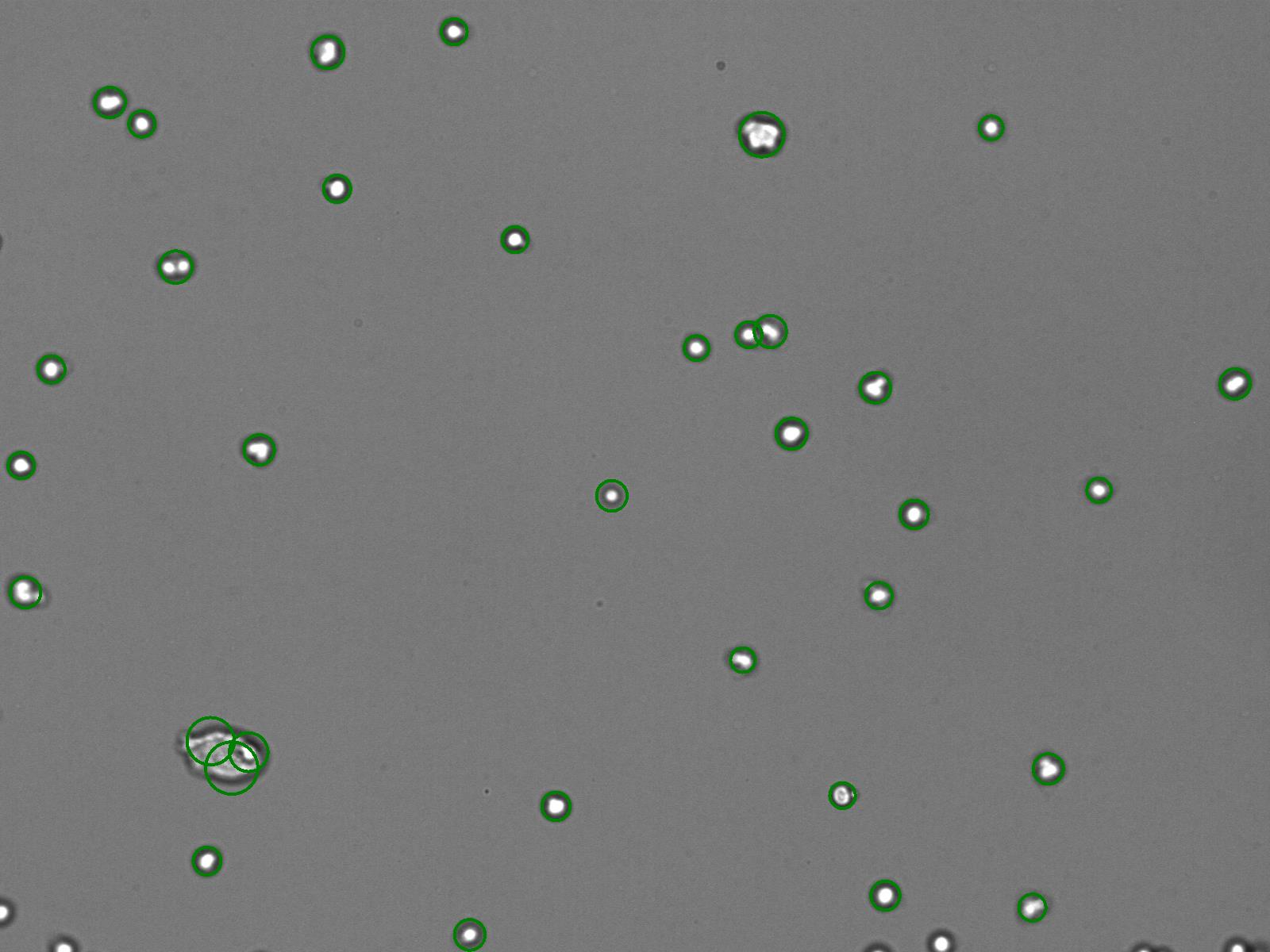

Supplement: Supplementary file 1 — Supplementary Information 1. [file 41598_2020_80576_MOESM1_ESM.zip › S1/Aggregate counts/day5/0mmHg Feb1 47 46/ML C3-010_2019-02-11_153019.bmp]

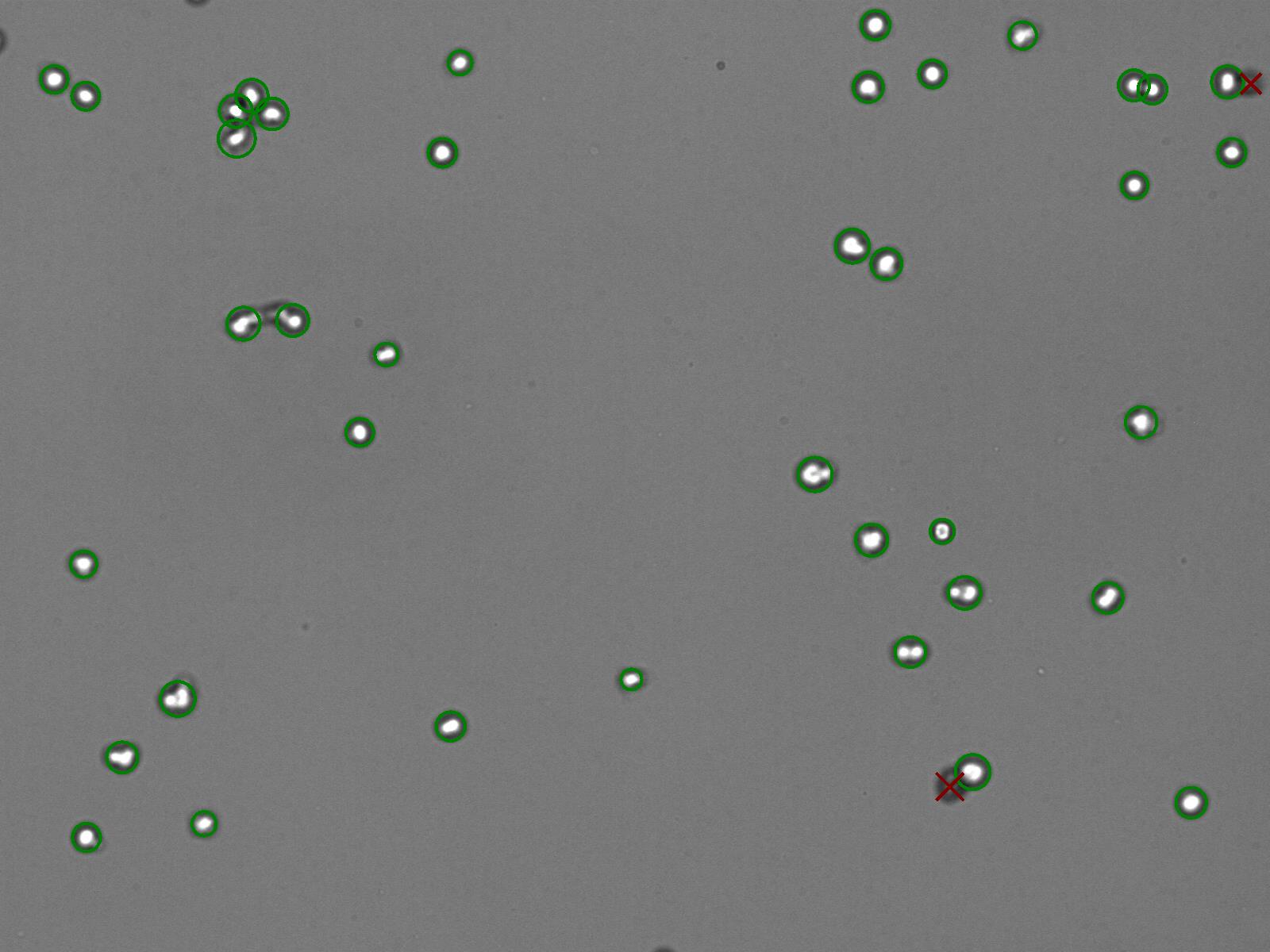

Supplement: Supplementary file 1 — Supplementary Information 1. [file 41598_2020_80576_MOESM1_ESM.zip › S1/Aggregate counts/day5/0mmHg Feb1 47 46/ML C3-011_2019-02-11_153019.bmp]

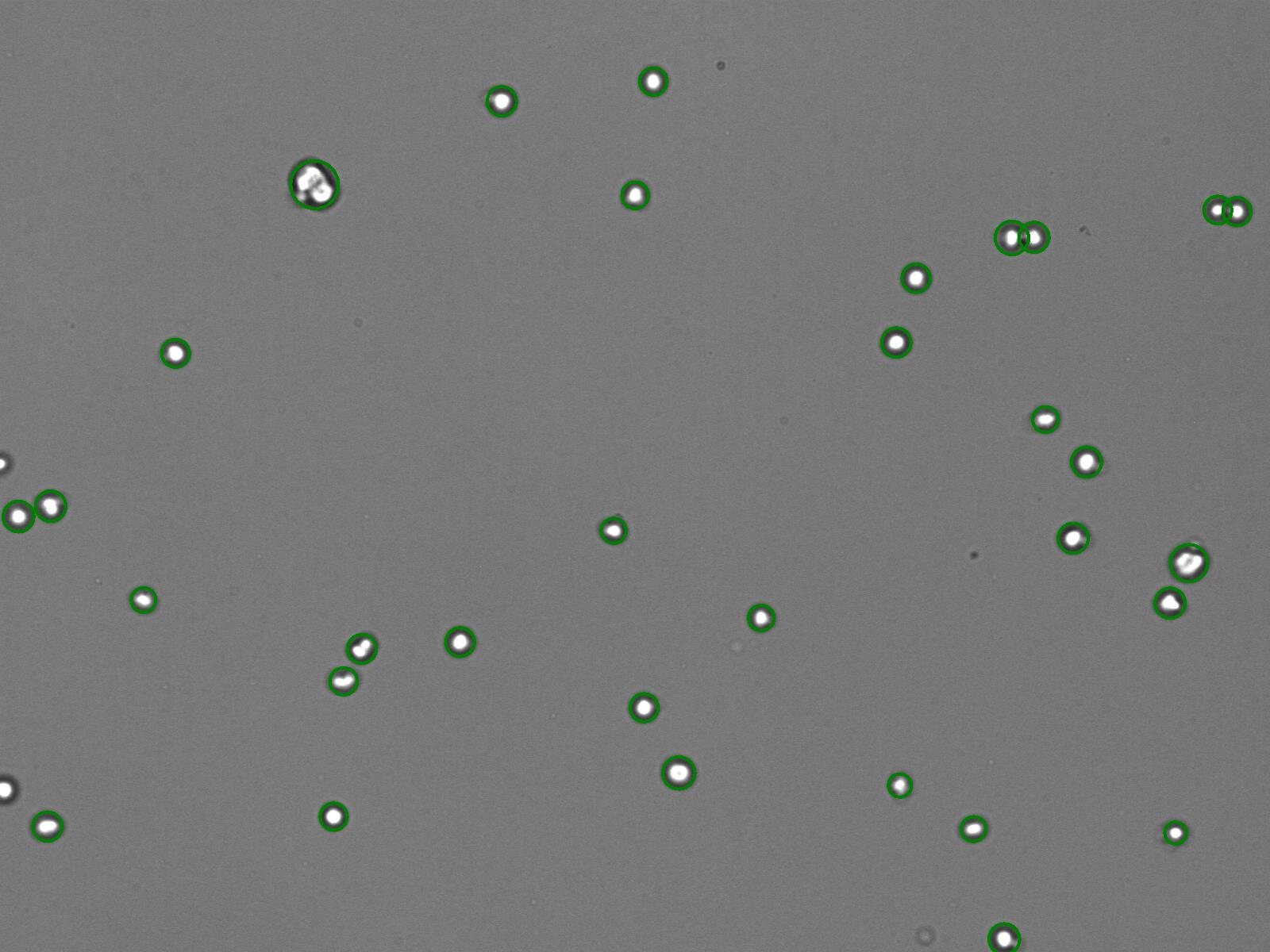

Supplement: Supplementary file 1 — Supplementary Information 1. [file 41598_2020_80576_MOESM1_ESM.zip › S1/Aggregate counts/day5/0mmHg Feb1 47 46/ML C3-012_2019-02-11_153020.bmp]

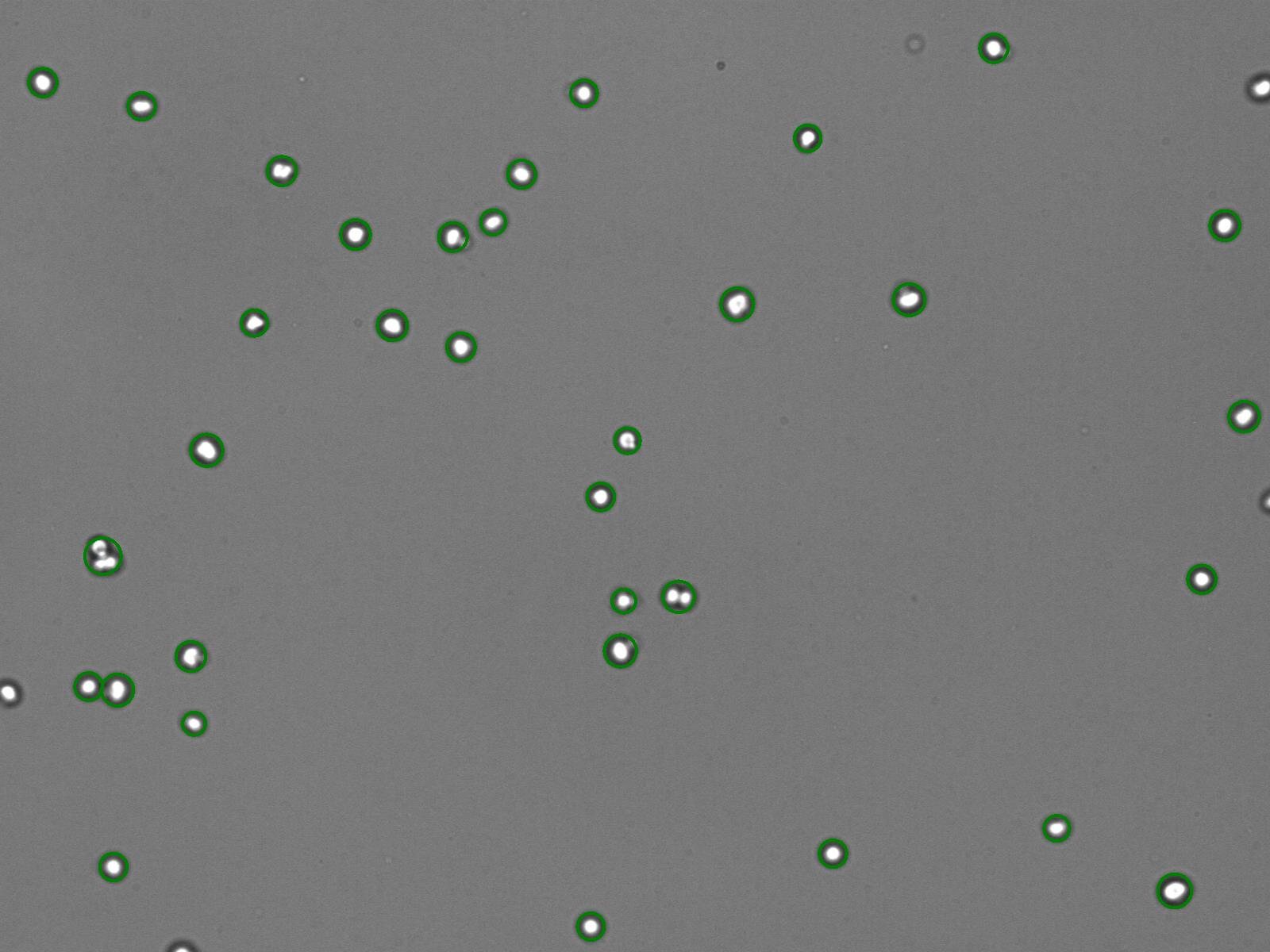

Supplement: Supplementary file 1 — Supplementary Information 1. [file 41598_2020_80576_MOESM1_ESM.zip › S1/Aggregate counts/day5/0mmHg Feb1 47 46/ML C3-013_2019-02-11_153020.bmp]

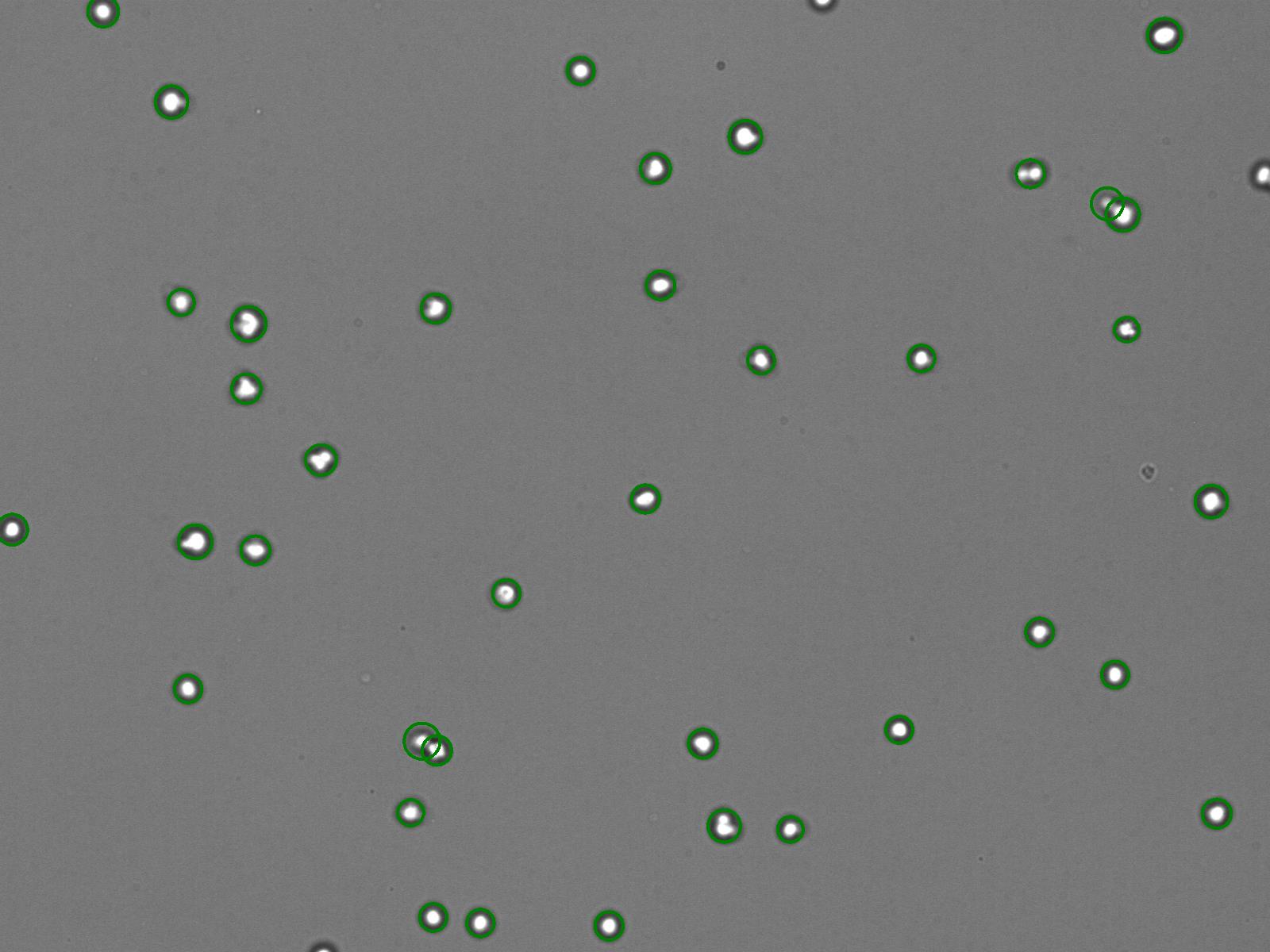

Supplement: Supplementary file 1 — Supplementary Information 1. [file 41598_2020_80576_MOESM1_ESM.zip › S1/Aggregate counts/day5/0mmHg Feb1 47 46/ML C3-014_2019-02-11_153020.bmp]

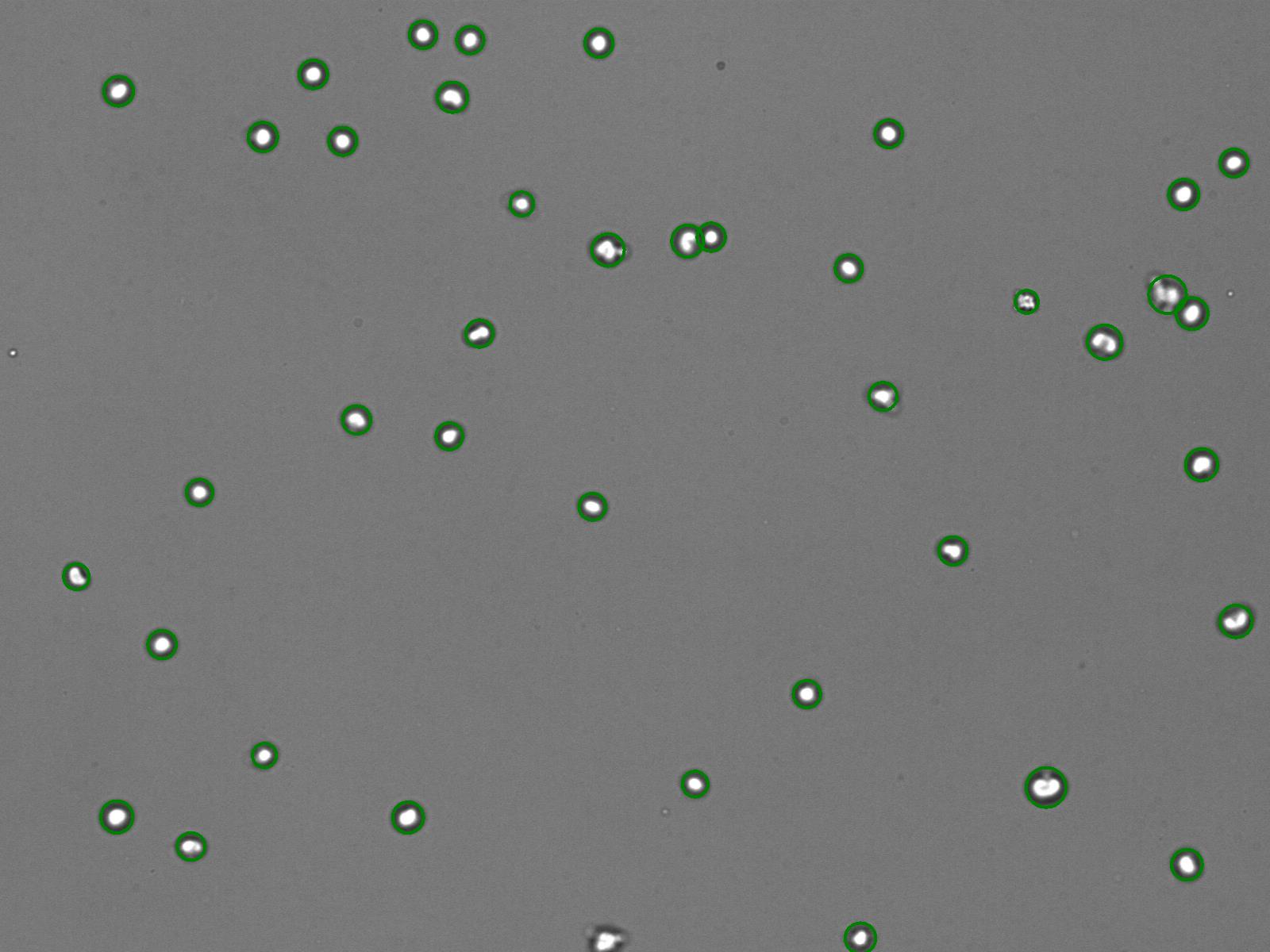

Supplement: Supplementary file 1 — Supplementary Information 1. [file 41598_2020_80576_MOESM1_ESM.zip › S1/Aggregate counts/day5/0mmHg Feb1 47 46/ML C3-015_2019-02-11_153021.bmp]

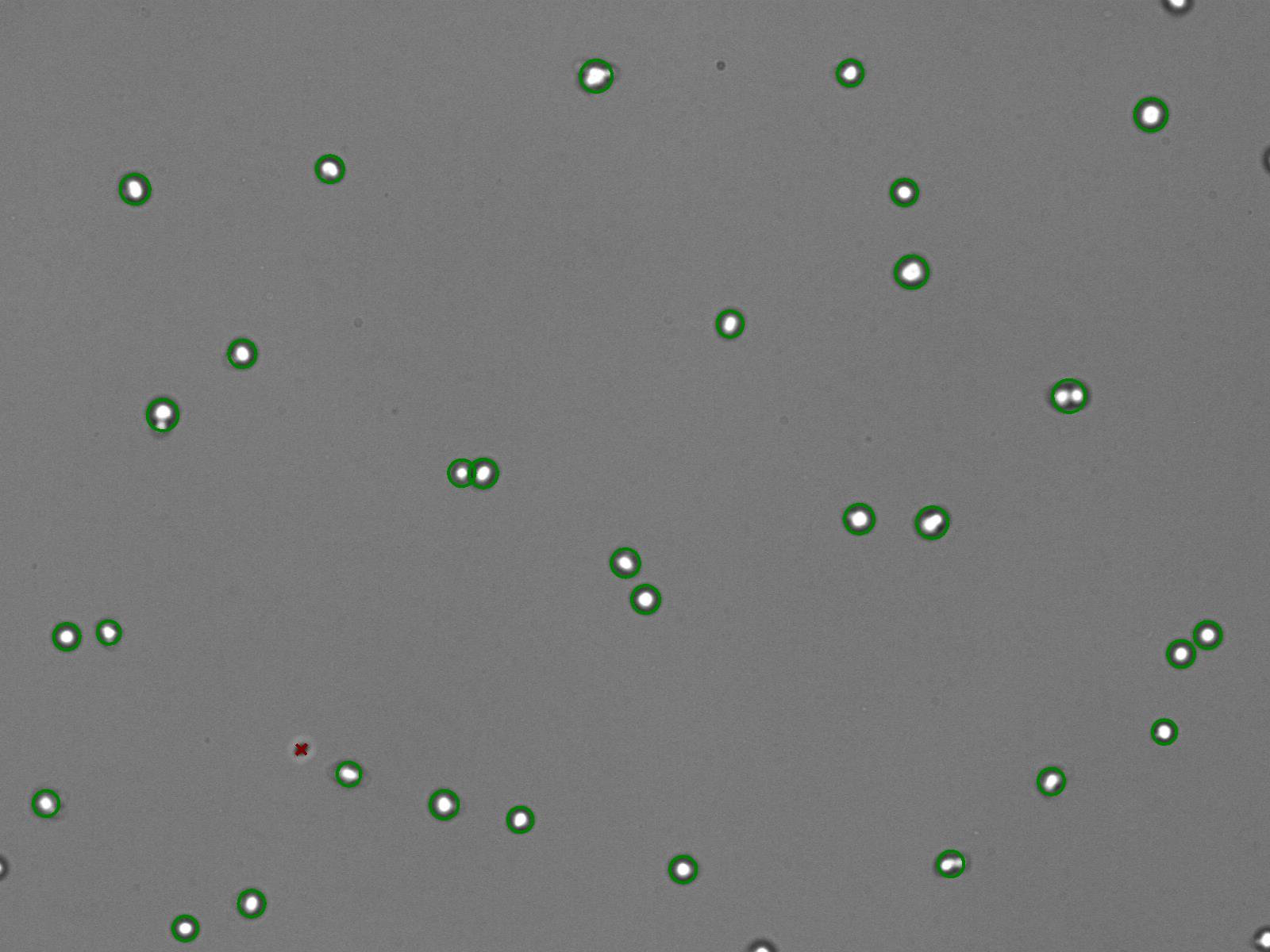

Supplement: Supplementary file 1 — Supplementary Information 1. [file 41598_2020_80576_MOESM1_ESM.zip › S1/Aggregate counts/day5/0mmHg Feb1 47 46/ML C3-016_2019-02-11_153021.bmp]

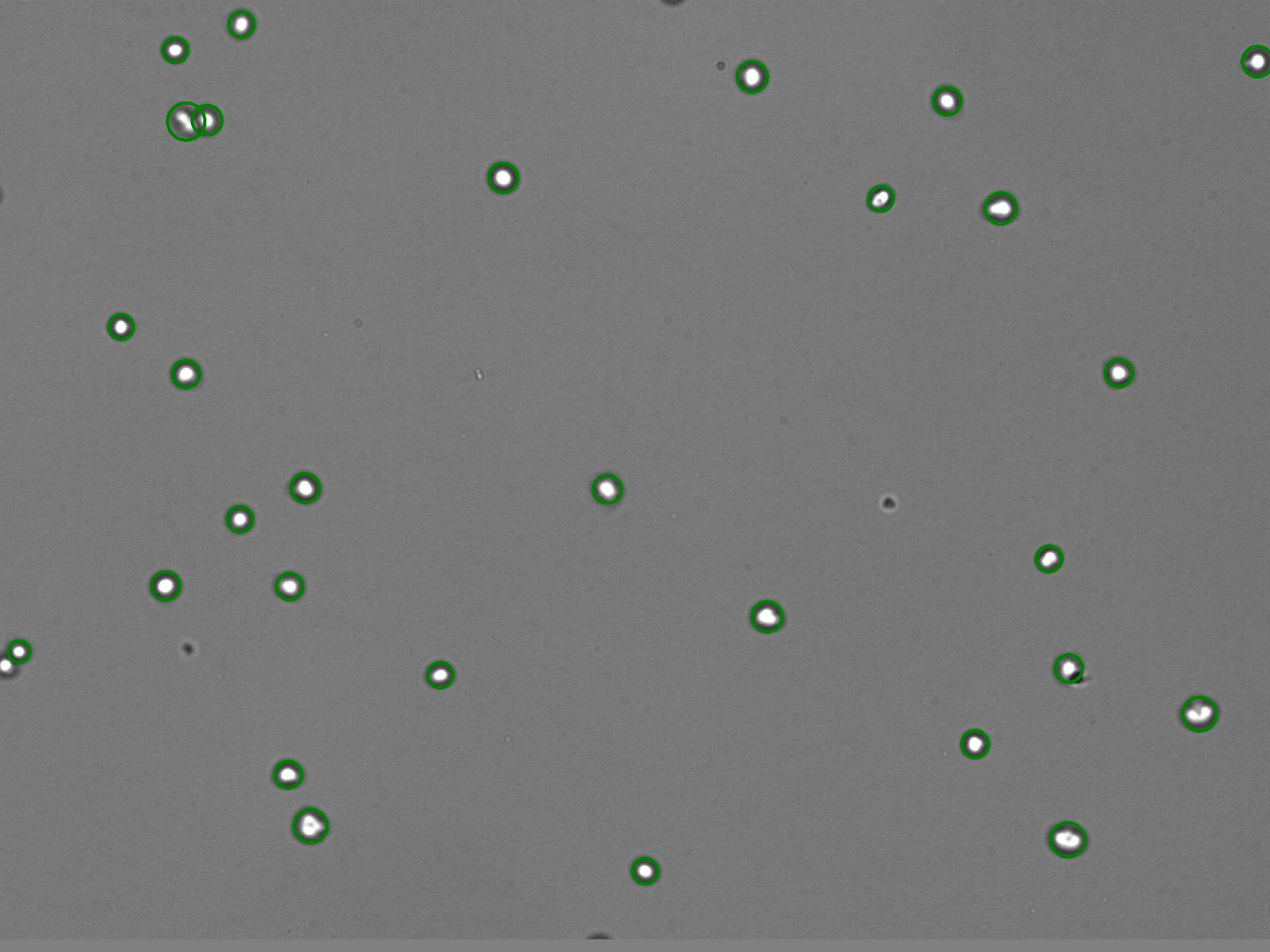

Supplement: Supplementary file 1 — Supplementary Information 1. [file 41598_2020_80576_MOESM1_ESM.zip › S1/Aggregate counts/day5/0mmHg Feb1 47 46/ML C3-017_2019-02-11_153021.bmp]

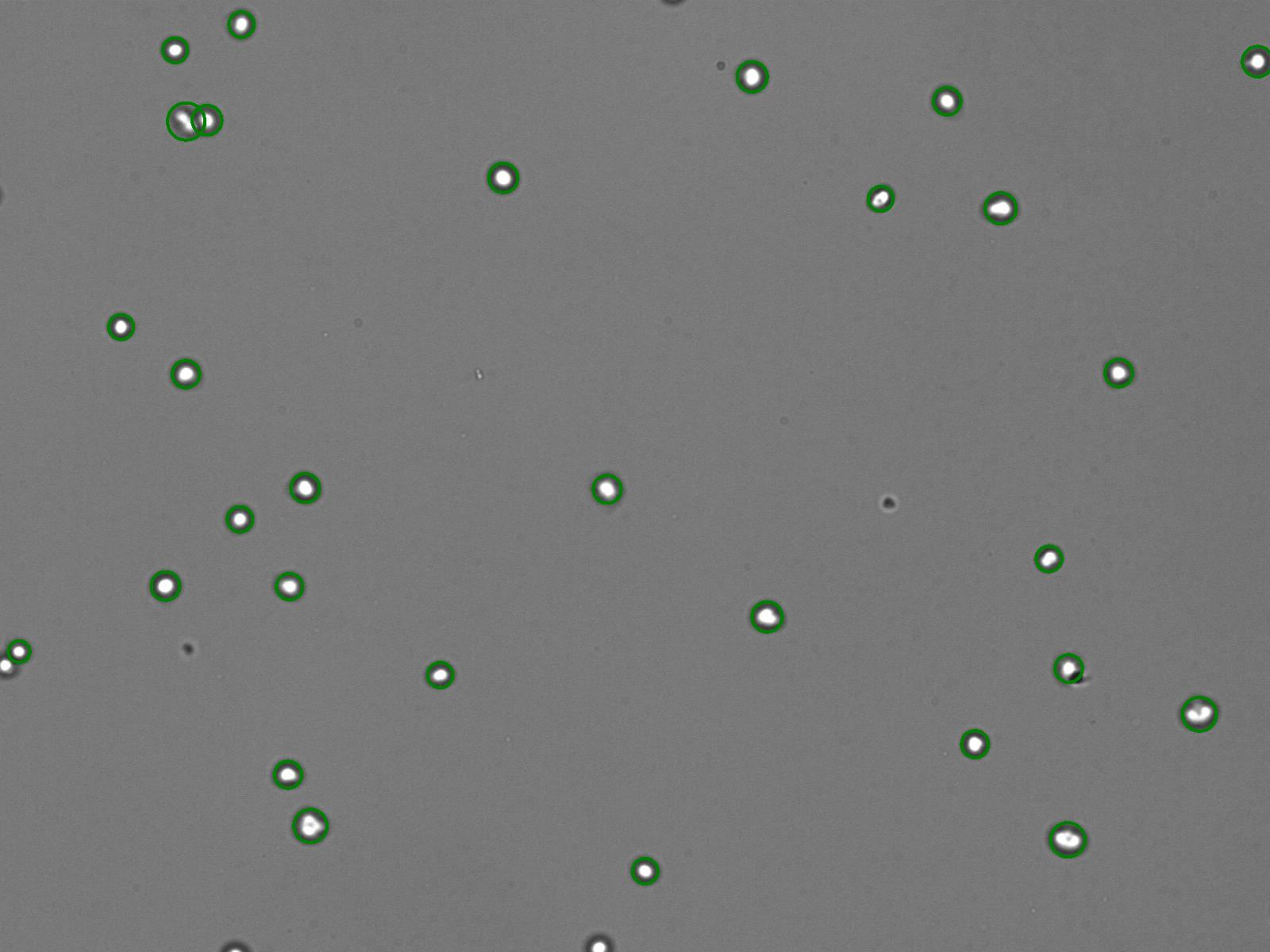

Supplement: Supplementary file 1 — Supplementary Information 1. [file 41598_2020_80576_MOESM1_ESM.zip › S1/Aggregate counts/day5/0mmHg Feb1 47 46/ML C3-017_2019-02-17_162617.bmp]

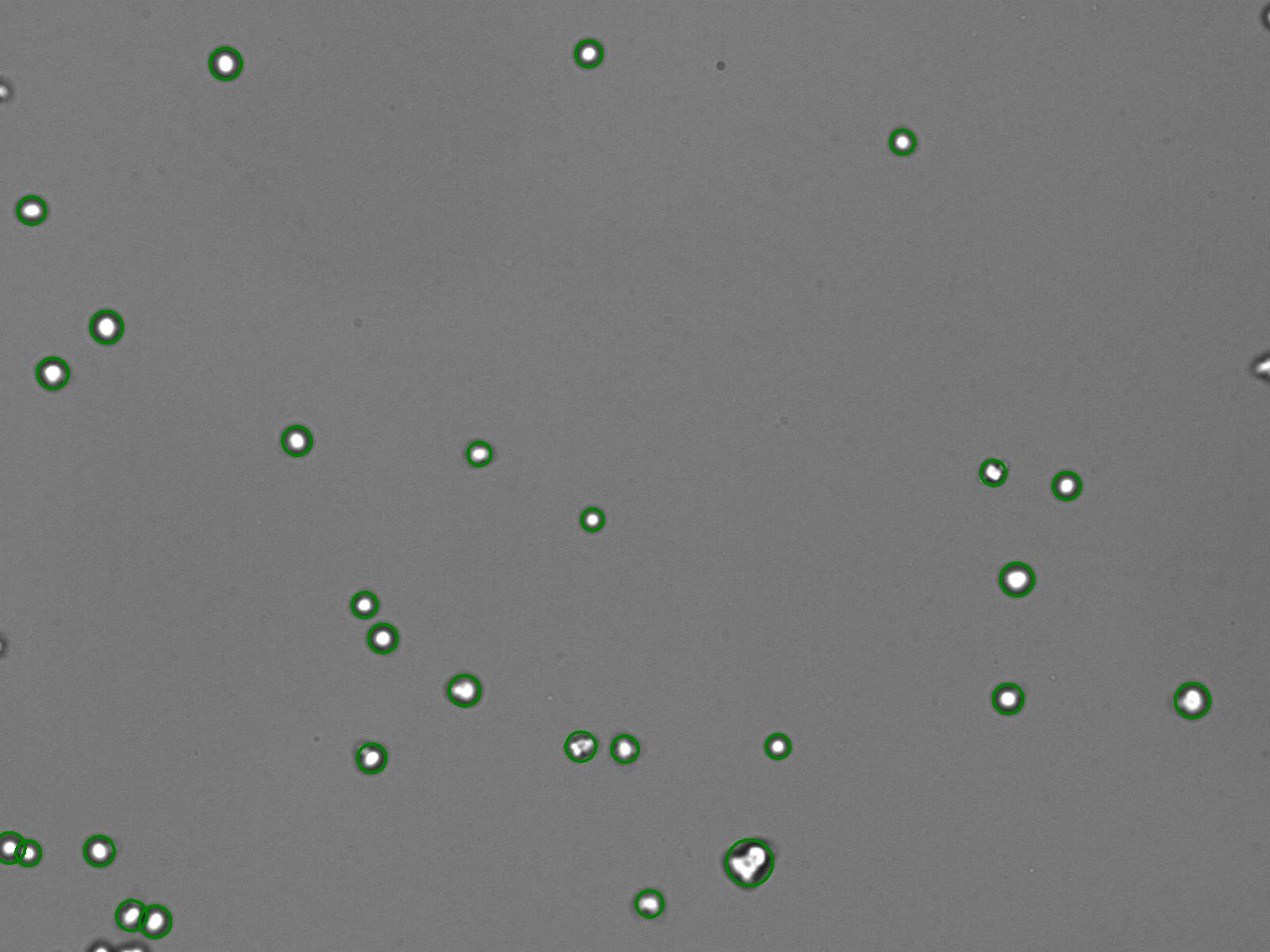

Supplement: Supplementary file 1 — Supplementary Information 1. [file 41598_2020_80576_MOESM1_ESM.zip › S1/Aggregate counts/day5/0mmHg Feb1 47 46/ML C3-018_2019-02-17_162617.bmp]

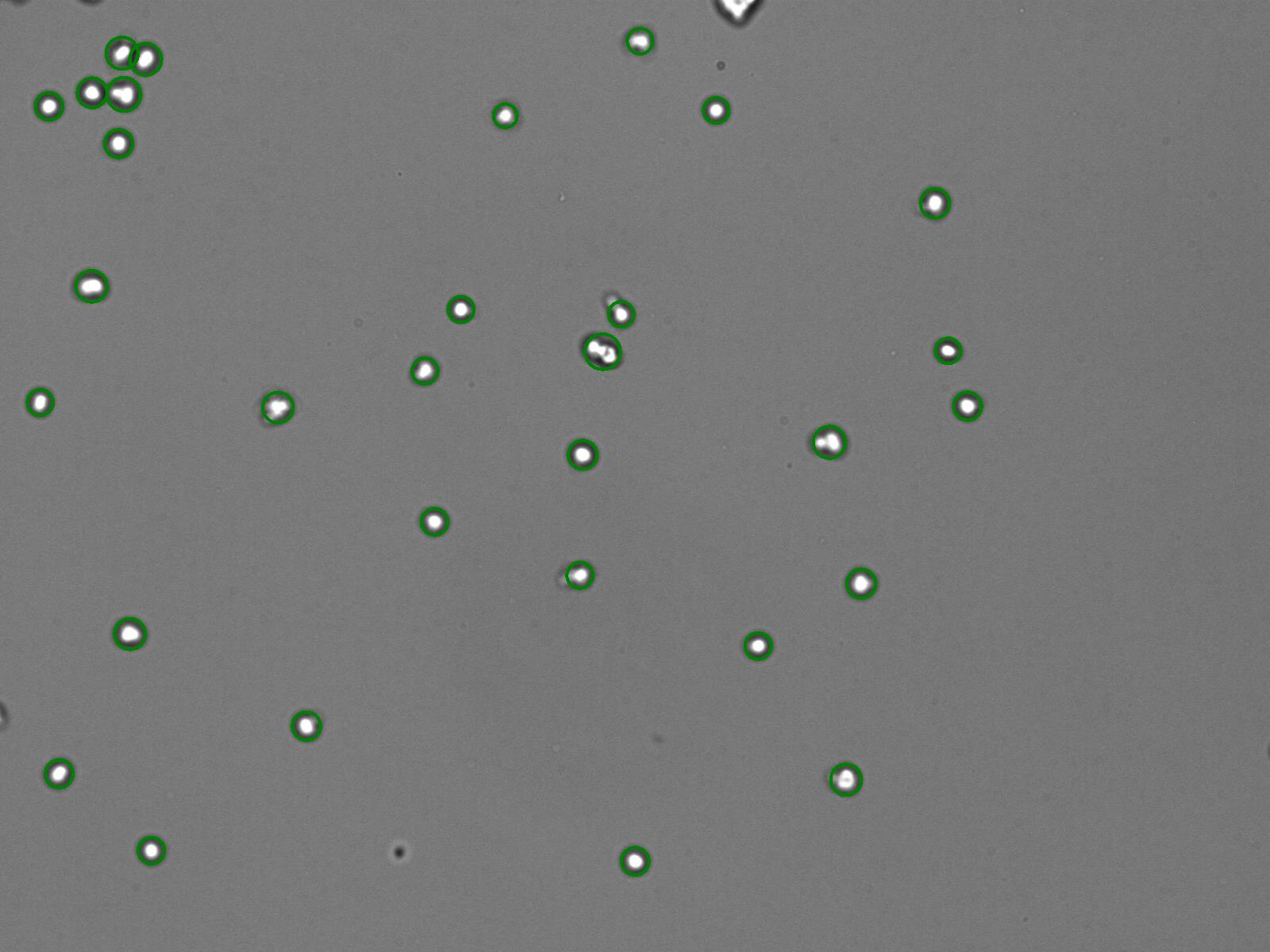

Supplement: Supplementary file 1 — Supplementary Information 1. [file 41598_2020_80576_MOESM1_ESM.zip › S1/Aggregate counts/day5/0mmHg Feb1 47 46/ML C3-019_2019-02-17_162618.bmp]

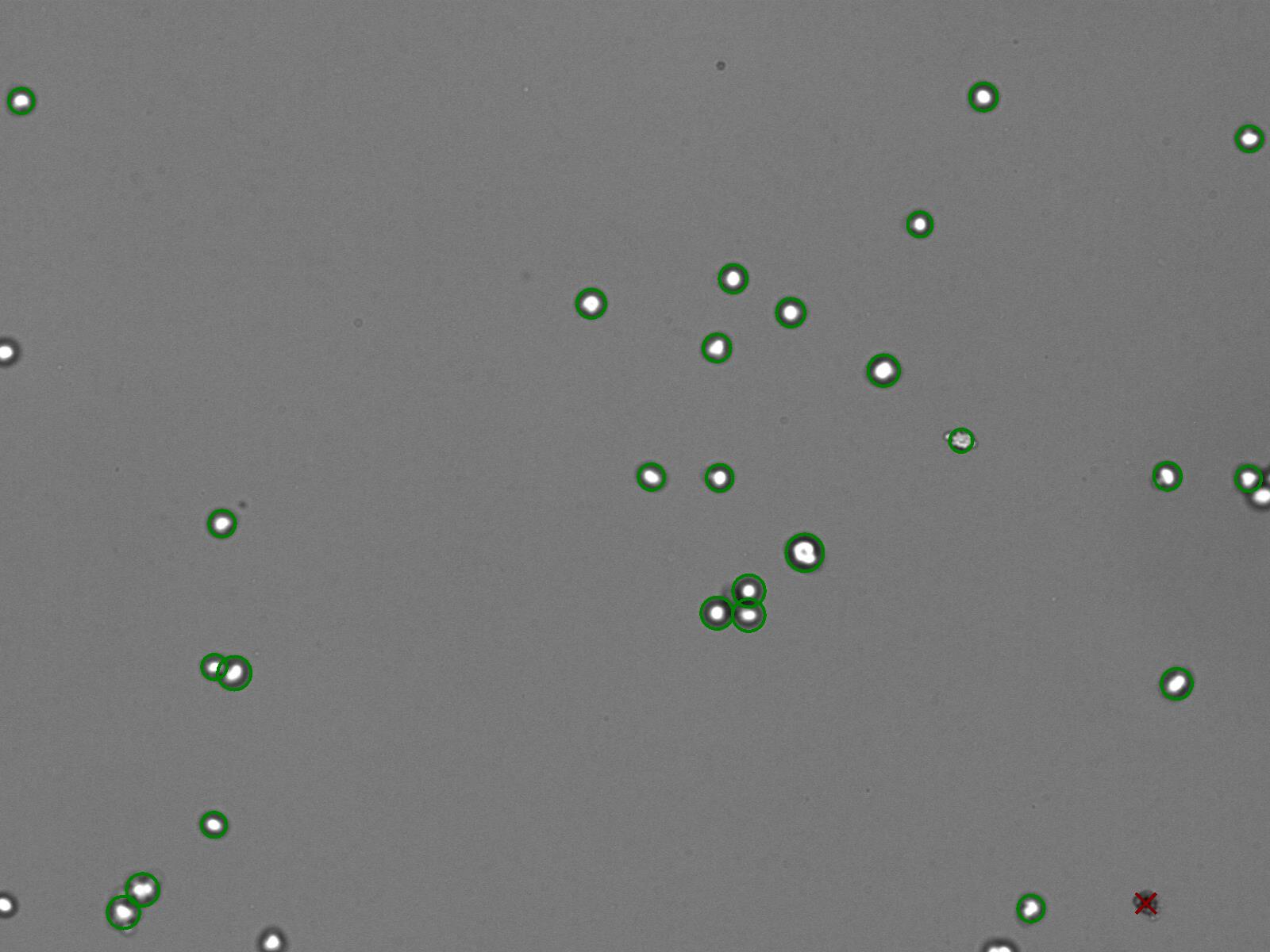

Supplement: Supplementary file 1 — Supplementary Information 1. [file 41598_2020_80576_MOESM1_ESM.zip › S1/Aggregate counts/day5/0mmHg Feb1 47 46/ML C3-020_2019-02-17_162618.bmp]

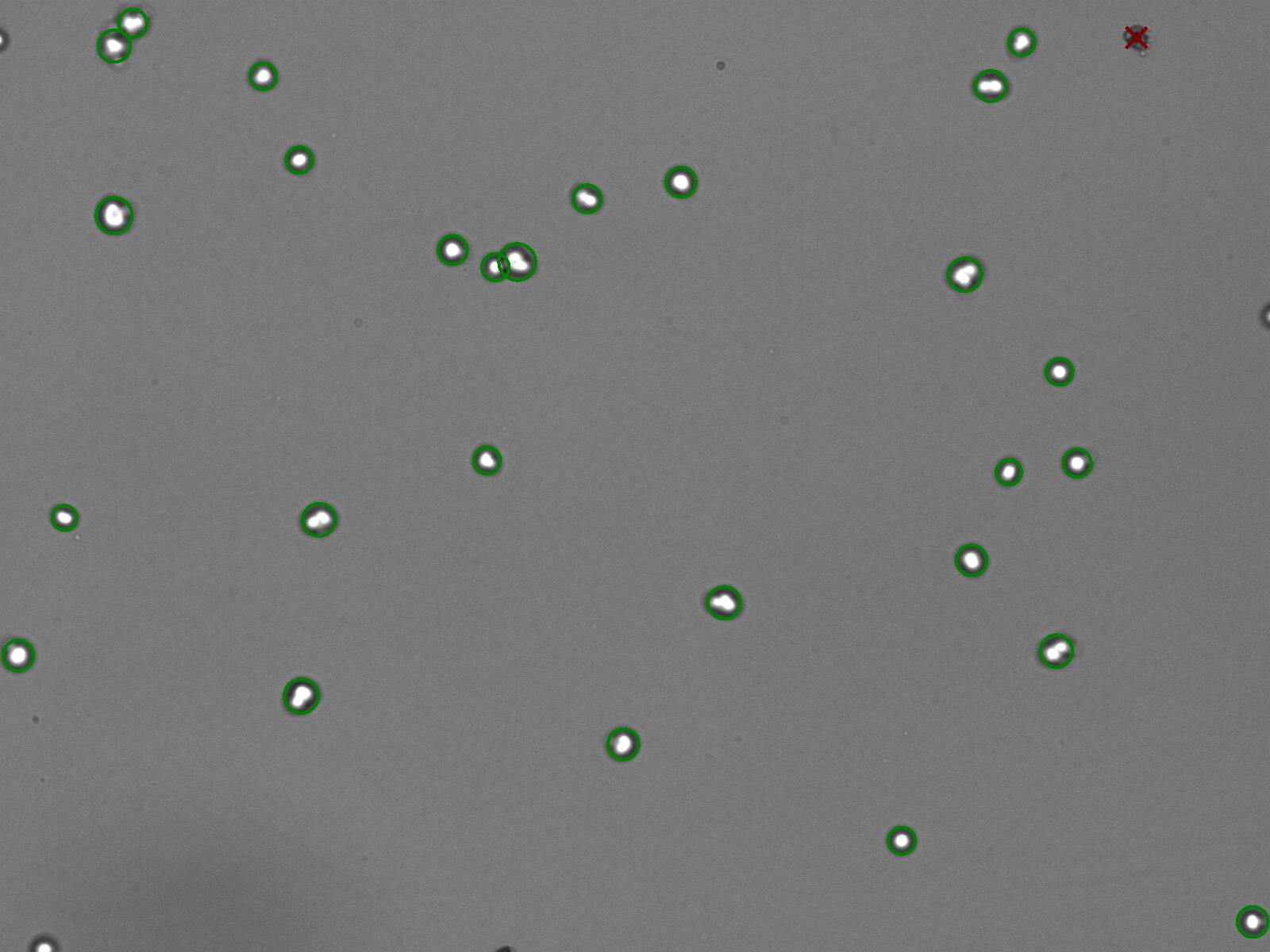

Supplement: Supplementary file 1 — Supplementary Information 1. [file 41598_2020_80576_MOESM1_ESM.zip › S1/Aggregate counts/day5/0mmHg Feb1 47 46/ML C3-021_2019-02-17_162618.bmp]

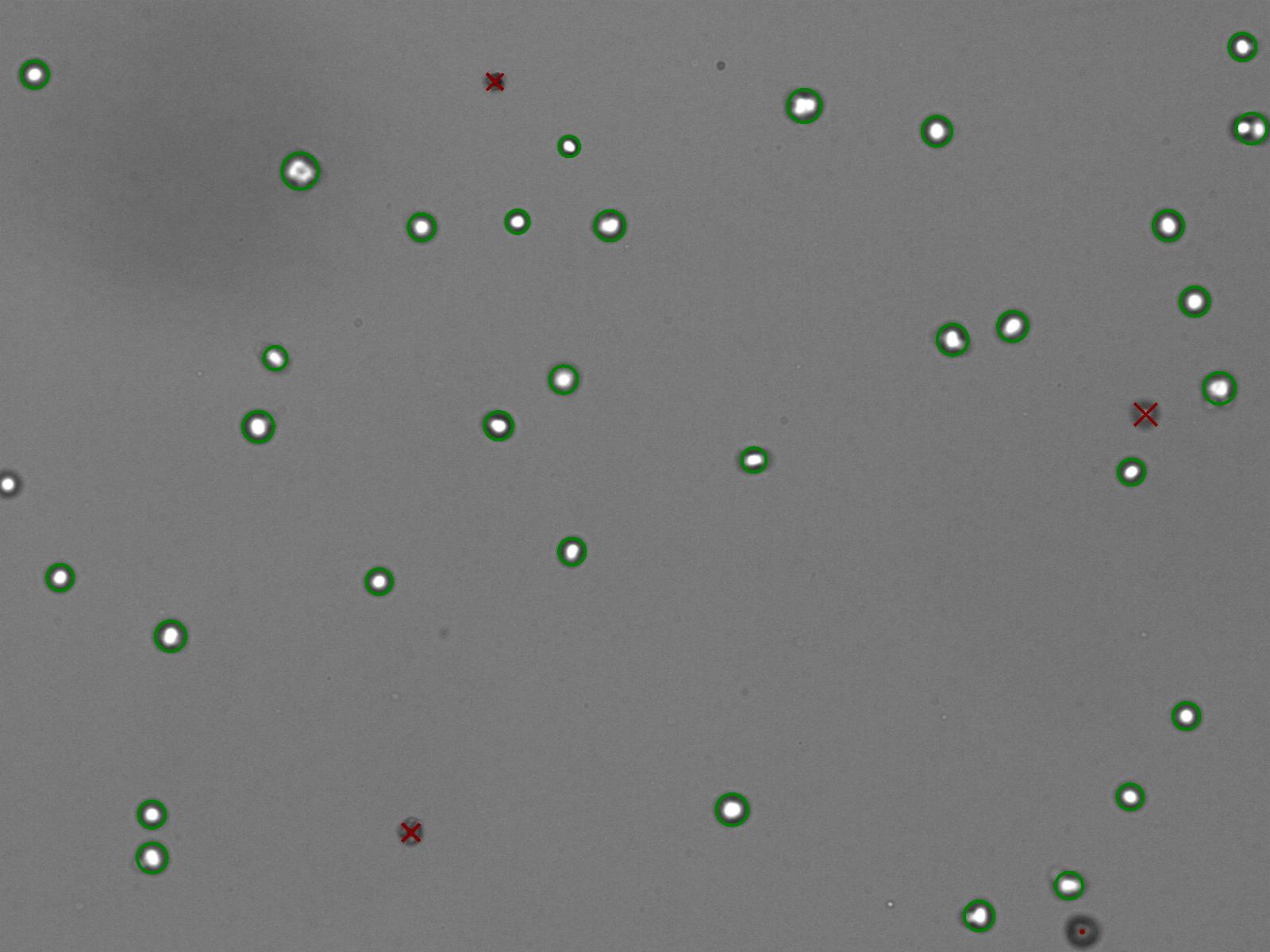

Supplement: Supplementary file 1 — Supplementary Information 1. [file 41598_2020_80576_MOESM1_ESM.zip › S1/Aggregate counts/day5/0mmHg Feb1 47 46/ML C3-022_2019-02-17_162618.bmp]

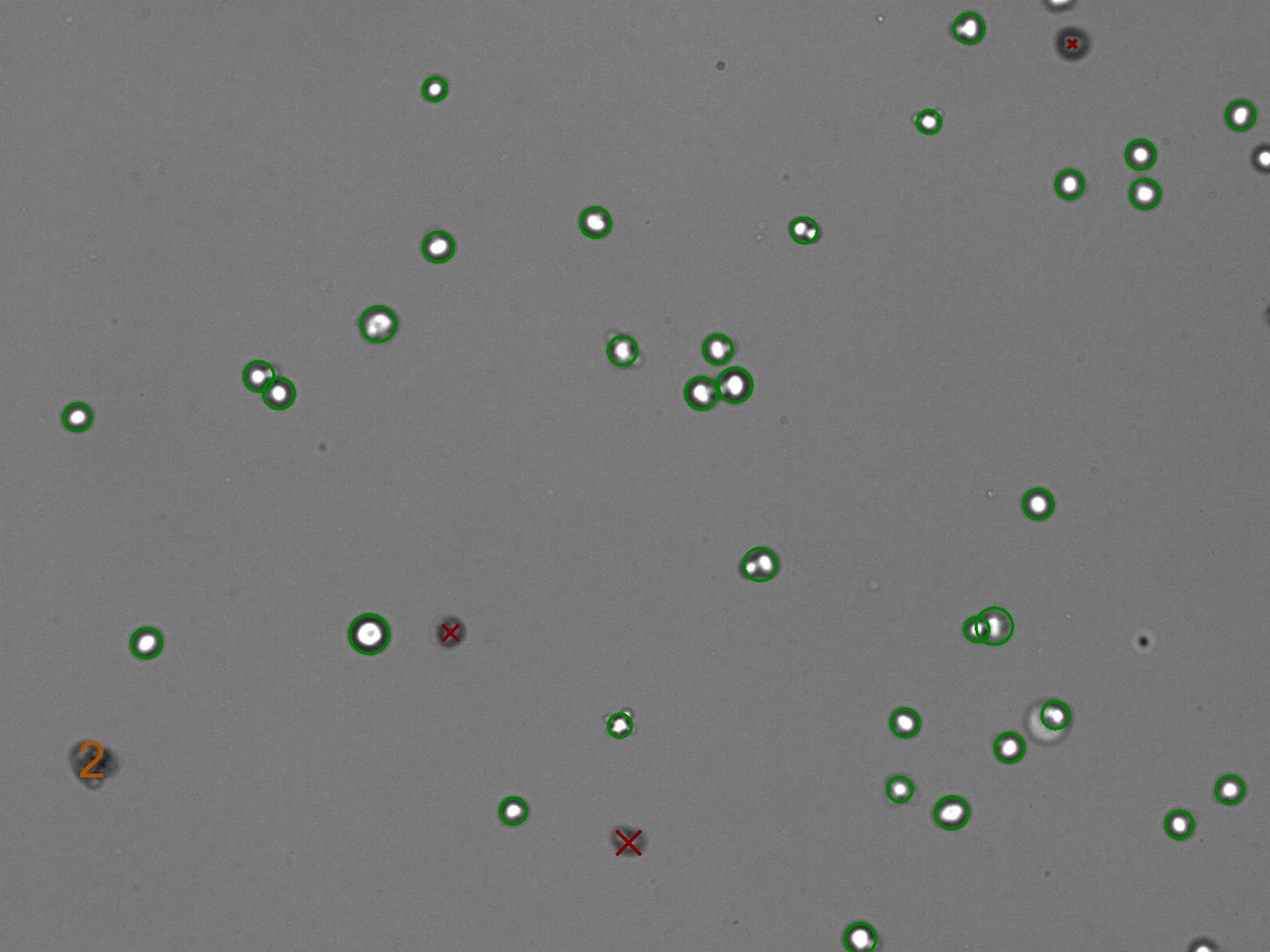

Supplement: Supplementary file 1 — Supplementary Information 1. [file 41598_2020_80576_MOESM1_ESM.zip › S1/Aggregate counts/day5/0mmHg Feb1 47 46/ML C3-023_2019-02-17_162619.bmp]

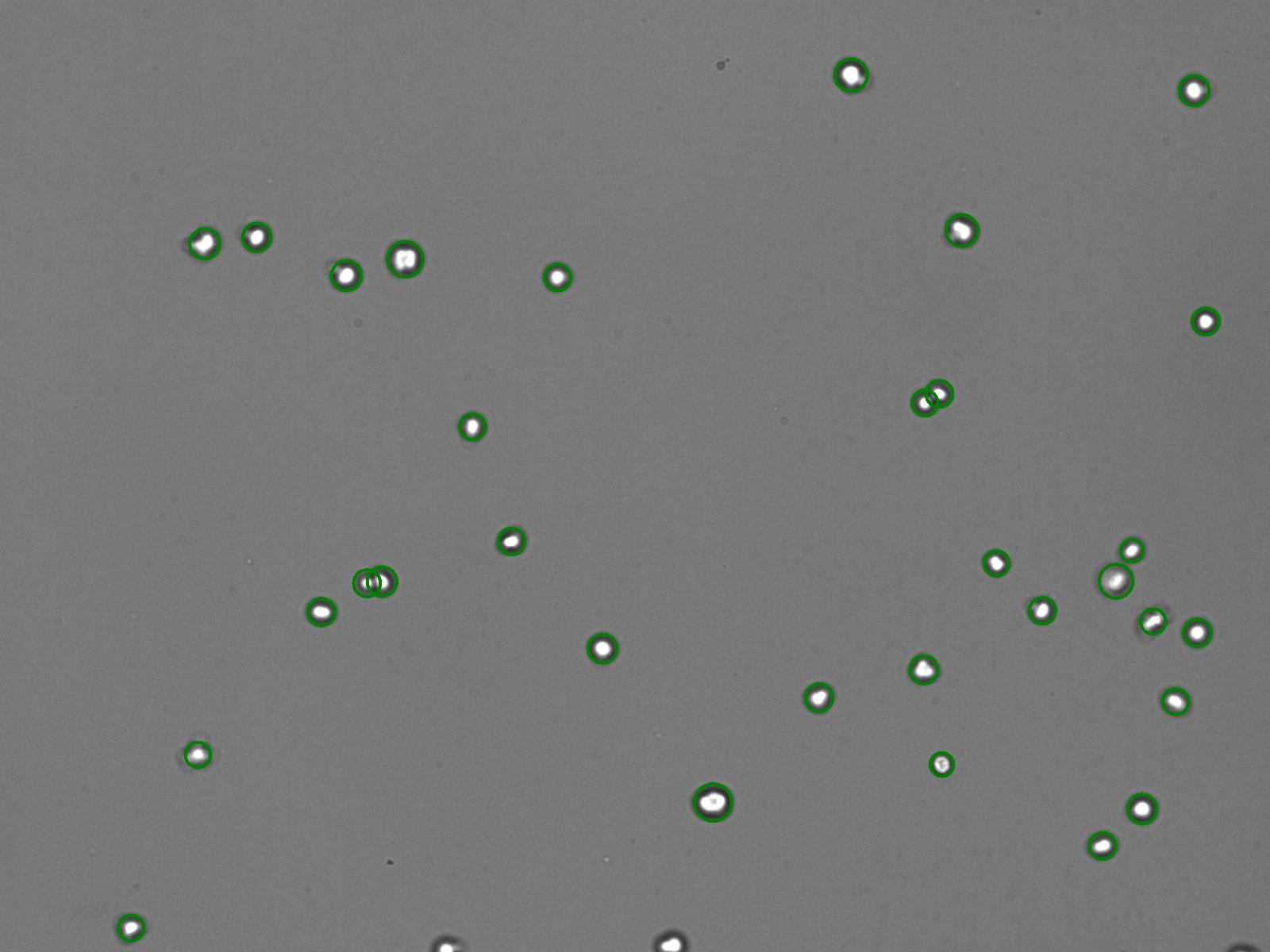

Supplement: Supplementary file 1 — Supplementary Information 1. [file 41598_2020_80576_MOESM1_ESM.zip › S1/Aggregate counts/day5/0mmHg Feb1 47 46/ML C3-024_2019-02-17_162619.bmp]

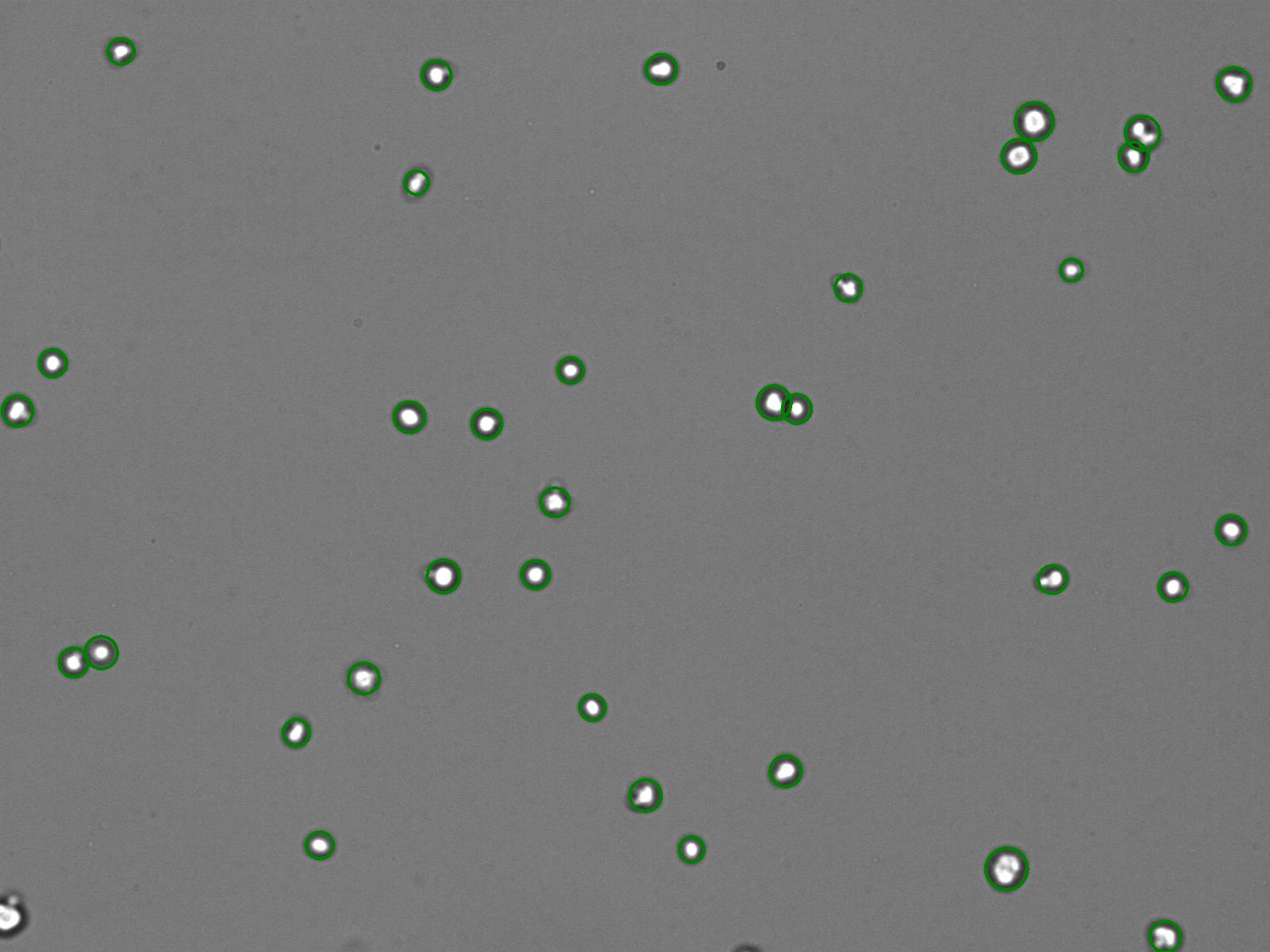

Supplement: Supplementary file 1 — Supplementary Information 1. [file 41598_2020_80576_MOESM1_ESM.zip › S1/Aggregate counts/day5/0mmHg Feb1 47 46/ML C3-025_2019-02-17_162619.bmp]

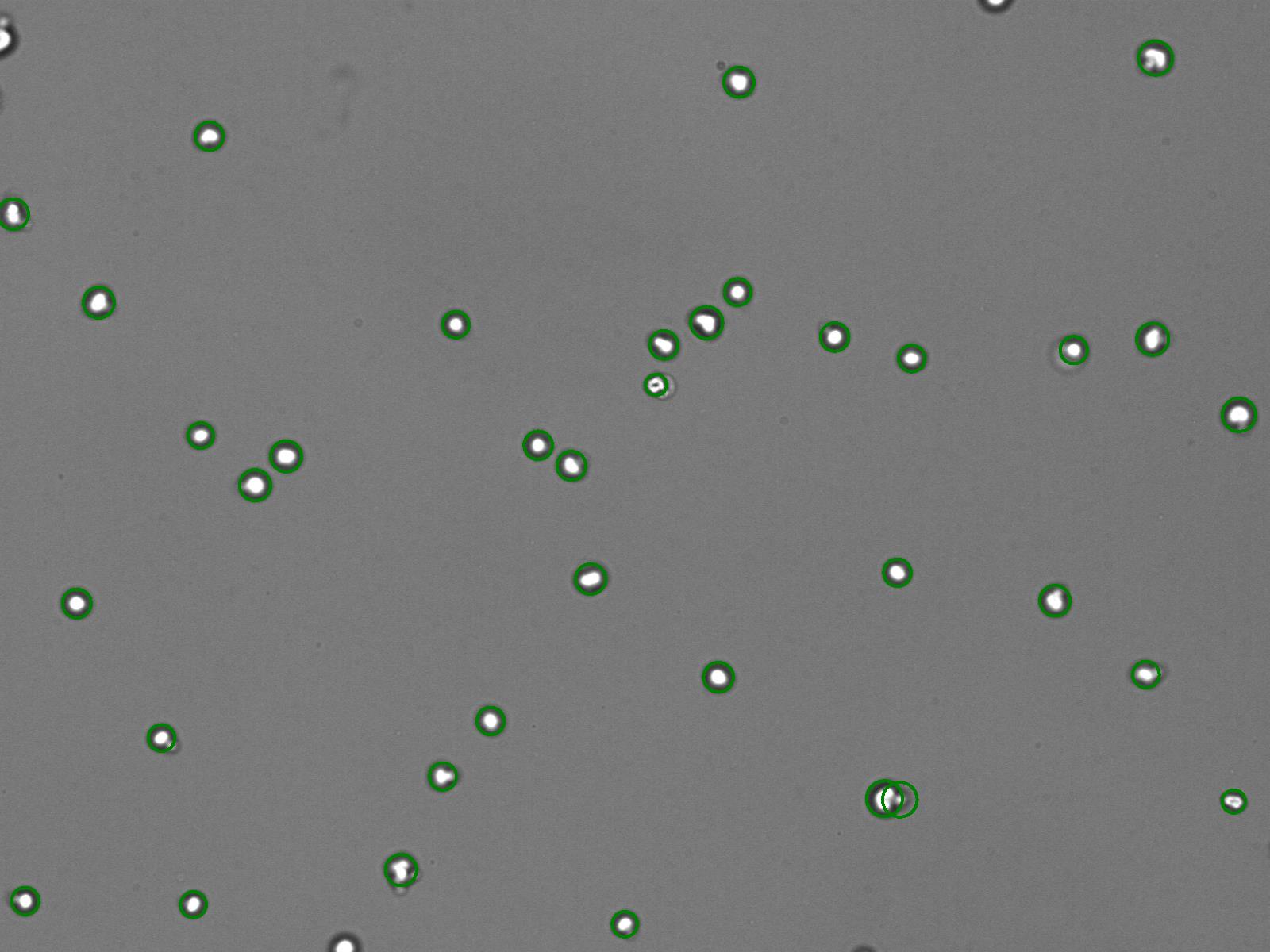

Supplement: Supplementary file 1 — Supplementary Information 1. [file 41598_2020_80576_MOESM1_ESM.zip › S1/Aggregate counts/day5/0mmHg Feb1 47 46/ML C3-026_2019-02-17_162620.bmp]

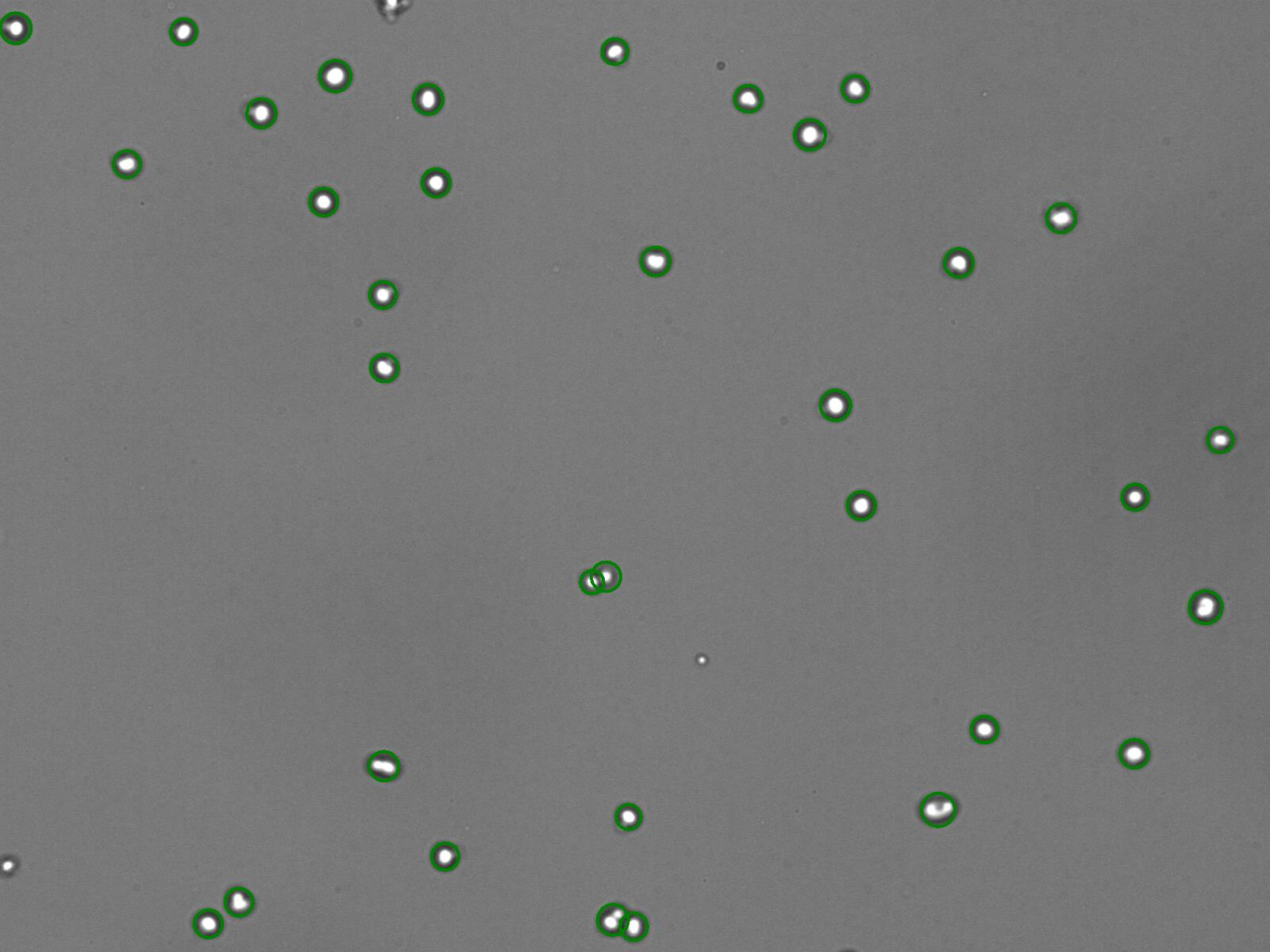

Supplement: Supplementary file 1 — Supplementary Information 1. [file 41598_2020_80576_MOESM1_ESM.zip › S1/Aggregate counts/day5/0mmHg Feb1 47 46/ML C3-027_2019-02-17_162620.bmp]

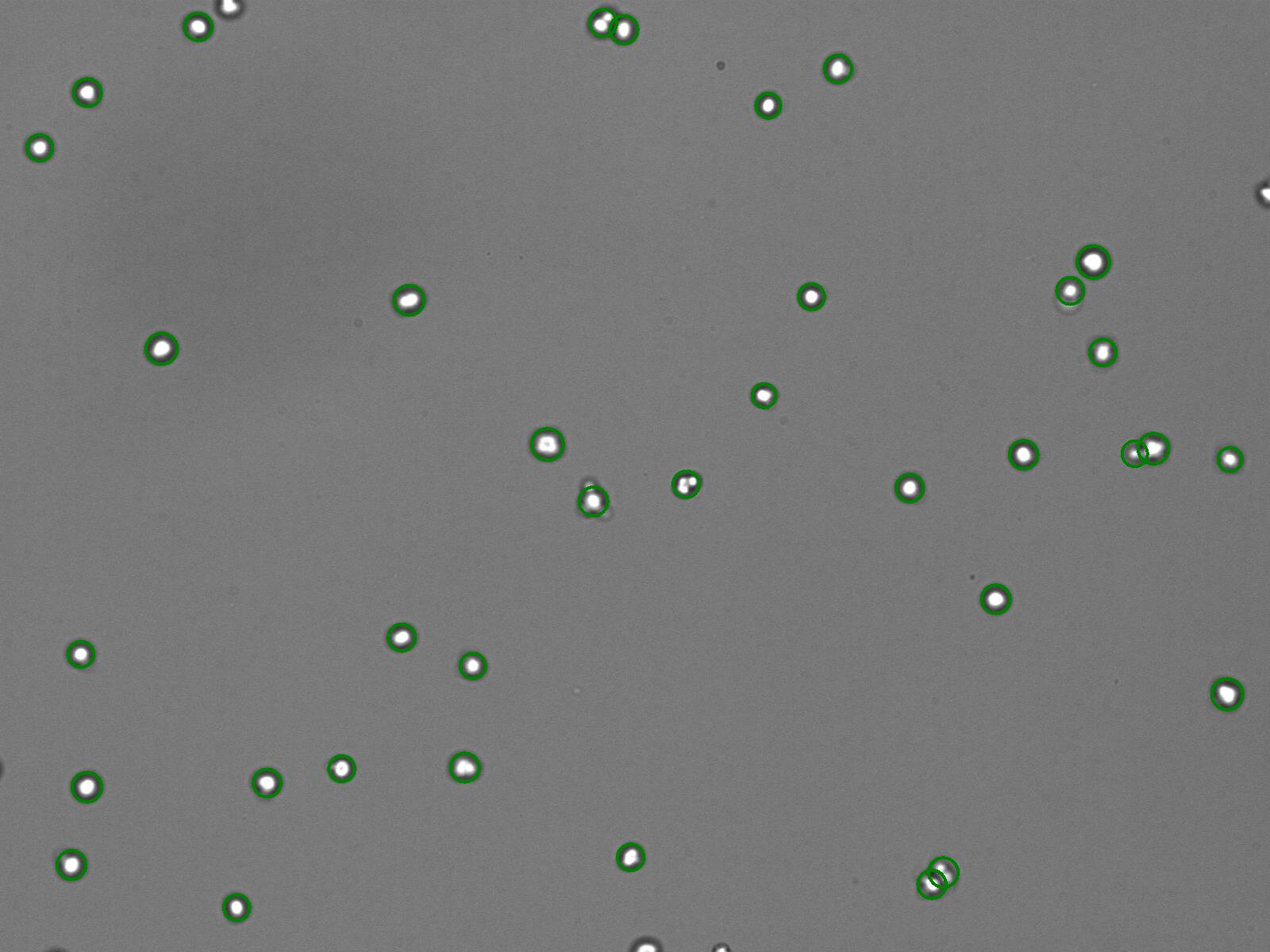

Supplement: Supplementary file 1 — Supplementary Information 1. [file 41598_2020_80576_MOESM1_ESM.zip › S1/Aggregate counts/day5/0mmHg Feb1 47 46/ML C3-028_2019-02-17_162620.bmp]

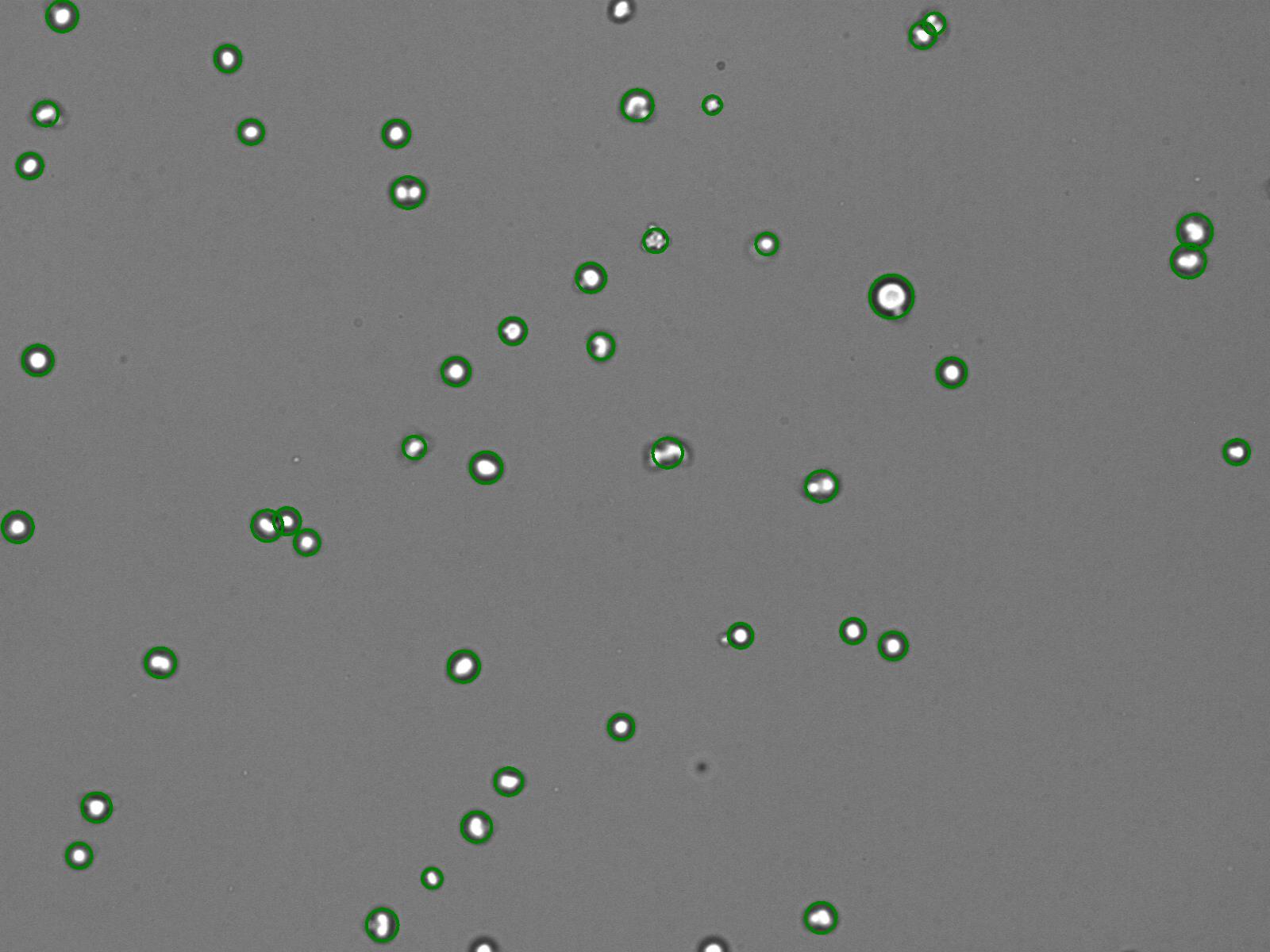

Supplement: Supplementary file 1 — Supplementary Information 1. [file 41598_2020_80576_MOESM1_ESM.zip › S1/Aggregate counts/day5/0mmHg Feb1 47 46/ML C3-029_2019-02-17_162620.bmp]

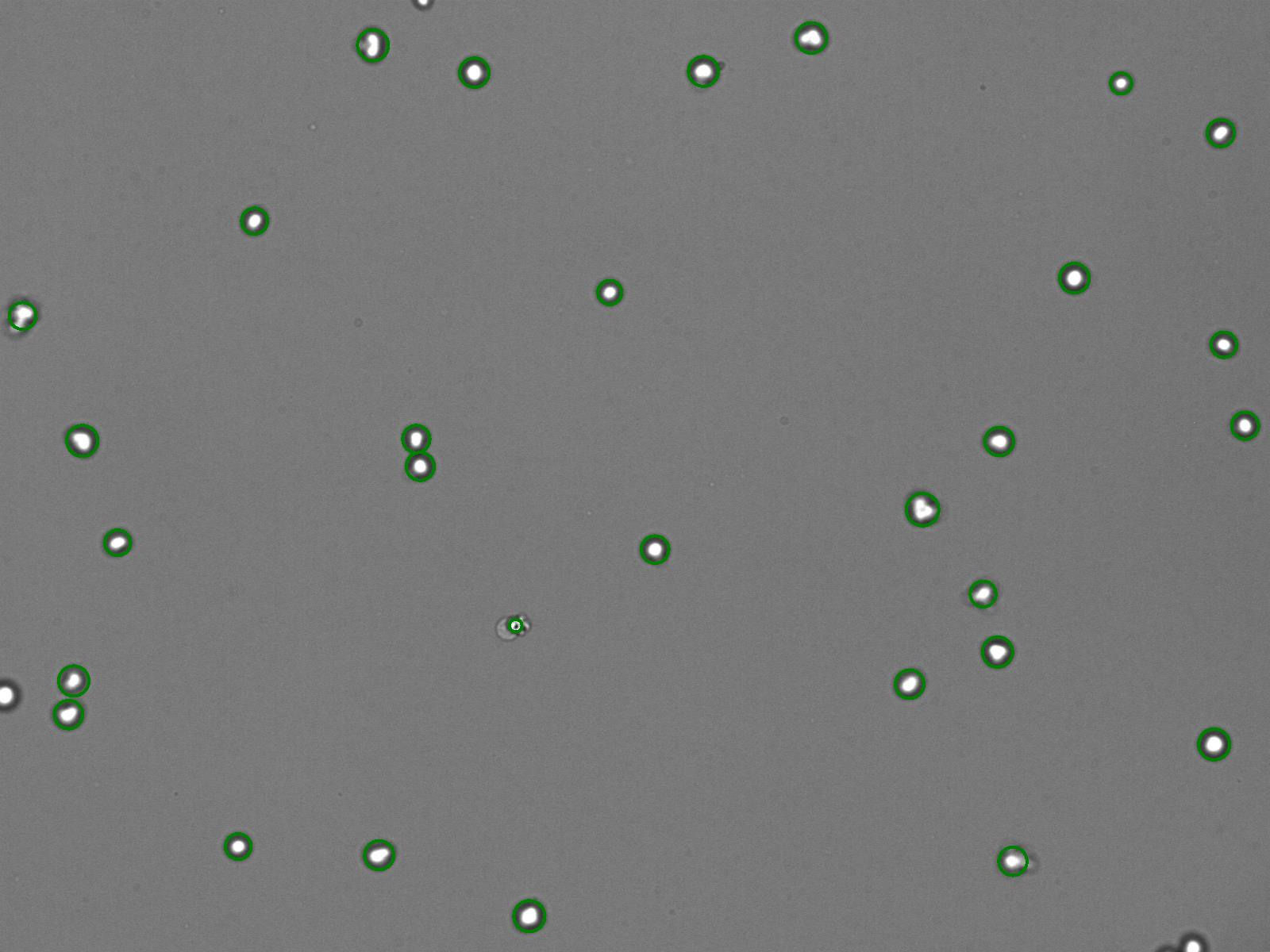

Supplement: Supplementary file 1 — Supplementary Information 1. [file 41598_2020_80576_MOESM1_ESM.zip › S1/Aggregate counts/day5/0mmHg Feb1 47 46/ML C3-030_2019-02-17_162620.bmp]

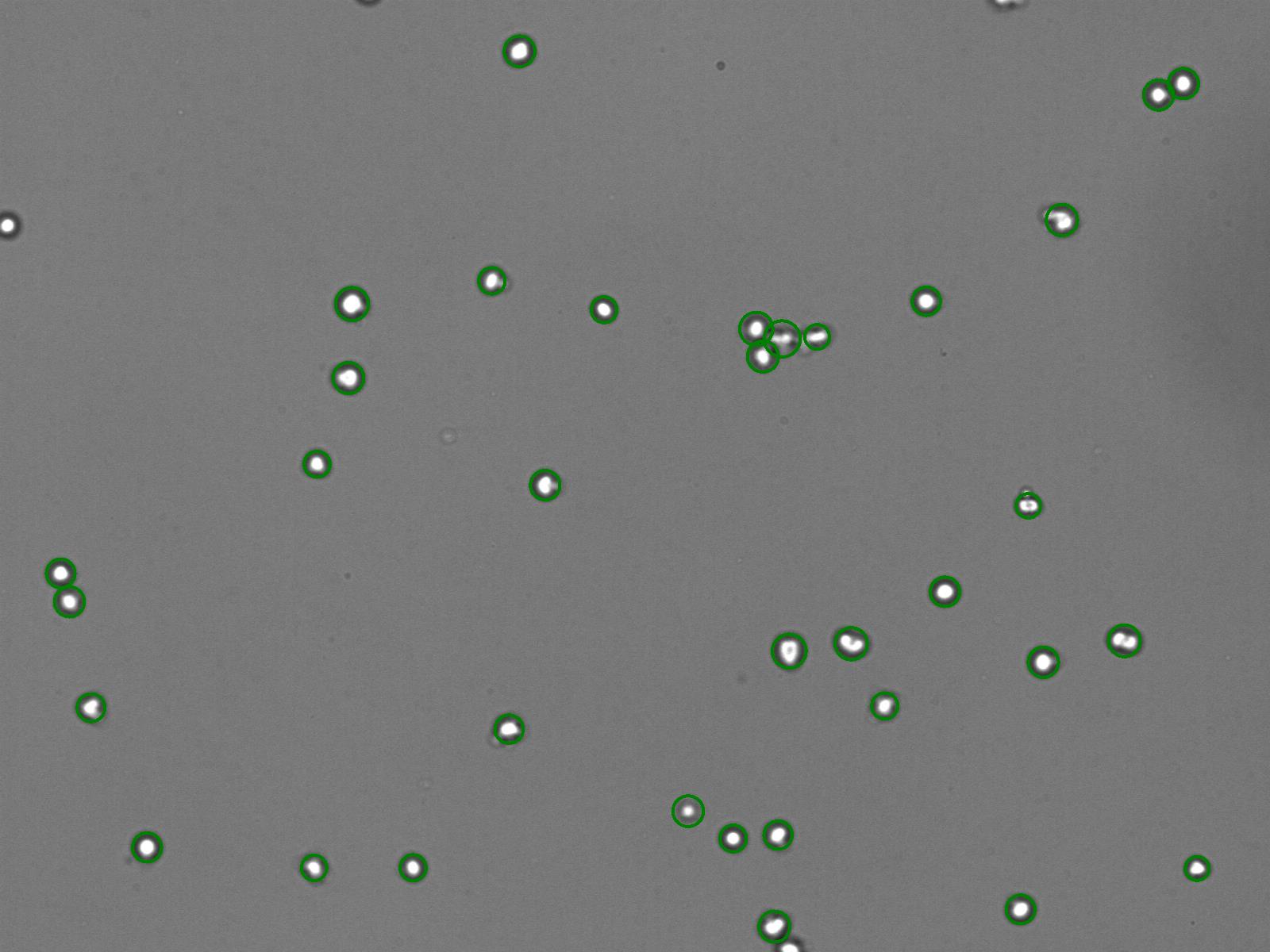

Supplement: Supplementary file 1 — Supplementary Information 1. [file 41598_2020_80576_MOESM1_ESM.zip › S1/Aggregate counts/day5/0mmHg Feb1 47 46/ML C3-031_2019-02-17_162621.bmp]

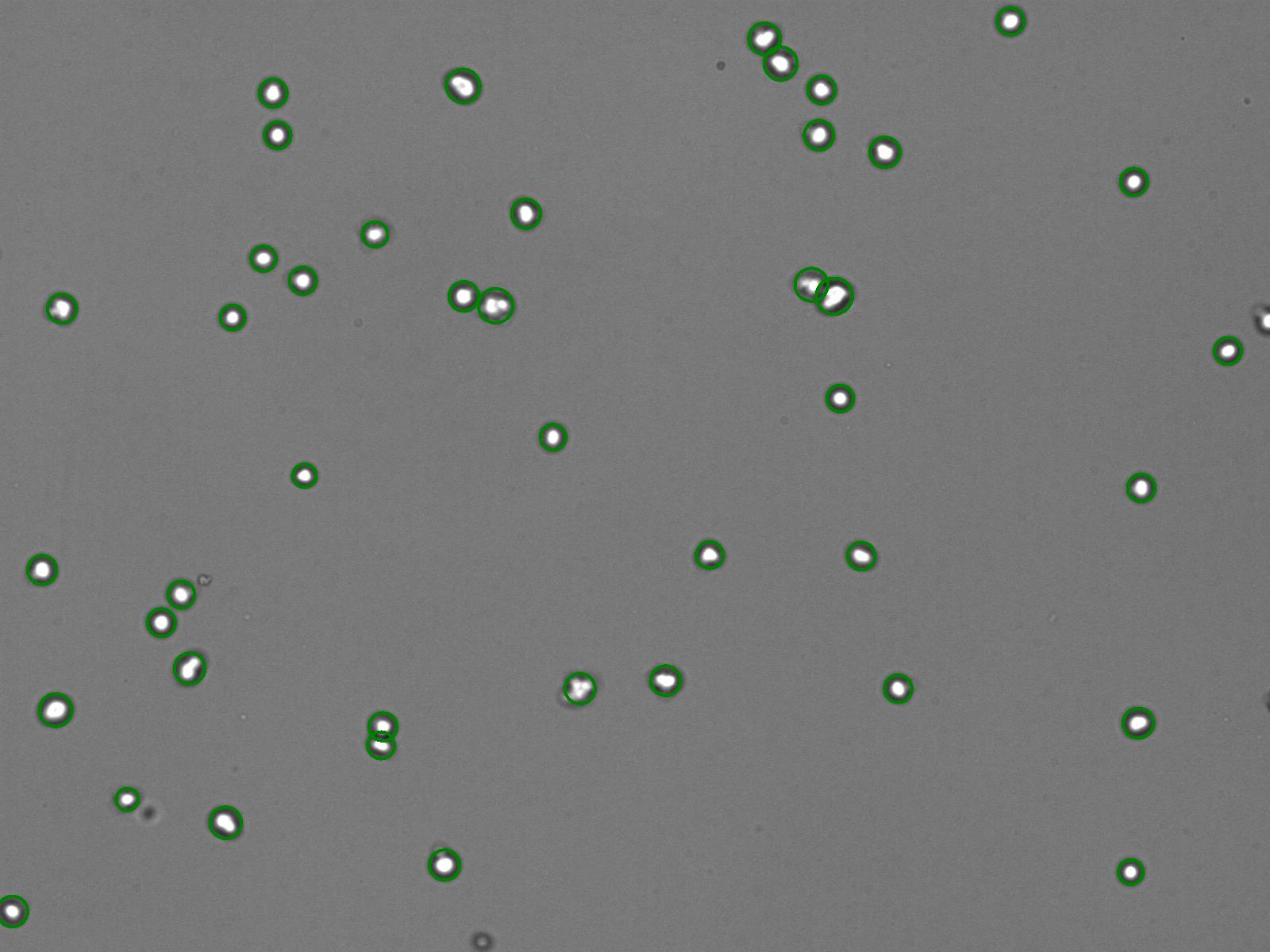

Supplement: Supplementary file 1 — Supplementary Information 1. [file 41598_2020_80576_MOESM1_ESM.zip › S1/Aggregate counts/day5/0mmHg Feb1 47 46/ML C3-032_2019-02-17_162621.bmp]

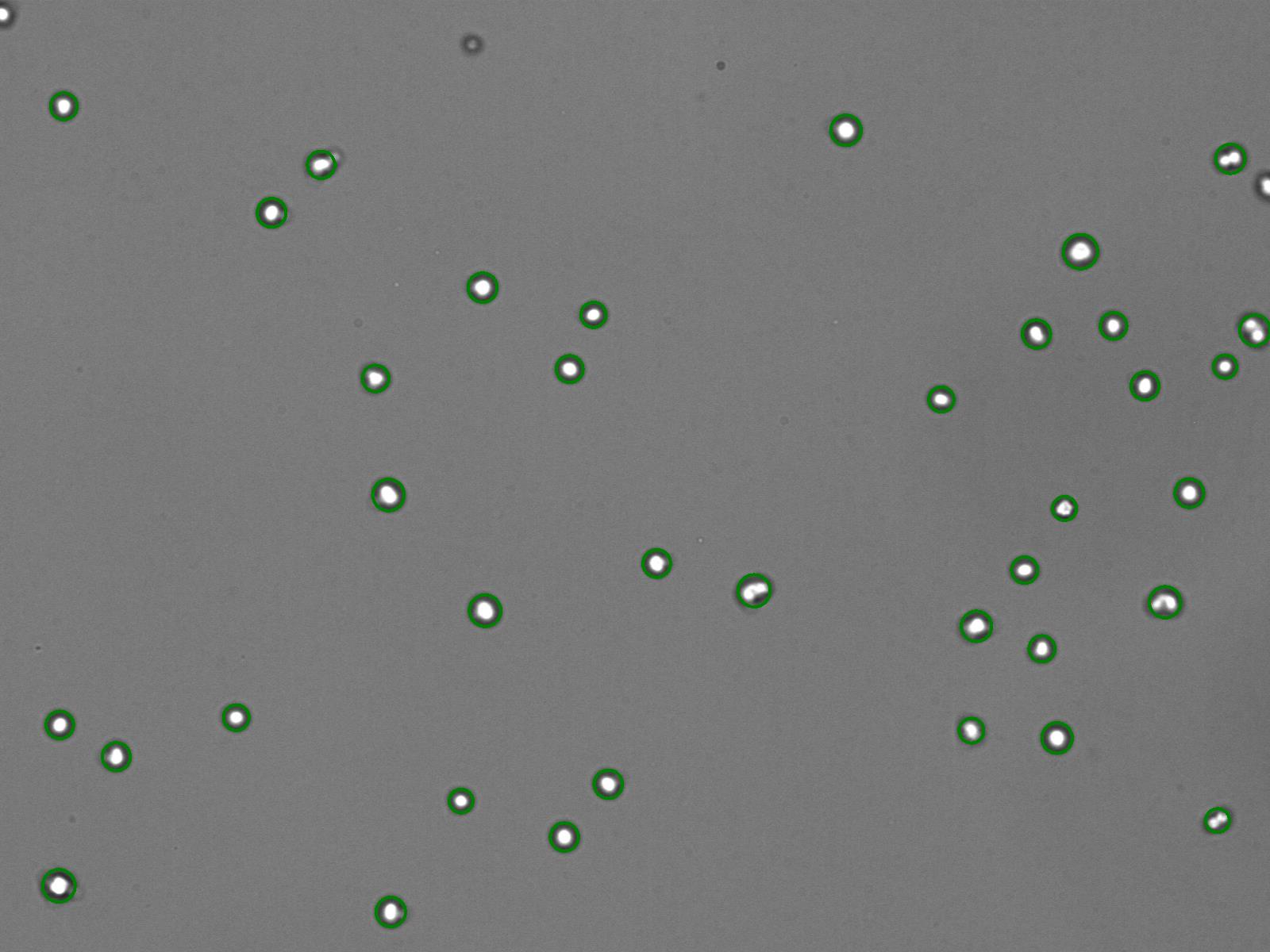

Supplement: Supplementary file 1 — Supplementary Information 1. [file 41598_2020_80576_MOESM1_ESM.zip › S1/Aggregate counts/day5/0mmHg Feb1 47 46/ML C3-033_2019-02-17_162621.bmp]

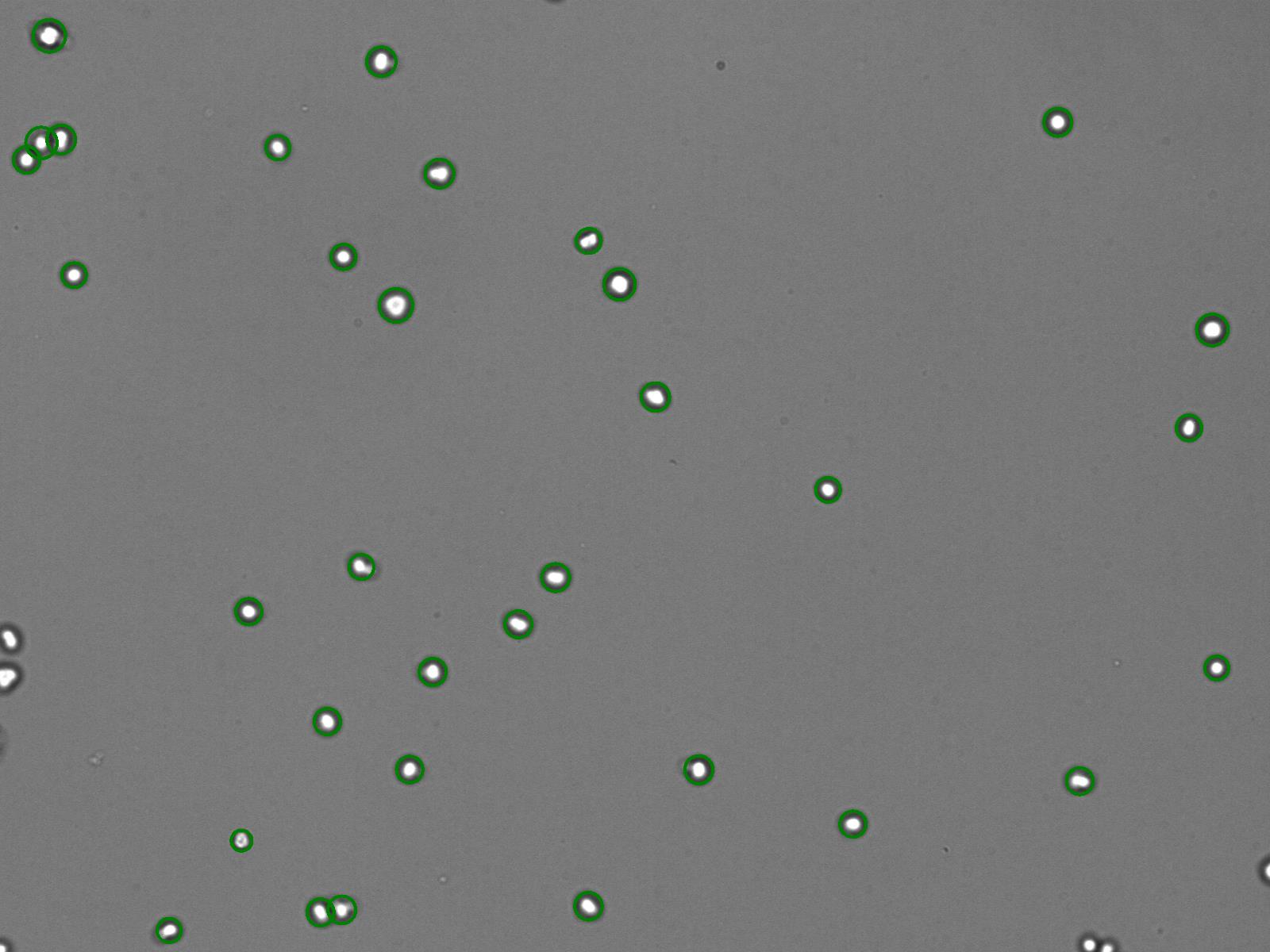

Supplement: Supplementary file 1 — Supplementary Information 1. [file 41598_2020_80576_MOESM1_ESM.zip › S1/Aggregate counts/day5/0mmHg Feb1 47 46/ML C3-034_2019-02-17_162621.bmp]

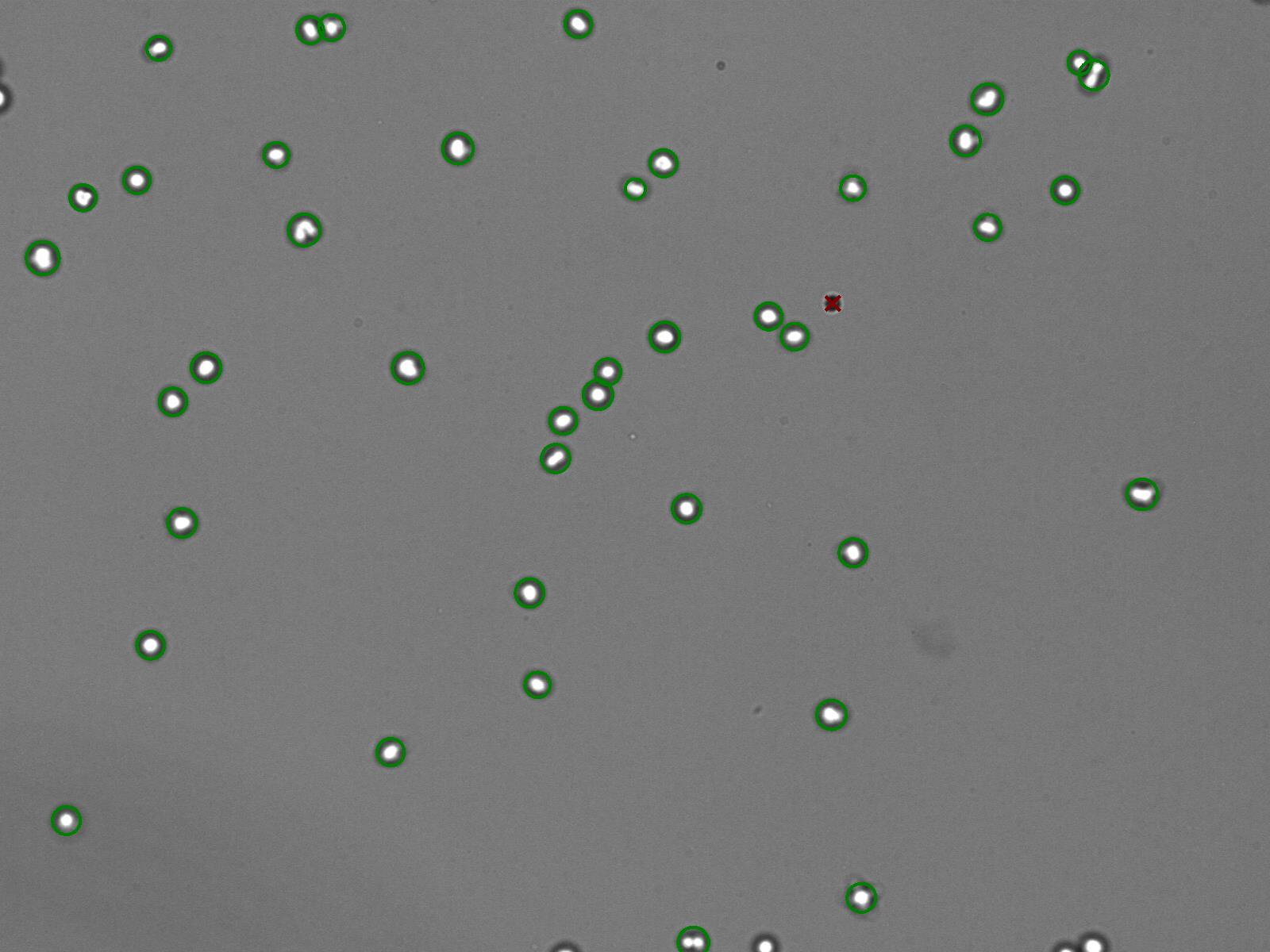

Supplement: Supplementary file 1 — Supplementary Information 1. [file 41598_2020_80576_MOESM1_ESM.zip › S1/Aggregate counts/day5/0mmHg Feb1 47 46/ML C3-035_2019-02-17_162622.bmp]

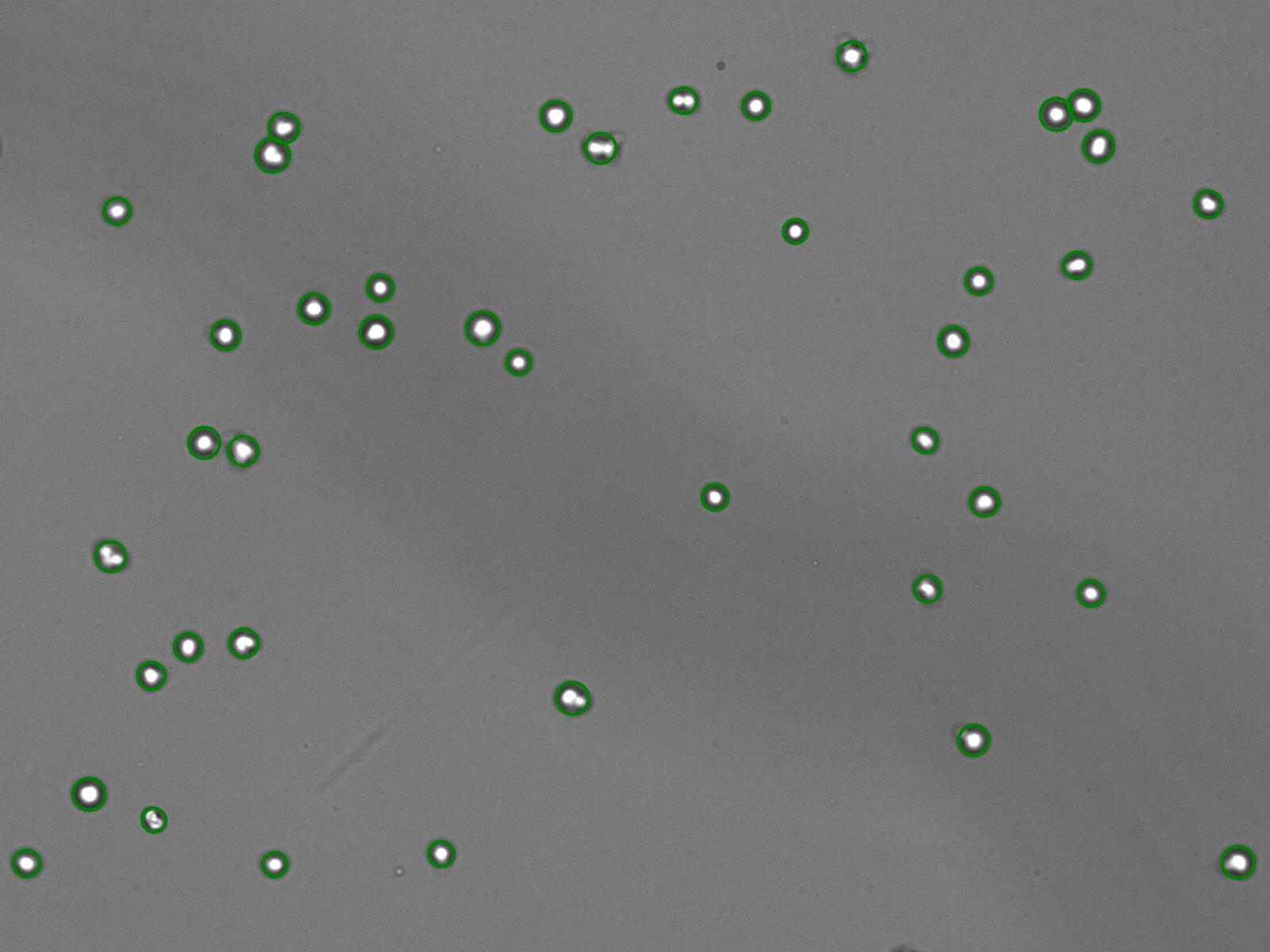

Supplement: Supplementary file 1 — Supplementary Information 1. [file 41598_2020_80576_MOESM1_ESM.zip › S1/Aggregate counts/day5/0mmHg Feb1 47 46/ML C3-036_2019-02-17_162622.bmp]

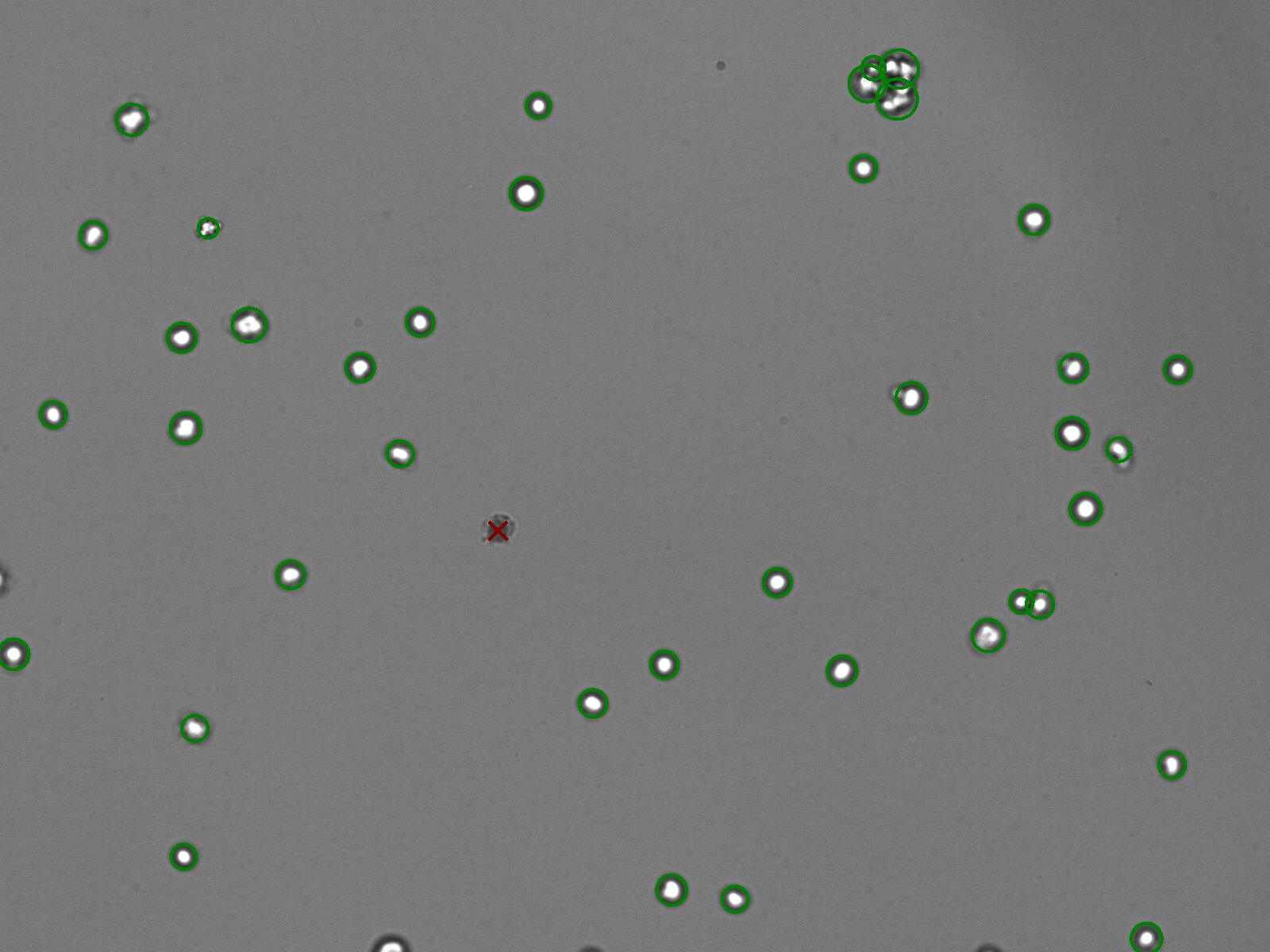

Supplement: Supplementary file 1 — Supplementary Information 1. [file 41598_2020_80576_MOESM1_ESM.zip › S1/Aggregate counts/day5/0mmHg Feb1 47 46/ML C3-037_2019-02-17_162622.bmp]

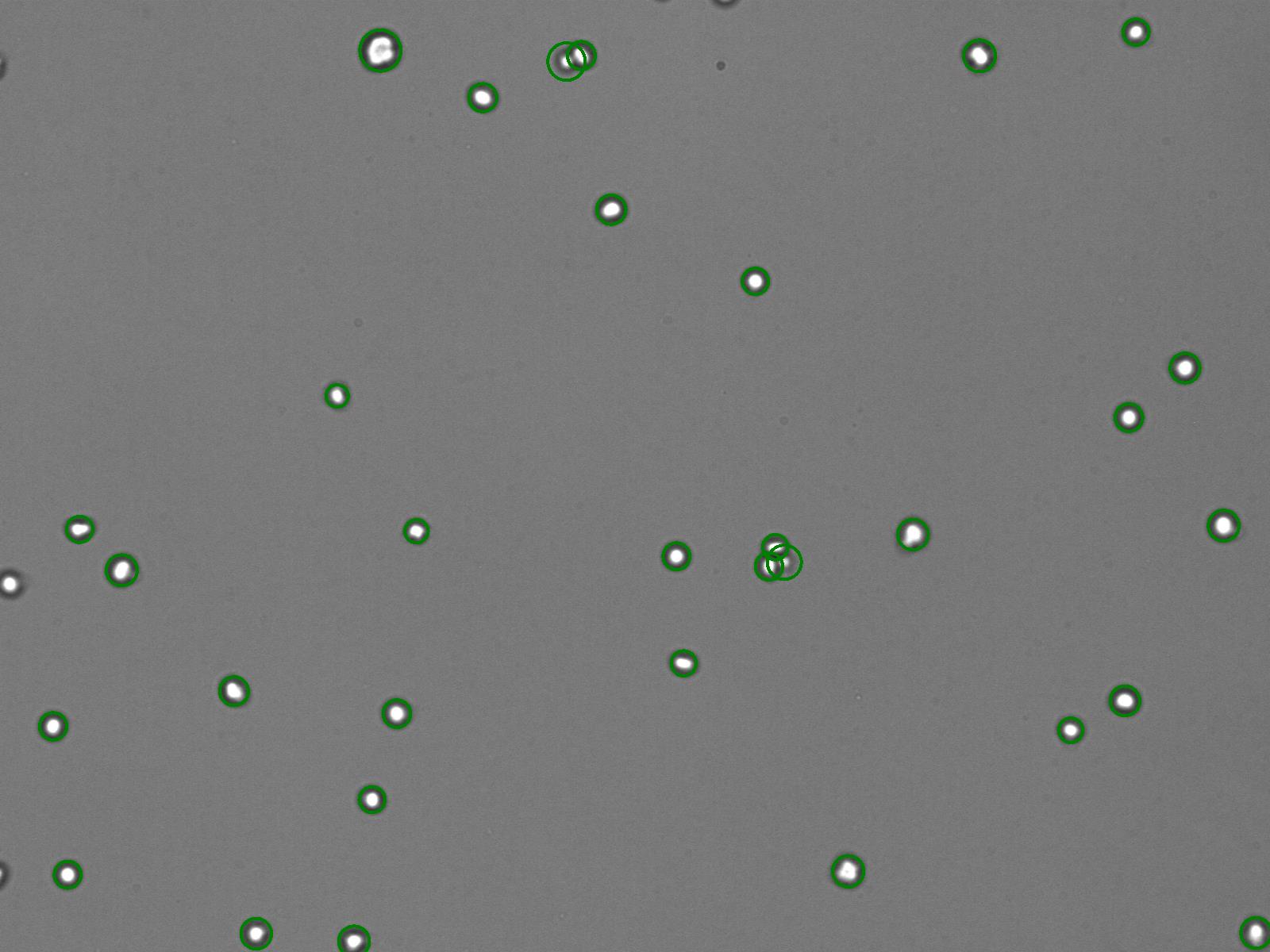

Supplement: Supplementary file 1 — Supplementary Information 1. [file 41598_2020_80576_MOESM1_ESM.zip › S1/Aggregate counts/day5/0mmHg Feb1 47 46/ML C3-038_2019-02-17_162623.bmp]

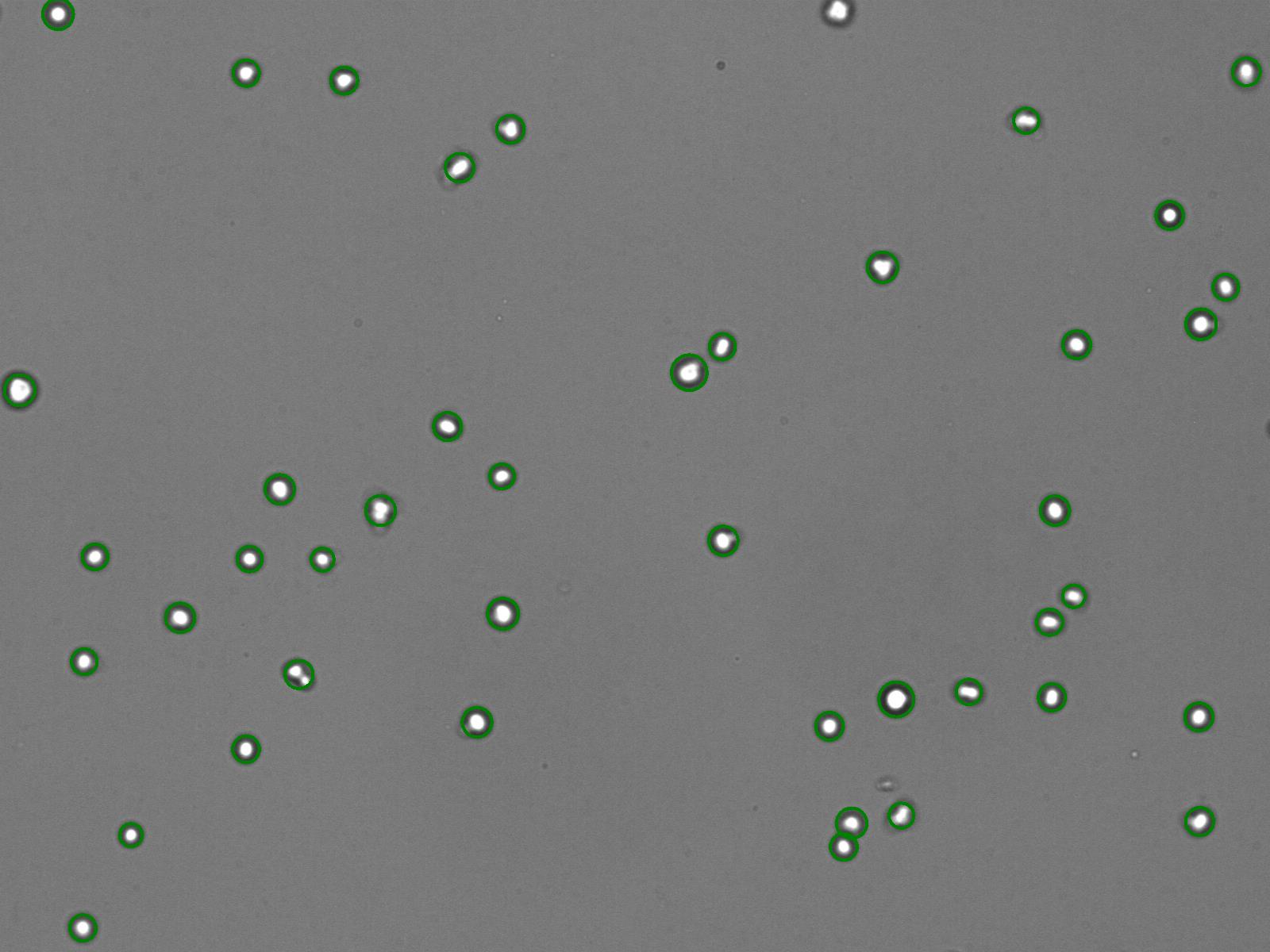

Supplement: Supplementary file 1 — Supplementary Information 1. [file 41598_2020_80576_MOESM1_ESM.zip › S1/Aggregate counts/day5/0mmHg Feb1 47 46/ML C3-039_2019-02-17_162623.bmp]

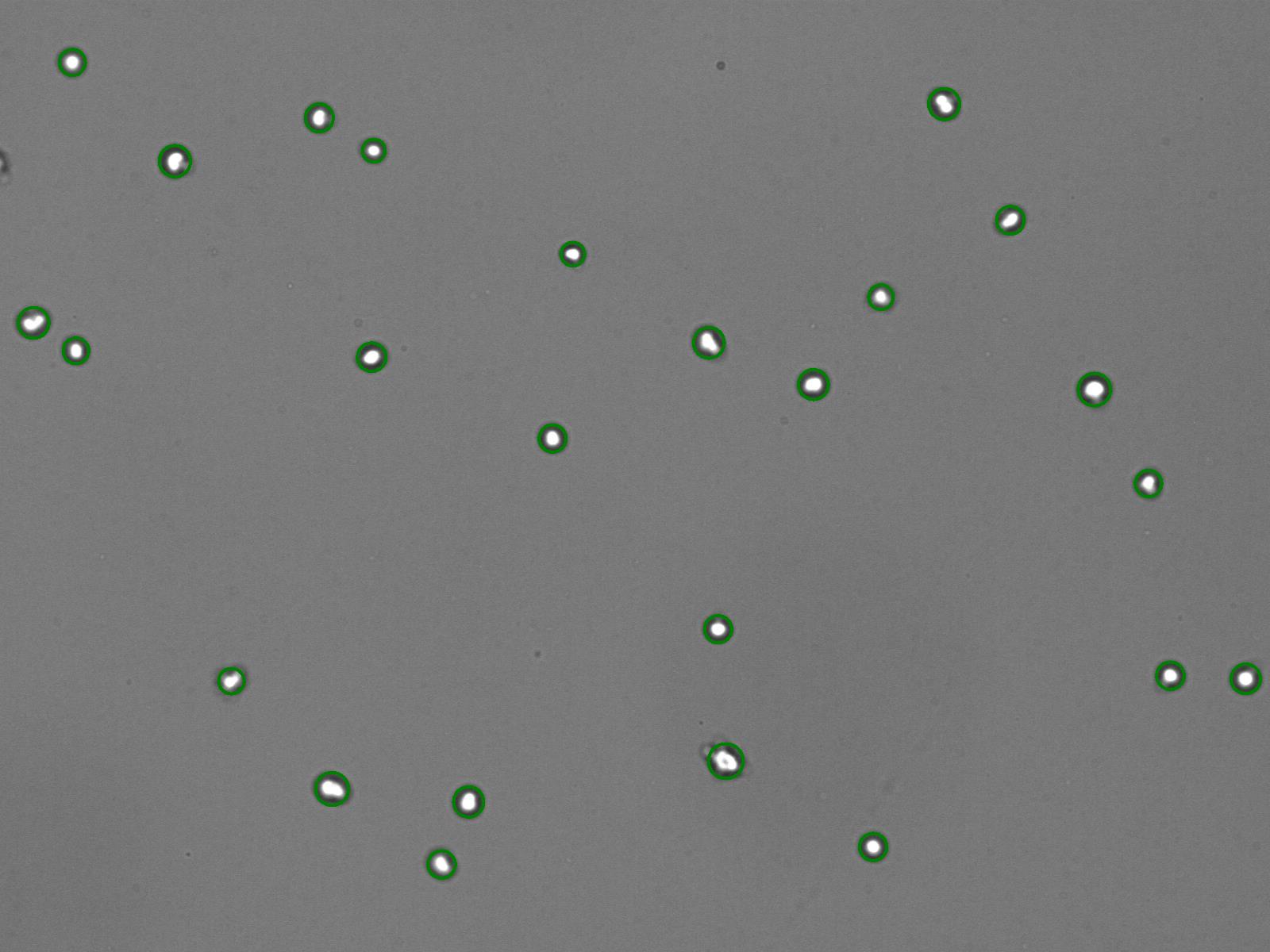

Supplement: Supplementary file 1 — Supplementary Information 1. [file 41598_2020_80576_MOESM1_ESM.zip › S1/Aggregate counts/day5/0mmHg Feb1 47 46/ML C3-040_2019-02-17_162623.bmp]

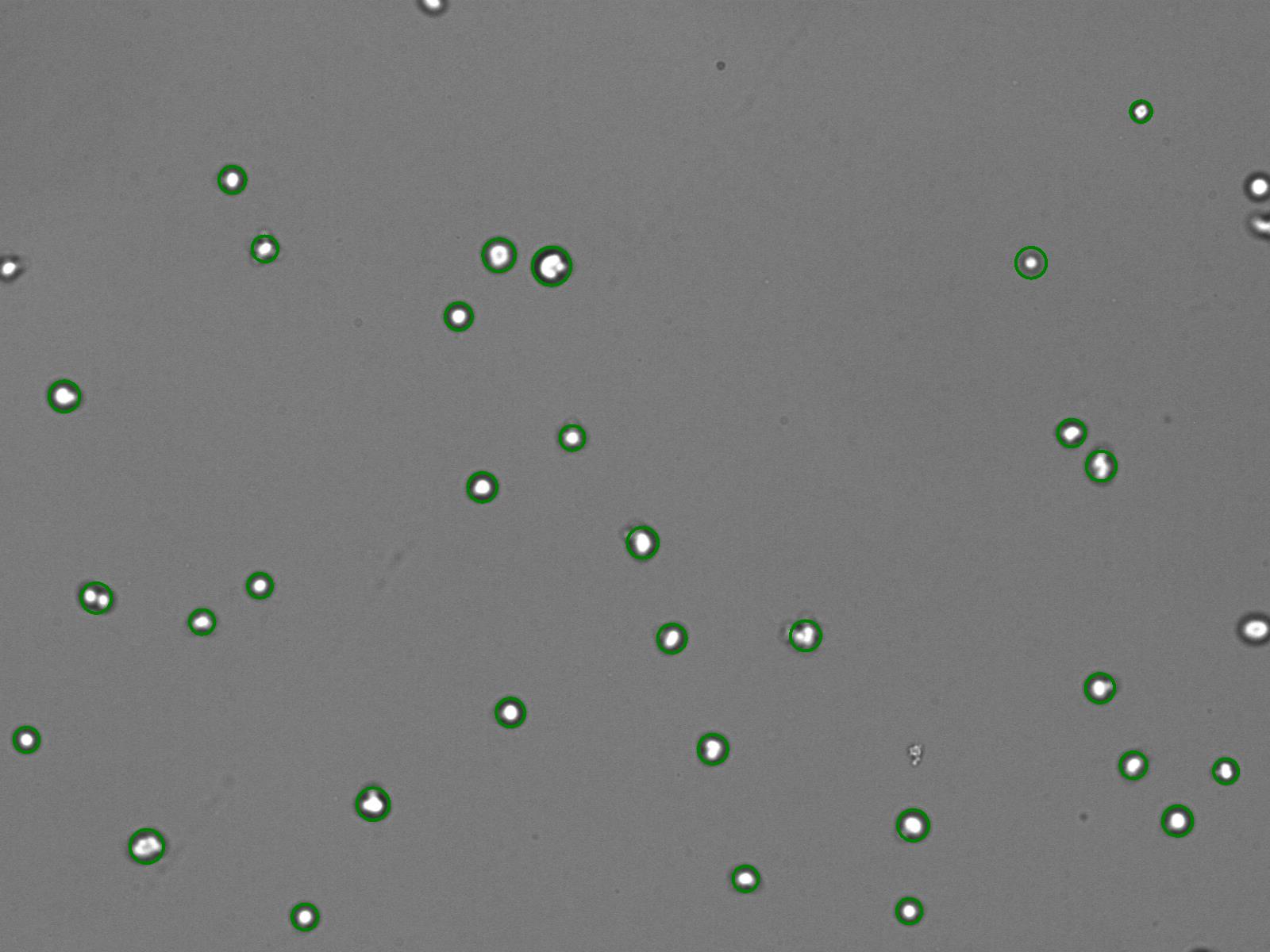

Supplement: Supplementary file 1 — Supplementary Information 1. [file 41598_2020_80576_MOESM1_ESM.zip › S1/Aggregate counts/day5/0mmHg Feb1 47 46/ML C3-041_2019-02-17_162623.bmp]

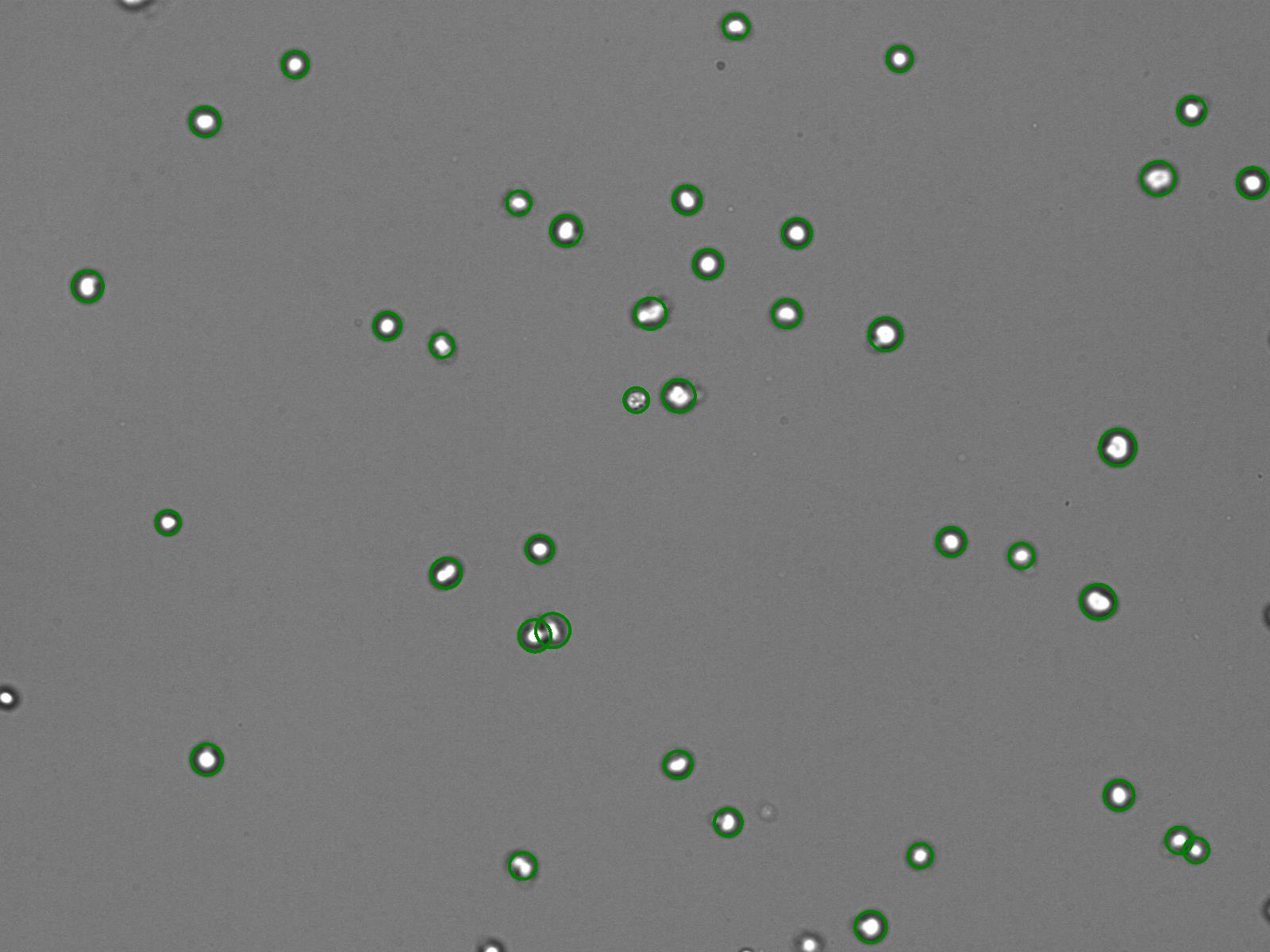

Supplement: Supplementary file 1 — Supplementary Information 1. [file 41598_2020_80576_MOESM1_ESM.zip › S1/Aggregate counts/day5/0mmHg Feb1 47 46/ML C3-042_2019-02-17_162624.bmp]

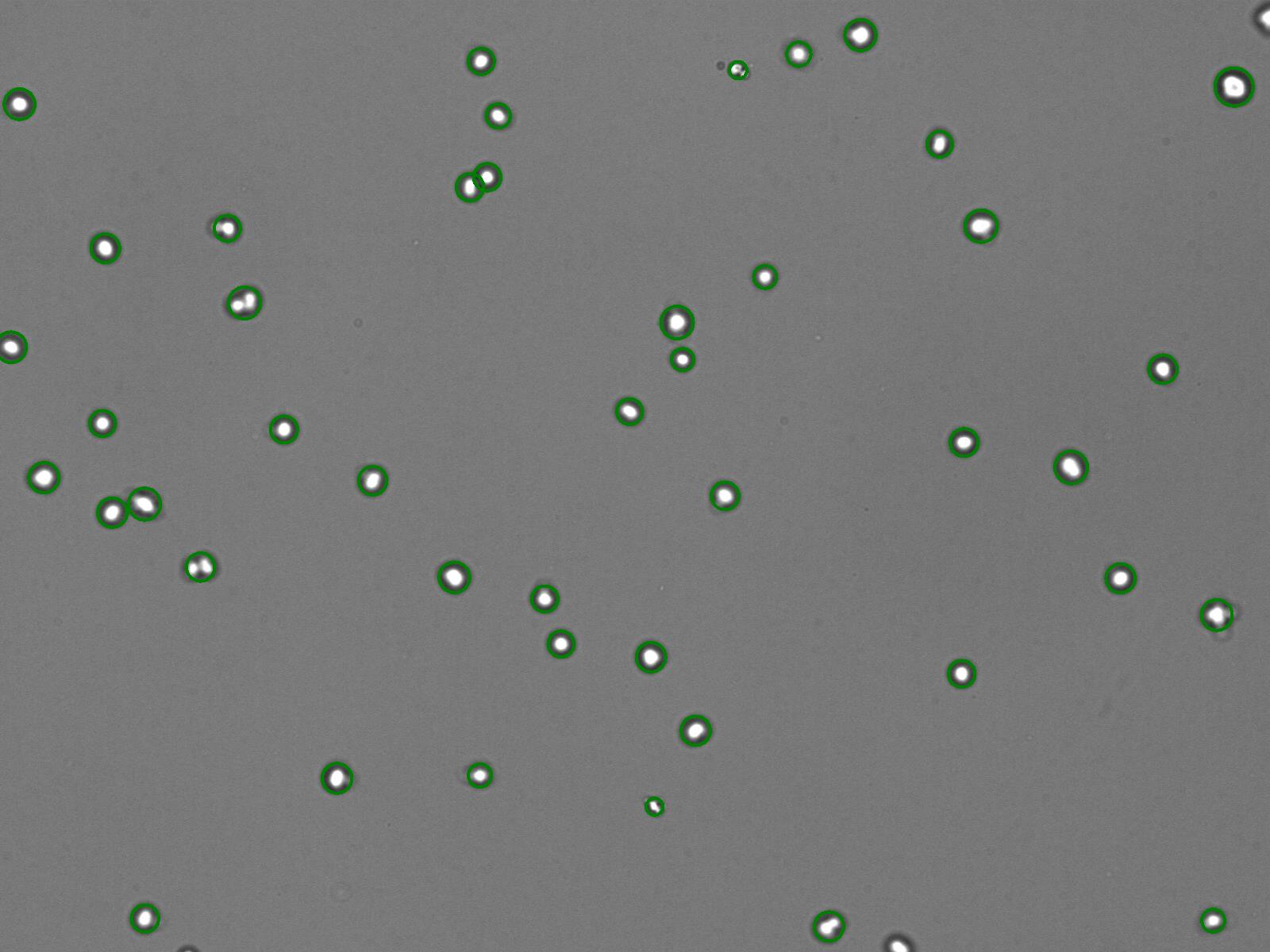

Supplement: Supplementary file 1 — Supplementary Information 1. [file 41598_2020_80576_MOESM1_ESM.zip › S1/Aggregate counts/day5/0mmHg Feb1 47 46/ML C3-043_2019-02-17_162624.bmp]

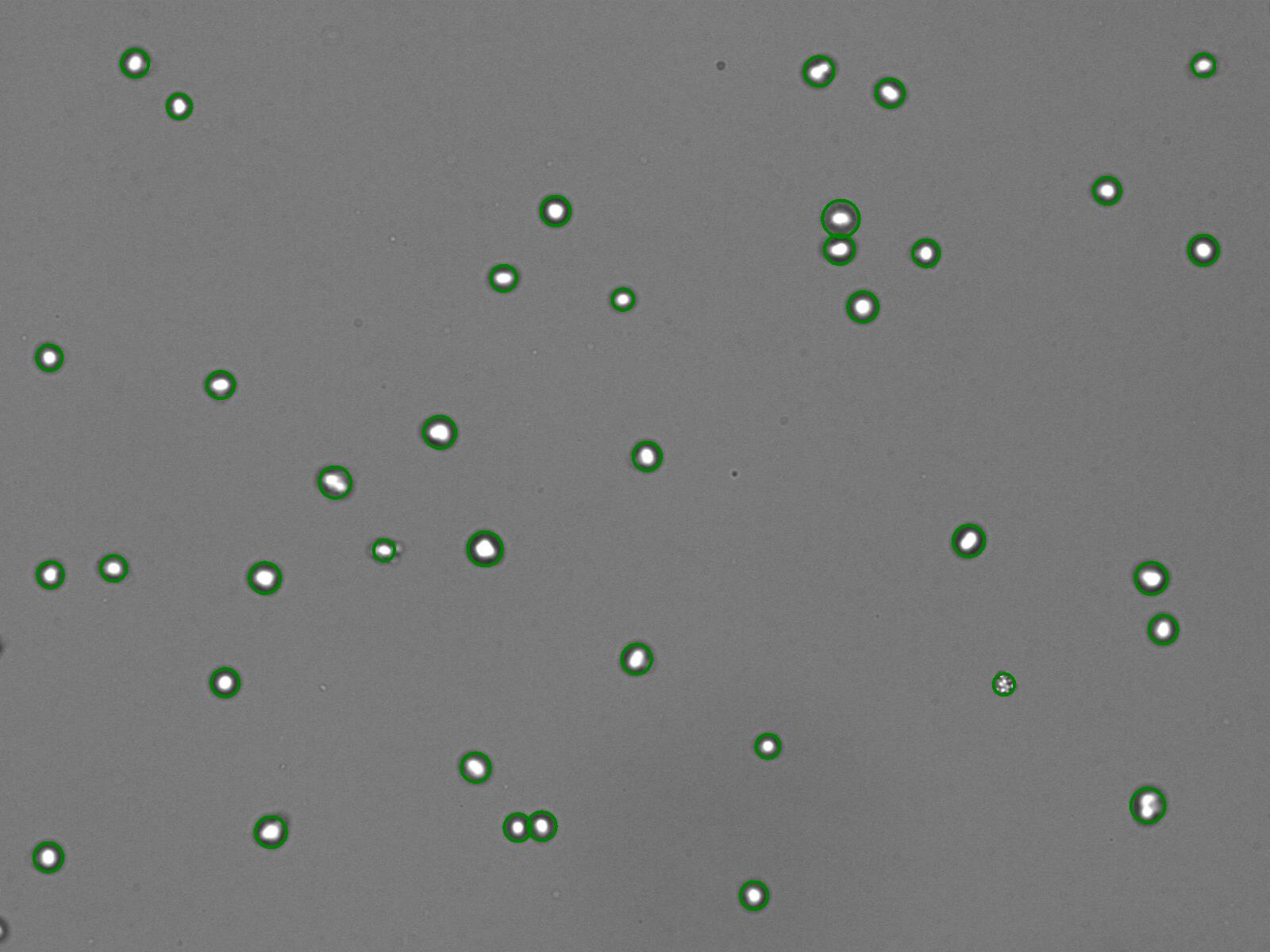

Supplement: Supplementary file 1 — Supplementary Information 1. [file 41598_2020_80576_MOESM1_ESM.zip › S1/Aggregate counts/day5/0mmHg Feb1 47 46/ML C3-044_2019-02-17_162624.bmp]

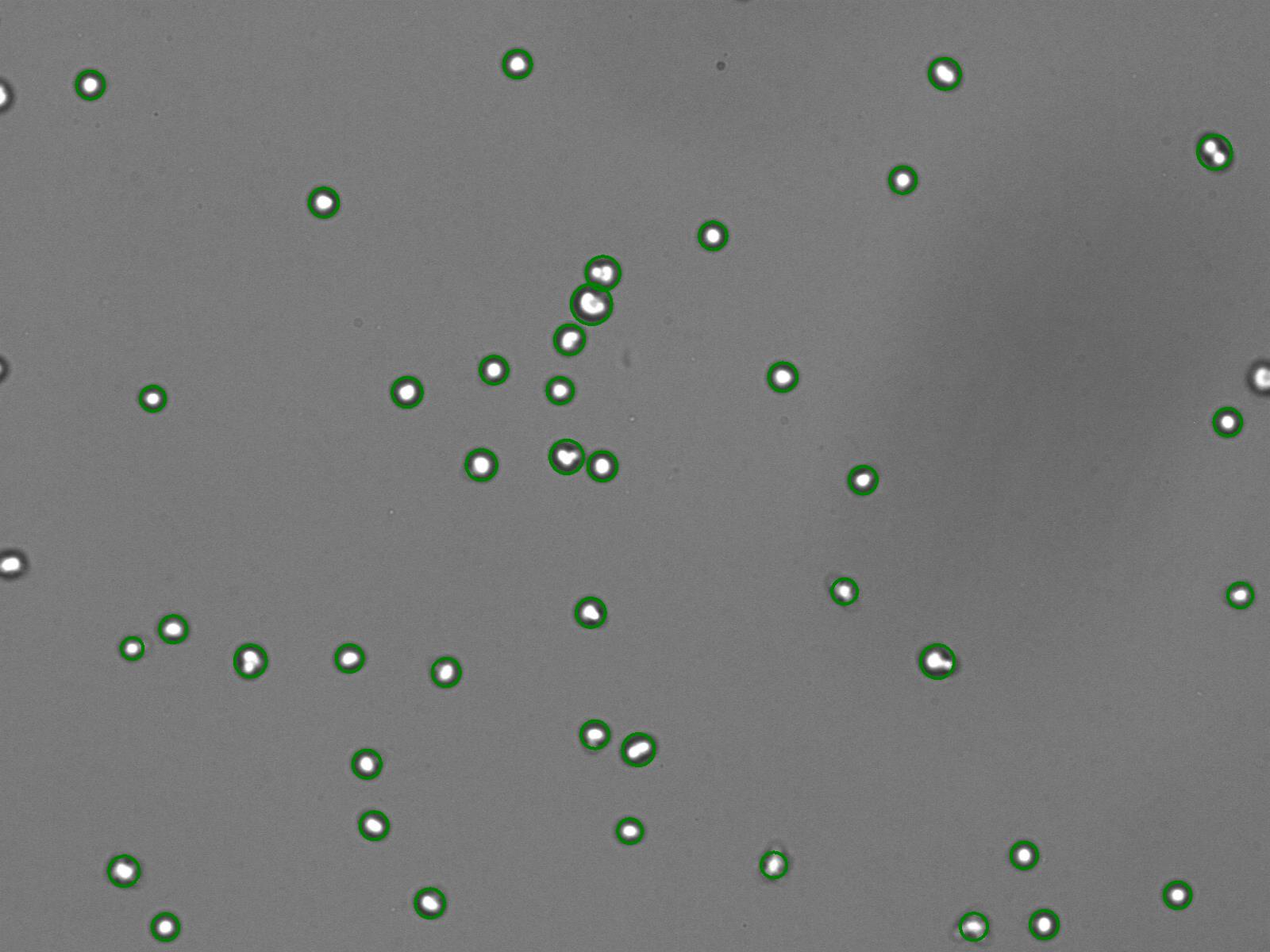

Supplement: Supplementary file 1 — Supplementary Information 1. [file 41598_2020_80576_MOESM1_ESM.zip › S1/Aggregate counts/day5/0mmHg Feb1 47 46/ML C3-045_2019-02-17_162625.bmp]

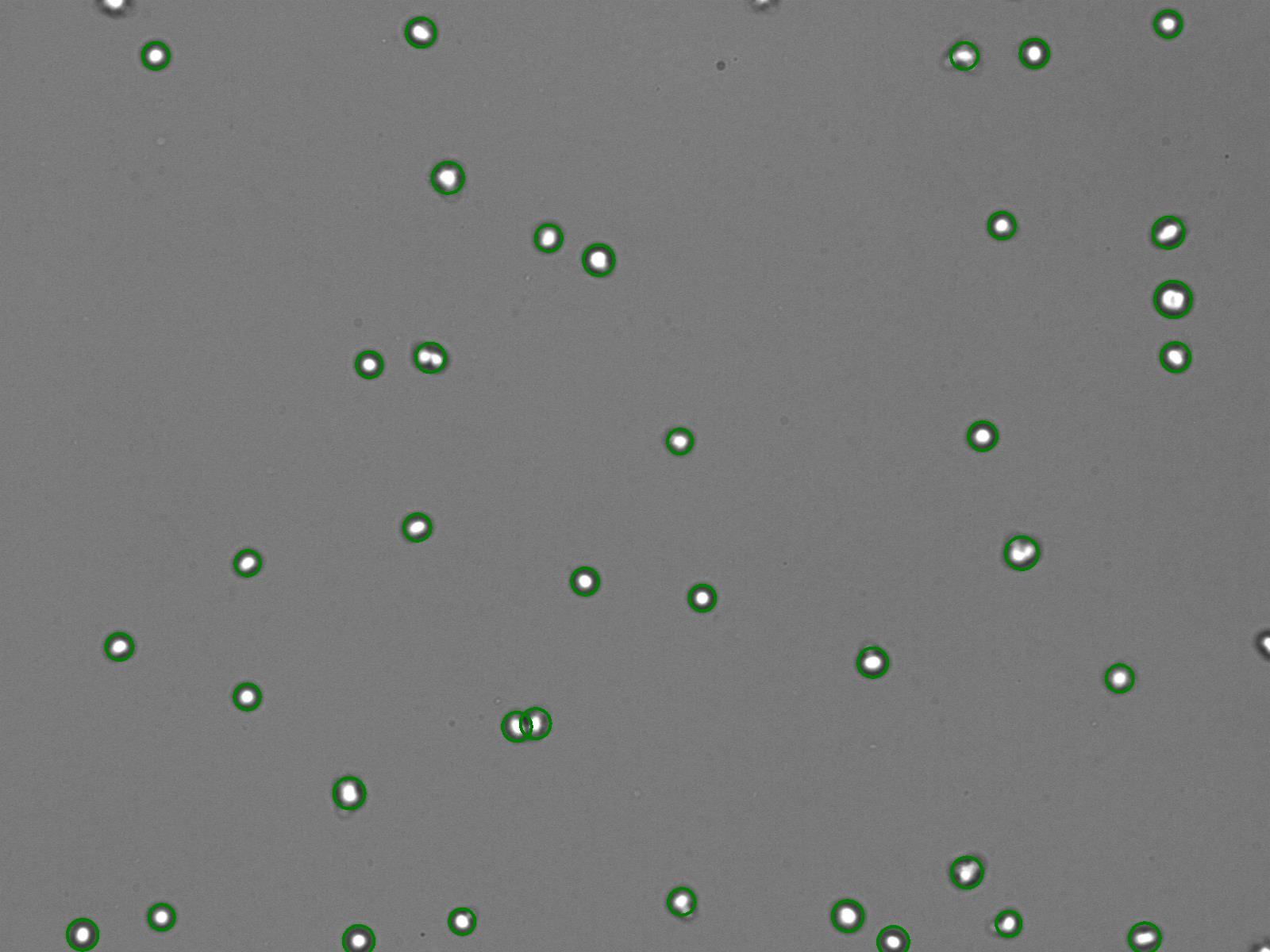

Supplement: Supplementary file 1 — Supplementary Information 1. [file 41598_2020_80576_MOESM1_ESM.zip › S1/Aggregate counts/day5/0mmHg Feb1 47 46/ML C3-046_2019-02-17_162625.bmp]

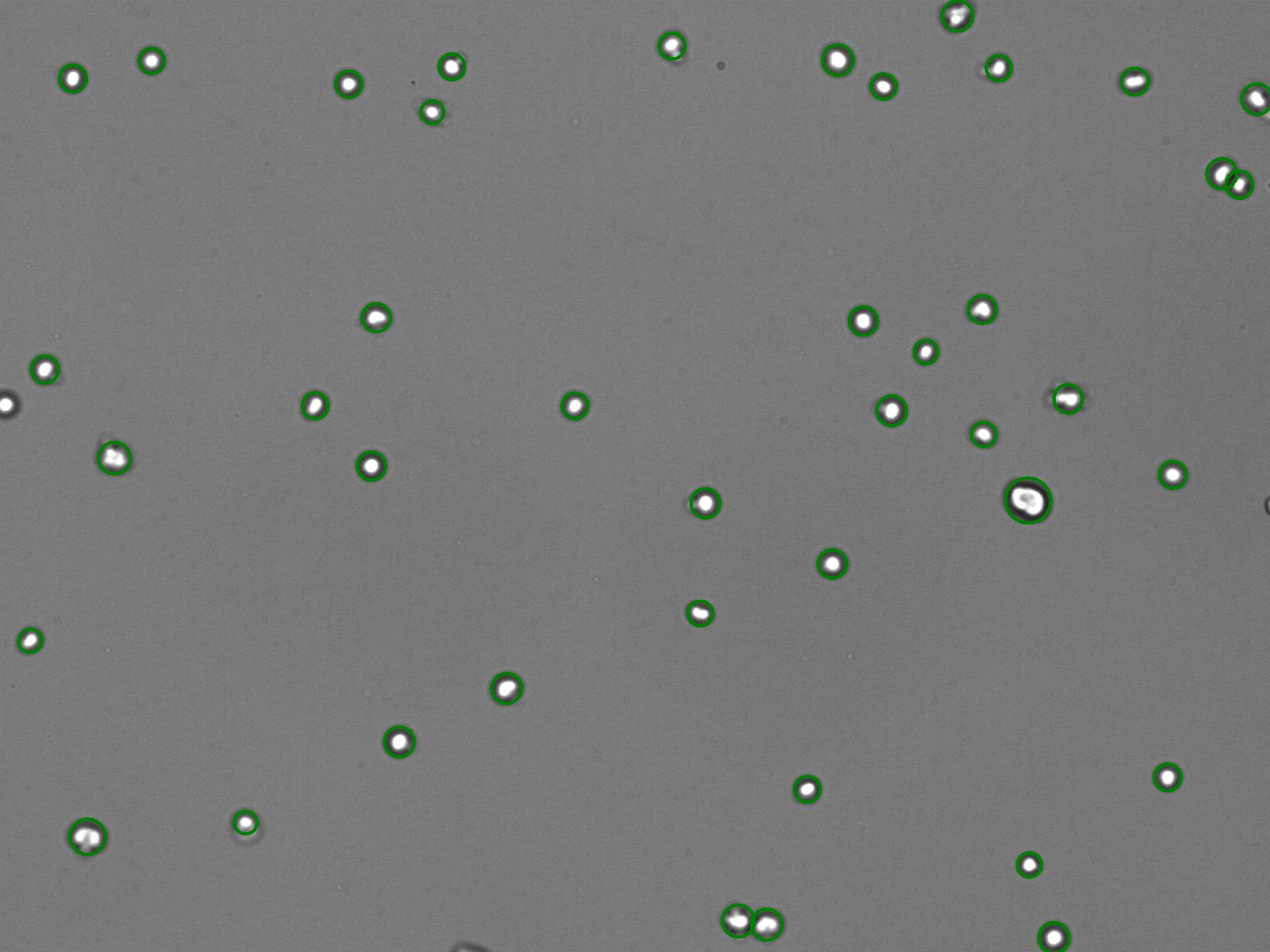

Supplement: Supplementary file 1 — Supplementary Information 1. [file 41598_2020_80576_MOESM1_ESM.zip › S1/Aggregate counts/day5/0mmHg Feb1 47 46/ML C3-047_2019-02-17_162625.bmp]

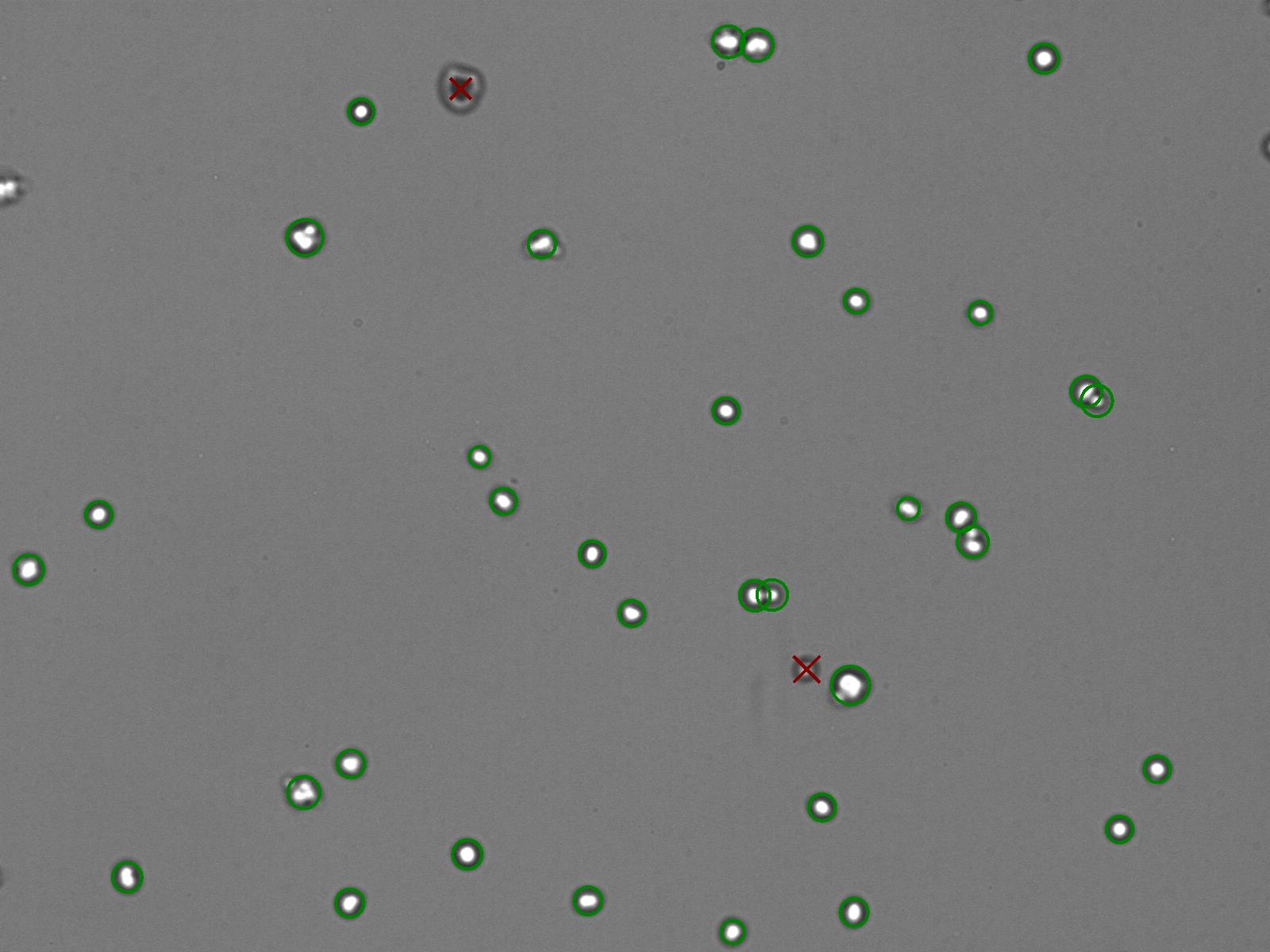

Supplement: Supplementary file 1 — Supplementary Information 1. [file 41598_2020_80576_MOESM1_ESM.zip › S1/Aggregate counts/day5/0mmHg Feb1 47 46/ML C3-048_2019-02-17_162625.bmp]

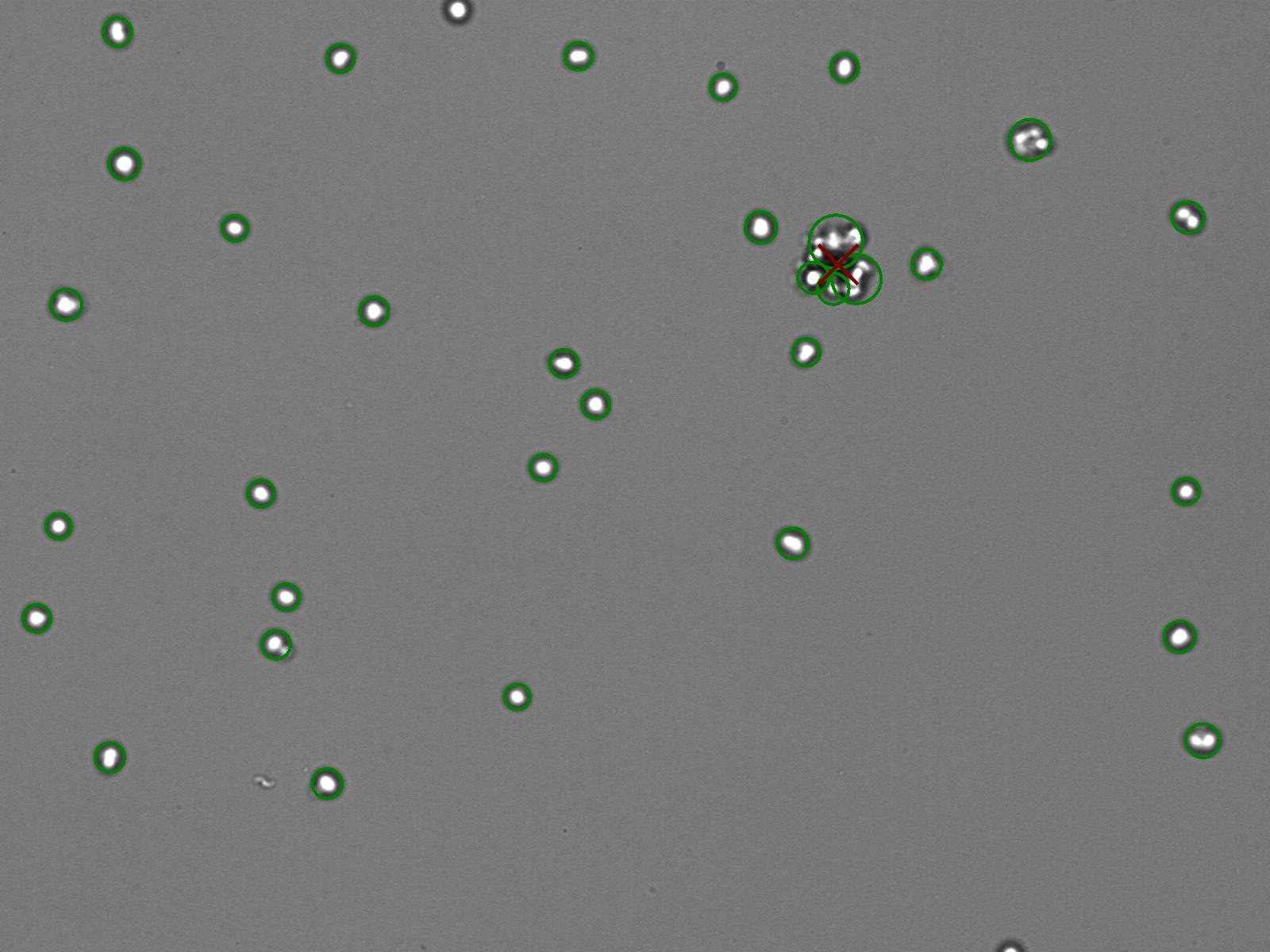

Supplement: Supplementary file 1 — Supplementary Information 1. [file 41598_2020_80576_MOESM1_ESM.zip › S1/Aggregate counts/day5/0mmHg Feb1 47 46/ML C3-049_2019-02-17_162626.bmp]

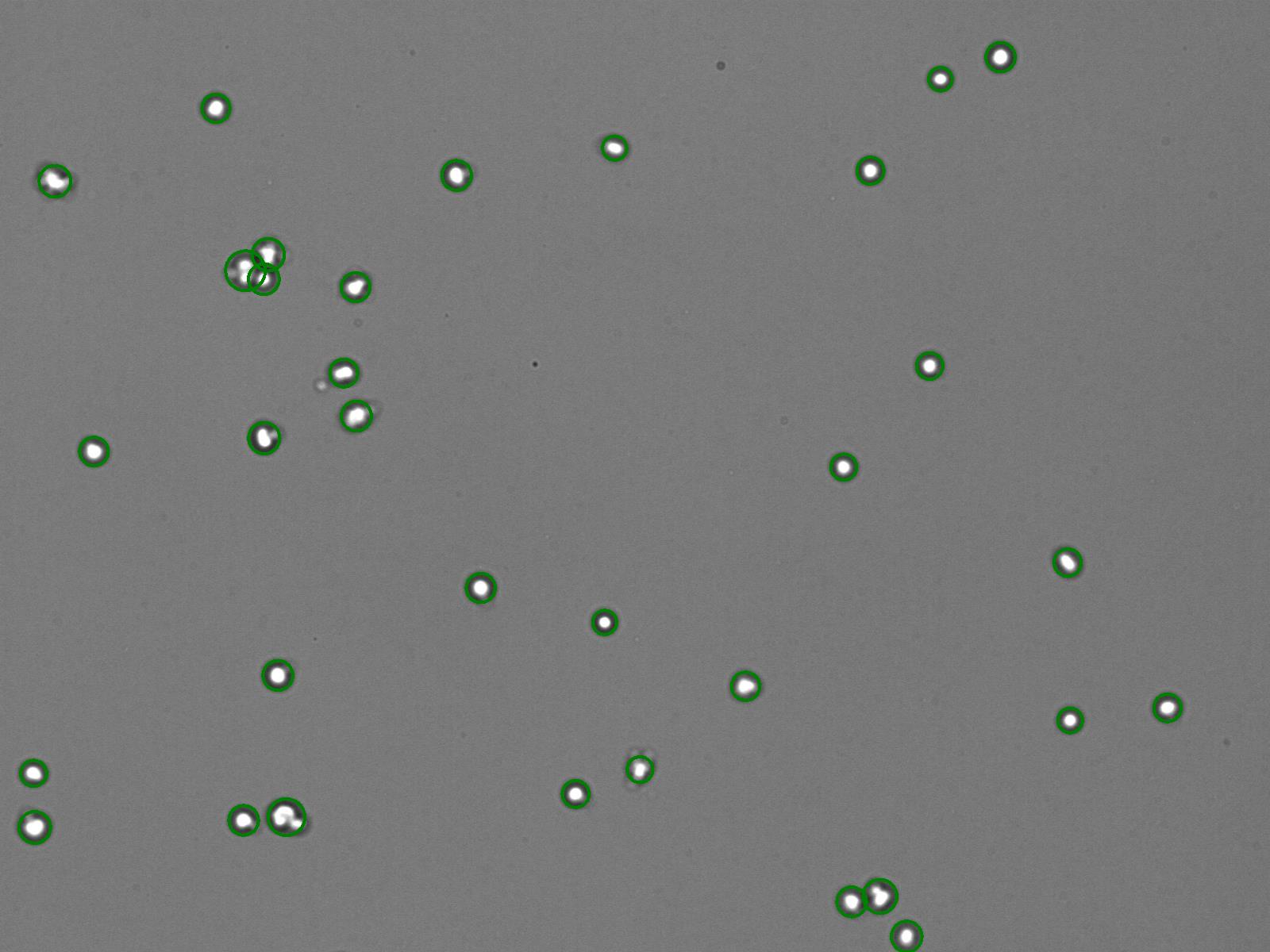

Supplement: Supplementary file 1 — Supplementary Information 1. [file 41598_2020_80576_MOESM1_ESM.zip › S1/Aggregate counts/day5/0mmHg Feb1 47 46/ML C3-050_2019-02-17_162626.bmp]

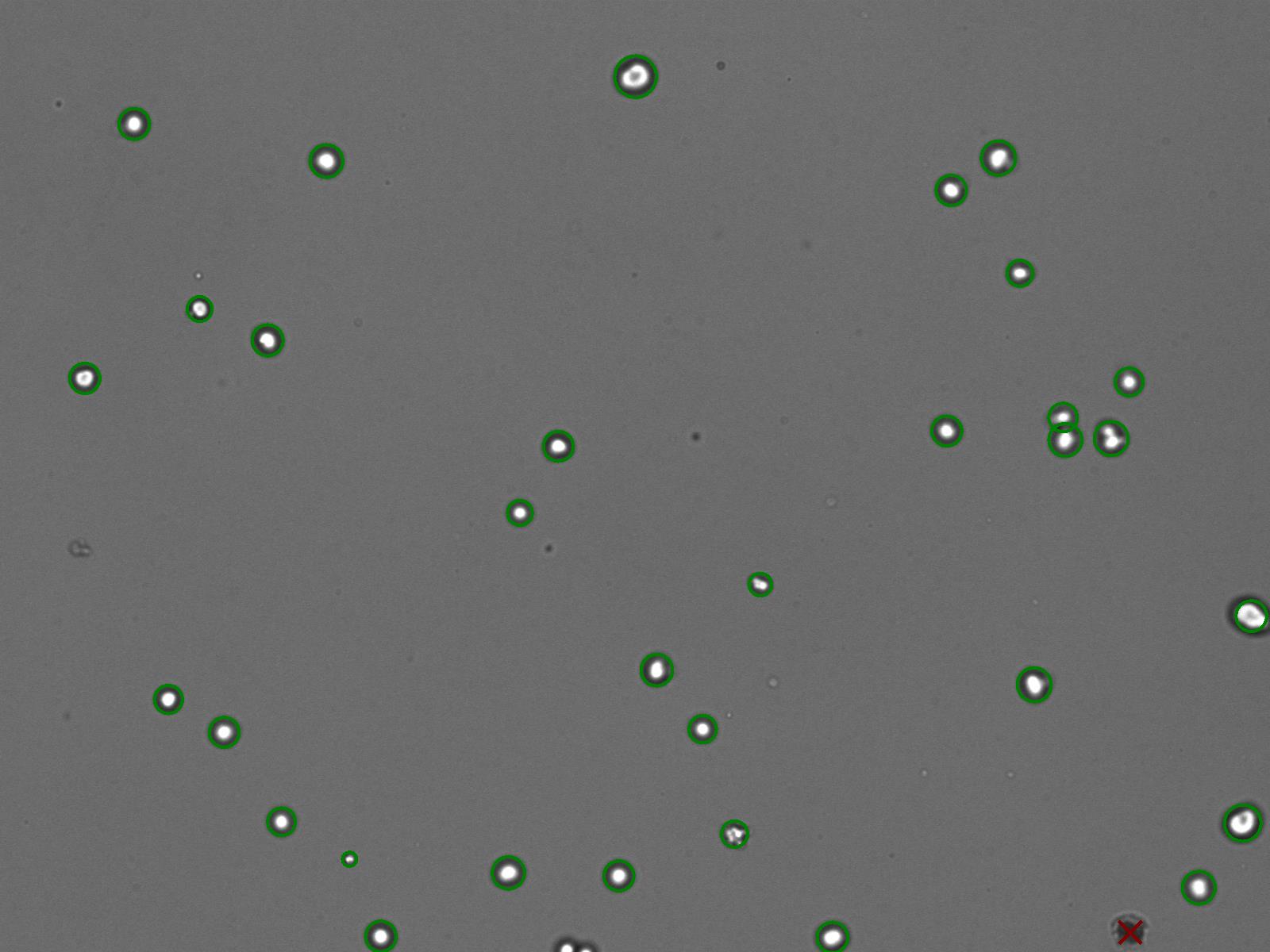

Supplement: Supplementary file 1 — Supplementary Information 1. [file 41598_2020_80576_MOESM1_ESM.zip › S1/Aggregate counts/day5/0mmHg Jan10 41 39/ML SS1 3-001_2019-02-19_112514.bmp]

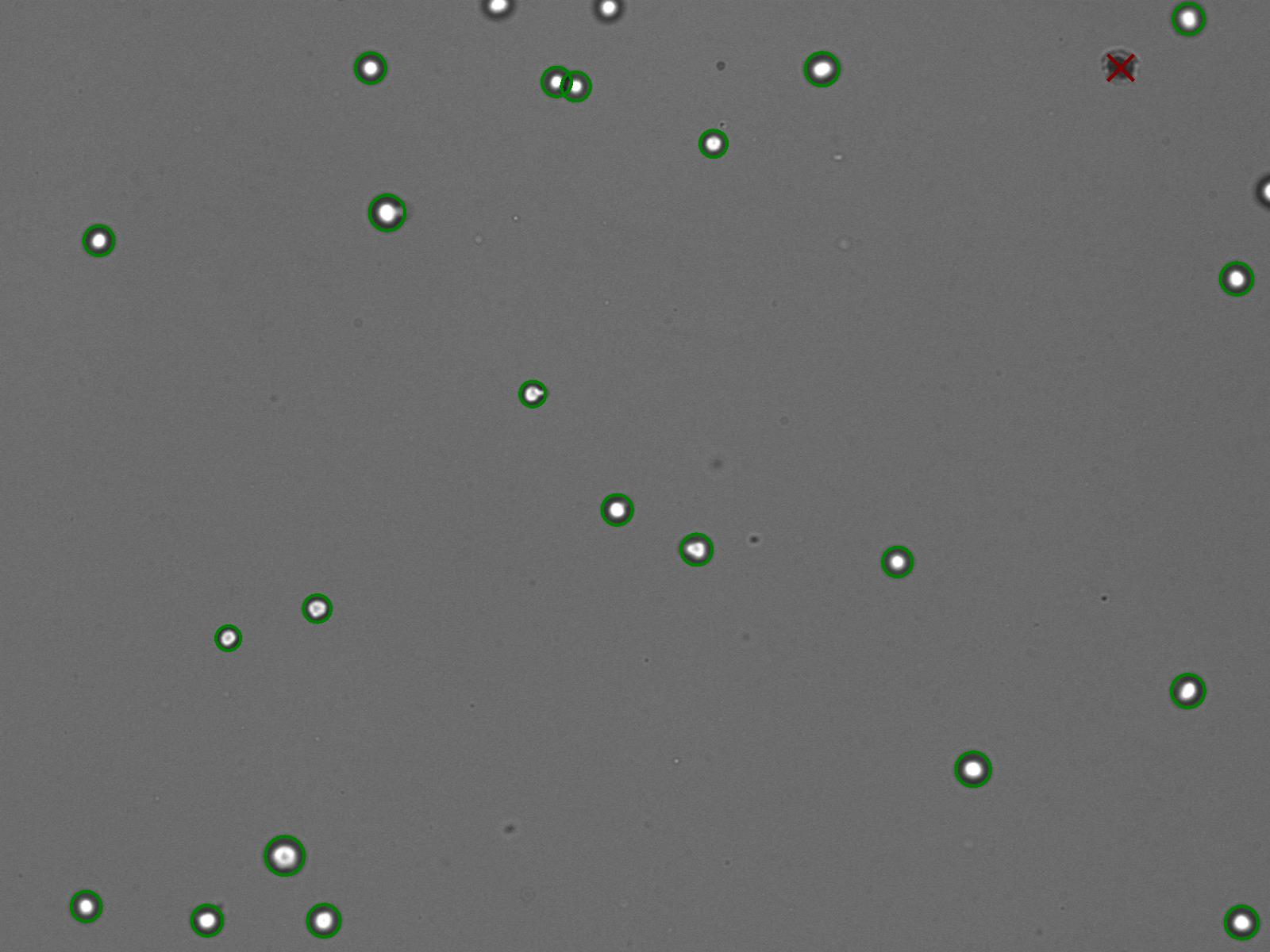

Supplement: Supplementary file 1 — Supplementary Information 1. [file 41598_2020_80576_MOESM1_ESM.zip › S1/Aggregate counts/day5/0mmHg Jan10 41 39/ML SS1 3-002_2019-02-19_112514.bmp]

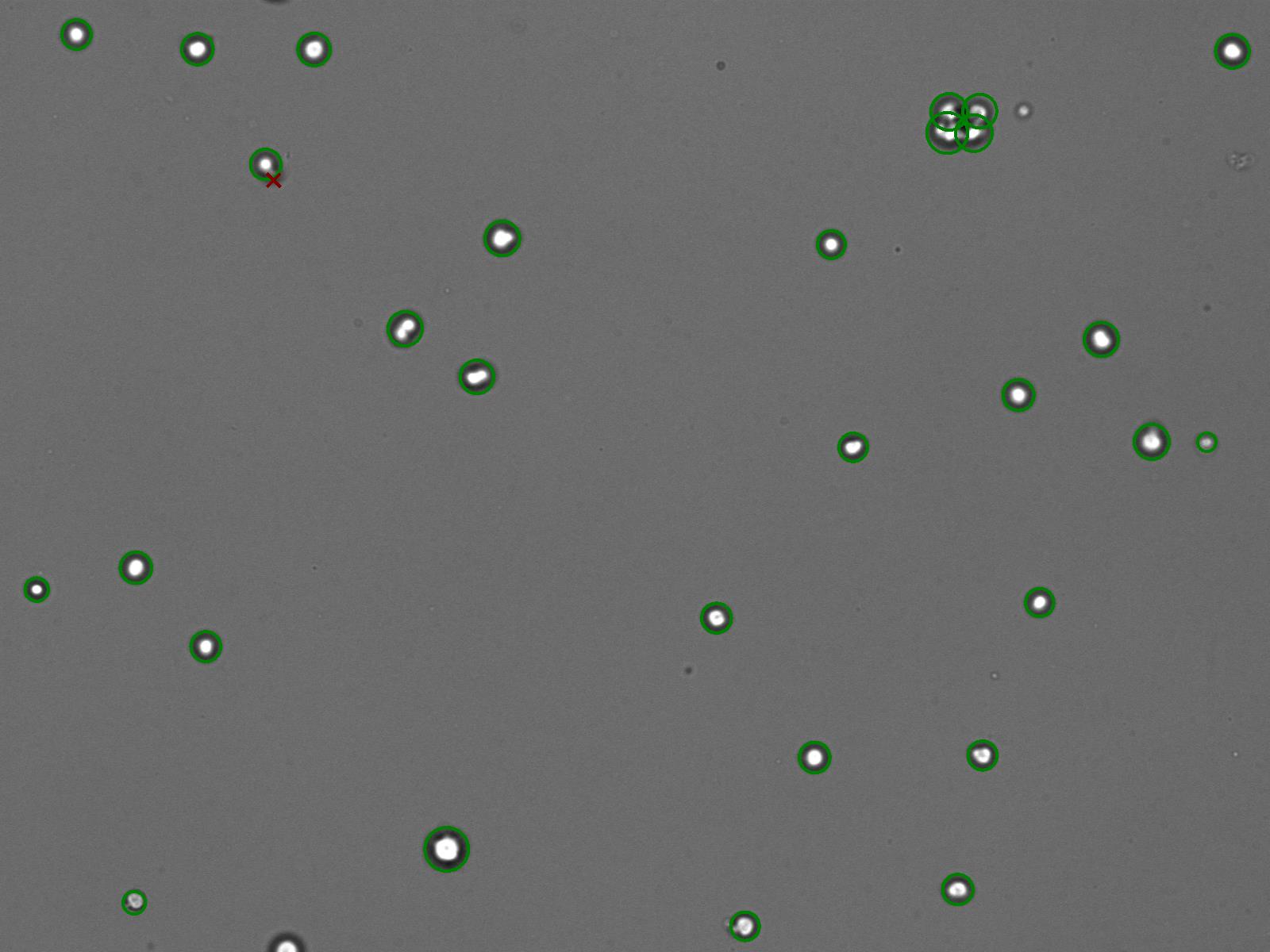

Supplement: Supplementary file 1 — Supplementary Information 1. [file 41598_2020_80576_MOESM1_ESM.zip › S1/Aggregate counts/day5/0mmHg Jan10 41 39/ML SS1 3-003_2019-02-19_112515.bmp]

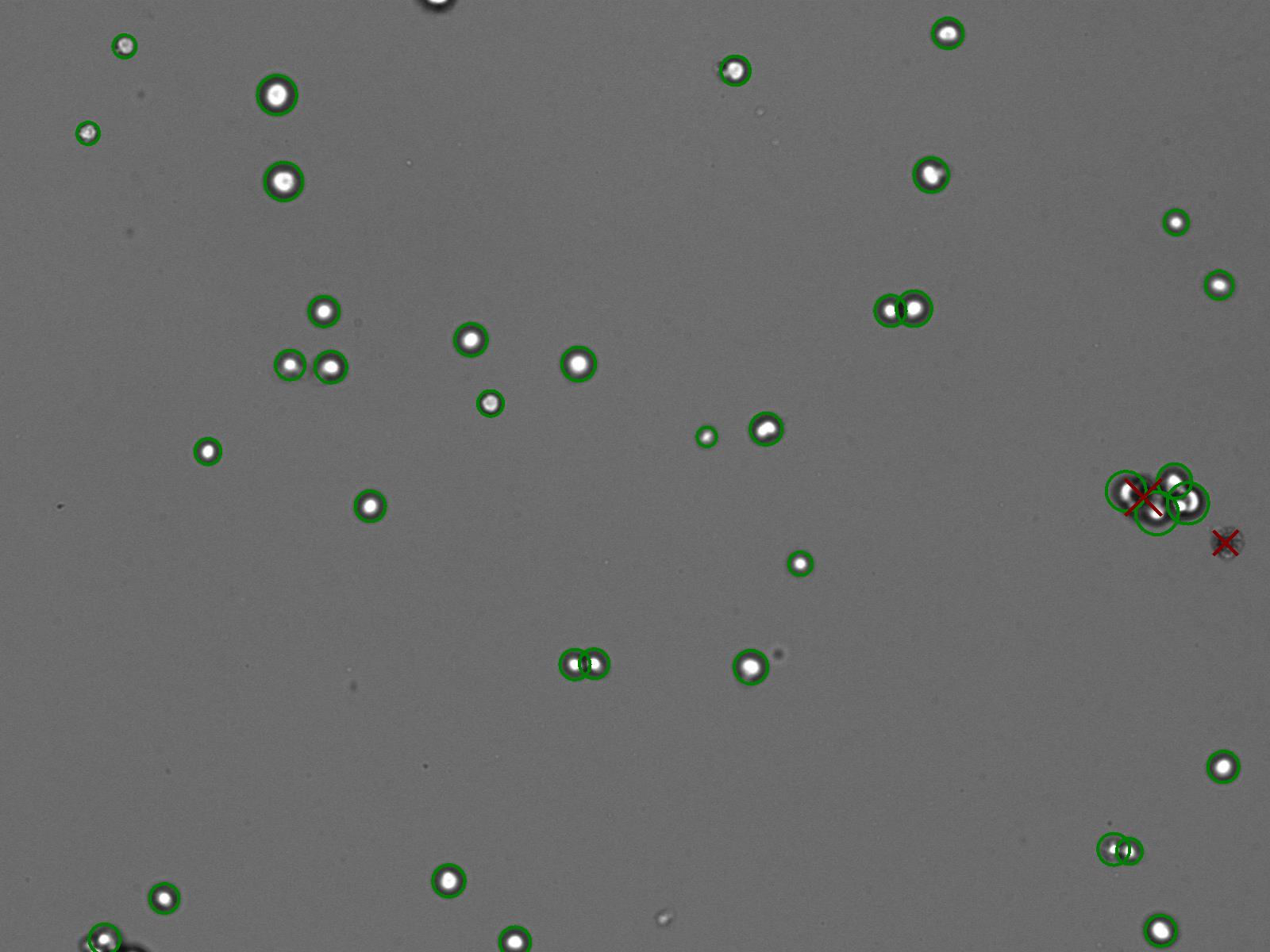

Supplement: Supplementary file 1 — Supplementary Information 1. [file 41598_2020_80576_MOESM1_ESM.zip › S1/Aggregate counts/day5/0mmHg Jan10 41 39/ML SS1 3-004_2019-02-19_112515.bmp]

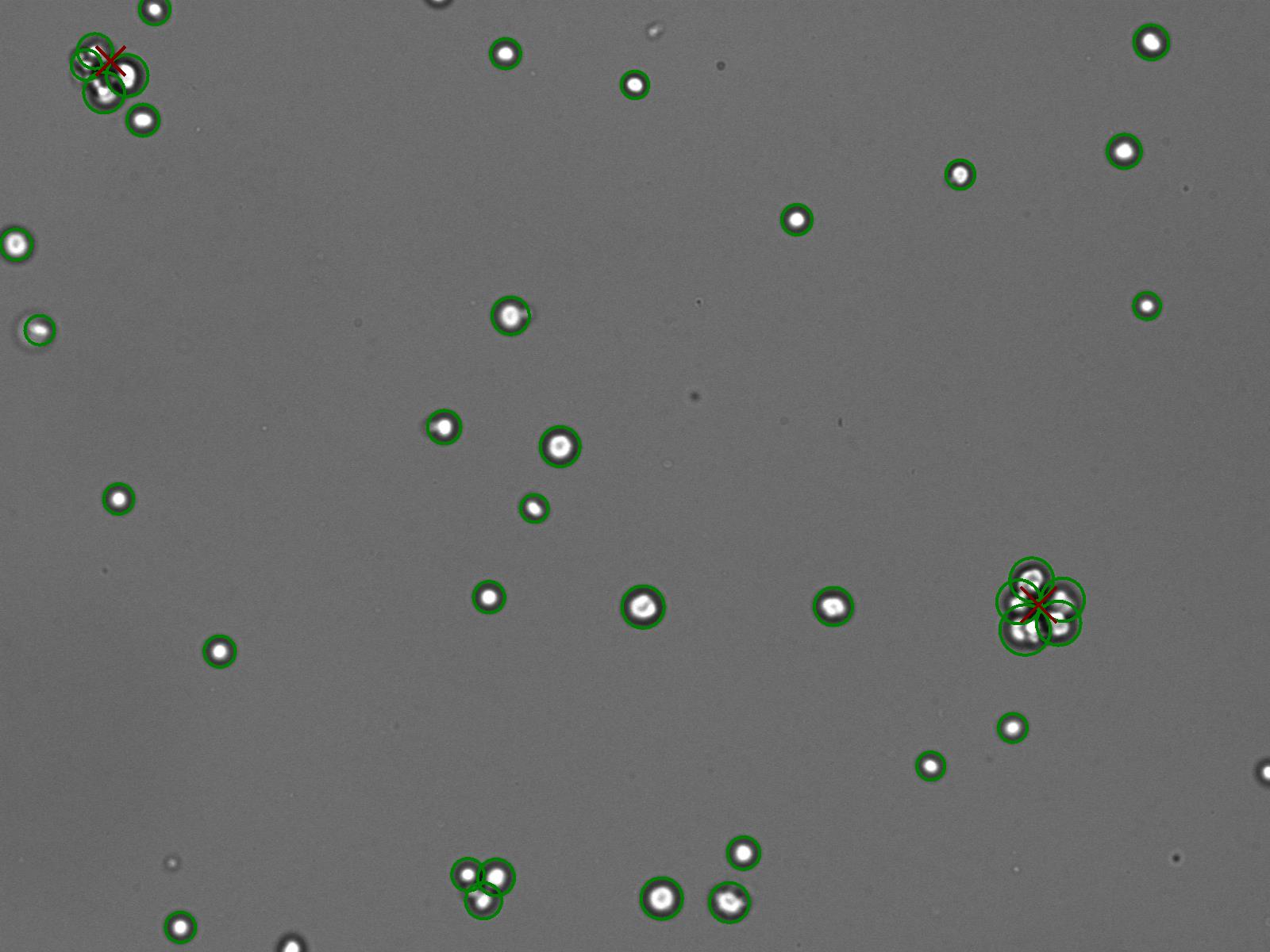

Supplement: Supplementary file 1 — Supplementary Information 1. [file 41598_2020_80576_MOESM1_ESM.zip › S1/Aggregate counts/day5/0mmHg Jan10 41 39/ML SS1 3-005_2019-02-19_112515.bmp]

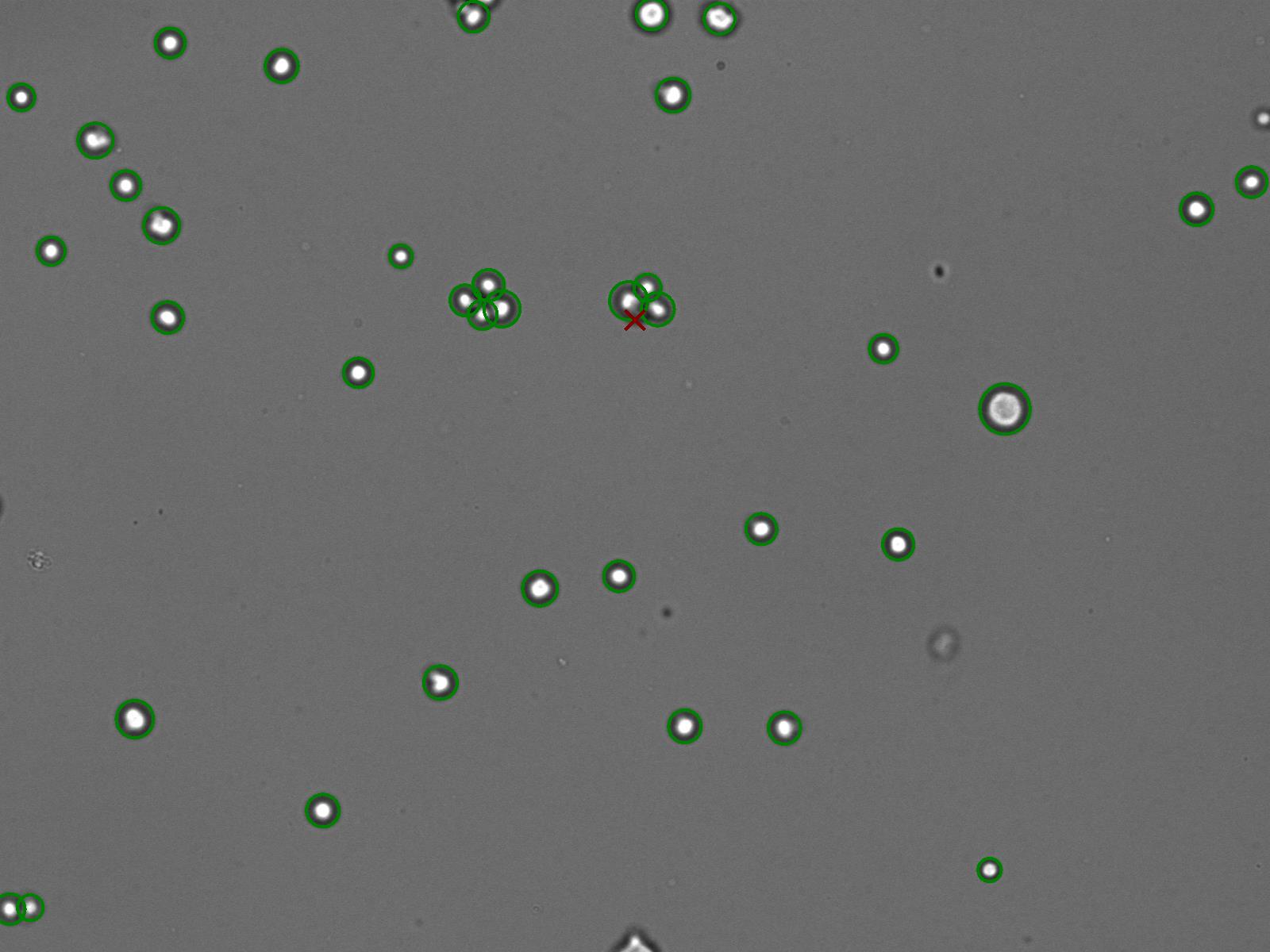

Supplement: Supplementary file 1 — Supplementary Information 1. [file 41598_2020_80576_MOESM1_ESM.zip › S1/Aggregate counts/day5/0mmHg Jan10 41 39/ML SS1 3-006_2019-02-19_112516.bmp]

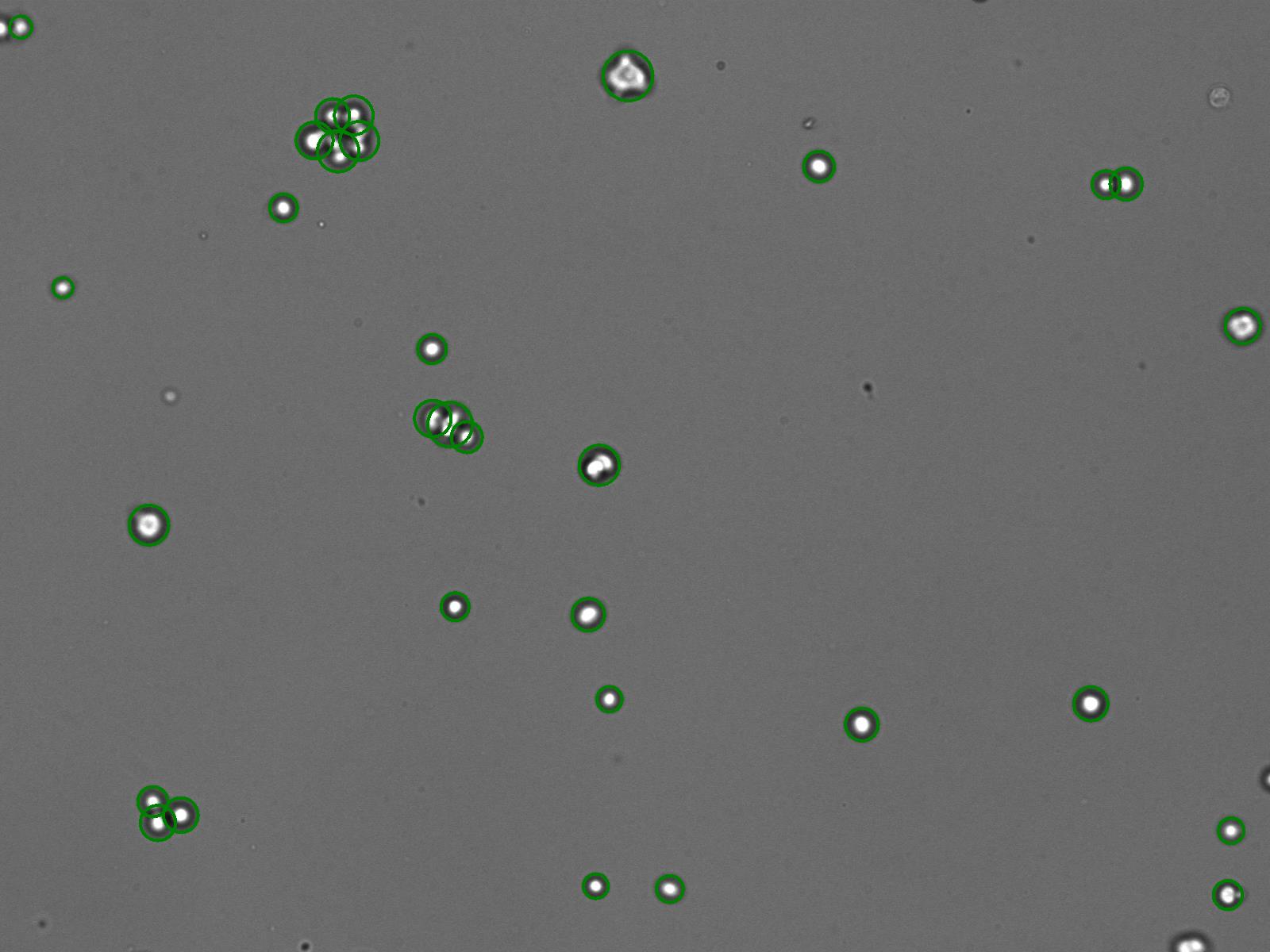

Supplement: Supplementary file 1 — Supplementary Information 1. [file 41598_2020_80576_MOESM1_ESM.zip › S1/Aggregate counts/day5/0mmHg Jan10 41 39/ML SS1 3-007_2019-02-19_112516.bmp]

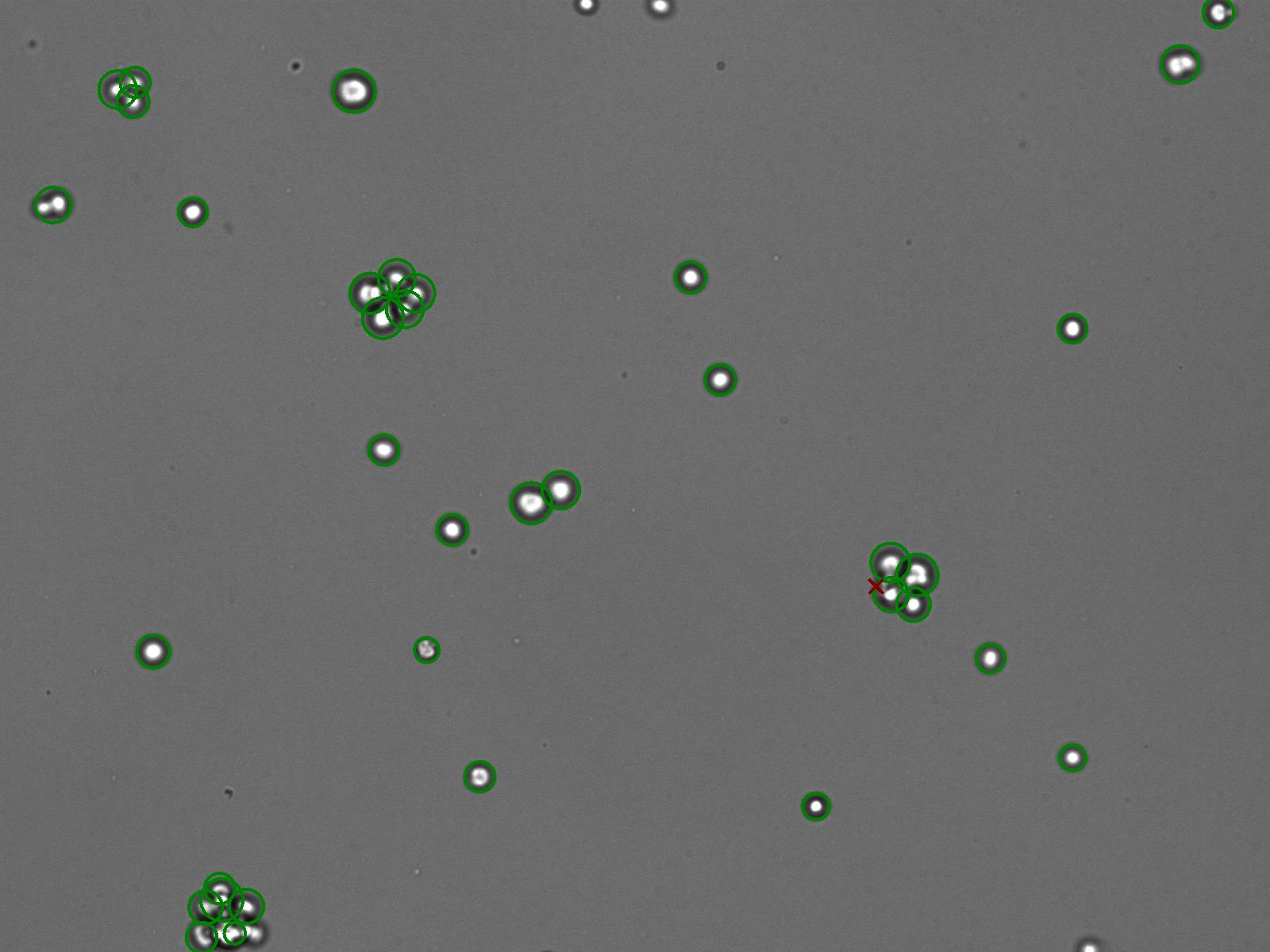

Supplement: Supplementary file 1 — Supplementary Information 1. [file 41598_2020_80576_MOESM1_ESM.zip › S1/Aggregate counts/day5/0mmHg Jan10 41 39/ML SS1 3-008_2019-02-19_112516.bmp]

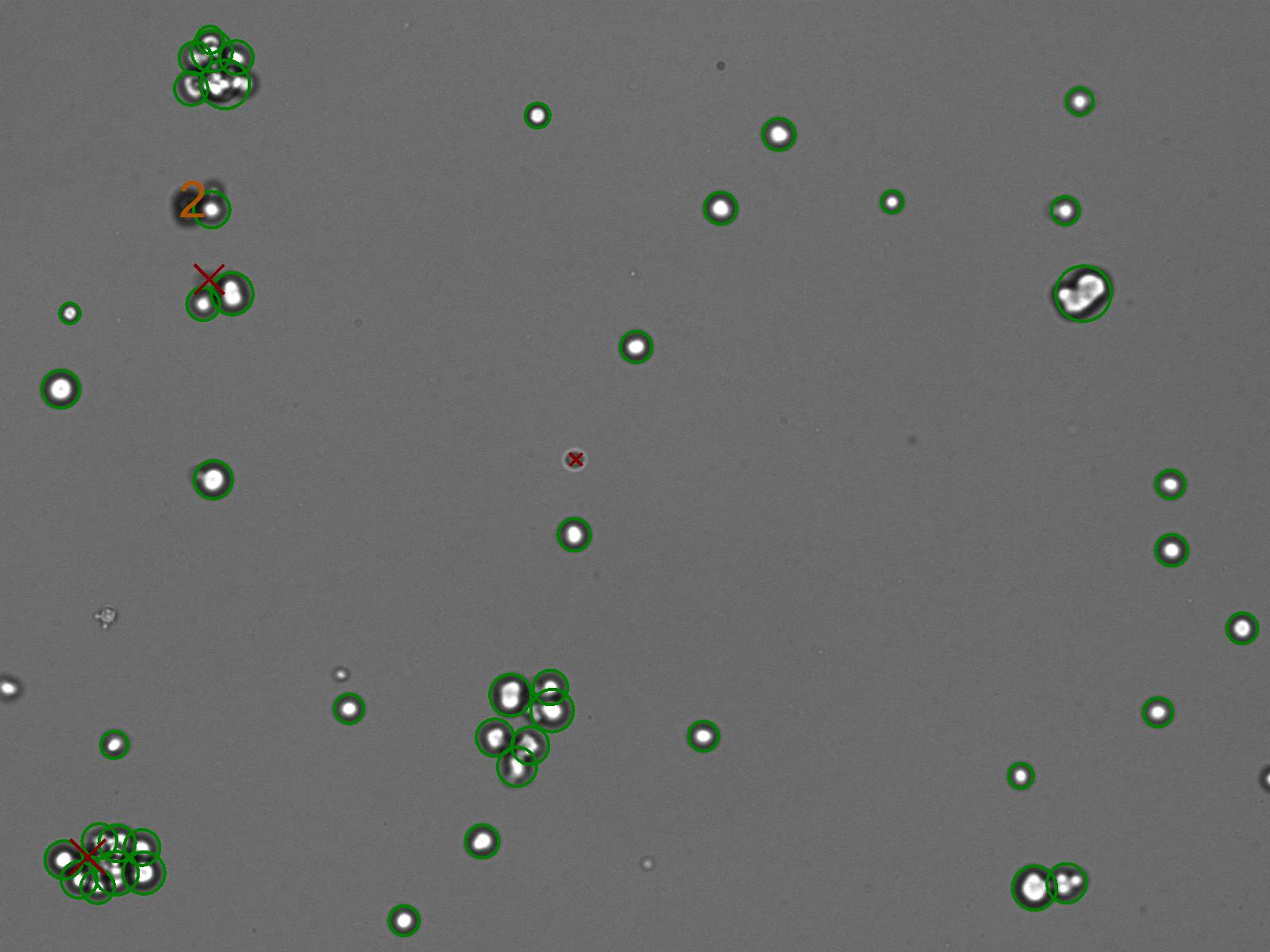

Supplement: Supplementary file 1 — Supplementary Information 1. [file 41598_2020_80576_MOESM1_ESM.zip › S1/Aggregate counts/day5/0mmHg Jan10 41 39/ML SS1 3-009_2019-02-19_112516.bmp]

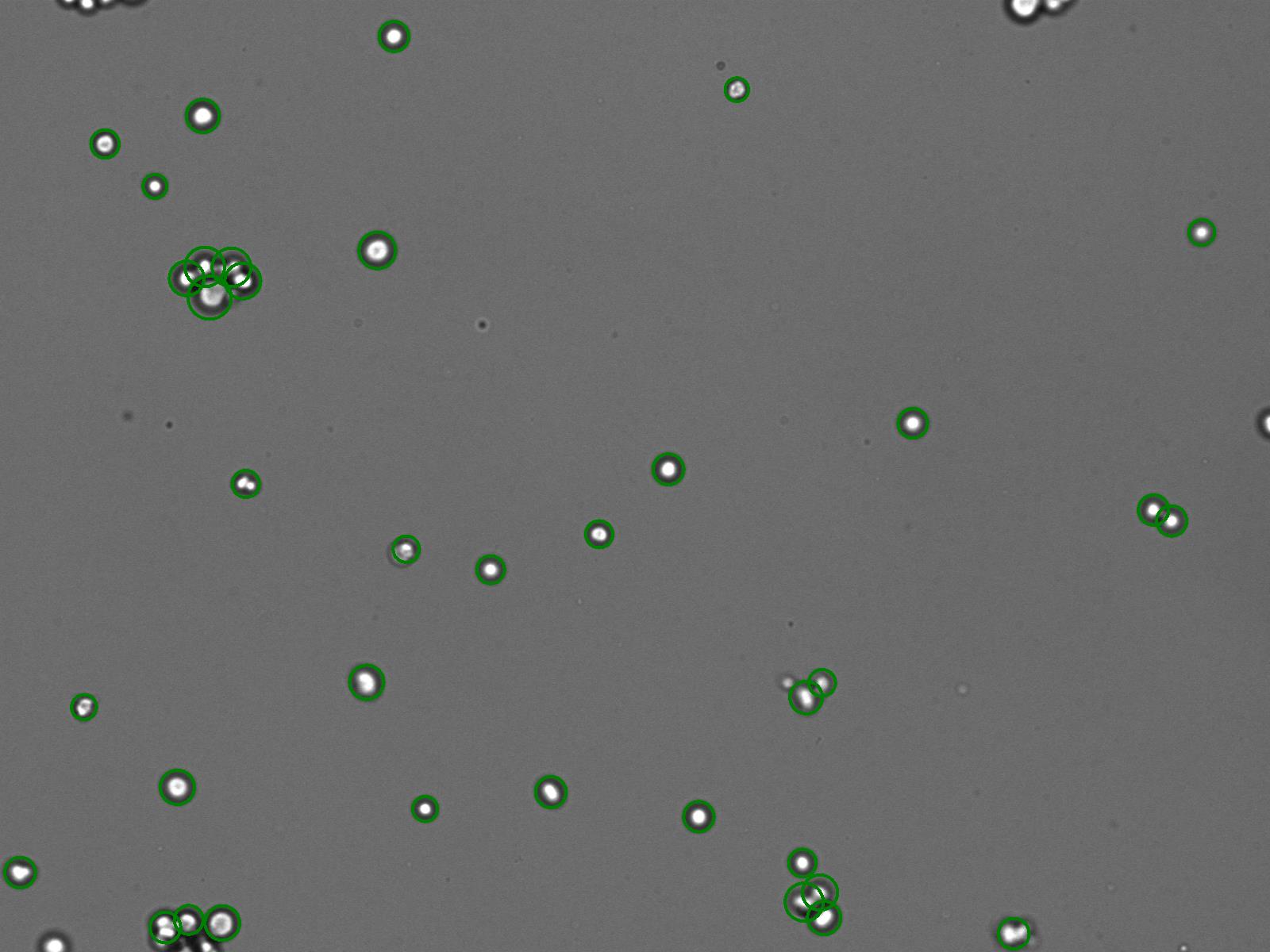

Supplement: Supplementary file 1 — Supplementary Information 1. [file 41598_2020_80576_MOESM1_ESM.zip › S1/Aggregate counts/day5/0mmHg Jan10 41 39/ML SS1 3-010_2019-02-19_112517.bmp]

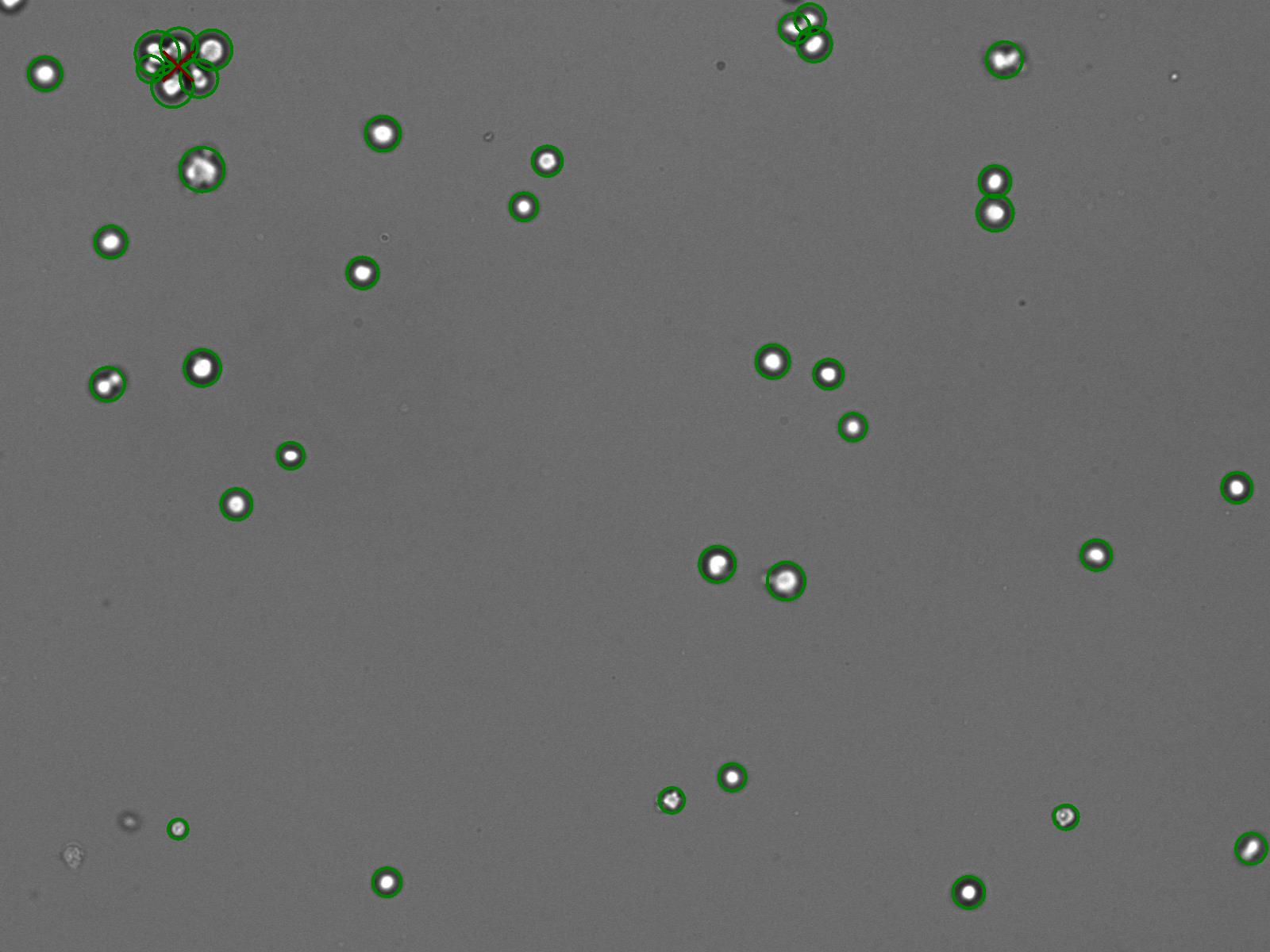

Supplement: Supplementary file 1 — Supplementary Information 1. [file 41598_2020_80576_MOESM1_ESM.zip › S1/Aggregate counts/day5/0mmHg Jan10 41 39/ML SS1 3-011_2019-02-19_112517.bmp]

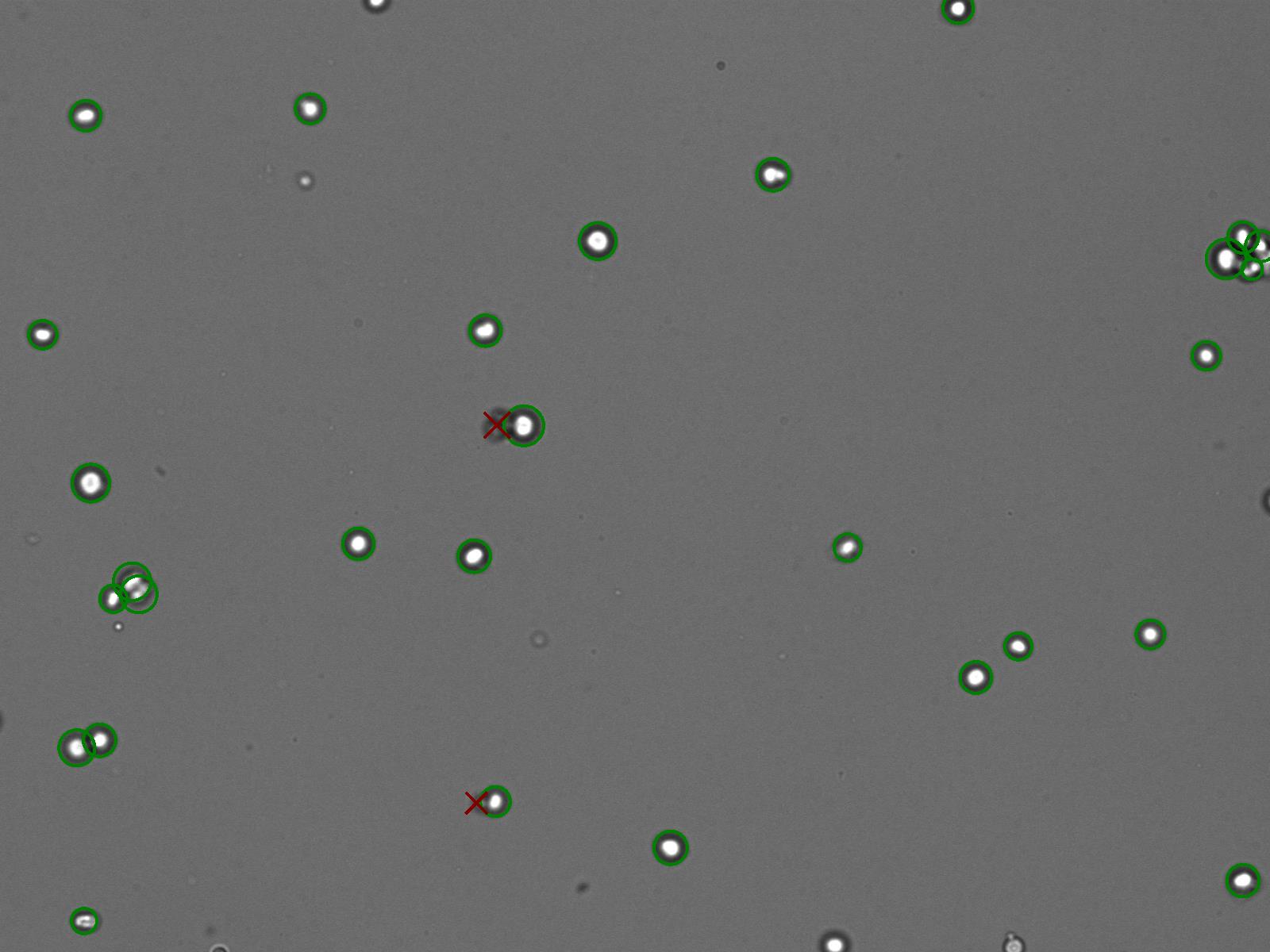

Supplement: Supplementary file 1 — Supplementary Information 1. [file 41598_2020_80576_MOESM1_ESM.zip › S1/Aggregate counts/day5/0mmHg Jan10 41 39/ML SS1 3-012_2019-02-19_112517.bmp]

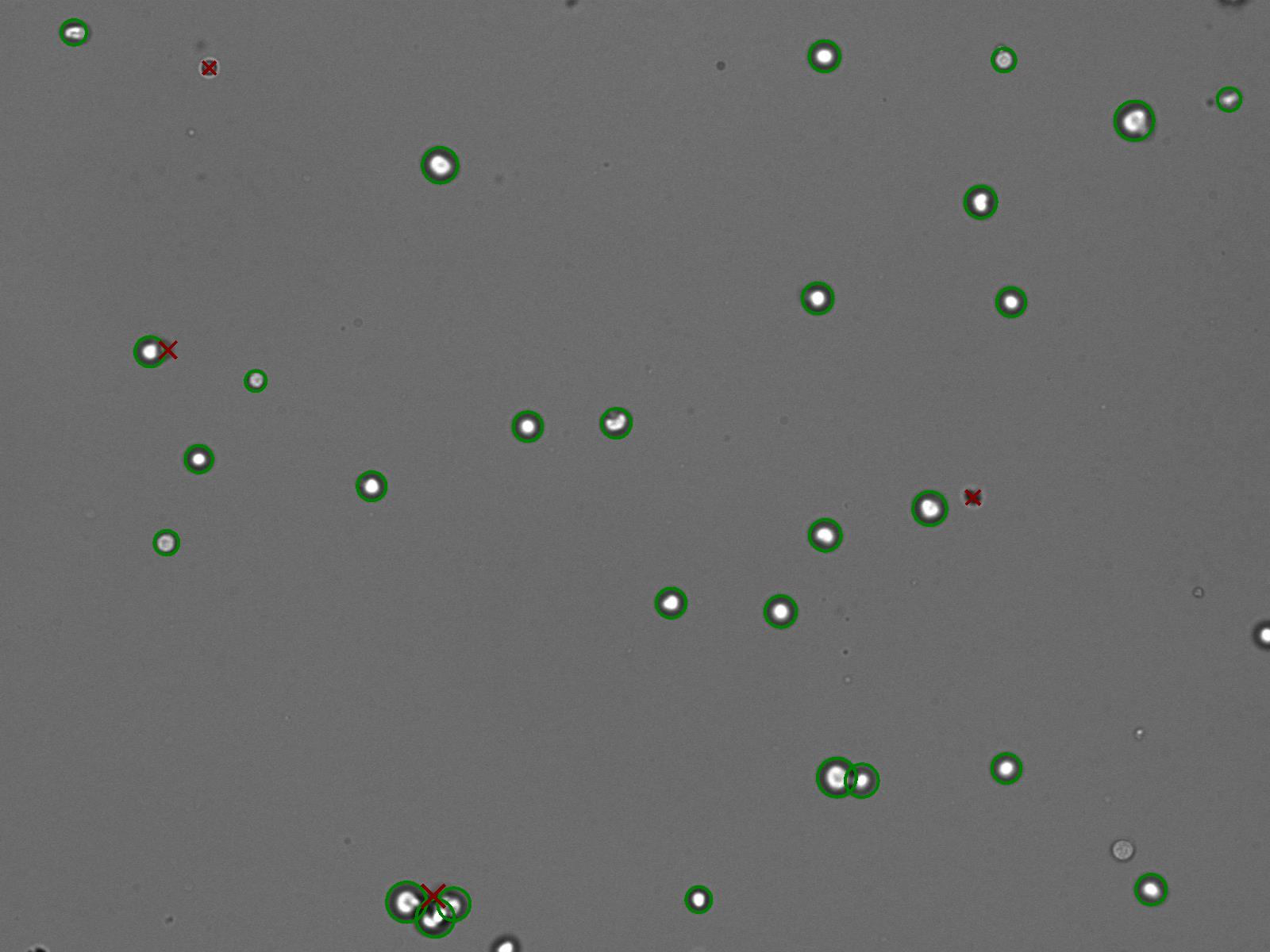

Supplement: Supplementary file 1 — Supplementary Information 1. [file 41598_2020_80576_MOESM1_ESM.zip › S1/Aggregate counts/day5/0mmHg Jan10 41 39/ML SS1 3-013_2019-02-19_112517.bmp]

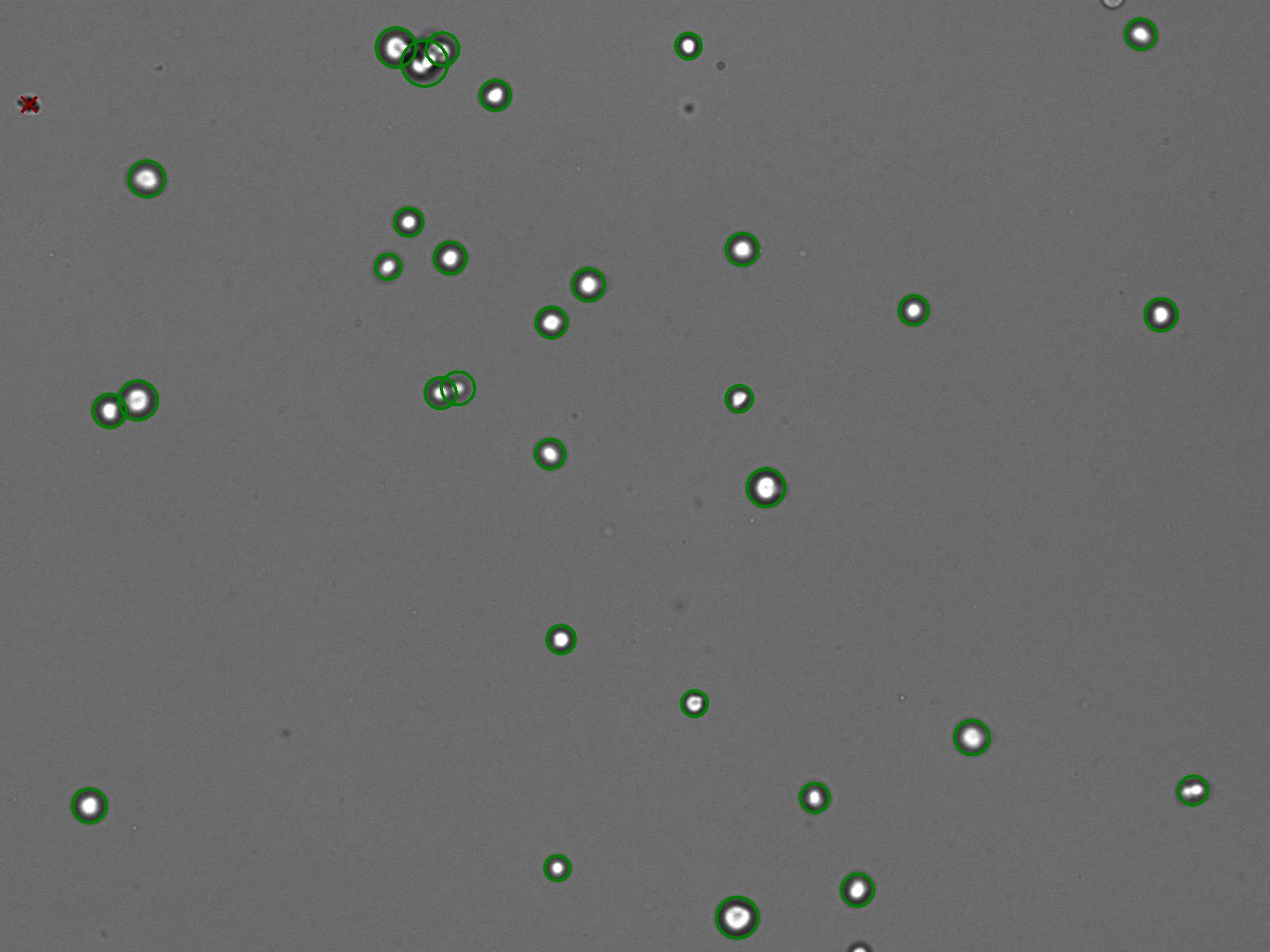

Supplement: Supplementary file 1 — Supplementary Information 1. [file 41598_2020_80576_MOESM1_ESM.zip › S1/Aggregate counts/day5/0mmHg Jan10 41 39/ML SS1 3-014_2019-02-19_112518.bmp]

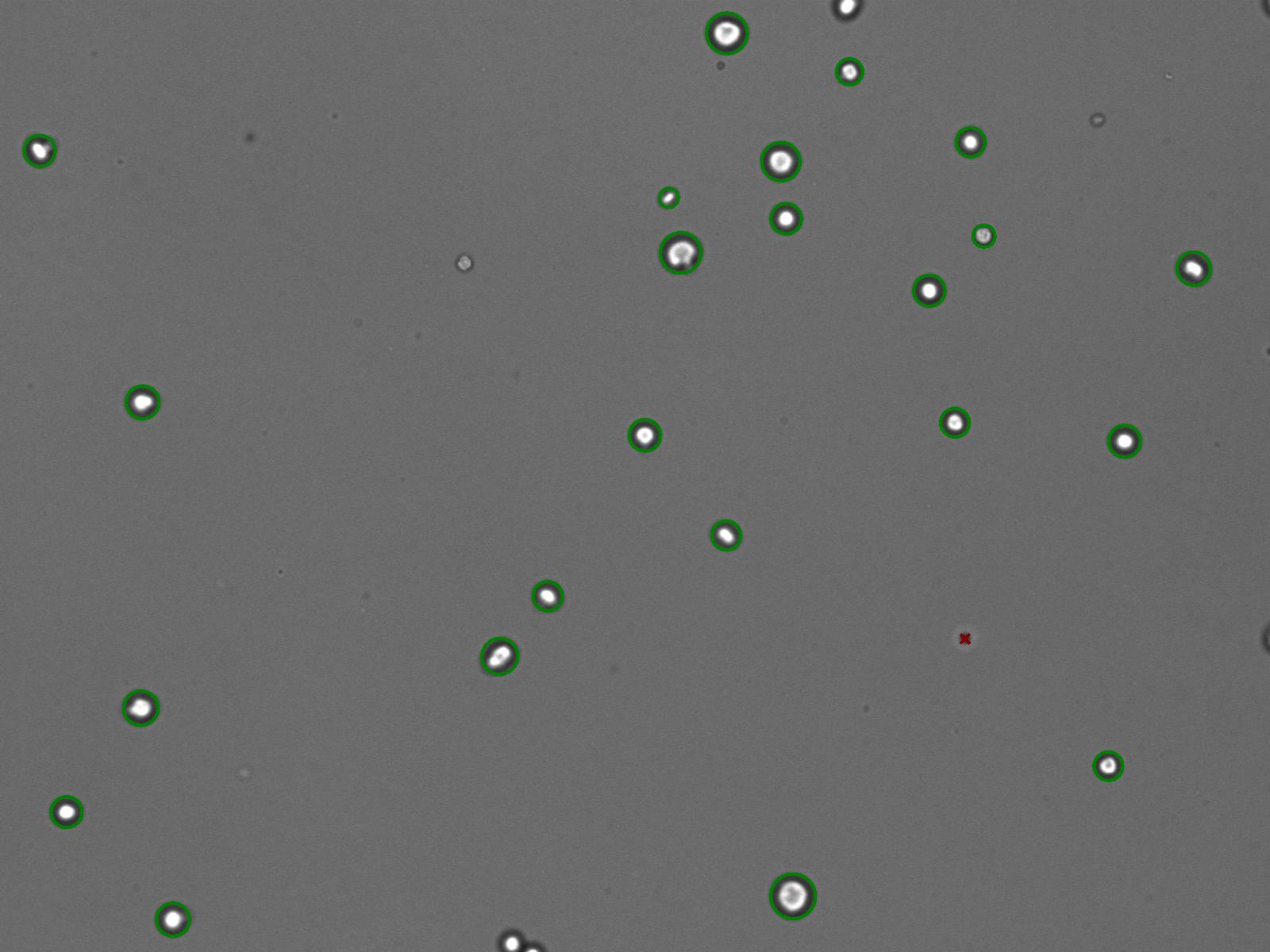

Supplement: Supplementary file 1 — Supplementary Information 1. [file 41598_2020_80576_MOESM1_ESM.zip › S1/Aggregate counts/day5/0mmHg Jan10 41 39/ML SS1 3-015_2019-02-19_112518.bmp]

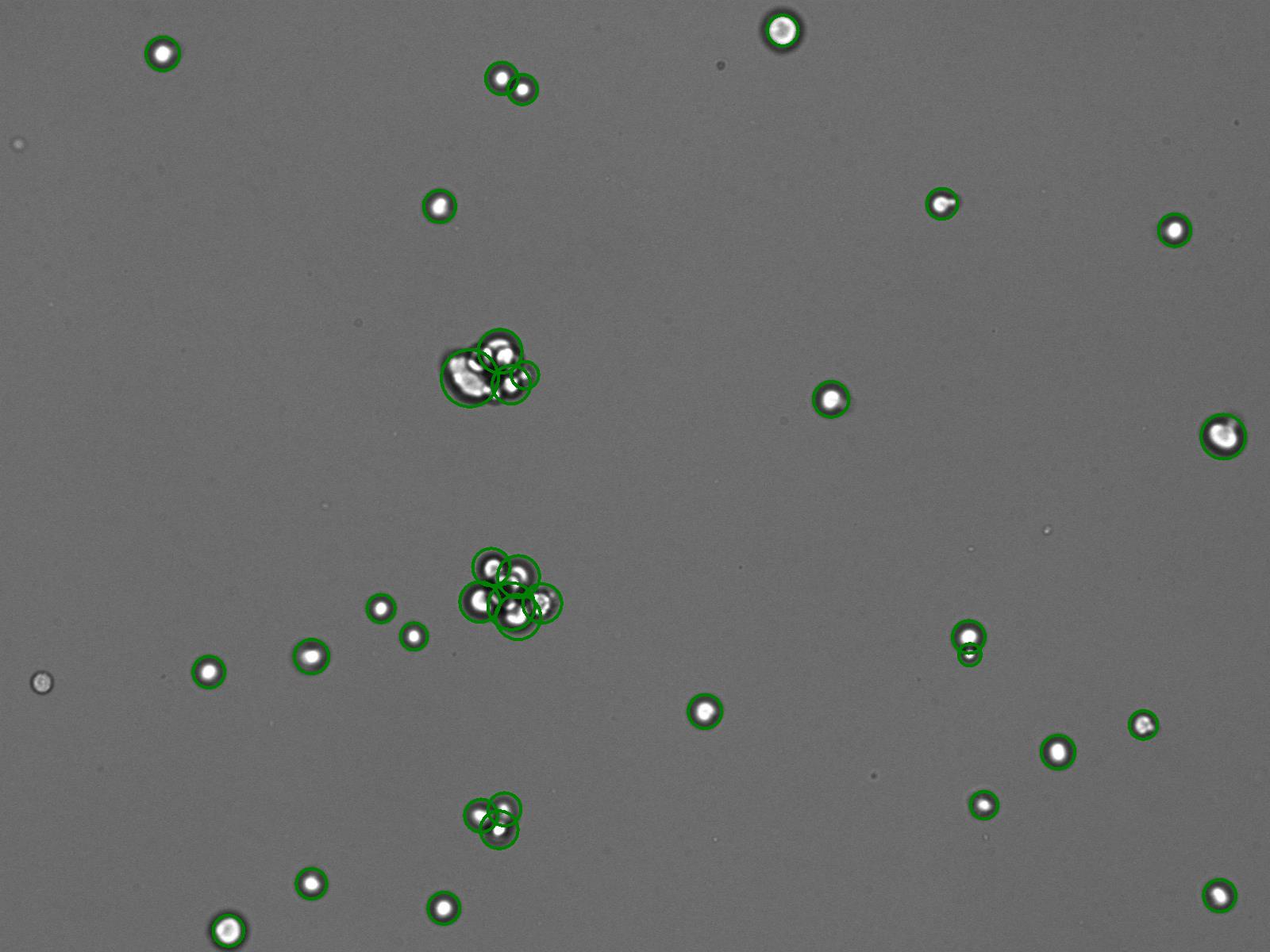

Supplement: Supplementary file 1 — Supplementary Information 1. [file 41598_2020_80576_MOESM1_ESM.zip › S1/Aggregate counts/day5/0mmHg Jan10 41 39/ML SS1 3-016_2019-02-19_112518.bmp]

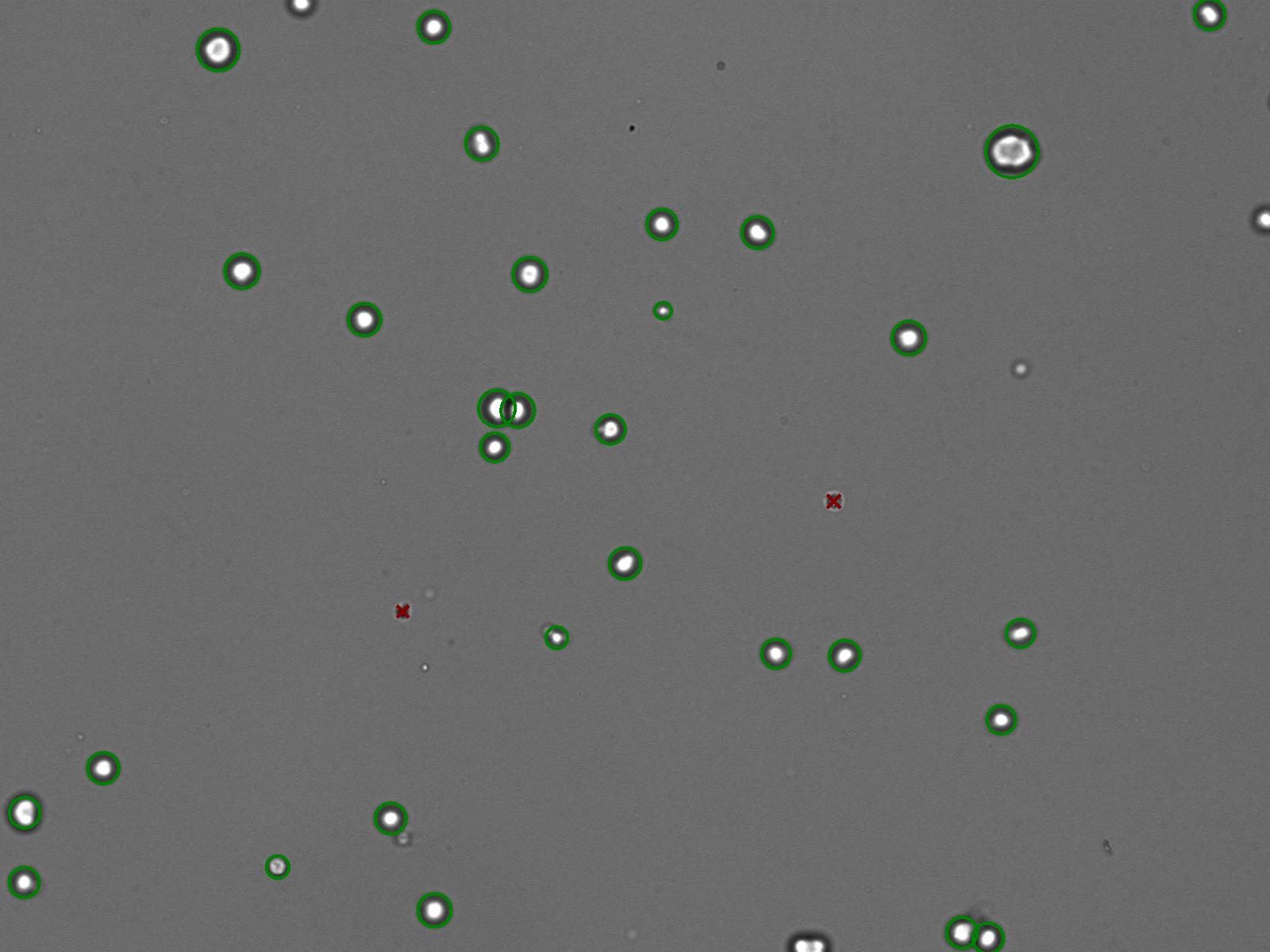

Supplement: Supplementary file 1 — Supplementary Information 1. [file 41598_2020_80576_MOESM1_ESM.zip › S1/Aggregate counts/day5/0mmHg Jan10 41 39/ML SS1 3-017_2019-02-19_112519.bmp]

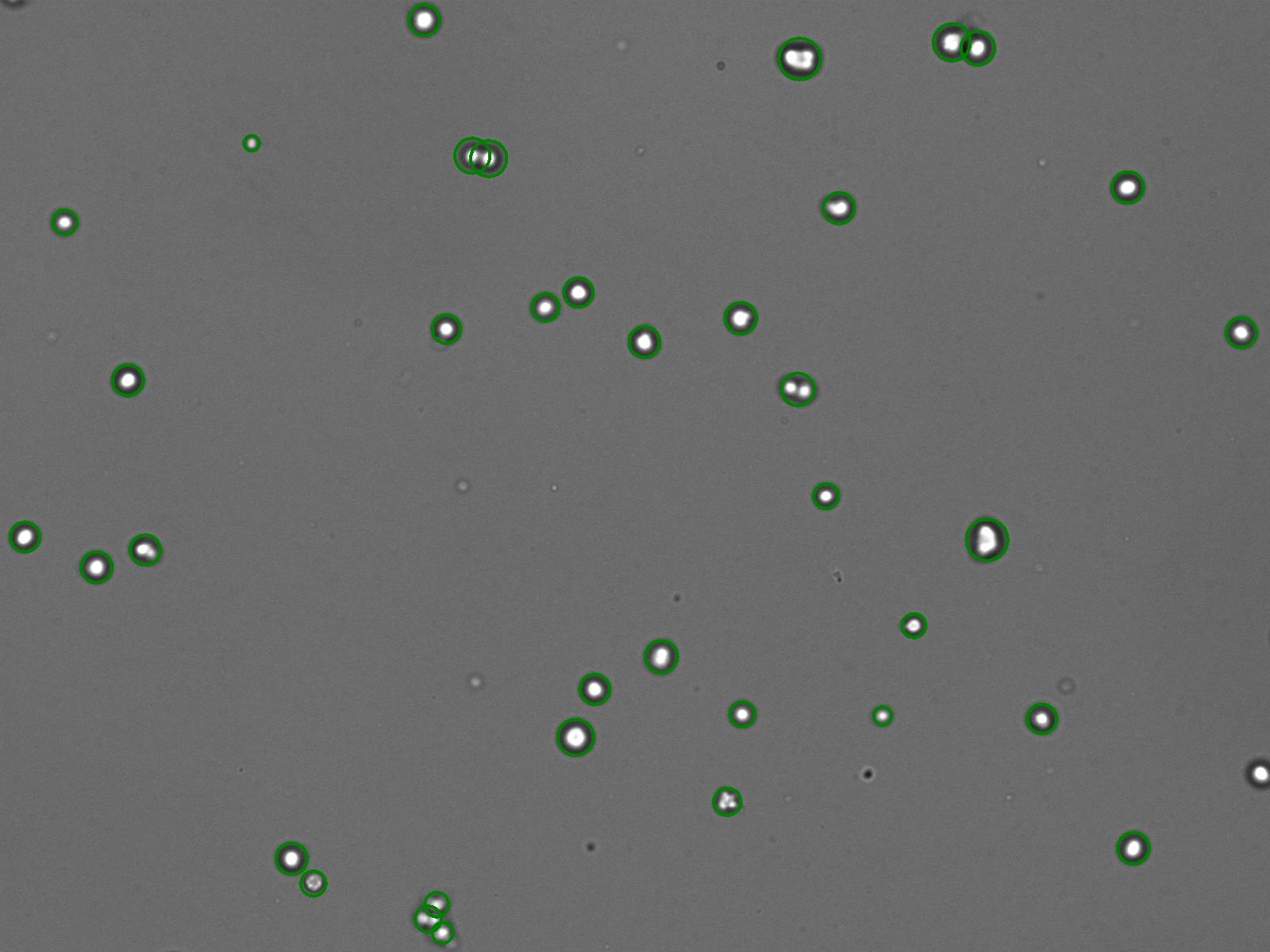

Supplement: Supplementary file 1 — Supplementary Information 1. [file 41598_2020_80576_MOESM1_ESM.zip › S1/Aggregate counts/day5/0mmHg Jan10 41 39/ML SS1 3-018_2019-02-19_112519.bmp]

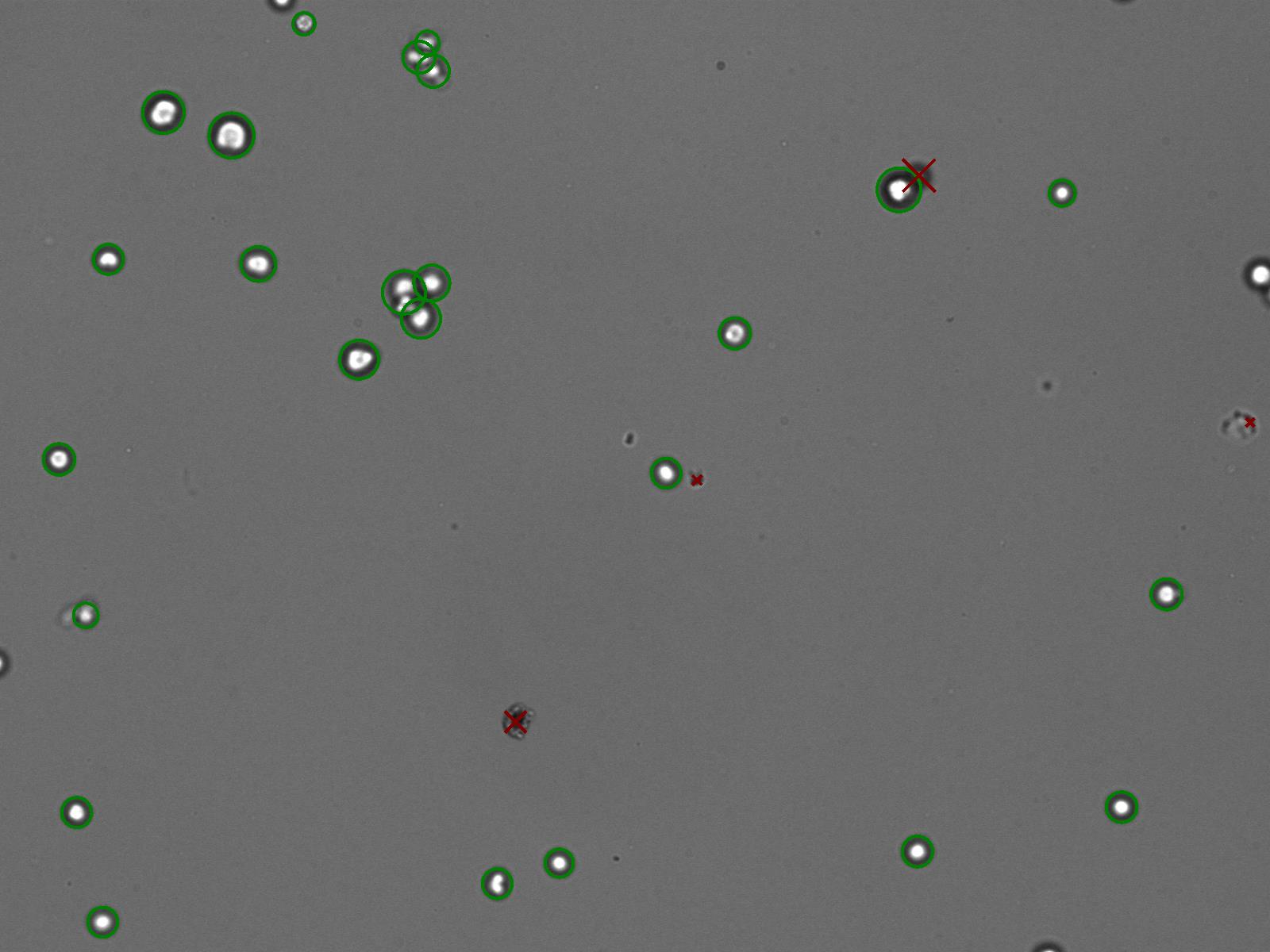

Supplement: Supplementary file 1 — Supplementary Information 1. [file 41598_2020_80576_MOESM1_ESM.zip › S1/Aggregate counts/day5/0mmHg Jan10 41 39/ML SS1 3-019_2019-02-19_112519.bmp]

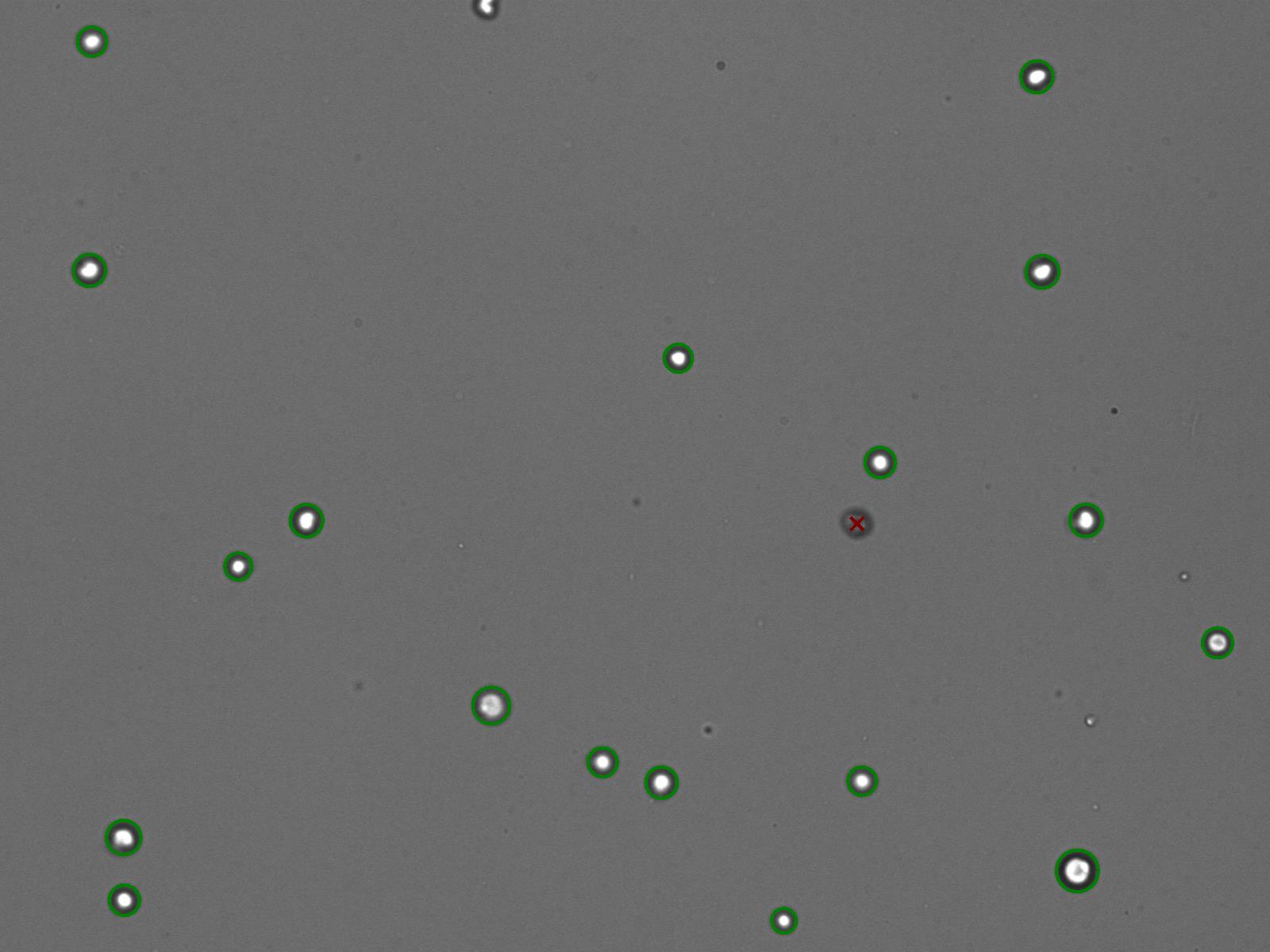

Supplement: Supplementary file 1 — Supplementary Information 1. [file 41598_2020_80576_MOESM1_ESM.zip › S1/Aggregate counts/day5/0mmHg Jan10 41 39/ML SS1 3-020_2019-02-19_112519.bmp]

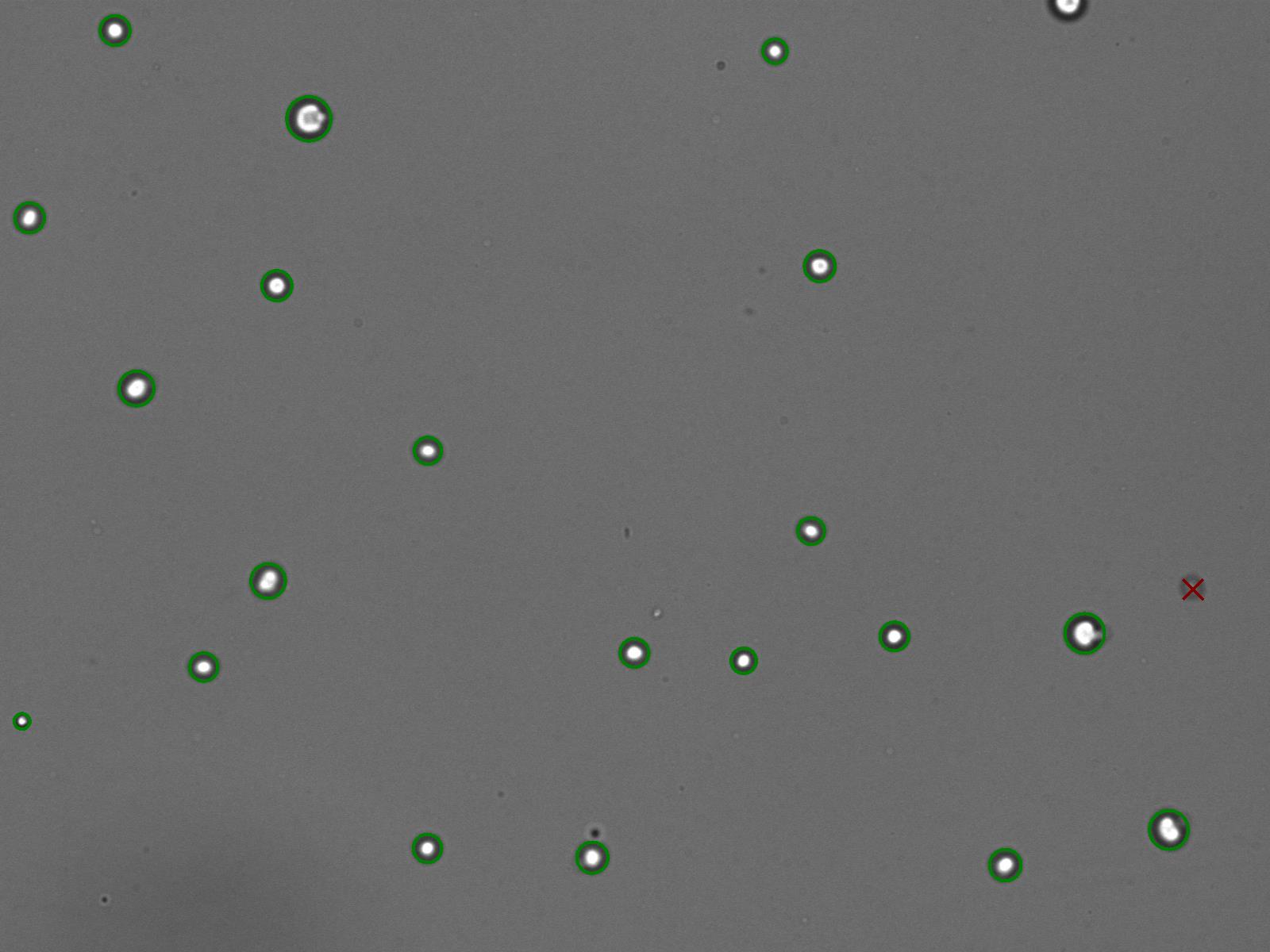

Supplement: Supplementary file 1 — Supplementary Information 1. [file 41598_2020_80576_MOESM1_ESM.zip › S1/Aggregate counts/day5/0mmHg Jan10 41 39/ML SS1 3-021_2019-02-19_112520.bmp]

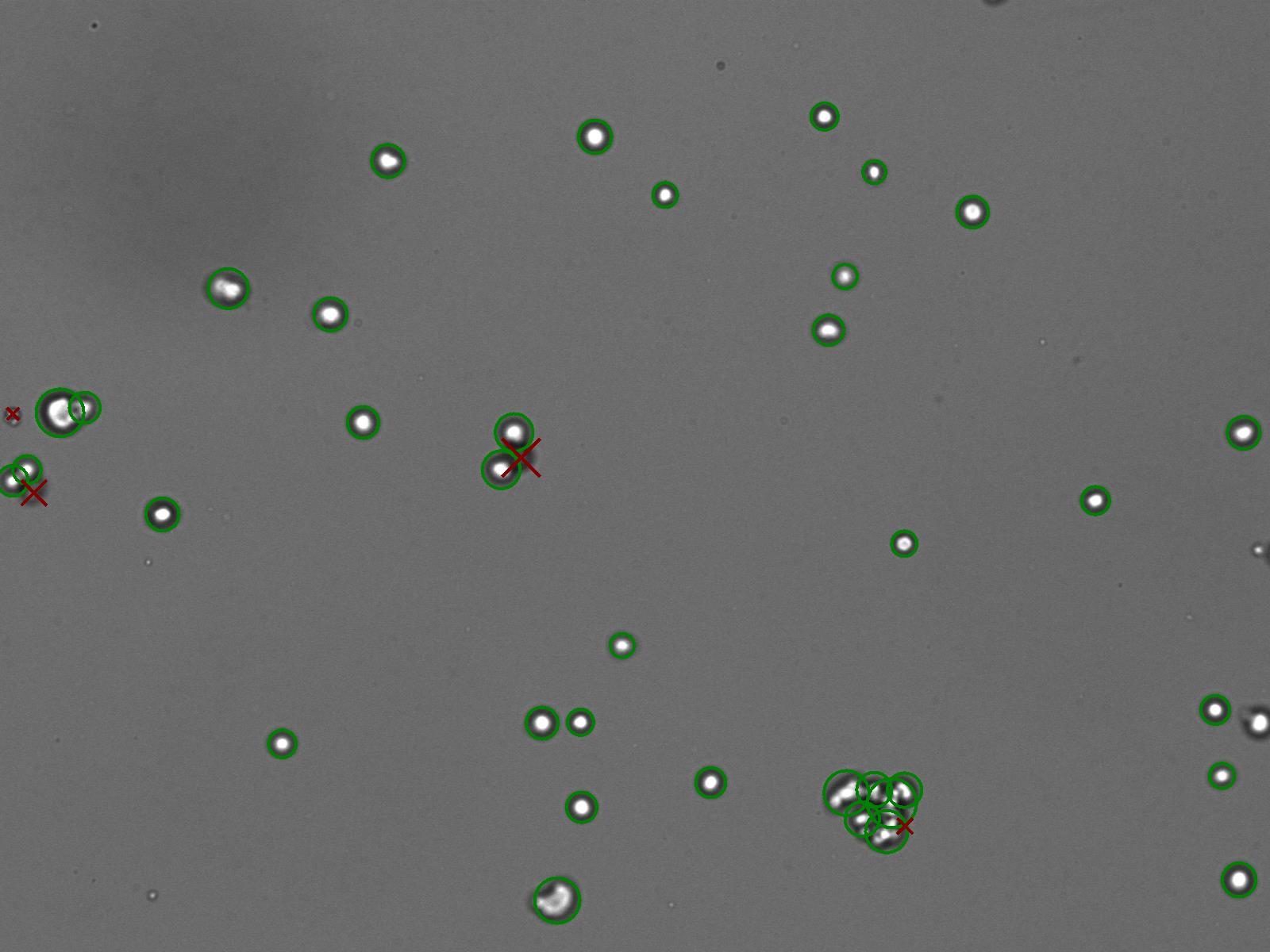

Supplement: Supplementary file 1 — Supplementary Information 1. [file 41598_2020_80576_MOESM1_ESM.zip › S1/Aggregate counts/day5/0mmHg Jan10 41 39/ML SS1 3-022_2019-02-19_112520.bmp]

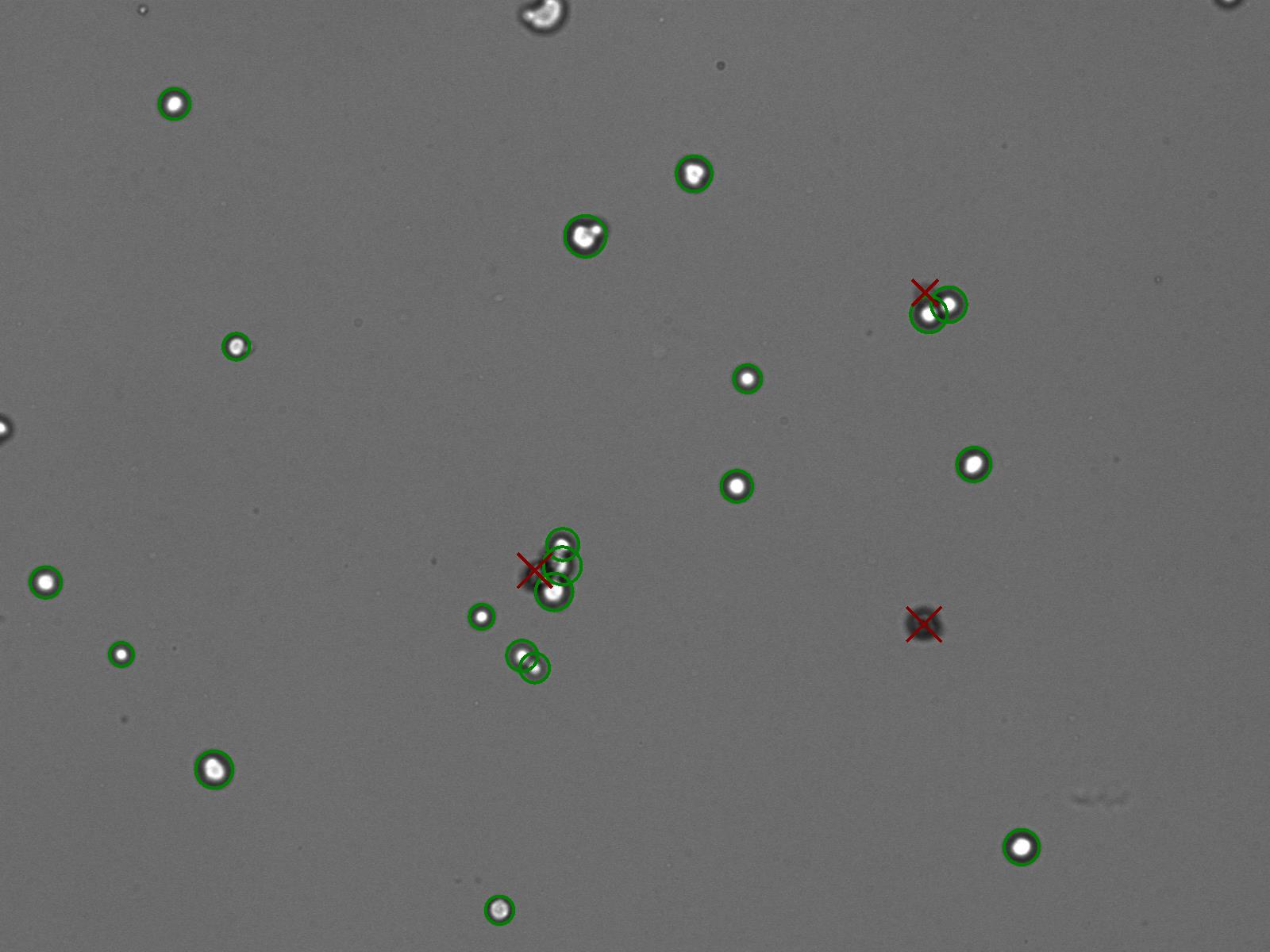

Supplement: Supplementary file 1 — Supplementary Information 1. [file 41598_2020_80576_MOESM1_ESM.zip › S1/Aggregate counts/day5/0mmHg Jan10 41 39/ML SS1 3-023_2019-02-19_112520.bmp]

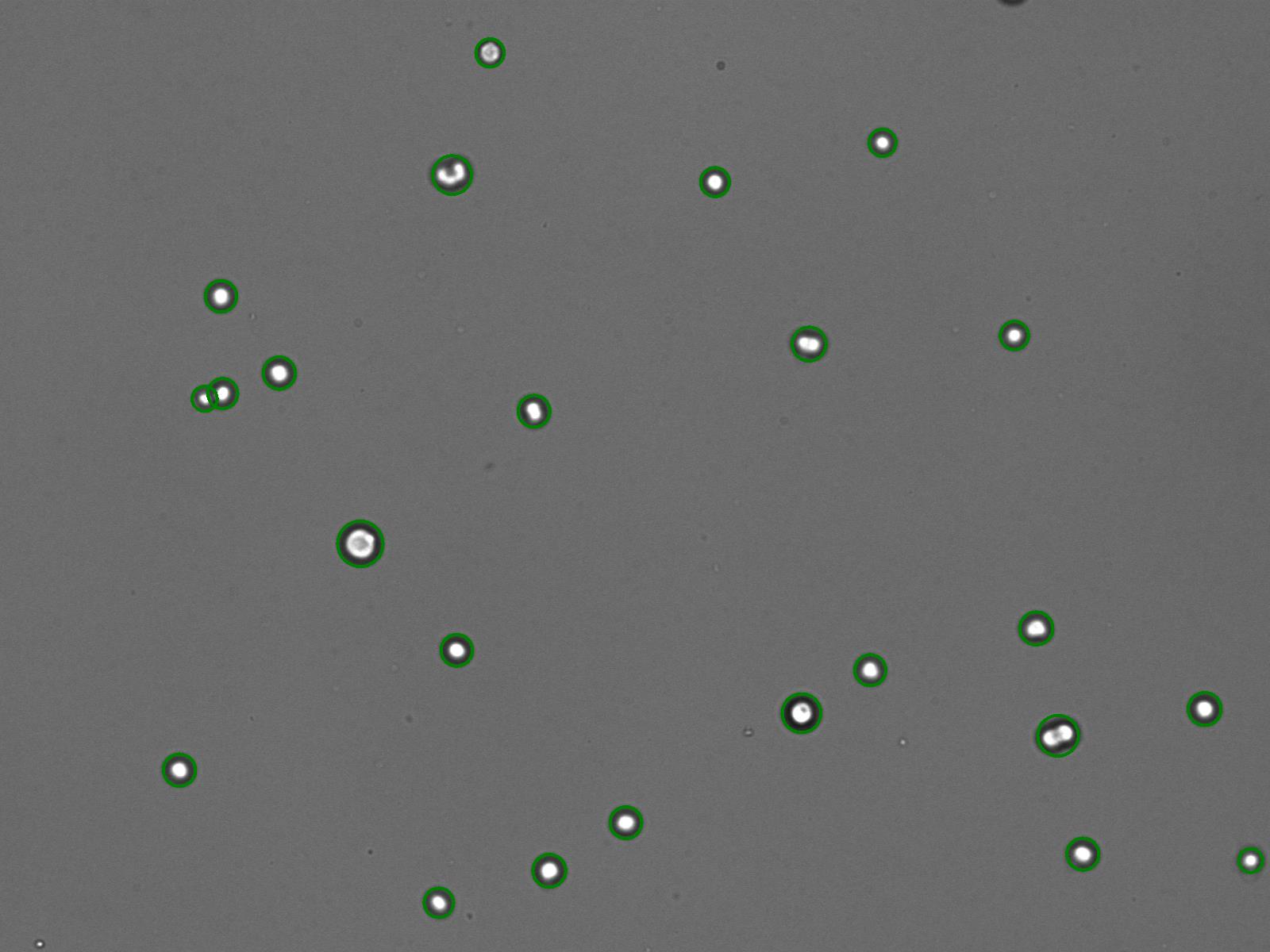

Supplement: Supplementary file 1 — Supplementary Information 1. [file 41598_2020_80576_MOESM1_ESM.zip › S1/Aggregate counts/day5/0mmHg Jan10 41 39/ML SS1 3-024_2019-02-19_112521.bmp]

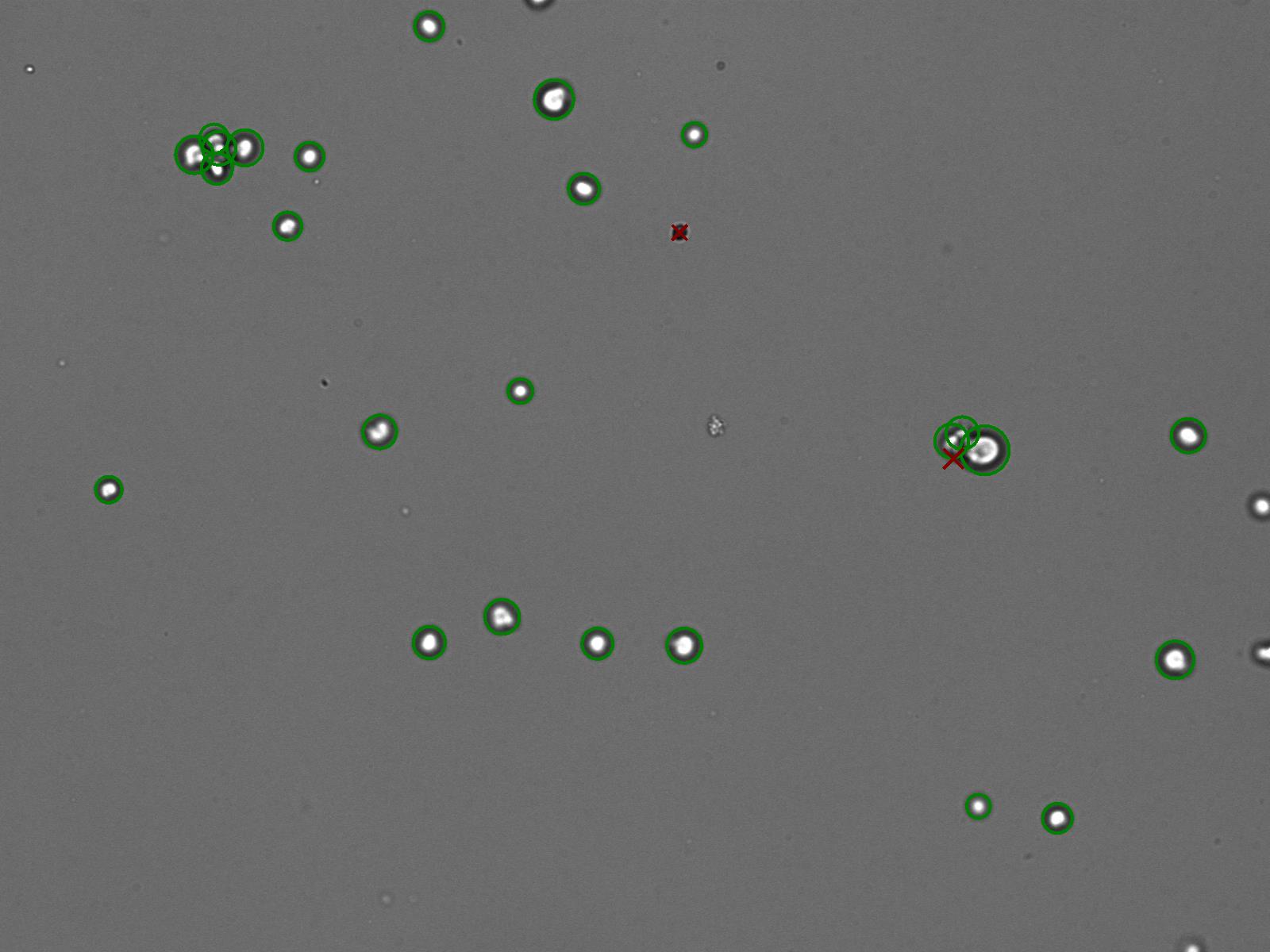

Supplement: Supplementary file 1 — Supplementary Information 1. [file 41598_2020_80576_MOESM1_ESM.zip › S1/Aggregate counts/day5/0mmHg Jan10 41 39/ML SS1 3-025_2019-02-19_112521.bmp]

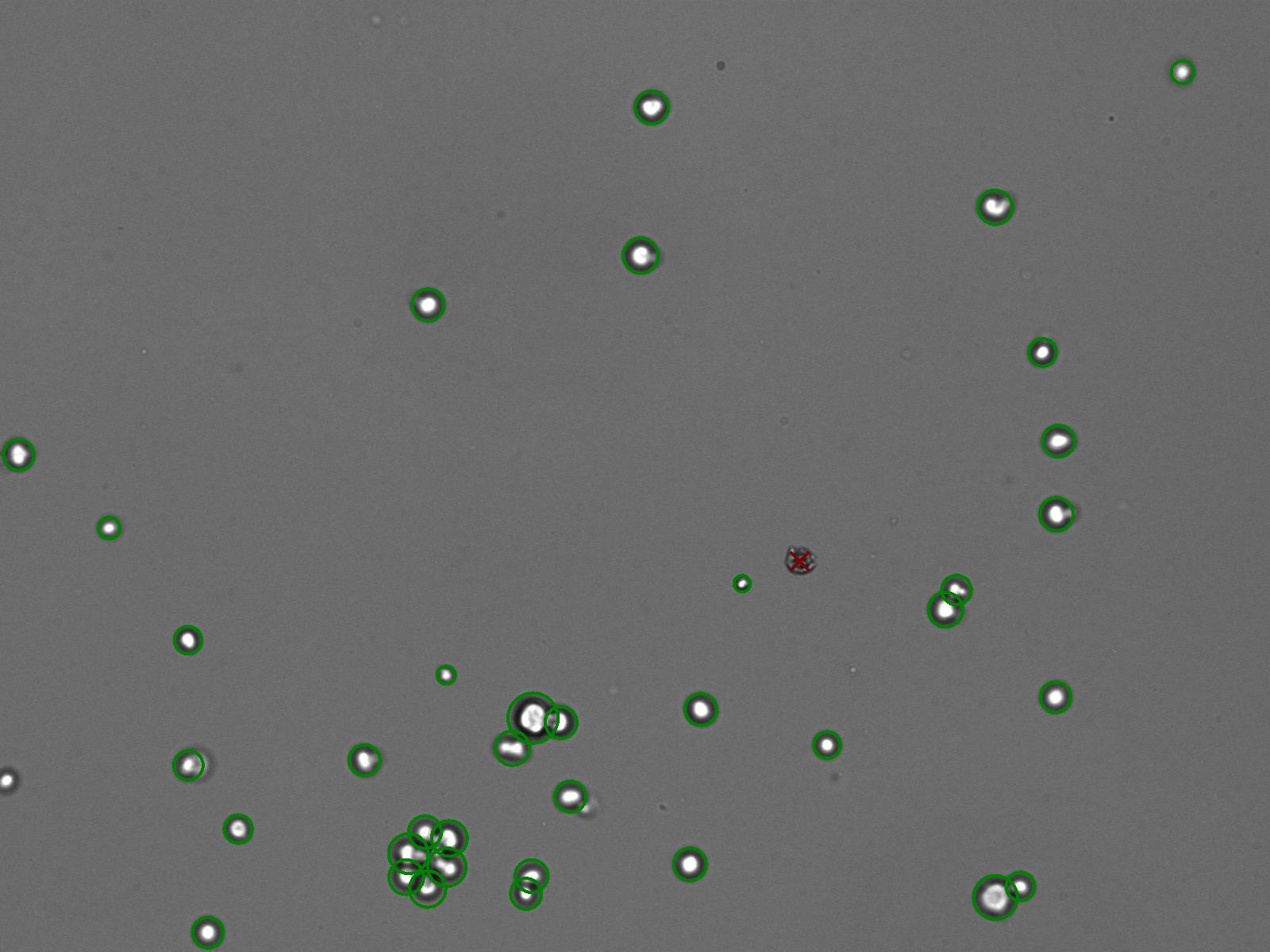

Supplement: Supplementary file 1 — Supplementary Information 1. [file 41598_2020_80576_MOESM1_ESM.zip › S1/Aggregate counts/day5/0mmHg Jan10 41 39/ML SS1 3-026_2019-02-19_112521.bmp]

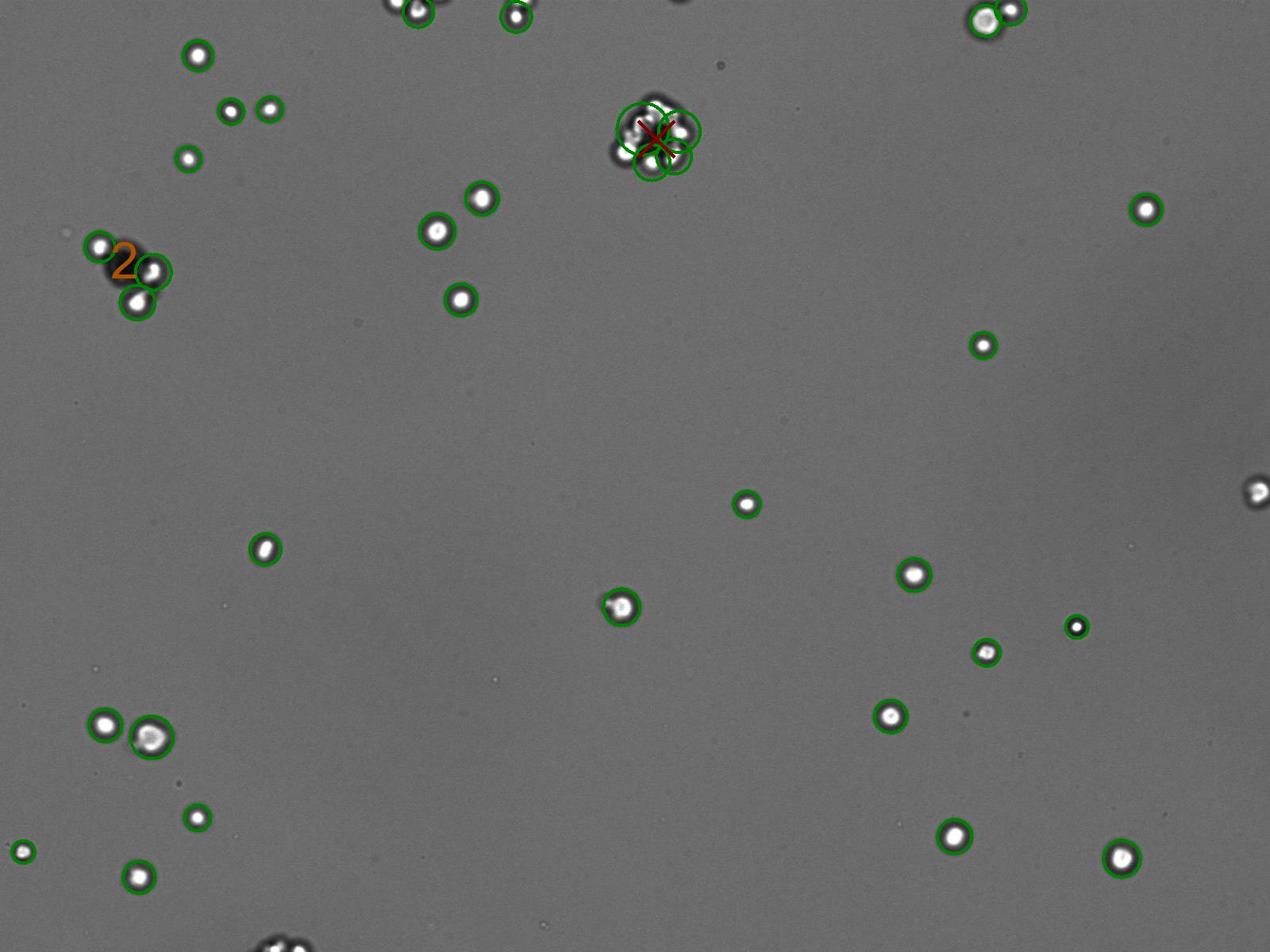

Supplement: Supplementary file 1 — Supplementary Information 1. [file 41598_2020_80576_MOESM1_ESM.zip › S1/Aggregate counts/day5/0mmHg Jan10 41 39/ML SS1 3-027_2019-02-19_112522.bmp]

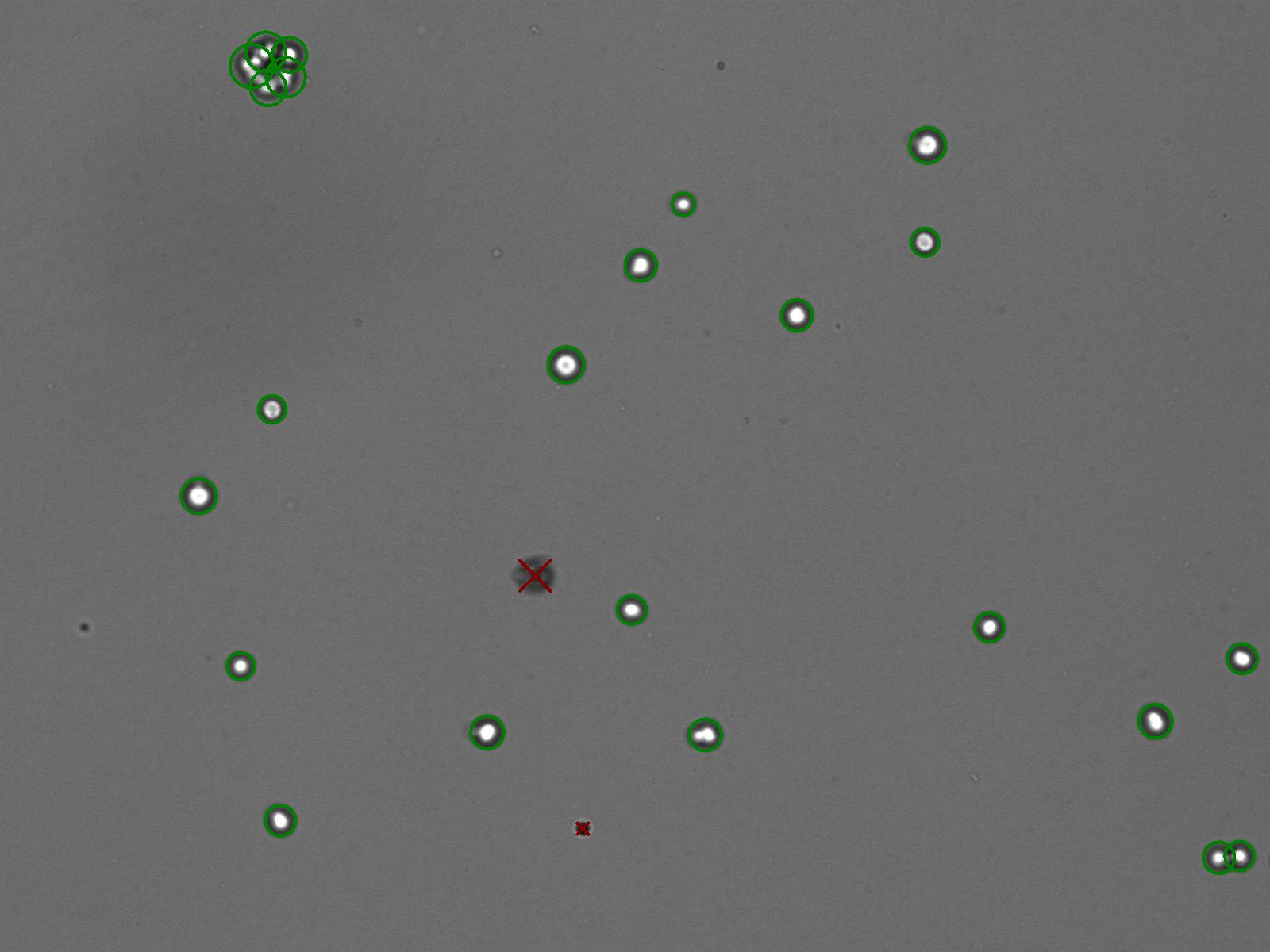

Supplement: Supplementary file 1 — Supplementary Information 1. [file 41598_2020_80576_MOESM1_ESM.zip › S1/Aggregate counts/day5/0mmHg Jan10 41 39/ML SS1 3-028_2019-02-19_112522.bmp]

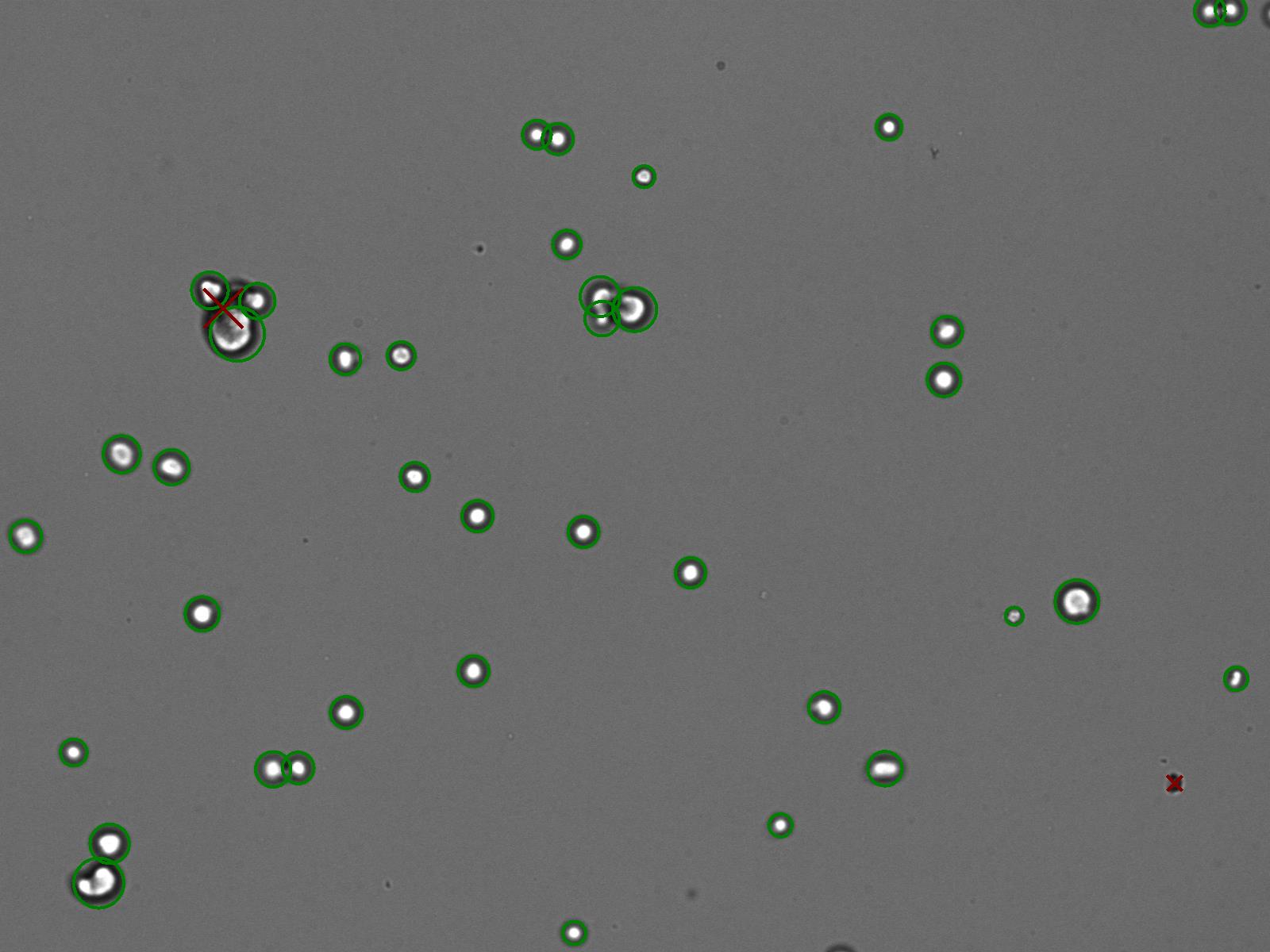

Supplement: Supplementary file 1 — Supplementary Information 1. [file 41598_2020_80576_MOESM1_ESM.zip › S1/Aggregate counts/day5/0mmHg Jan10 41 39/ML SS1 3-029_2019-02-19_112522.bmp]

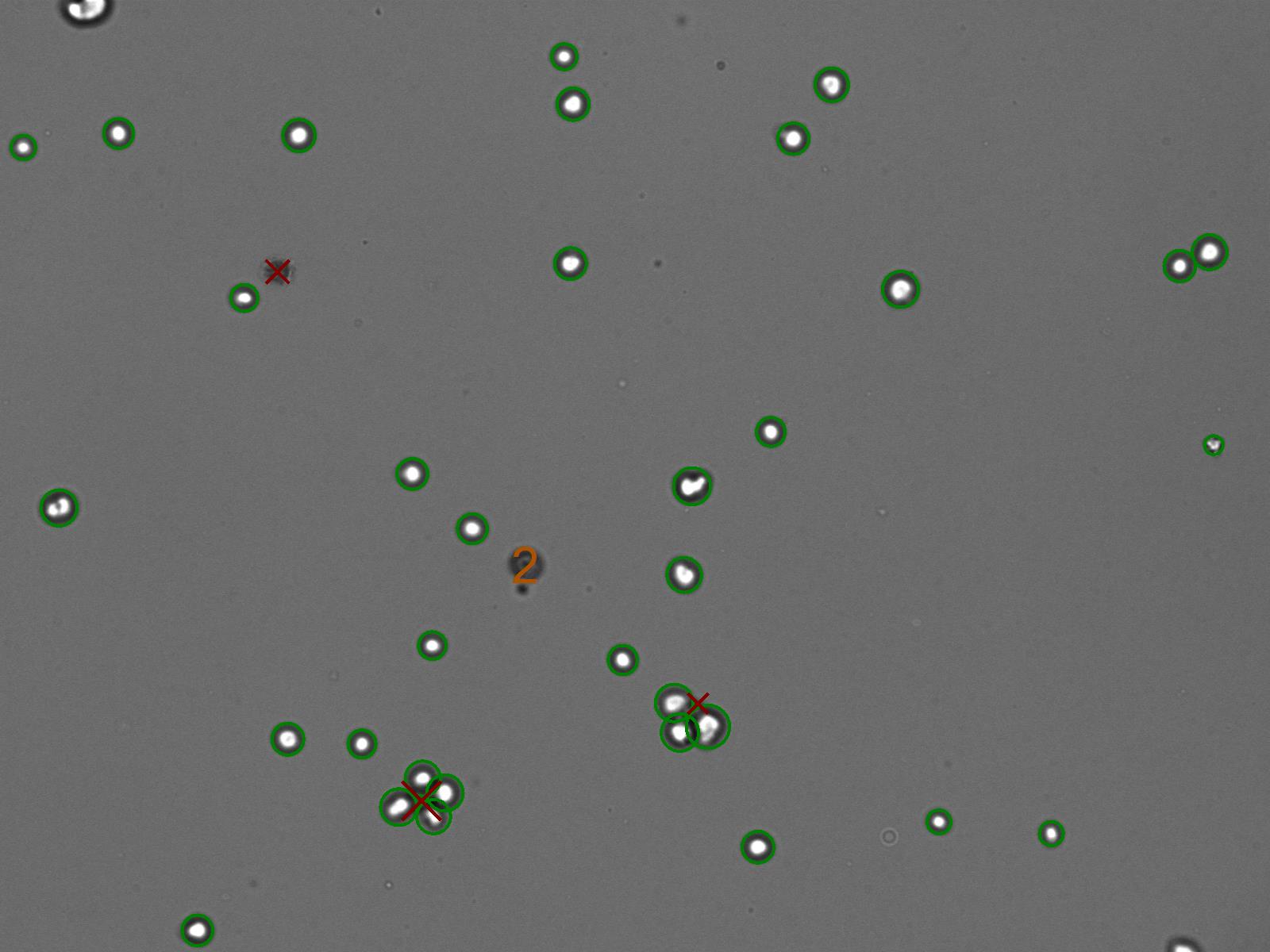

Supplement: Supplementary file 1 — Supplementary Information 1. [file 41598_2020_80576_MOESM1_ESM.zip › S1/Aggregate counts/day5/0mmHg Jan10 41 39/ML SS1 3-030_2019-02-19_112523.bmp]

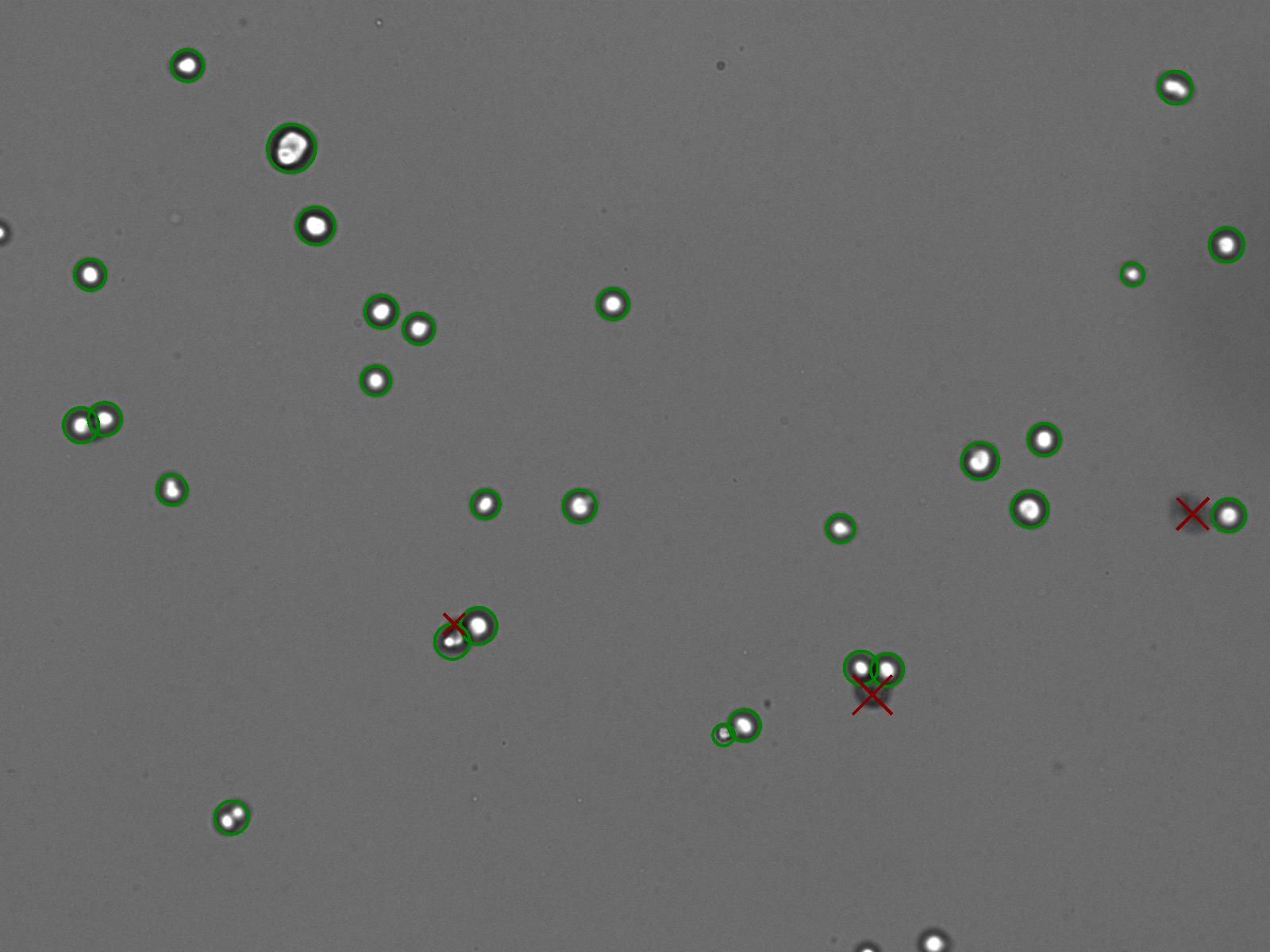

Supplement: Supplementary file 1 — Supplementary Information 1. [file 41598_2020_80576_MOESM1_ESM.zip › S1/Aggregate counts/day5/0mmHg Jan10 41 39/ML SS1 3-031_2019-02-19_112523.bmp]

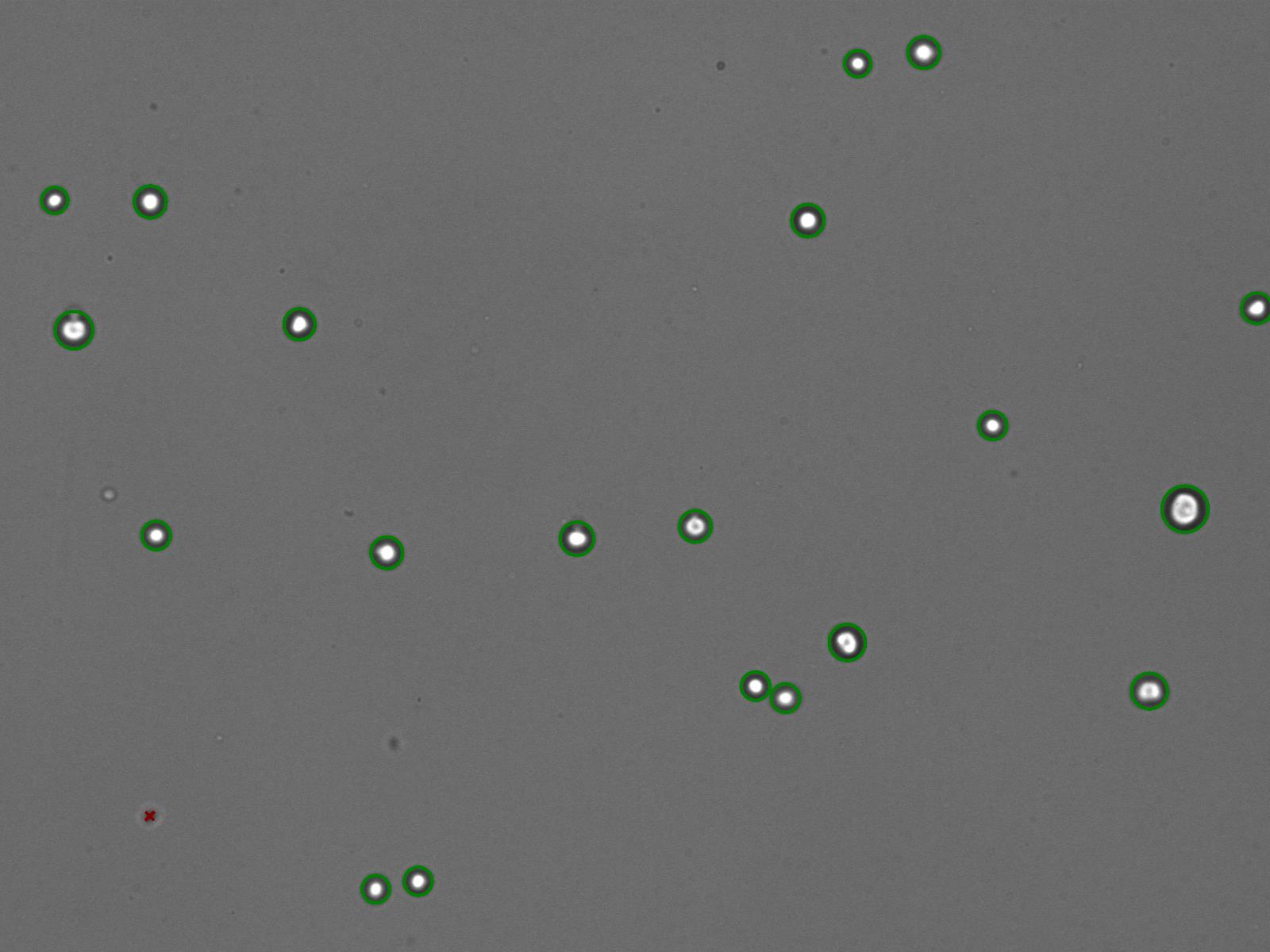

Supplement: Supplementary file 1 — Supplementary Information 1. [file 41598_2020_80576_MOESM1_ESM.zip › S1/Aggregate counts/day5/0mmHg Jan10 41 39/ML SS1 3-032_2019-02-19_112523.bmp]

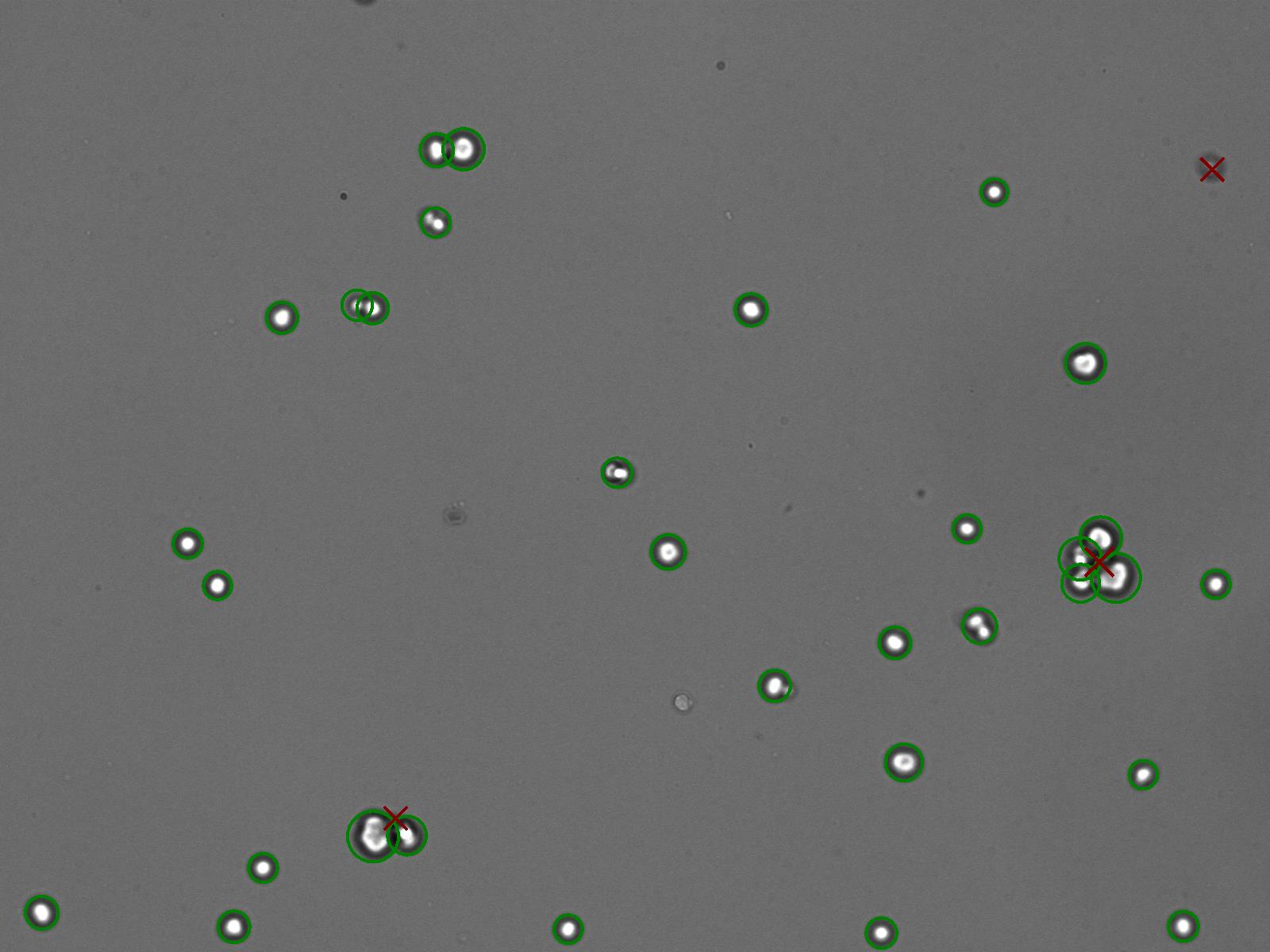

Supplement: Supplementary file 1 — Supplementary Information 1. [file 41598_2020_80576_MOESM1_ESM.zip › S1/Aggregate counts/day5/0mmHg Jan10 41 39/ML SS1 3-033_2019-02-19_112523.bmp]

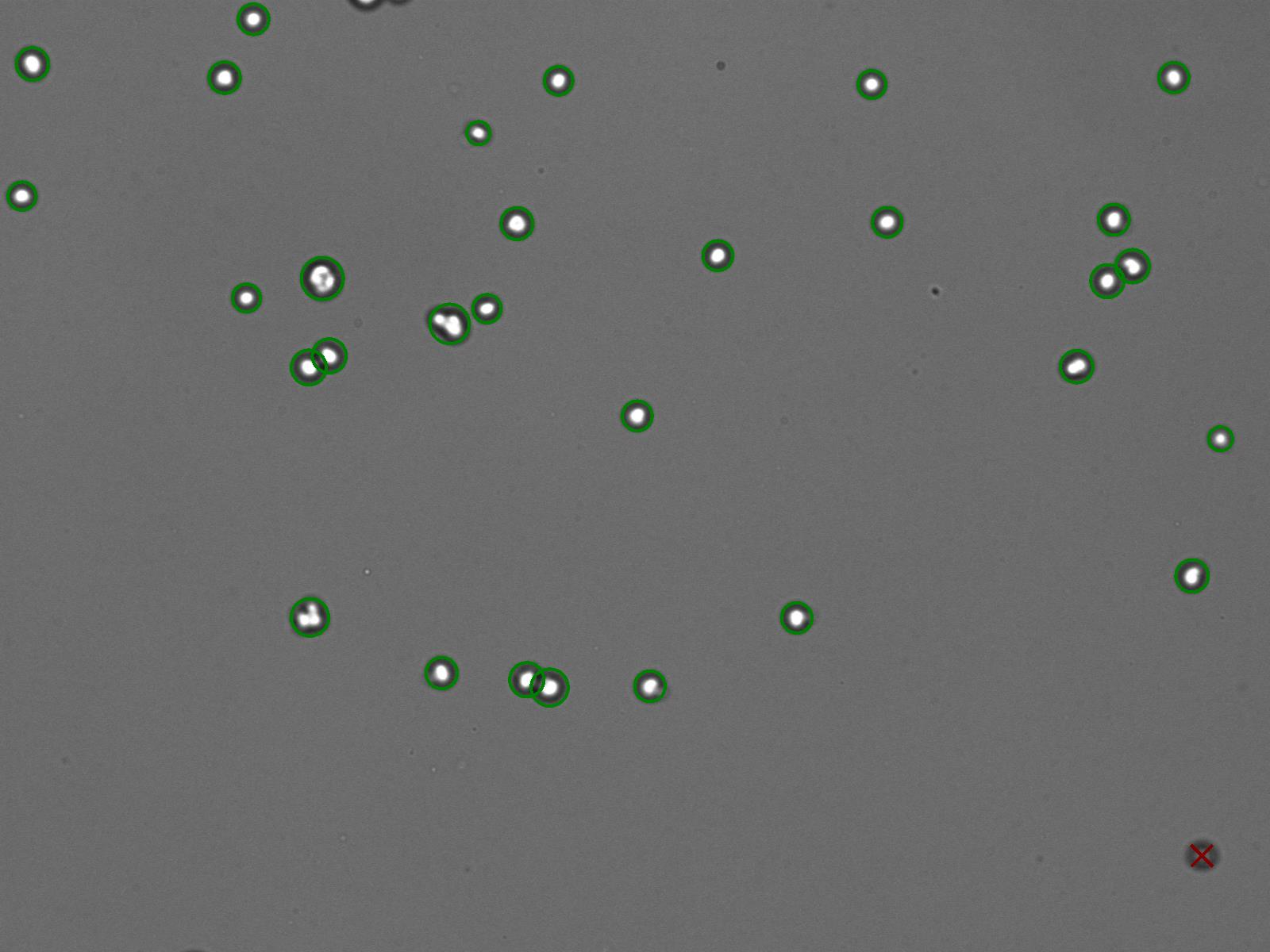

Supplement: Supplementary file 1 — Supplementary Information 1. [file 41598_2020_80576_MOESM1_ESM.zip › S1/Aggregate counts/day5/0mmHg Jan10 41 39/ML SS1 3-034_2019-02-19_112524.bmp]

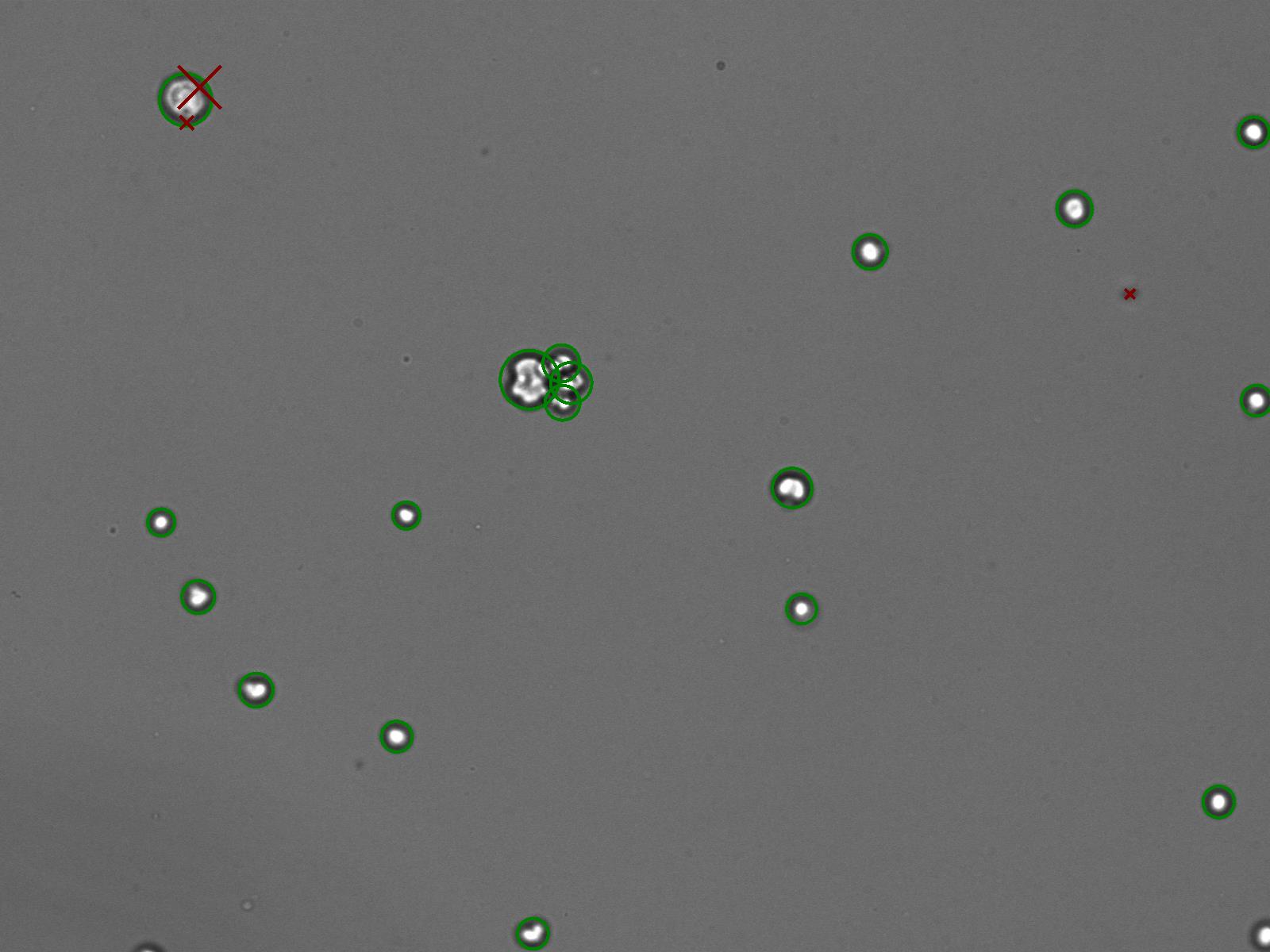

Supplement: Supplementary file 1 — Supplementary Information 1. [file 41598_2020_80576_MOESM1_ESM.zip › S1/Aggregate counts/day5/0mmHg Jan10 41 39/ML SS1 3-035_2019-02-19_112524.bmp]

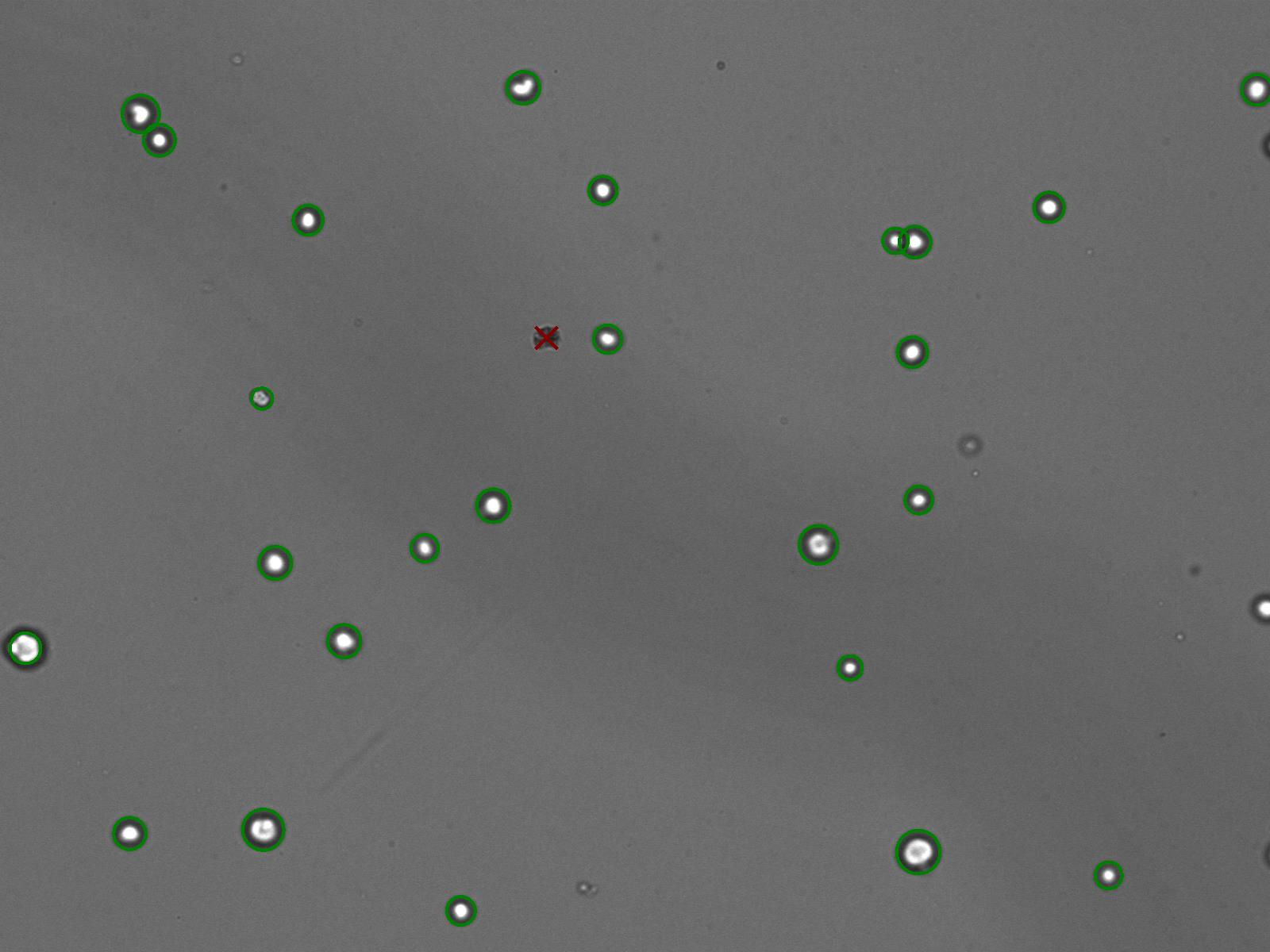

Supplement: Supplementary file 1 — Supplementary Information 1. [file 41598_2020_80576_MOESM1_ESM.zip › S1/Aggregate counts/day5/0mmHg Jan10 41 39/ML SS1 3-036_2019-02-19_112525.bmp]

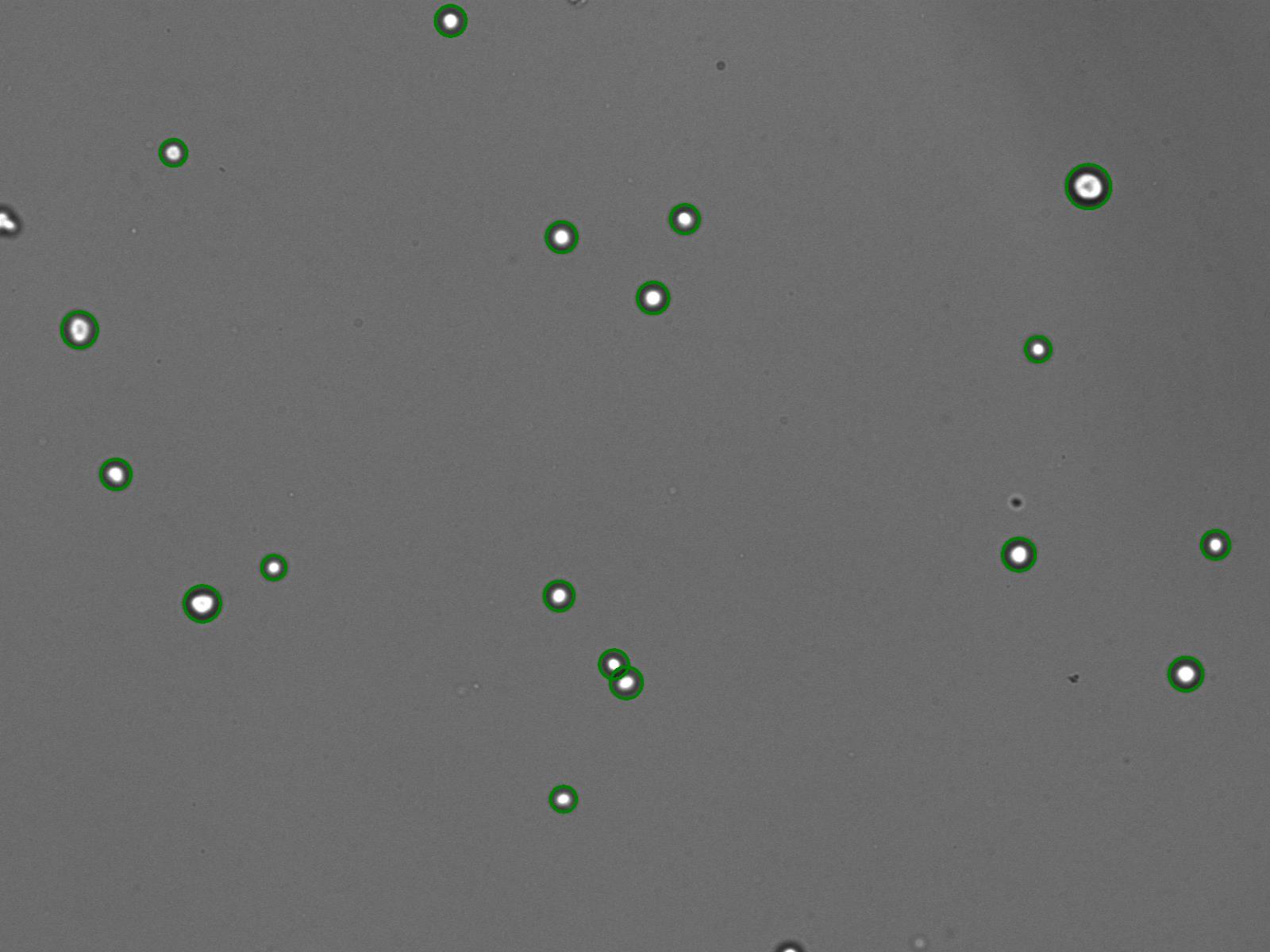

Supplement: Supplementary file 1 — Supplementary Information 1. [file 41598_2020_80576_MOESM1_ESM.zip › S1/Aggregate counts/day5/0mmHg Jan10 41 39/ML SS1 3-037_2019-02-19_112525.bmp]

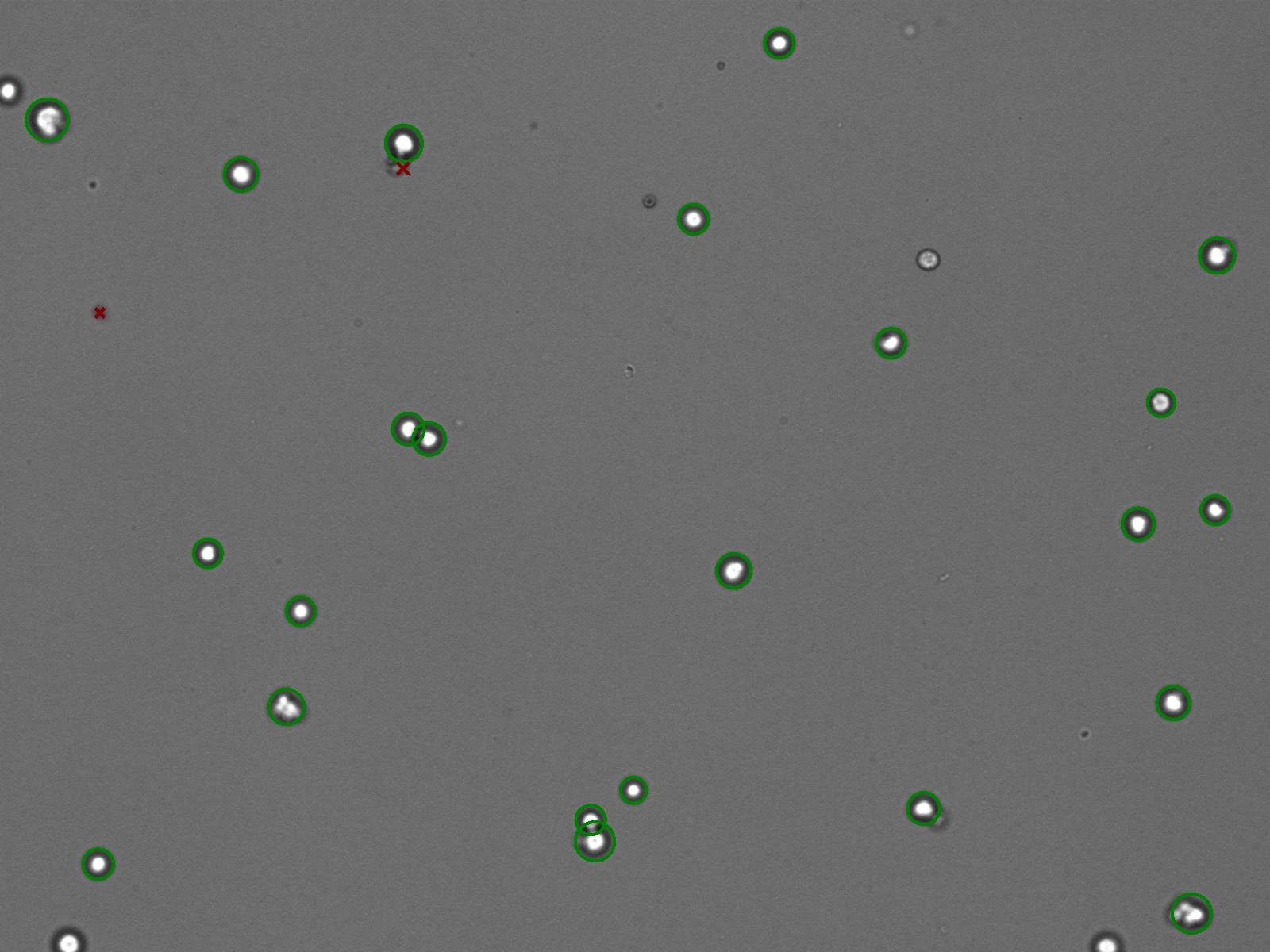

Supplement: Supplementary file 1 — Supplementary Information 1. [file 41598_2020_80576_MOESM1_ESM.zip › S1/Aggregate counts/day5/0mmHg Jan10 41 39/ML SS1 3-038_2019-02-19_112525.bmp]

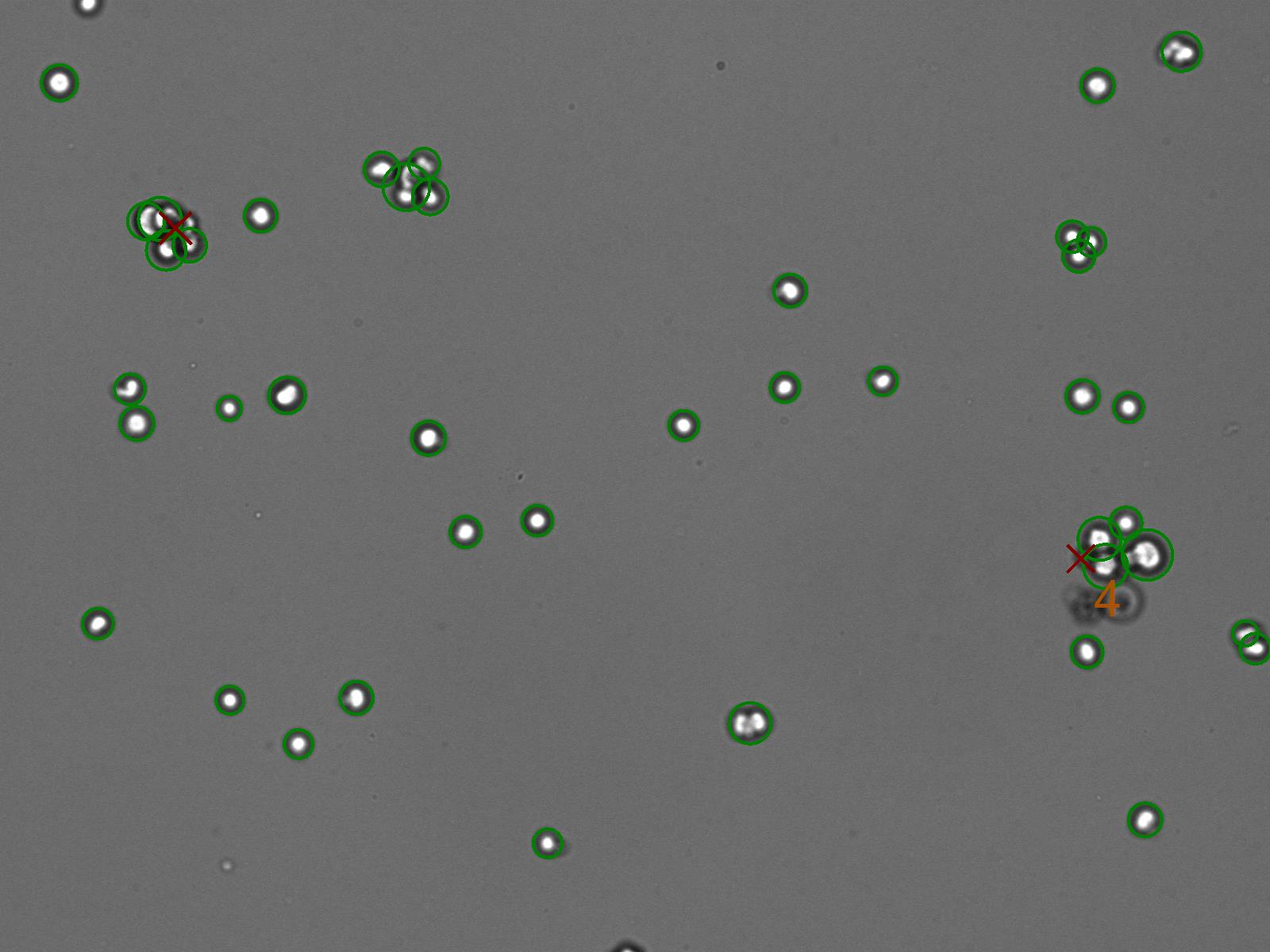

Supplement: Supplementary file 1 — Supplementary Information 1. [file 41598_2020_80576_MOESM1_ESM.zip › S1/Aggregate counts/day5/0mmHg Jan10 41 39/ML SS1 3-039_2019-02-19_112525.bmp]

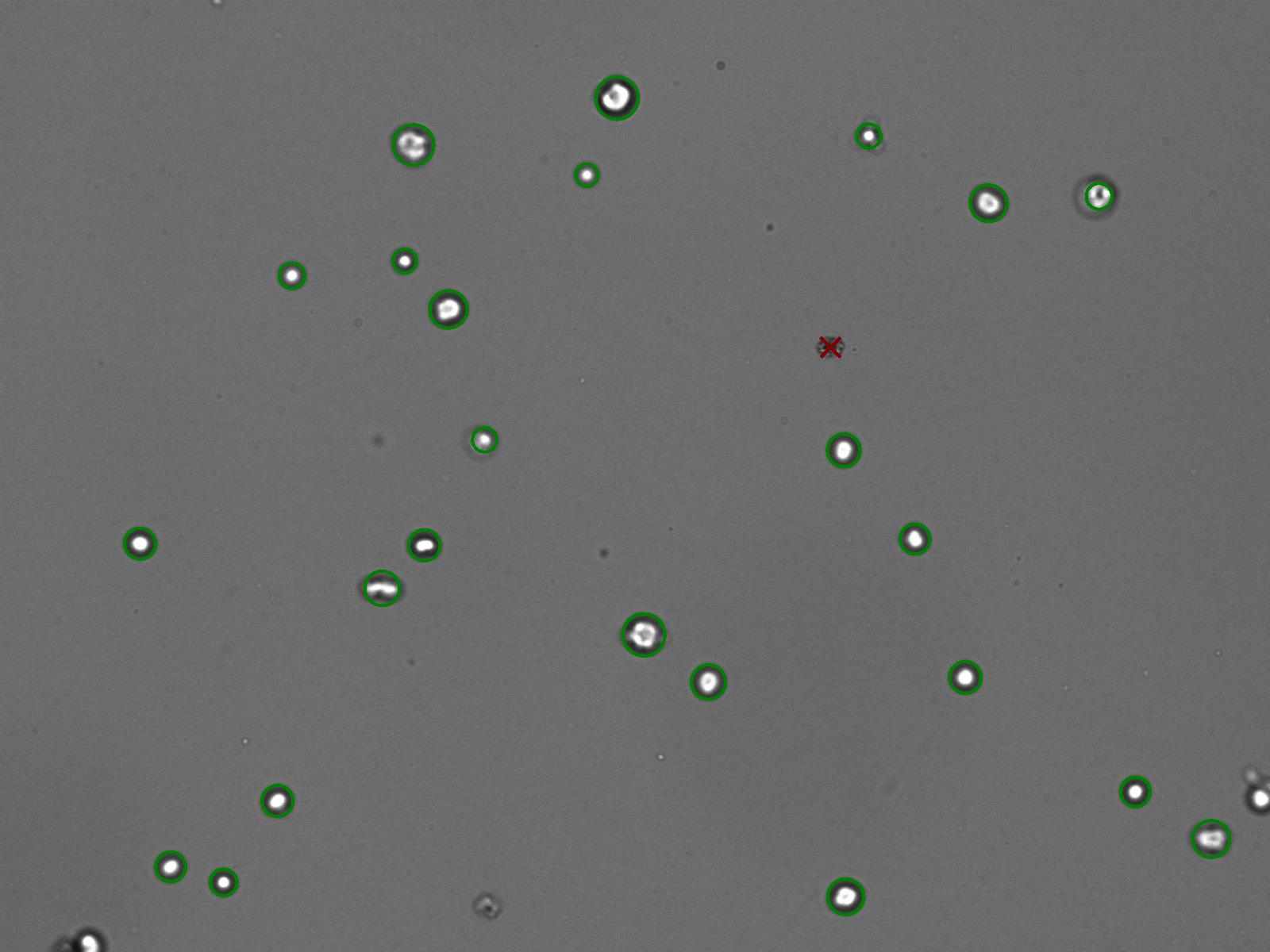

Supplement: Supplementary file 1 — Supplementary Information 1. [file 41598_2020_80576_MOESM1_ESM.zip › S1/Aggregate counts/day5/0mmHg Jan10 41 39/ML SS1 3-040_2019-02-19_112526.bmp]

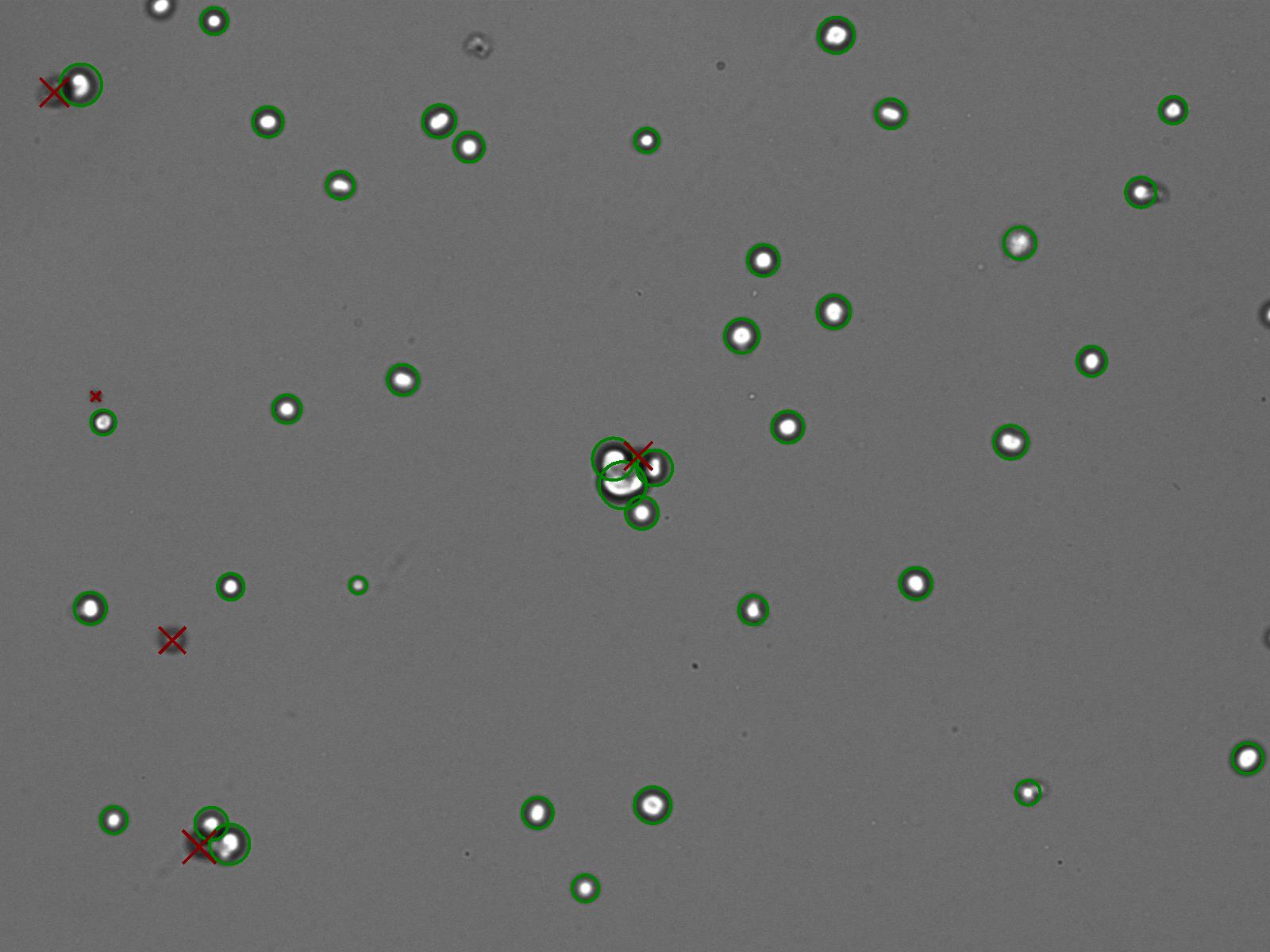

Supplement: Supplementary file 1 — Supplementary Information 1. [file 41598_2020_80576_MOESM1_ESM.zip › S1/Aggregate counts/day5/0mmHg Jan10 41 39/ML SS1 3-041_2019-02-19_112526.bmp]

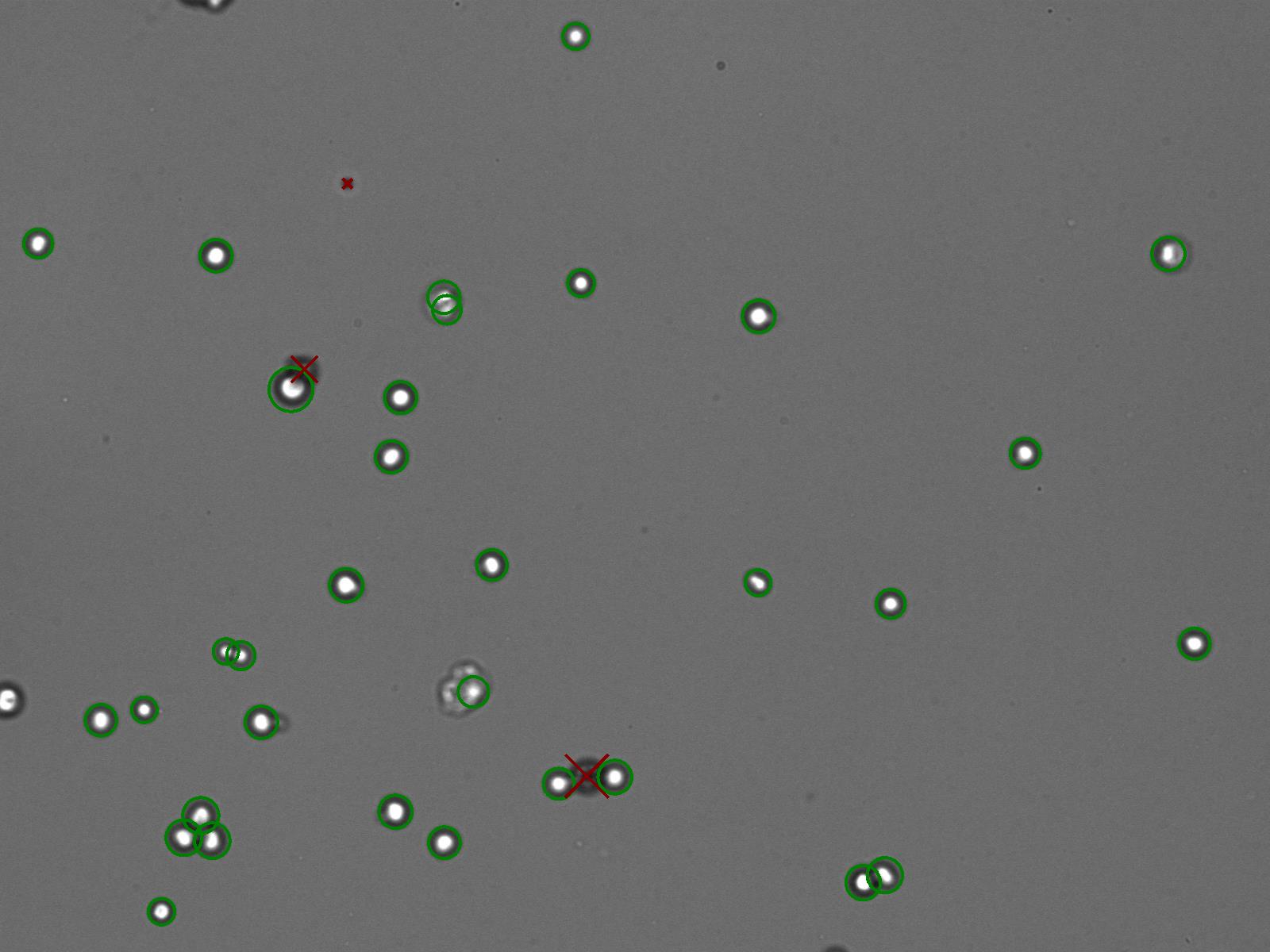

Supplement: Supplementary file 1 — Supplementary Information 1. [file 41598_2020_80576_MOESM1_ESM.zip › S1/Aggregate counts/day5/0mmHg Jan10 41 39/ML SS1 3-042_2019-02-19_112526.bmp]

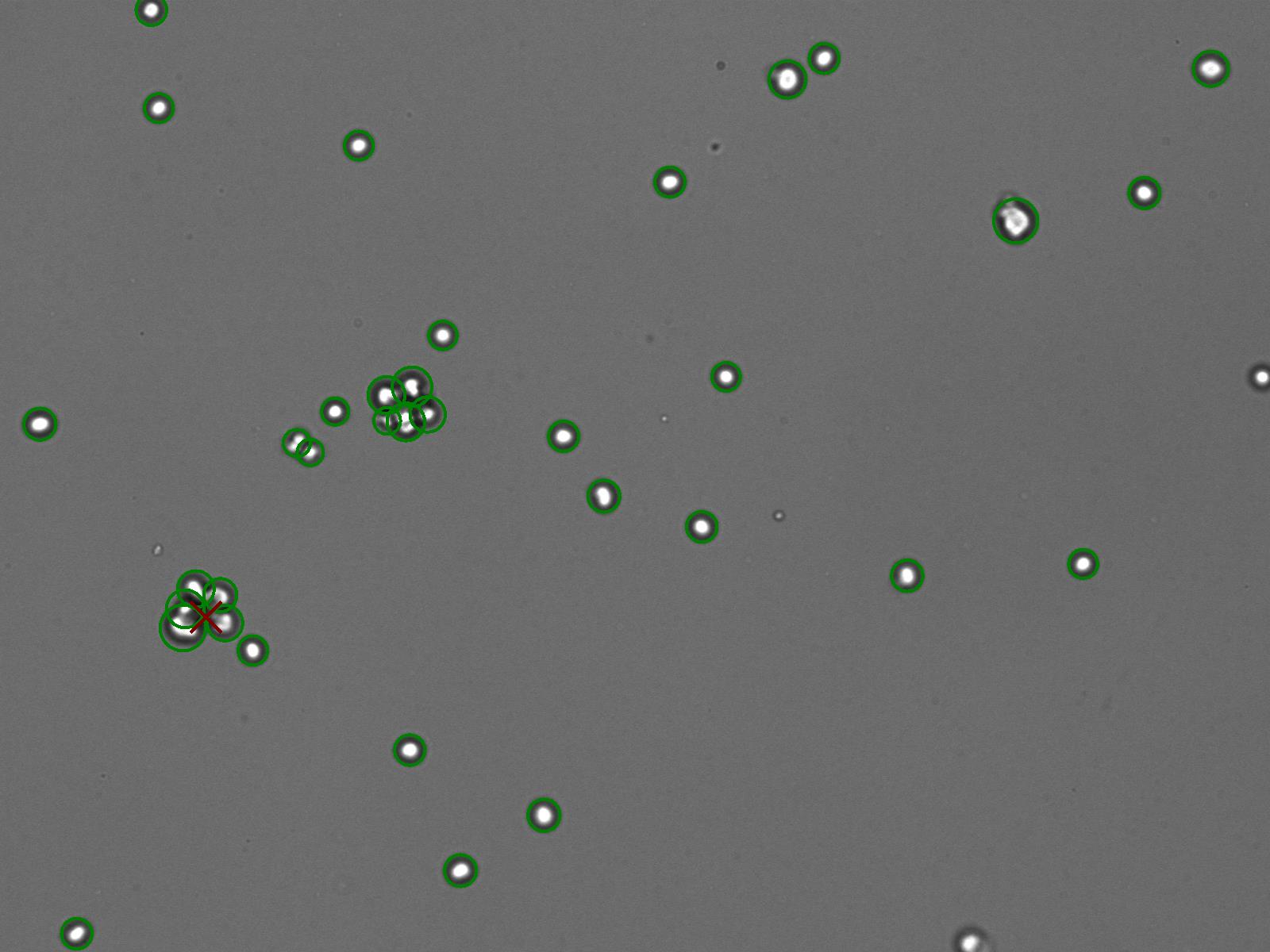

Supplement: Supplementary file 1 — Supplementary Information 1. [file 41598_2020_80576_MOESM1_ESM.zip › S1/Aggregate counts/day5/0mmHg Jan10 41 39/ML SS1 3-043_2019-02-19_112527.bmp]

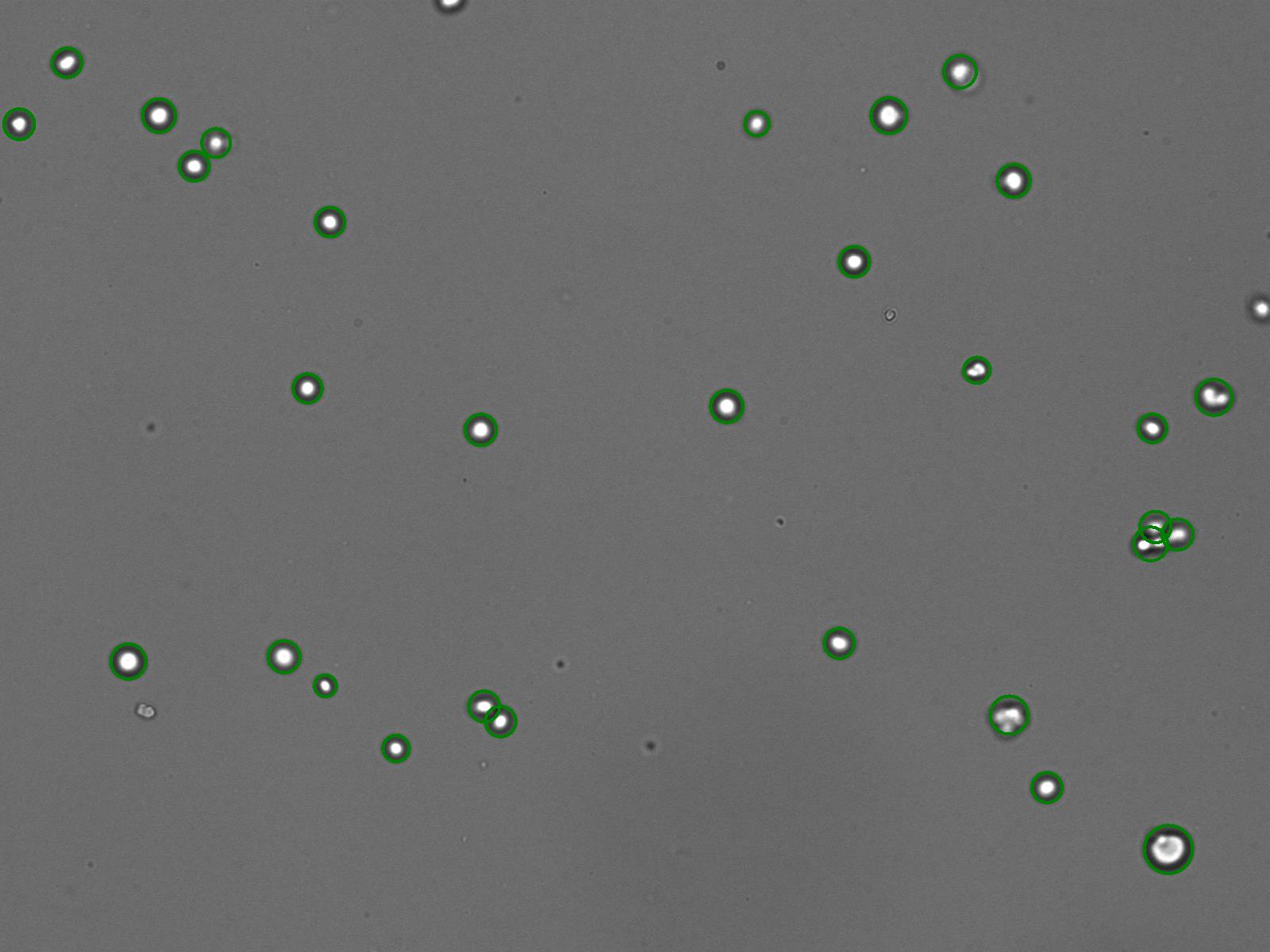

Supplement: Supplementary file 1 — Supplementary Information 1. [file 41598_2020_80576_MOESM1_ESM.zip › S1/Aggregate counts/day5/0mmHg Jan10 41 39/ML SS1 3-044_2019-02-19_112527.bmp]

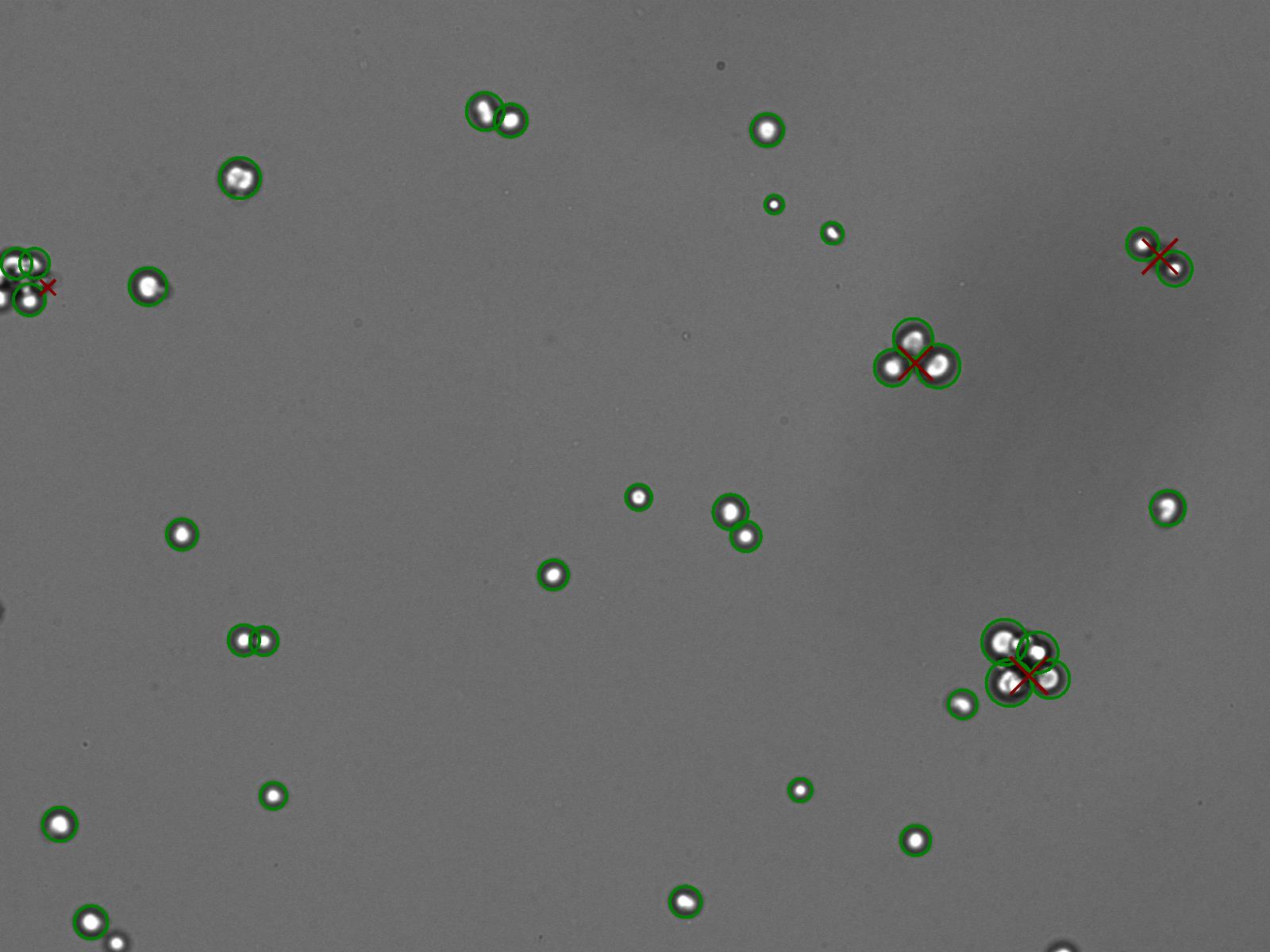

Supplement: Supplementary file 1 — Supplementary Information 1. [file 41598_2020_80576_MOESM1_ESM.zip › S1/Aggregate counts/day5/0mmHg Jan10 41 39/ML SS1 3-045_2019-02-19_112527.bmp]

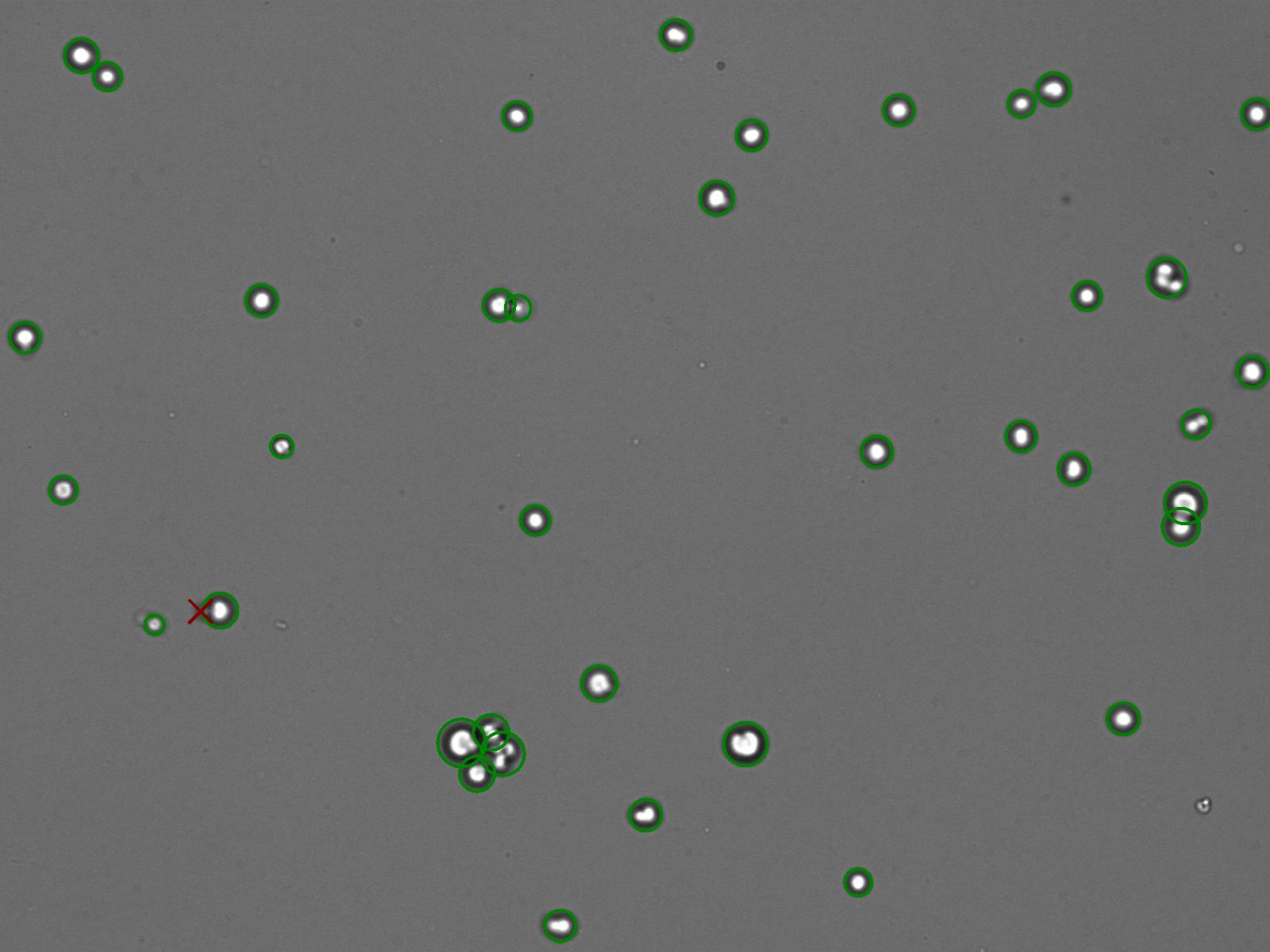

Supplement: Supplementary file 1 — Supplementary Information 1. [file 41598_2020_80576_MOESM1_ESM.zip › S1/Aggregate counts/day5/0mmHg Jan10 41 39/ML SS1 3-046_2019-02-19_112528.bmp]

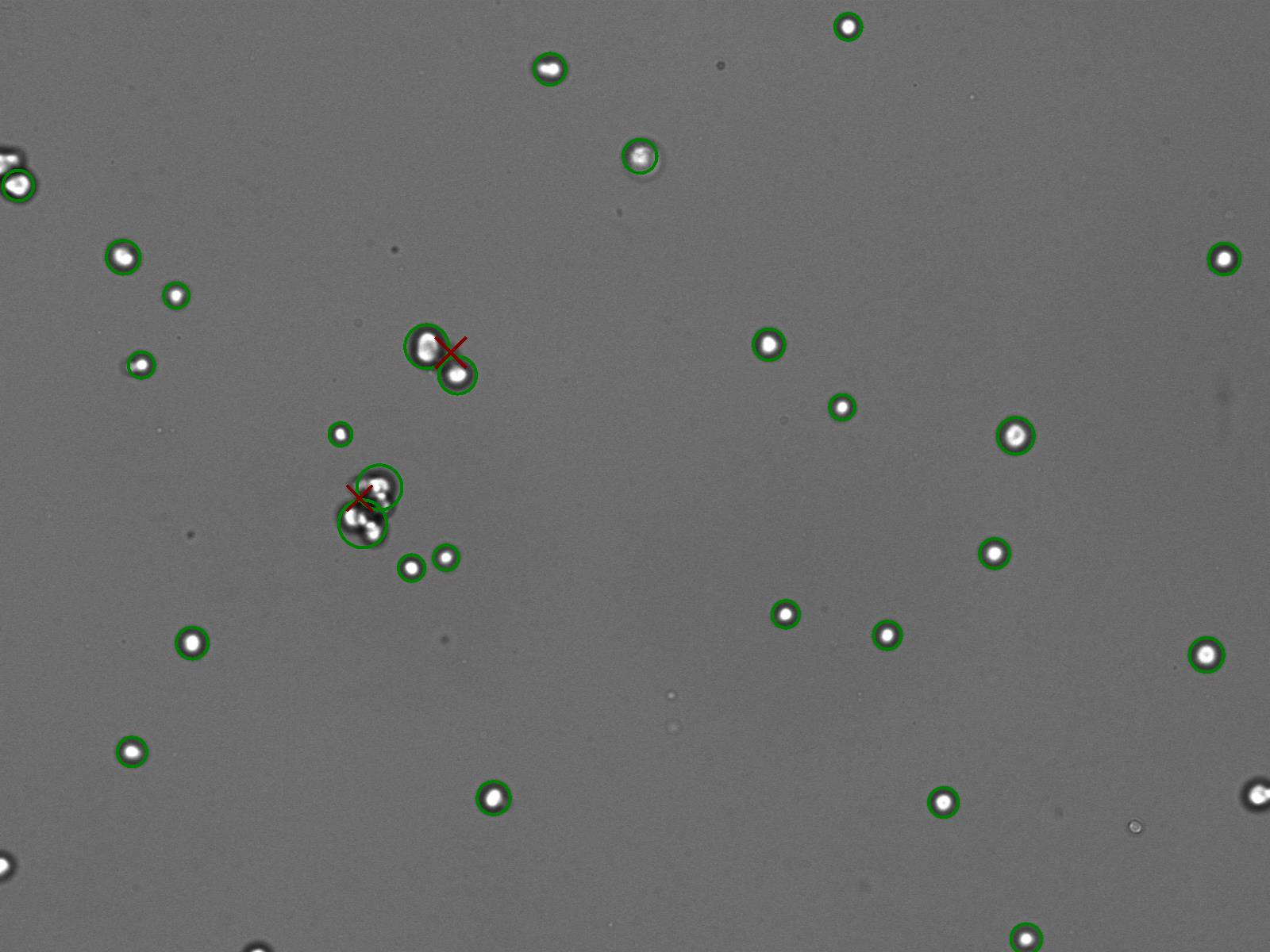

Supplement: Supplementary file 1 — Supplementary Information 1. [file 41598_2020_80576_MOESM1_ESM.zip › S1/Aggregate counts/day5/0mmHg Jan10 41 39/ML SS1 3-047_2019-02-19_112528.bmp]

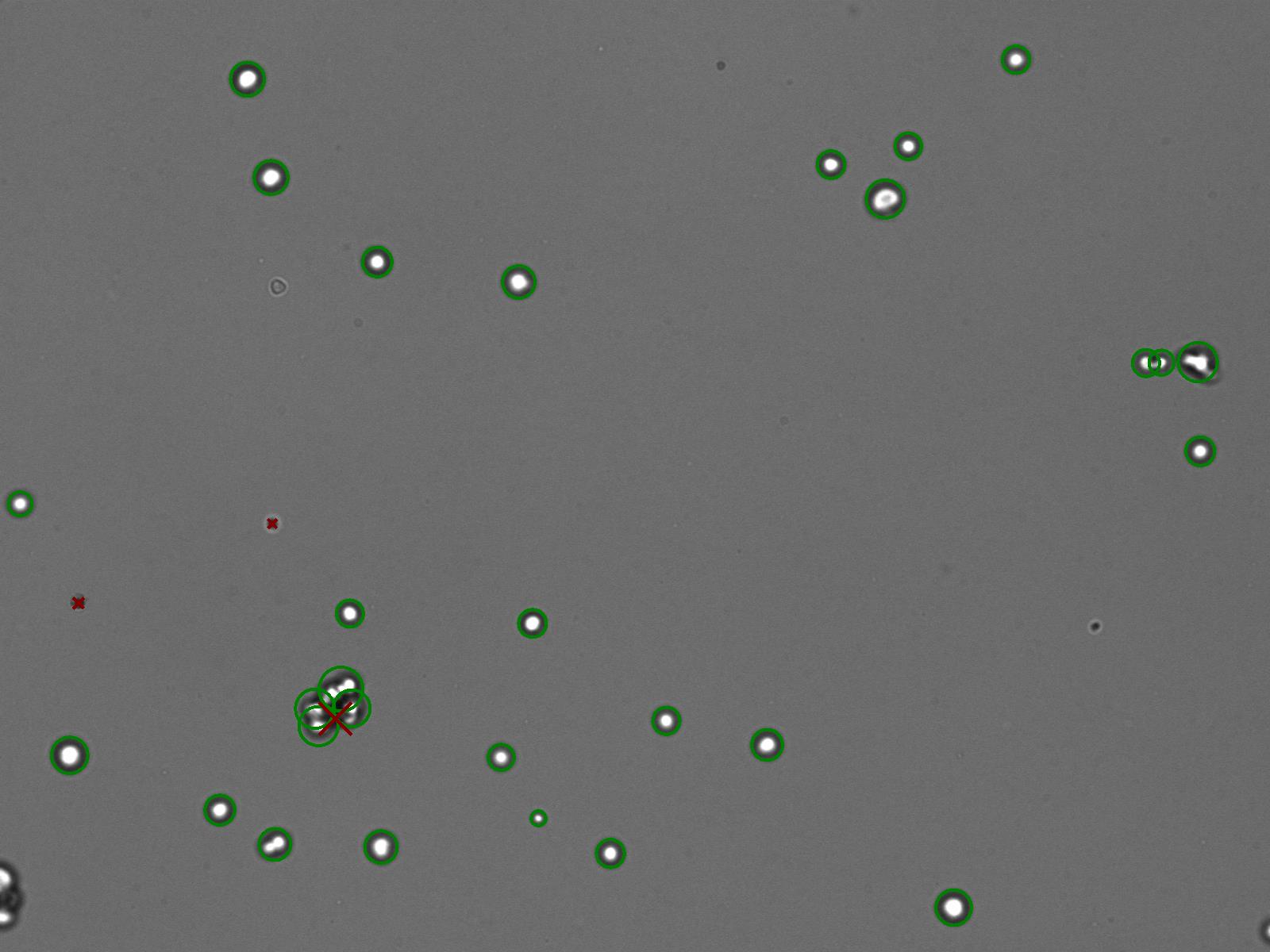

Supplement: Supplementary file 1 — Supplementary Information 1. [file 41598_2020_80576_MOESM1_ESM.zip › S1/Aggregate counts/day5/0mmHg Jan10 41 39/ML SS1 3-048_2019-02-19_112528.bmp]

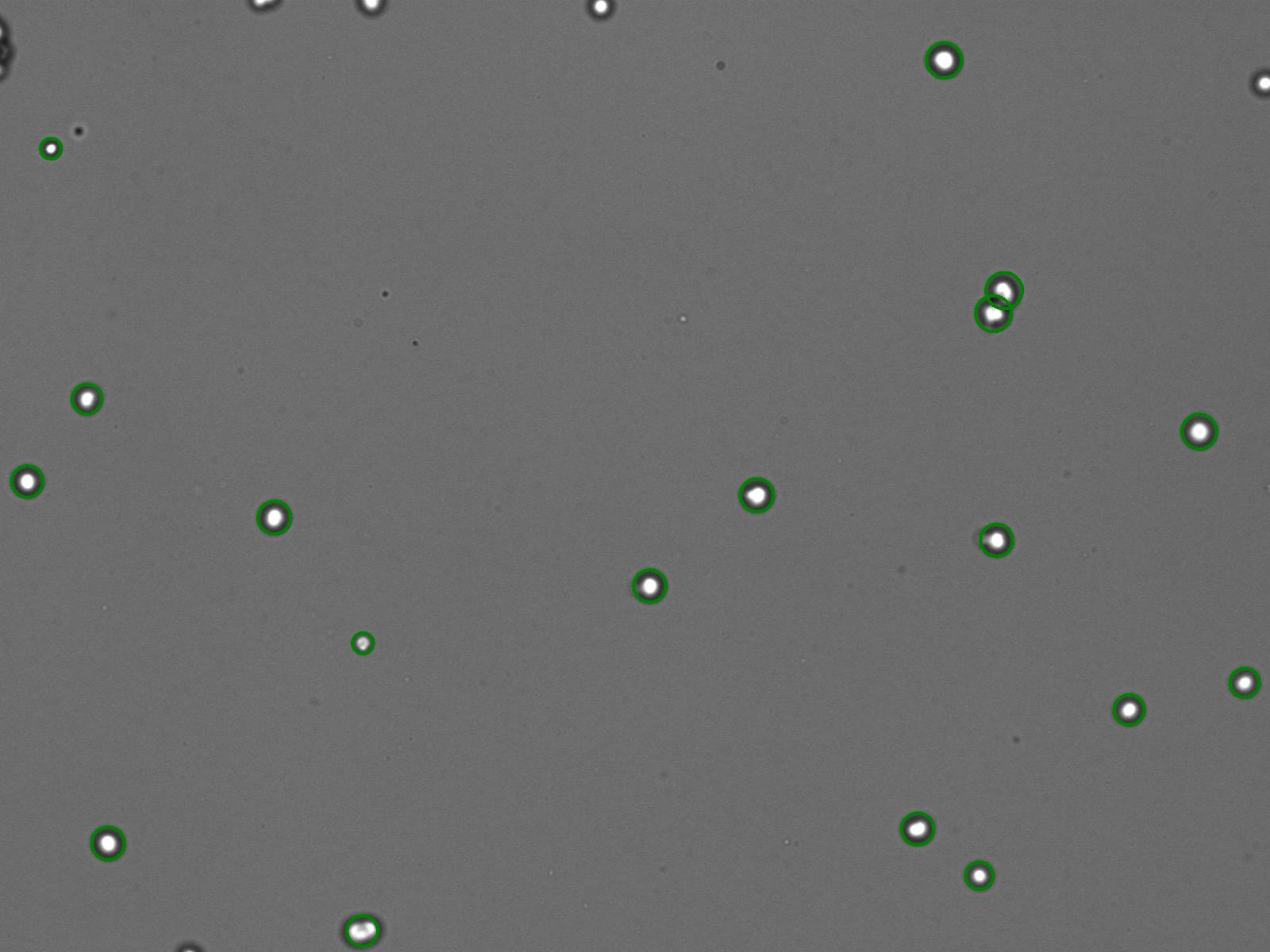

Supplement: Supplementary file 1 — Supplementary Information 1. [file 41598_2020_80576_MOESM1_ESM.zip › S1/Aggregate counts/day5/0mmHg Jan10 41 39/ML SS1 3-049_2019-02-19_112529.bmp]
