# Supplementary material for: Investigating the influence of physiologically relevant hydrostatic pressure on CHO cell batch culture
Source: Sci Rep. 2021 Jan 8;11:162. doi: 10.1038/s41598-020-80576-8 (PMC7794228; doi:10.1038/s41598-020-80576-8)
Supplement: Supplementary file 2 — Supplementary Information 2. [file 41598_2020_80576_MOESM2_ESM.docx]

**Supplementary Information**

**Investigating the influence of physiologically relevant hydrostatic pressure on CHO cell batch culture**

Menglin Shang^1,3^, Taehong Kwon^8^, Jean-Francois P. Hamel^7^, Chwee Teck Lim^1,2,3,4,9^, Bee Luan Khoo^5^ and Jongyoon Han*^1,4,6,8^

^1^Critical Analytics for Manufacturing Personalized-Medicine (CAMP) IRG, Singapore-MIT Alliance for Research and Technology (SMART) Centre, Singapore

^2^Mechanobiology Institute, National University of Singapore, Singapore

^3^Department of Biomedical Engineering, National University of Singapore, 7 Engineering Drive 1, Singapore 117574

^4^Department of Mechanical Engineering, National University of Singapore, Singapore

^5^Department of Biomedical Engineering, City University of Hong Kong, Hong Kong

^6^Department of Electrical Engineering and Computer Science, Department of Biological Engineering, Massachusetts Institute of Technology, Cambridge, Massachusetts, USA

^7^Department of Chemical Engineering, Massachusetts Institute of Technology, Cambridge, Massachusetts, USA

^8^Research Laboratory of Electronics, Massachusetts Institute of Technology, Cambridge, Massachusetts, USA

^9^Institute for Health Innovation and Technology, 14 Medical Drive, Singapore 117599


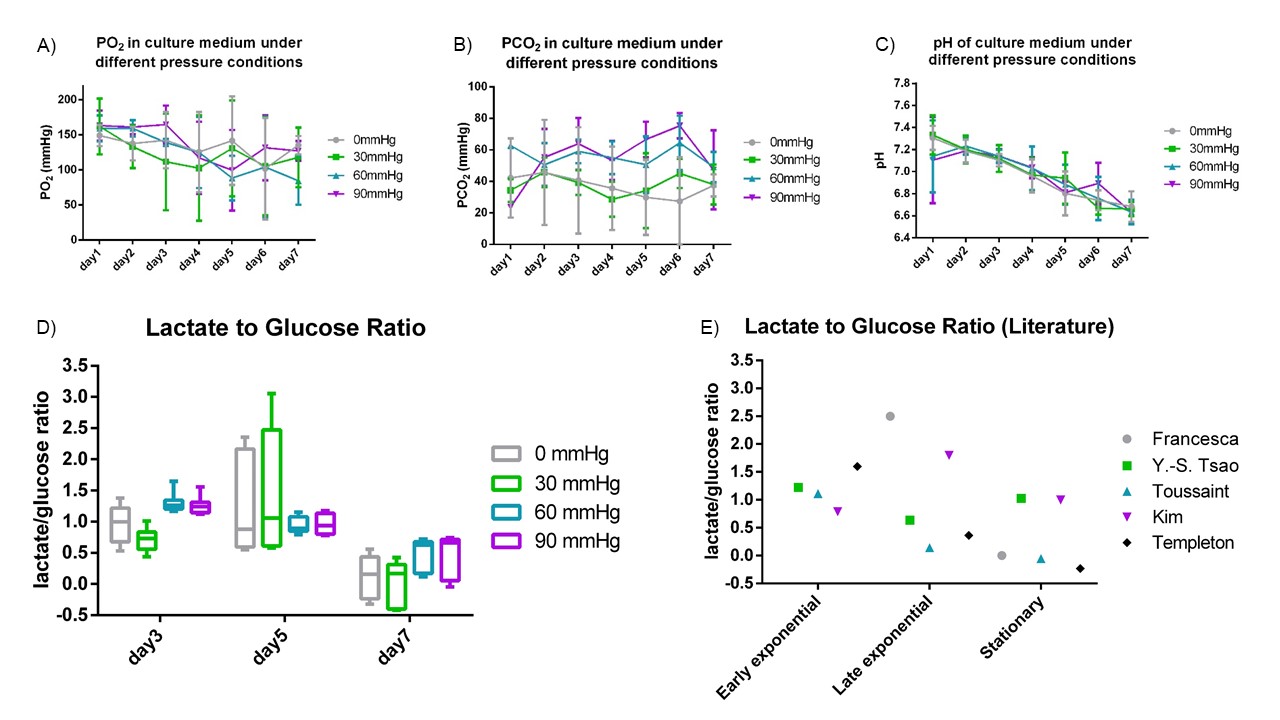


**Figure S1 Additional FLEX2 (automated cell culture analyzer) measurements of the CHO cell culture medium. A)** Oxygen partial pressure (pO_2_) in the culture medium. **B)** Carbon dioxide partial pressure (pCO_2_) in the culture medium. **C)** pH of the culture medium. **D)** Lactate/glucose ratio of CHO cells under different pressure conditions on Day 3, Day 5, and Day 7. **E)** Lactate/glucose ratio from literature.


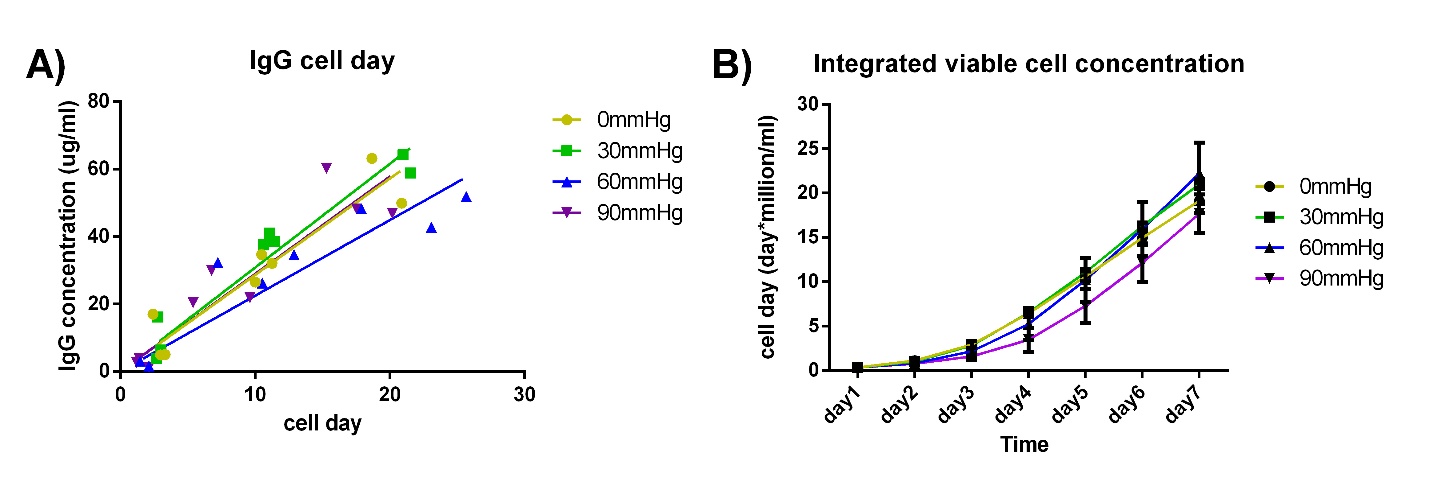


**Figure S2 Additional information about IgG_1_ production and cell concentration change under pressure. A)** The plot of IgG_1_ production over IVC. **B)** IVC under different pressure conditions.


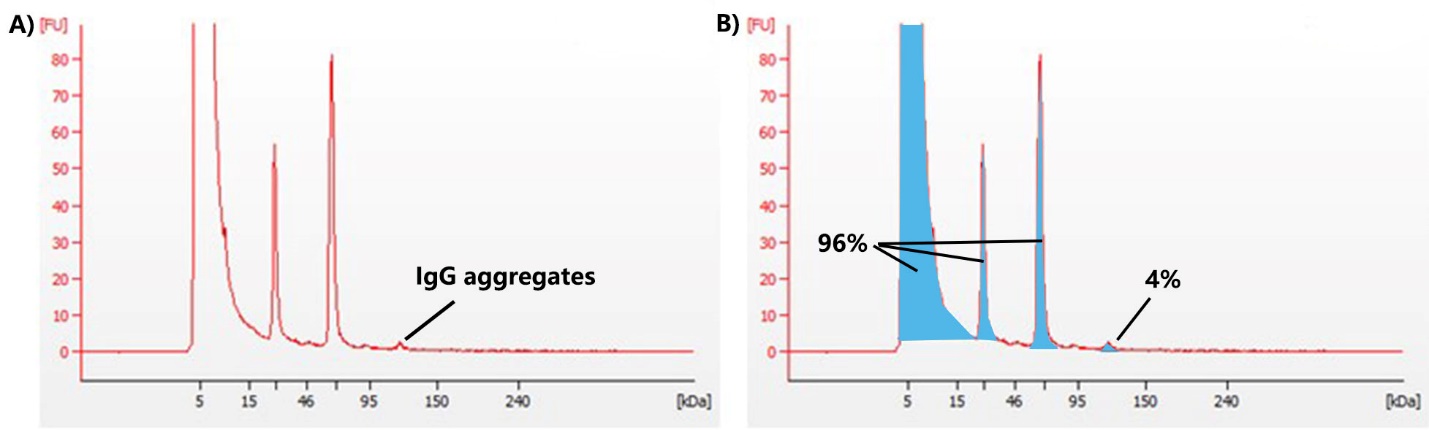


**Figure S3 IgG_1_ aggregate analysis. A)** The representative analytical result with an indication of non-disulphide-linked IgG_1_ aggregate. **B)** Illustration of the area under peaks.

**Table S1 Tabulated P-values for viable cell number, cell viability and cell diameter.** Sample data was compared with a value under 0 mmHg at the same sampling time.

|  | **Viable cell number** | | | **Cell viability** | | | **Cell diameter** | | |
| --- | --- | --- | --- | --- | --- | --- | --- | --- | --- |
| **Day** | **30**  **mmHg** | **60**  **mmHg** | **90**  **mmHg** | **30**  **mmHg** | **60**  **mmHg** | **90**  **mmHg** | **30**  **mmHg** | **60**  **mmHg** | **90**  **mmHg** |
| **1** | 3.72E-01 | 3.12E-01 | 3.72E-01 | 3.66E-01 | 1.13E-01 | 3.66E-01 | 7.46E-02 | 5.06E-02 | 7.46E-02 |
| **3** | 4.87E-01 | 2.73E-02 | 2.84E-06 | 4.51E-01 | 6.49E-02 | 1.87E-01 | 4.73E-01 | 3.94E-01 | 1.91E-01 |
| **7** | 3.65E-02 | 1.81E-06 | 5.70E-05 | 5.62E-02 | 1.09E-03 | 8.20E-04 | 4.29E-01 | 6.10E-02 | 7.90E-02 |

**Table S2 Tabulated P-values for Glucose, Lactate and Ammonium concentration change.** Sample data was compared with a value under 0 mmHg at the same sampling time

| **Day** | **30**  **mmHg** | **60**  **mmHg** | **90**  **mmHg** | **30**  **mmHg** | **60**  **mmHg** | **90**  **mmHg** | **30**  **mmHg** | **60**  **mmHg** | **90**  **mmHg** |
| --- | --- | --- | --- | --- | --- | --- | --- | --- | --- |
|  | **Glucose concentration** | | | **Lactate concentration** | | | **Ammonium concentration** | | |
| **2** | 3.96E-01 | 9.01E-02 | 1.34E-03 | 2.94E-01 | 3.79E-01 | 8.41E-02 | 3.83E-01 | 1.78E-02 | 4.32E-03 |
| **3** | 6.51E-02 | 2.09E-03 | 2.07E-08 | 3.38E-01 | 1.68E-01 | 8.72E-02 | 1.50E-01 | 1.12E-04 | 8.34E-07 |
| **4** | 1.52E-01 | 4.28E-02 | 4.47E-05 | 4.88E-01 | 2.97E-01 | 2.29E-02 | 1.11E-01 | 1.20E-03 | 3.68E-06 |
| **5** | 4.39E-01 | 3.64E-01 | 3.54E-02 | 3.51E-01 | 2.27E-01 | 2.26E-01 | 1.34E-01 | 1.01E-02 | 1.03E-03 |
| **6** | 3.95E-01 | 2.69E-01 | 1.70E-01 | 4.97E-01 | 1.89E-01 | 4.54E-01 | 1.43E-01 | 2.69E-01 | 1.25E-02 |
| **7** | 3.84E-01 | 2.93E-01 | 2.27E-01 | 4.37E-01 | 4.94E-03 | 8.54E-02 | 3.32E-01 | 1.39E-02 | 3.77E-01 |
|  | **Glucose consumption rate** | | | **Lactate production rate** | | | **Ammonium production rate** | | |
| **2** | 5.35E-02 | 3.56E-01 | 3.25E-01 | 3.58E-01 | 6.62E-05 | 1.55E-03 | 2.66E-01 | 4.63E-01 | 4.54E-01 |
| **3** | 4.91E-01 | 6.46E-05 | 5.68E-04 | 4.73E-01 | 3.40E-01 | 8.82E-02 | 4.94E-01 | 5.17E-03 | 5.83E-04 |
| **4** | 3.57E-01 | 1.74E-04 | 4.97E-05 | 1.64E-01 | 4.29E-04 | 2.15E-07 | 1.30E-01 | 2.51E-01 | 1.58E-01 |
| **5** | 1.07E-01 | 6.91E-02 | 3.37E-02 | 1.21E-03 | 3.69E-01 | 4.25E-02 | 3.00E-01 | 6.43E-03 | 5.35E-02 |
| **6** | 2.58E-01 | 6.86E-02 | 1.33E-01 | 3.41E-01 | 1.02E-04 | 2.47E-02 | 4.05E-01 | 4.16E-02 | 2.69E-03 |
